# Design, Synthesis, *In Vitro* and *In Vivo* Characterization of CDC42 GTPase Interaction Inhibitors for the Treatment of Cancer

**DOI:** 10.1021/acs.jmedchem.3c00276

**Published:** 2023-04-07

**Authors:** Nicoletta Brindani, Linh M. Vuong, Isabella Maria Acquistapace, Maria Antonietta La Serra, José Antonio Ortega, Marina Veronesi, Sine Mandrup Bertozzi, Maria Summa, Stefania Girotto, Rosalia Bertorelli, Andrea Armirotti, Anand K. Ganesan, Marco De Vivo

**Affiliations:** † Molecular Modeling and Drug Discovery Lab, Istituto Italiano di Tecnologia, Via Morego 30, Genova 16163, Italy; ‡ Department of Dermatology, University of California, Irvine, California 92697, United States; § Analytical Chemistry Facility, Istituto Italiano di Tecnologia, Via Morego 30, Genova 16163, Italy; ∥ Structural Biophysics Facility, Istituto Italiano di Tecnologia, Via Morego 30, Genova 16163, Italy; ⊥ Translational Pharmacology Facility, Istituto Italiano di Tecnologia, Via Morego 30, Genova 16163, Italy

## Abstract

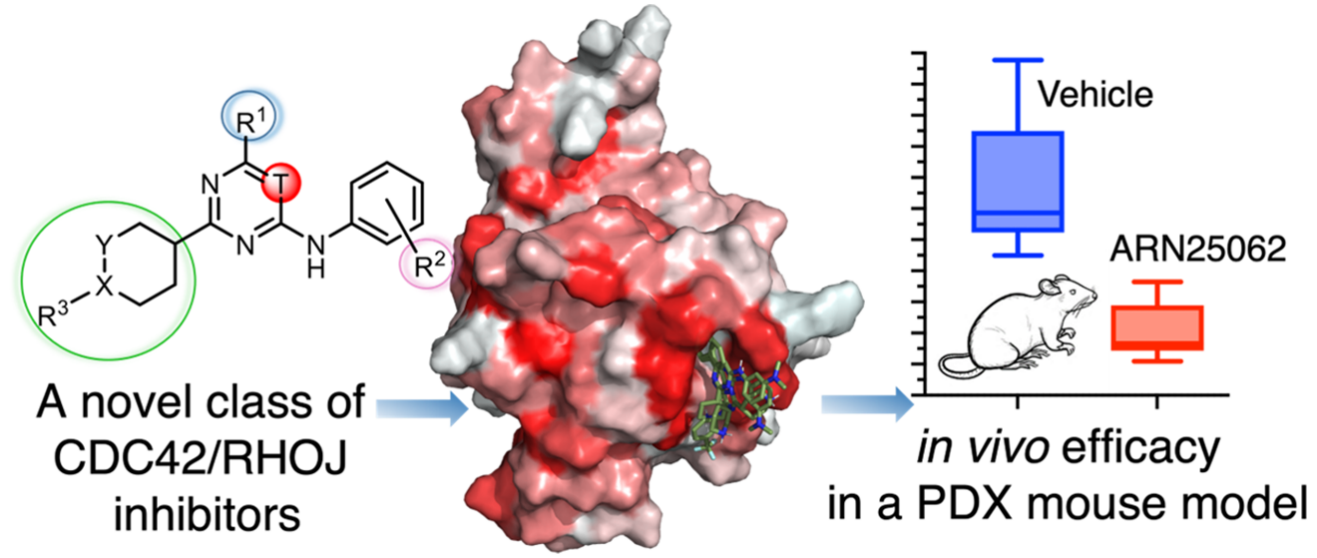

CDC42 GTPases (RHOJ, CDC42, and RHOQ) are overexpressed in multiple tumor types and activate pathways critical for tumor growth, angiogenesis, and metastasis. Recently, we reported the discovery of a novel lead compound, ARN22089, which blocks the interaction of CDC42 GTPases with specific downstream effectors. ARN22089 blocks tumor growth in BRAF mutant mouse melanoma models and patient-derived xenografts (PDXs) *in vivo*. ARN22089 also inhibits tumor angiogenesis in three-dimensional vascularized microtumor models *in vitro*. Notably, ARN22089 belongs to a novel class of trisubstituted pyrimidines. Based on these results, we describe an extensive structure–activity relationship of ∼30 compounds centered on ARN22089. We discovered and optimized two novel inhibitors (**27**, ARN25062, and **28**, ARN24928), which are optimal back-up/follow-up leads with favorable drug-like properties and *in vivo* efficacy in PDX tumors. These findings further demonstrate the potential of this class of CDC42/RHOJ inhibitors for cancer treatment, with lead candidates ready for advanced preclinical studies.

## Introduction

CDC42 GTPases (CDC42, RHOJ, and RHOQ) are small guanosine triphosphate (GTP)-binding proteins that are known to regulate tumor growth, angiogenesis, metastasis, and cell resistance to targeted therapies.^1−5^ CDC42 GTPases are essential molecular switches within the cell. Their active/inactive state depends on whether they are bound to GTP or guanosine diphosphate (GDP).^6^ When CDC42 GTPases are bound to GTP, they change their structural conformation, allowing protein surface interactions that are complementary to their downstream effectors. These include, but are not limited to, p21-activated protein kinases (PAKs).^7^ Notably, PAKs are known to be involved in invasion, migration, and oncogenic transformation.^8,9^ Many groups have sought to design small molecules that inhibit PAK kinases by targeting the large and flexible ATP binding pocket in the kinase domain or by targeting a large autoinhibitory region that is observed in group I PAKs (PAK1, PAK2, and PAK3).^10^ However, developed agents have failed to reach the clinic secondary to their poor selectivity. For example, existing PAK inhibitors act on multiple isoforms of PAKs, including PAK2, which is thought to induce cardiotoxicity with a narrow therapeutic window.^11^

**Figure 1:**
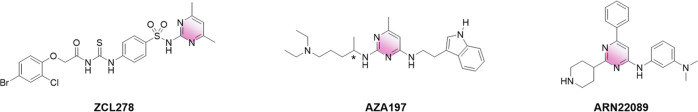
Examples of selective CDC42 inhibitors, previously reported: ZCL278, AZA197, and our lead ARN22089.^12^

CDC42 GTPases themselves have been previously considered “undruggable” due to their globular structure with the lack of alternative binding pockets to the active site, where there is high affinity for GTP or GDP.^1,12^ Despite these restrictions, one of the main approaches to targeting CDC42 involves designing agents that block CDC42 activation by targeting the CDC42-GEF protein–protein interaction interfaces.^13^ Several small molecules have been identified that inhibit CDC42 activation.^13−16^ Among them, ZCL278 and AZA197 (Figure 1) represent two notable examples that have been shown to inhibit CDC42 activation and reduce prostate and colon cancer migration and invasion.^17,18^ Unfortunately, many agents that target CDC42 activation also block activation of the GTPase RAC1,^13^ which can inhibit platelet aggregation, resulting in hematologic side effects.^19^

**Figure 2:**
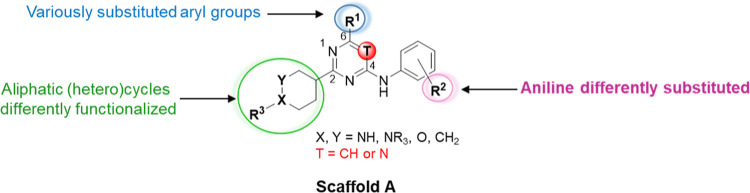
Representation of the points subject to chemical manipulation of scaffold A.

We recently discovered a new compound, ARN22089 (Figure 1), designed to specifically inhibit the interaction between CDC42 and its downstream kinase PAK. This molecule differs from the other small molecules that interfere with CDC42-GEF interaction.^1^ Notably, this compound does not block RAC1 effector interactions.^12^ In our initial disclosure of the compound ARN22089 (Figure 1), we reported its *in vitro* and *in vivo* efficacy and overall drug-like profile, with a brief description of the medicinal chemistry effort for the discovery and characterization of this novel lead compound. We discovered that this lead has broad activity against a panel of cancer cell lines and could inhibit tumor growth and tumor angiogenesis.^12^ Administration of this drug either biweekly intravenously (I.V.) or twice daily subcutaneous (S.Q.) did not induce any notable side effects in mice, in contrast to PAK inhibitors that induce cardiotoxicity even at low doses. Here, we report the extensive structure–activity relationship (SAR) exploration and the complete rational drug design study centered on the ARN22089 discovery, with seventeen additional new derivatives. The overall drug discovery effort includes 29 inhibitors (Figure 2). In addition to the resulting SAR, we highlight the identification and characterization *in vitro* and *in vivo* of two optimal back-up/follow-up leads, **27** and **28** (ARN25062 and ARN24928).

## Results and Discussion

### Hit to Lead Process toward the Discovery of Compound ARN22089

To briefly recapitulate our drug discovery program, we applied a virtual screening campaign on a collection of ∼20,000 molecules to identify 68 promising compounds that were experimentally tested for their antiproliferative activity in SKM28 melanoma cells—a cell line that we previously determined was sensitive to RHOJ depletion.^12^ This effort allowed us to identify ARN12405 as a starting hit that inhibited the interaction between RHOJ or CDC42 and its downstream effector PAK (IC_50_ = 16.4 μM, Table 1, entry 1).^12^ The hit features a pyrimidine core bearing a 3-piperidine, a 4-chloroaniline, and a 4-pyridine in 2, 4, and 6 position of the central heterocycle, respectively (Table 1, entry 1).^12^ Looking at the hit structure, we envisioned four points of modification. Therefore, we systematically changed the functional groups of our scaffold A (Figure 2) to improve the potency and the drug-like properties of the hit compound. We mainly explored (i) pyridines with different positions of nitrogen and some simple aromatic rings on C6 of the pyrimidine core, (ii) anilines on position C4 with different substituents embedding electron-donating or electron-withdrawing groups in ortho, meta, and para positions, especially focusing on the dimethylamino and methoxy groups as the main substituents, (iii) several aliphatic six-member (hetero)cycles on C2 between two pyridine nitrogen, and (iv) the core of the scaffold retaining the best substituents on a triazine core.

**Table 1:**
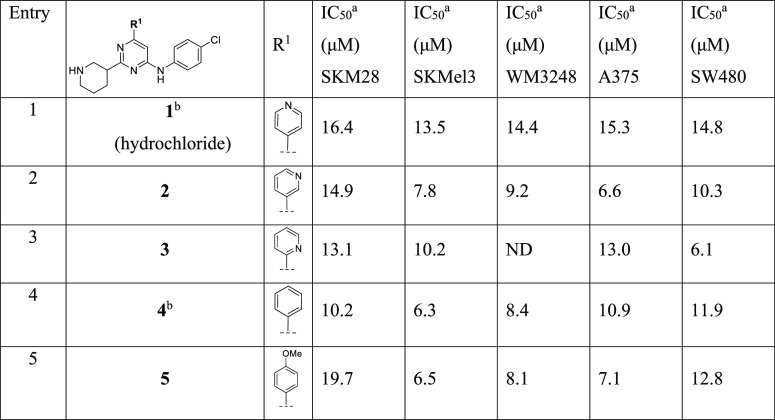
Modification at R^1^

a All IC_50_ values have an *R*^2^ > 0.90.

b Compounds initially reported by Jahid et al.^12^

c ND = not determined.

The antiproliferative activity of all compounds was mainly assessed in SKM28 cells, and the antiproliferative activity of the largest part of analogues was further evaluated in four additional cancer cell lines: three melanoma cell lines (SKMel3, WM3248, and A375) and a colon cancer line SW480 (Tables 1–5). All three melanoma lines have a BRAF (Val600Glu) mutation, while SW480 cells have a KRAS (Gly12Val) mutation. These transformed cells have been shown to have RHOJ/CDC42 activity that synergizes with known Ras/Raf effector pathways (Raf-MEK-ERK and PI3K-Akt).^20,21^

**Table 2:**
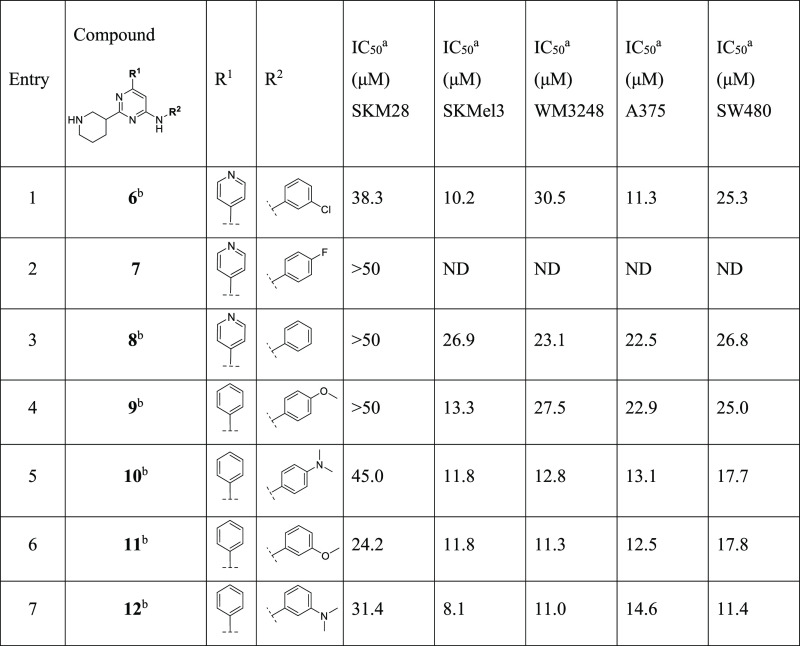
Modification at R^2^

a All IC_50_ values have an *R*^2^ > 0.90.

b Compounds initially reported by Jahid et al.^12^

c ND = not determined.

**Table 3:**
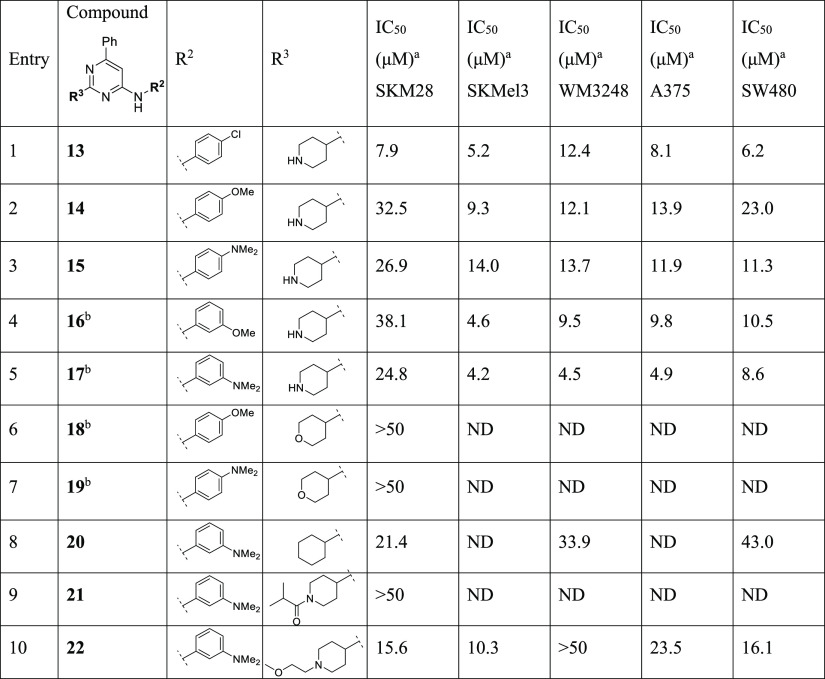
Modification at R^3^

a All IC_50_ values have an *R*^2^ > 0.90.

b Compounds initially reported by Jahid et al.^12^

c ND = not determined.

**Table 4:**
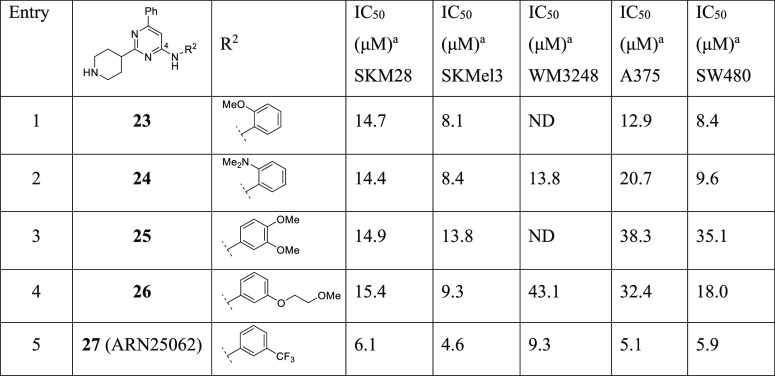
Expansion of R^2^ Exploration

a All IC_50_ values have an *R*^2^ > 0.90.

b ND = not determined.

**Table 5:**
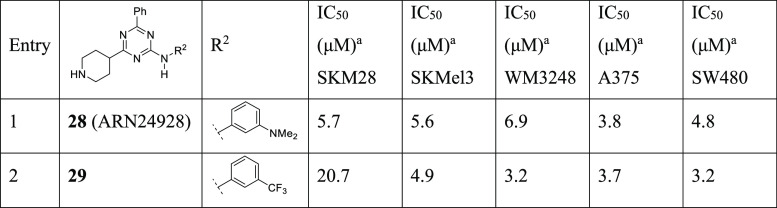
Triazine Analogues and Related Antiproliferative Activity

a All IC_50_ values have an *R*^2^ > 0.90.

#### Optimization of R^1^

All the evaluated modification of R^1^ did not drastically alter the potency in the SKM28 cell line. In detail, moving pyridine nitrogen from 4 (**1**, ARN12405) to 3 or 2 positions as in derivatives **2** and **3** maintained the activity (IC_50_ = 14.9 μM and 13.1 mM in SKM28 cell lines, respectively, entries 1–3, Table 1). However, removal of pyridine nitrogen in analogue **4** moderately increased the activity with an IC_50_ of 10.2 μM in SKM28 (entry 4, Table 1). The introduction of the electron-donating methoxy group as in **5** in para position of the phenyl ring maintained the activity (entry 5, Table 1). Generally, all derivatives **1–5** exhibited a good potency in the other cell lines, showing an IC_50_ in the range of 6.3–15.3 μM.

#### Optimization of R^2^

We then explored the variation of R^2^ in order to alter the electronics of the aniline ring (Table 2). Moving the chlorine from para position to meta position at the aromatic ring in the scaffold with a pyridine and a 3-piperidine gave a 2-fold decrease in activity for **6** (IC_50_ 38.3 μM in SKM28) compared to **1** (entry 1, Table 2). Instead, the activity was maintained in SKMel3 and A375. The substitution of chlorine with a fluorine in para position in derivative **7** and the naked phenyl ring in **8** dropped the activity in SKM28 (IC_50_ > 50 μM, entries 2, 3, Table 2). On the other hand, compound **8** showed a slightly decreased potency in the other four cell lines, with IC_50_ in the range of 20–30 μM. Since these modifications led to a marked decrease in potency in the cell SKM28, we decided to continue the exploration of R^2^, using as the reference compound analogue **4**, which displayed a comparable potency to the hit **1**.

Maintaining the phenyl ring on C6 and a 3-piperidine on C2, we mainly investigated the effects of methoxy and dimethylamino groups in para and meta positions, which generated the derivatives **9–12** (entries 4–7, Table 2). The introduction of these substituents in para position resulted in a total or partial loss of activity in SKM28 (entries 4 and 5, Table 2). The same substituents in meta position moderately diminished the activity of the compounds. Specifically, we observed a 2- and 3-fold reduction for **11** (IC_50_ = 24.2 μM) and **12** (IC_50_ alone 31.4 μM), respectively. In the other four cell lines, derivative **9** led to only a slightly decreased activity, while compounds **10–12** exhibited a good activity with IC_50_ in the 8.1–17.8 μM range.

#### Optimization of R^3^

Examining R^3^ substituents, the initial goal was to move the piperidine nitrogen from position 3 to 4, therefore removing the stereocenter and increasing the chemical tractability (synthesis and chirality). At the beginning, we synthesized the 4-piperidine counterpart of **4**, which generated compound **13** with 4-chloroaniline on C4 and the phenyl substituent on C6 of pyrimidine, which exhibited one-digit micromolar potency in all cell lines (entry 1, Table 3). We then investigated in depth the role of methoxy and dimethylamino substituents on the aniline moiety in combination with 4-piperidine in position 2 (entries 2–5, Table 3), which exhibited a different effect compared to related analogues with a 3-piperidine moiety. This trend would suggest that there is a different way of interaction of 4-piperidine analogues compared to related 3-piperidine equivalents. Generally, the methoxy group in the para and meta position of compounds **14** and **16** has a detrimental effect on the activity in SKM28, showing approximately a 3-fold decrease in potency (entries 2 and 4, Table 3) compared to **4**, while the related dimethylamino counterpart in **15** and **17** remained moderately active. On the other hand, compounds **14–17** showed a good antiproliferative effect toward the other four cell lines.

The oxo counterparts **18** and **19** of compounds **14** and **15** resulted in complete loss of activity (IC_50_ > 50 μM), indicating that a pyrane in C2 is not tolerated (entries 6 and 7, Table 3). Removing the heteroatom through the insertion of the cyclohexyl substituent as in compound **20** resulted in a 2- or 4-fold decrease in activity compared to **4**. Finally, we masked the nitrogen of the piperidine with an acyl and a methoxy alkyl group as in **21** and **22** to understand the role of a free basic center and the potential interaction mediated by its substituents. While derivative **21** did not display a good potency, the insertion of a methoxyethyl group at the piperidine nitrogen in **22** restored the activity mainly in SKM28, SKMel3, and SW480, likely due to supplementary interactions established by the terminal methoxy group.

#### Further Expansion of Aniline as R^2^

Starting from the evidence that the type and conformation of the aliphatic cycle on C2 strongly affect the inhibitory capability of this chemical class, we further expanded the exploration of the aniline moiety maintaining a 4-piperidine on C2 and a phenyl on C6. This was well tolerated as shown for derivate **17** (ARN22089, Table 3). Thus, we completed the evaluation of the main investigated methoxy and dimethyl amino groups synthesizing the ortho-substituted analogues **23** and **24** (Table 4). The shifting of these groups to the ortho position increased the potency in SKM28 compared to the meta and para counterpart, restoring the activity with an IC_50_ of ∼14 μM. On the other hand, the activity in the other cell lines decreased by ∼2–3-fold. A similar effect in the SKM28 cell line was observed with analogues **25** and **26**, which feature a 3,4-dimethoxyphenyl- and a 3-methoxyethyloxyphenyl substituent, respectively. Despite **25** and **26** maintaining the activity in SKM28 and SKMel3, they displayed the weakest effect in other cell lines. Finally, the insertion of an electron-withdrawing H bond acceptor like the trifluoromethyl group in meta position as in compound **27** boosted the potency in all five cancer cell lines, with single-digit micromolar IC_50_ (entry 5, Table 4).

In summary, *meta*-trifluorophenyl and *meta*-*N*,*N*-dimethylaminophenyl R^2^ aniline substituents on C4 stand out as the best functionalities in combination with a phenyl on C6 and a 4-piperidine on C2. This returned a robust antiproliferative activity in all cancer cell lines. In view of these data, we shifted the dimethylaniline and trifluoromethylaniline groups into a triazine core, generating compounds **28** and **29** and symmetrizing the system. In general, this strategy confirmed the positive contribution of the selected substituents that exhibited a similar or slightly improved potency in all cell lines, with the exception of compound **29** toward SKM28 (IC_50_ = 20.7 μM).

### Evaluation of Drug-like Properties

After this initial evaluation, we assessed the drug-like profile of these novel RHOJ/CDC42 inhibitors by studying the kinetic solubility and the plasma and phase I microsomal stability of 19 selected compounds (Table 6). Previously, we selected **17** (ARN22089) as the most promising compound, since it displayed the best compromise between potency, solubility, metabolic stability, and chemical tractability.^12^**17** was selected for further *in vivo* studies.^12^ Notably, the starting hit **1** exhibited a good kinetic solubility of 222 μM, an excellent plasma stability over 120 min, and an acceptable microsomal stability of 48 min (Table 6, entry 1). All compounds exhibited an excellent kinetic solubility (>150 μM), with the exception of analogues **2–5**, **13**, and **29** (Table 6). From these data emerged that only the 4-pyridine on C6 in combination with a chloroaniline on C4 gave a good kinetic solubility, while a 3-pyridine, a 2-pyridine, a naked phenyl, or a *p*-methoxyphenyl drastically dropped the solubility. Indeed, phenyl and *p*-methoxy derivatives (**4** and **5**) exhibited the lowest kinetic solubility (<1 μM), followed by compounds **2** and **3** (38 ± 4 and 45 ± 3 μM, respectively); so, they were excluded for further investigations. Surprisingly, the introduction of the trifluoromethyl group on the aniline moiety moderately diminished the solubility in the pyrimidine derivative **27** compared to previously selected compound **17**, while when −CF_3_ was inserted in the triazine core as in compound **29**, the solubility dropped to 1 μM. All compounds showed a good plasma stability (>70 min), with most of them showing acceptable microsomal stability (*t*_1/2_ > 40 min). Furthermore, the solubility of compounds with Sk > 30 μM was confirmed by NMR measurement. This preliminary analysis is crucial in order to avoid false-positive results in the binding assays, which we performed via microscale thermophoresis (MST) and NMR. The compounds were tested by NMR in the binding assay buffer at different concentrations (from 10 to 100 μM) in the presence of an internal reference (4-trifluoromethyl benzoic acid, 400 μM) to assess their solubility and their aggregation state under these experimental conditions (SPAM filter,^22^Table 6). The solubility data obtained by NMR were consistent with the kinetic solubility values (data not shown). Notably, derivatives **27** (ARN25062) and **28** (ARN24928) resulted to be the best compounds in terms of potency in all cell lines, kinetic solubility, also showing a strong improvement in plasma and microsomal stability.

**Table 6:** Kinetic Solubility and Plasma and Microsomal Stability in Mice of Selected Compounds^a^

entry	compound	S kinetic (μM)	aggregation by NMR (μM)	*T*_1/2_ plasma (min)	*T*_1/2_ microsomal (min)
1	**1**	222	ND	>120	48
2	**2**	38	no aggreg. up to 50	104	17
3	**3**	45	no aggreg. up to 50	>120	52
4	**4**^b^	<1	ND	>120	46
5	**5**	<1	ND	>120	ND
6	**10**^b^	246	100	>120	43
7	**11**^b^	237	no aggreg. up to 100	>120	21
8	**12**^b^	>250	no aggreg. up to 50	119	20
9	**13**	3	ND	>120	52
10	**14**	>250	no aggreg. up to 100	95	>60
11	**15**	180	30	>120	49
12	**16**^b^	>250	100	>120	27
13	**17** (ARN22089)^b^	250	100	71	27
14	**22**	107	100	>120	12
14	**23**	219	50	>120	46
15	**24**	210	50	>60	>60
16	**25**	>250	no aggreg. up to 100	97	54
17	**27** (ARN25062)	168	100	>120	45
18	**28** (ARN24928)	209	100	>120	>60
19	**29**	1	ND	>120	>60

a ND = not determined.

b Compound initially reported by Jahid et al.^12^

### Microscale Thermophoresis and NMR for CDC42-Binding Validation

In order to evaluate target engagement of the most interesting compounds, we exploited two different techniques: microscale thermophoresis (MST) and NMR analysis. Among the selected 19 compounds, **2**, **4**, **5**, **13**, **15**, and **29** were excluded from the analysis due to their low kinetic solubility or aggregation at a concentration of < 50 μM (Table 6).

MST is a biophysical technique that saw a rapid increase in popularity in the past few years, especially as a method for fragment screening. This was mainly due to the low amount of sample required, short run time, absence of sample-immobilization requirements, and the ability to detect binders in a wide range of affinities (both strong and weak binders). Target proteins are Red-NHS labeled to monitor their change in thermophoresis in a temperature gradient that is produced by an IR-laser on a small area of the sample solution. The interaction with a small molecule leads to a shift in the protein thermophoresis, which is measured by an UV detector and given as the output.

**Figure 3:**
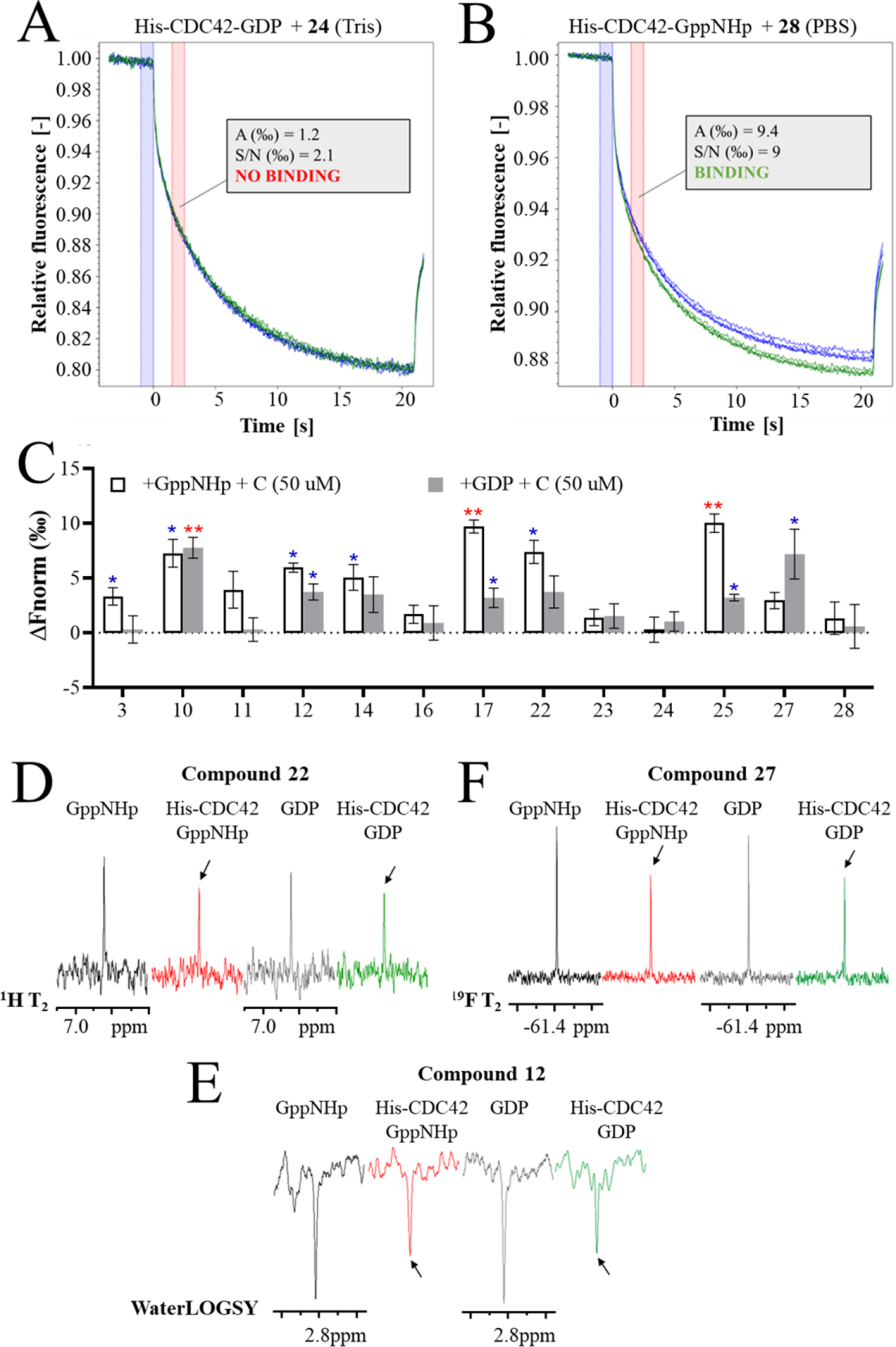
(A,B) MST analysis of selected compounds. An example of binding check by MST [compounds **24**-(A) and **28**-(B)]. The MST traces were recorded as follows: 3 s MST power off, 20 s MST power on, and 1 s MST power off. The amplitude of the response (A) and the signal-to-noise ratio (S/N) at 1.5−2.5 s were taken into account to evaluate binding. Compound **24** thermophoresis experiment was set up in Tris buffer pH 7.5 for the evaluation of the binding to inactive His-CDC42 (loaded with GDP). Compound **28** thermophoresis experiment was set up in PBS buffer for the evaluation of the binding to active His-CDC42 (loaded with GppNHp). (C) graph displays the response amplitude of the experiment setup in Tris buffer. The amplitude of the response is the difference in normalized fluorescence (Δ*F*_norm_ [‰] = *F*_hot_/*F*_cold_) between the protein/compound sample and a protein-only sample. Compounds (C) were tested at 50 μM toward the activated (loaded with GppNHp) or inactivated (loaded with GDP) His-CDC42. A single blue asterisk indicates a signal-to-noise ratio above 5, while double red asterisks indicate a signal-to-noise ratio above 12. (D−F) examples of binding check by NMR of compound **22** [^1^H NMR T2 filter-(D)], compound **27** [^19^F T2 filter-(E)], and compound **12** [WaterLOGSY-(F)]. Compounds were tested: in the absence of protein, while in the presence of GppNHp (black) or GDP (gray), and in the presence of activated (loaded with GppNHp) His-CDC42 (red) or inactivated (loaded with GDP) His-CDC42 (green). The arrows indicate where a difference in the compound NMR signal is observed after the addition of the protein, highlighting a binding event.

In this MST analysis, wild-type His-CDC42 (Ile4-Pro182) was used as target macromolecule. The fusion protein was expressed, purified, and loaded with GDP (90% efficiency of loading) or GppNHp (>98% efficiency of loading), as previously described.^12^ The difference in normalized fluorescence (Δ*F*_norm_ [‰] = *F*_hot_/*F*_cold_) between a protein–compound sample and a protein-only sample was registered. Binding events were tested at a compound concentration of 50 μM throughout all the experiments (Figure 3 and Table S1, Supporting Information). Assays were set up in Tris–HCl buffer (for the binding to His-CDC42 loaded with GppNHp or GDP) and phosphate-buffered saline (PBS) buffer (for the binding to His-CDC42 loaded with GppNHp only).

Three compounds were highlighted for their confidence in binding and signal-to-noise ratio higher than 12 (Table S1, Supporting Information): **17**,^12^ the previously identified lead compound, **10**, and **25**. All three compounds carry a phenyl ring on C6 of the pyrimidine and a 4-piperidine cycle on C2. The aniline moiety is instead substituted at position 3 and/or 4 with either a dimethylamine group or a methoxy group. Additional binders were identified with a lower signal-to-noise ratio (between 5 and 12). They are **3**, **12**, **14**, **22**, **27**, and **28** (Figure 3 and Table S1, Supporting Information).

To further strengthen the binding evidence obtained through MST, we evaluated the binding of our derivatives by NMR.^23^ Here, it was employed as a secondary assay to confirm the binders identified by MST: derivatives **3**, **10**, **12**, **14**, **17**, **22**, **25**, **27**, and **28**. First, the stability and the aggregation state of both (loaded with GppNHp) His-CDC42 and (loaded with GDP) His-CDC42 proteins were assessed by the ^19^F transverse relaxation (T_2_) filter^24^ testing a mixture of 35 fluorinated fragments (soluble and not aggregating) in the absence and in the presence of the proteins just after purification (*t*_0_) and 24 h later (*t*_1_). No differences in the NMR spectra of the mixture in the absence and in the presence of proteins are visible at both *t*_0_ and *t*_1_, suggesting that there are no diffused bindings, hence no aggregation, and that the protein is stable at least for 24 h under our experimental conditions (Table 6 and Figure S1, Supporting Information). The MST binders were then tested in the absence and in the presence of both His-CDC42 by ^1^H or ^19^F (fluorinated compounds) T_2_ filters^24^ and WaterLOGSY (water-ligand observation with gradient spectroscopy) experiments.^25^ In T_2_ filter experiments, the NMR signals of a compound that binds the protein show line broadening in the presence of the protein, resulting in a reduction in their intensities, as reported in the examples of Figures 3D and 3E as ^1^H and ^19^F T_2_ filter spectra, respectively. The WaterLOGSY experiment is based on magnetization transfer from bulk water to the compounds that interact with a macromolecule. In the presence of binding, the WaterLOGSY signal of the compound changes from negative to less negative or to positive, as shown in the example of Figure 3F. As reported in Table S1 in the Supporting Information, MST hits were confirmed by NMR, but according to NMR data, compounds **3**, **14**, **22**, and **28** bind also to the inactivated form of His-CDC42. This result is not surprising as it is well known that NMR is highly sensitive even to very weak binders.^26^

### Molecular Modeling for Binding Validation

The previously identified drug binding pocket localized at the CDC42-PAK protein–protein interface was used for our computational studies.^12^ Accordingly, we performed molecular docking calculations of the follow-up lead molecules **27** (ARN25062) and **28** (ARN24928) on the GTP-bound active form of CDC42.

**Figure 4:**
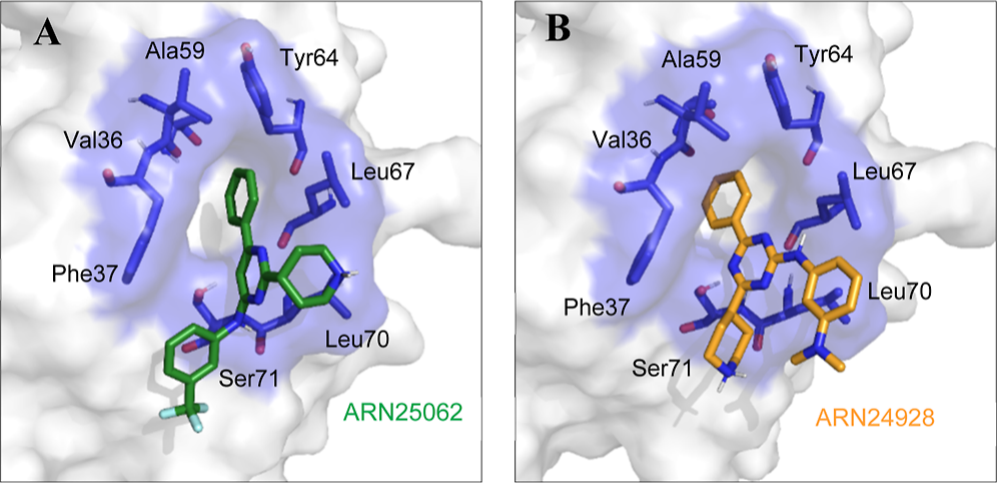
Model structure of compounds **27** [ARN25062, panel (A)] and **28** [ARN24928, panel (B)] bound to the allosteric drug-binding pocket of CDC42 is reported. The structure of CDC42 is represented as the white surface, while the identified drug-binding pocket is shown in both sticks and transparent blue surface. ARN25062 and ARN24928 are reported as green sticks and yellow, respectively.

Modeling predicts that both **27** (ARN25062) and **28** (ARN24928) fit within the effector pocket of CDC42 (Figure 4A,B), which is consistent with the binding mode of **17** (ARN22089) and the hit **1** binding mode in CDC42, as previously reported (Figure 4).^12^

**Figure 5:**
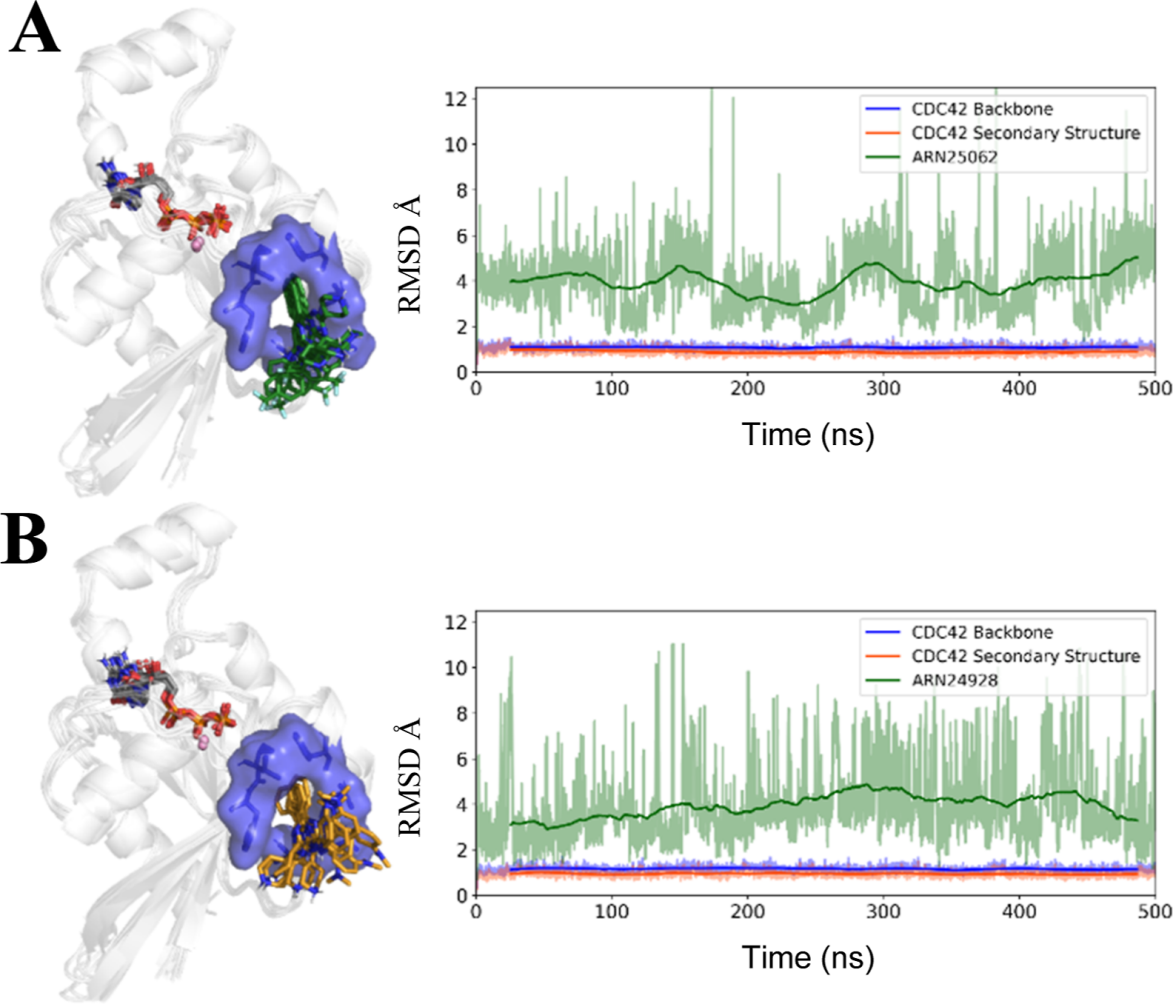
MD simulations of the protein–ligand complexes. The structural representation of CDC42 in complex with compound **27** [ARN25062, panel (A)] or compound **28** [ARN24928, panel (B)] is reported on the left. CDC42 is represented as the schematic, while the binding pocket is highlighted as both the sticks and transparent blue surface. Multiple MD snapshots of the **27** (ARN25062, green) and **28** (ARN24928, yellow) binding poses are shown as sticks. On the right, the rmsd over time for the CDC42-ligand complexes is reported with the rmsd running averages highlighted in bold.

The hydrophobic nature of the pocket easily accommodates the phenyl on C6, which represents the anchor point for binding of both the lead **17** (ARN22089) and for our back-up compounds, as shown in Figure S2. Noteworthily, a different orientation of the piperidine portion in the binding mode with respect to the other active compounds has been predicted for compound **27** (ARN25062), where the accommodation of the phenyl ring is maintained, while the positioning of the pyridine core is inverted (Figure 4). This is probably due to the close positively charged Lys5, which attracts the electronegative group −CF_3_ (Figure S3). However, further structural analyses are needed in order to determine the accurate compound binding mode. In this regard, to further assess the predicted binding poses, we performed equilibrium molecular dynamics (MD) simulations of both **27** (ARN25062) and **28** (ARN24928) in complex with CDC42 (Figure 5).

As for the previously reported hit and lead compounds,^12^ the binding pose of both **27** (ARN25062) and **28** (ARN24928) in the CDC42-ligand complexes is preserved during 500 ns-long MD simulations (rmsd = 3.90 ± 1.30 Å and 3.80 ± 1.64 for **27** (ARN25062) and **28** (ARN24928) CDC42 complexes, respectively (Figure 5A,B, right panels). Indeed, as shown in Figure 5 (left panels), **27** (ARN25062) and **28** (ARN24928) steadily bind the target pocket throughout the MD simulations.

### *In Vivo* Pharmacokinetics of the Selected Follow-Up/Back-Up Leads **27** and **28**

**Figure 6:**
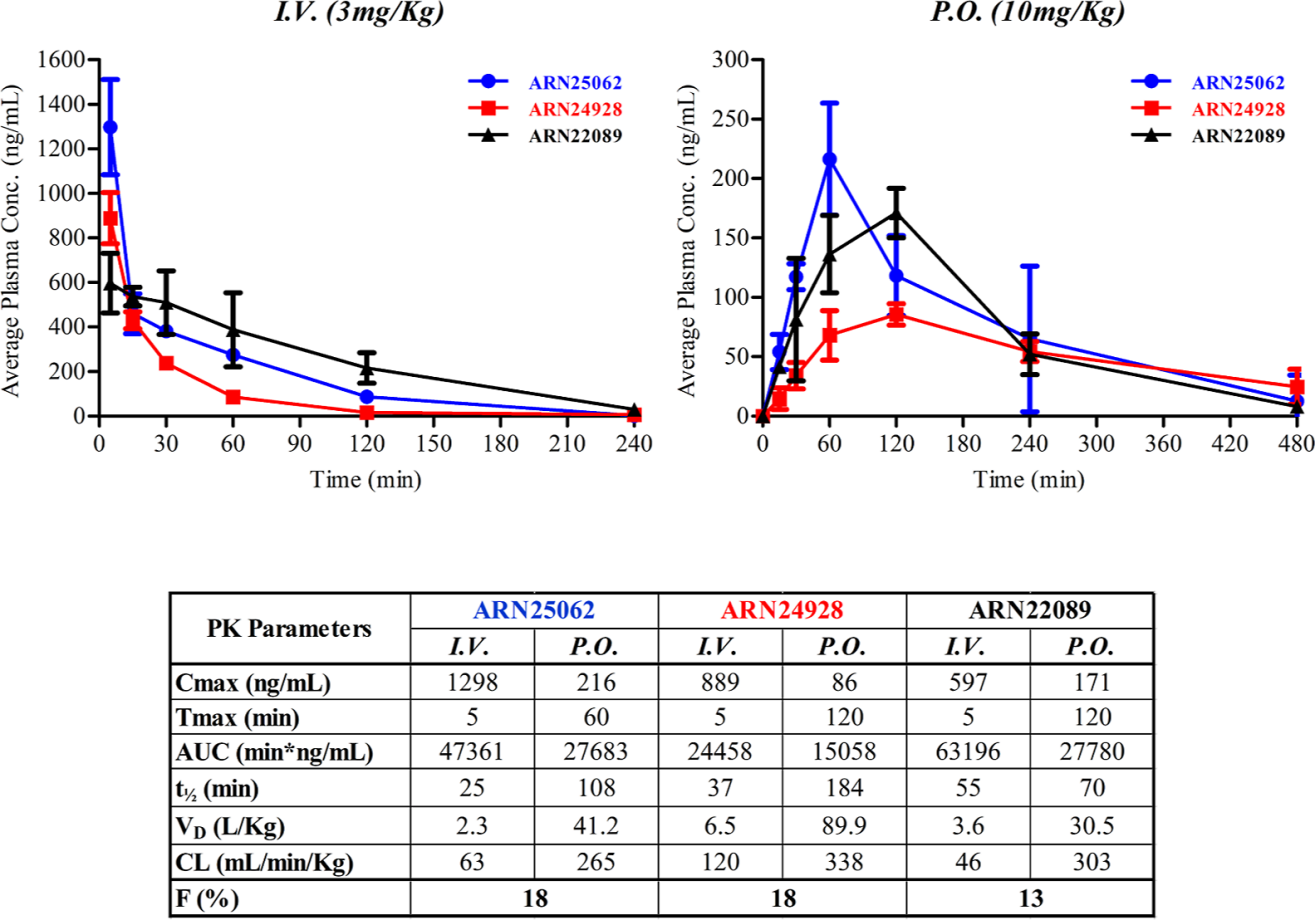
Mouse PK profiles of ARN25062, ARN24928, and ARN22089^12^ following intravenous (I.V.) and oral (P.O) administration at 3 and 10 mg/kg, respectively, and the corresponding observed and calculated PK parameters.

On the basis of the overall results and drug-like profile of our compounds, **27** (ARN25062) and **28** (ARN24928) were selected as candidates for *in vivo* pharmacokinetics (PK) studies, in view of further experiments to assess their *in vivo* efficacy in animal models (mouse species) of cancer and also compare the PK profile to the lead’s one (**17**, ARN22089, Figure 6).

We tested two different routes of administration: (i) I.V. injection at a concentration of 3 mg/kg (*n* = 3 animals for each time point), and ii) oral (P.O.) treatment at a dose of 10 mg/kg (*n* = 3 animals for each time point, Figure 6). The two back-ups showed PK profiles comparable to ARN22089, as previously evaluated.^12^ During the PK studies, via either I.V. or P.O. administration, ARN25062 and ARN24928 were well tolerated by all animals, and no treatment-related toxicological signs were observed. Indeed, I.V. profiles of ARN25062 and ARN24928 reached a *C*_max_ of 1298 and 889 ng/mL in 5 min after administration, respectively, followed by a protracted elimination phase. Both compounds were still detectable after 2 h at a concentration of 87 and 17 ng/mL, respectively. After oral administration (10 mg/kg), ARN25062 achieved the maximum concentration (*C*_max_ = 216 ng/mL) in 1 h, significantly faster than compound ARN24928 (*C*_max_ = 86 ng/mL and *T*_max_ = 2 h), and both compounds were still detectable after 8 h at concentrations of 13 and 25 ng/mL, respectively. In particular, compound ARN24928 showed good exposure with a long half-life of 37 min (I.V.) and 184 min (P.O.). In summary, these data indicate that both compounds are well tolerated after a single injection and possess a favorable PK profile, comparable to that of the lead ARN22089.^12^

### *In Vivo* Efficacy of the Selected Follow-Up/Back-Up Leads **27** and **28**

**Figure 7:**
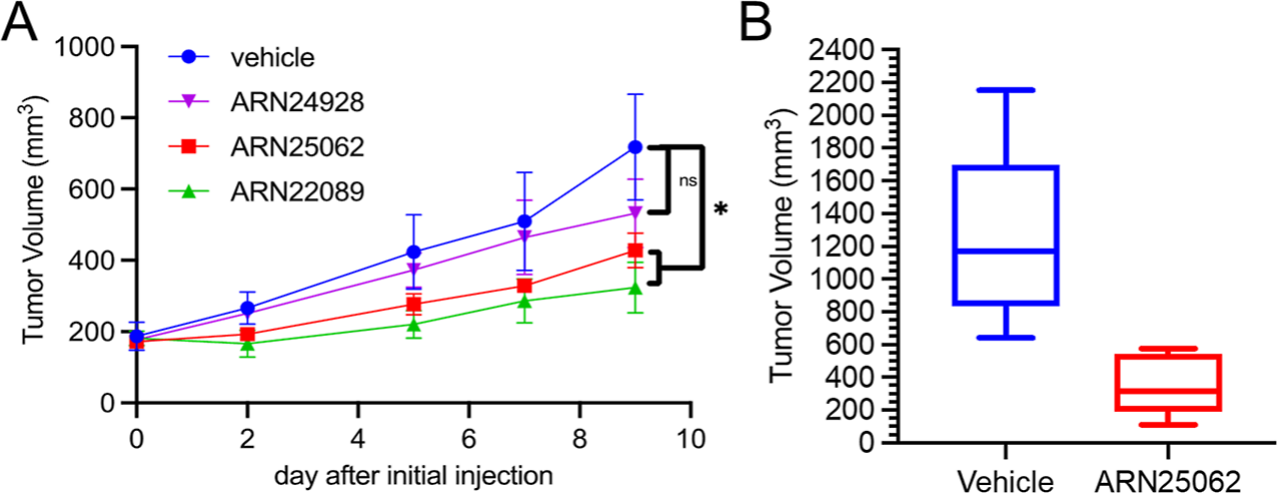
ARN25062 but not ARN24928 significantly inhibits tumor growth in the PDX model. (A) When initial tumors reached 150–250 mm^3^, mice were injected I.V. with 10 mg/kg ARN25062, ARN24928, or ARN22089 (4 tumors per group) or with vehicle (3 tumors per group), every day for a week. Values are given as individual tumor volume, error bars as SEM, two-tail *T*-test, *p-value ≤ 0.05, ^ns^*p*-value ≤ 0.125. (B) Mice were treated with 10 mg/kg ARN25062 or with vehicle daily for 2 weeks. Tumors were extracted, and volumes were calculated. Values are tumor volumes (mm^3^) from ARN25062 (*n* = 6) or vehicle (*n* = 6), error bars as SD, two-tail *T*-test, *p*-value ≤ 0.0025.

We previously showed that ARN22089 significantly inhibited the growth of patient-derived xenografts (PDXs) in NOD scid gamma (NSG) mice at 25 mg/kg I.V. twice a week.^12^ Similarly, to test the efficacy of **27** (ARN25062) and **28** (ARN24928) *in vivo*, we inoculated PDX tumor chuck underneath the skin in NSG mice on both flanks and treated the mice with 10 mg/kg ARN25062 or ARN24928 or vehicle via I.V. daily for a week. We included the lead compound, ARN22089, to directly compare the efficacy of the two new derivatives with the initial lead’s one. ARN25062 significantly inhibited tumor growth, with similar efficacy as the lead compound (Figure 7A), compared to ARN24928, which had less efficacy (Figure 7A). To determine the efficacy of longer term administration on tumor growth, we extended the treatment for ARN25062 for a total of 2 weeks of daily I.V. administration at a dose of 10 mg/kg. After 2 weeks, the tumors were harvested, and the volume (mm^3^) was calculated for each tumor. 2 week daily treatment reduced tumor volume significantly for ARN25062 compared to vehicle (Figure 7B). Both ARN25062 and ARN22089 had a similar effect after 2 weeks, and there was no body weight change or overt toxicity observed in the drug-treated animals (data not shown).

### Chemistry

**Scheme 1:**
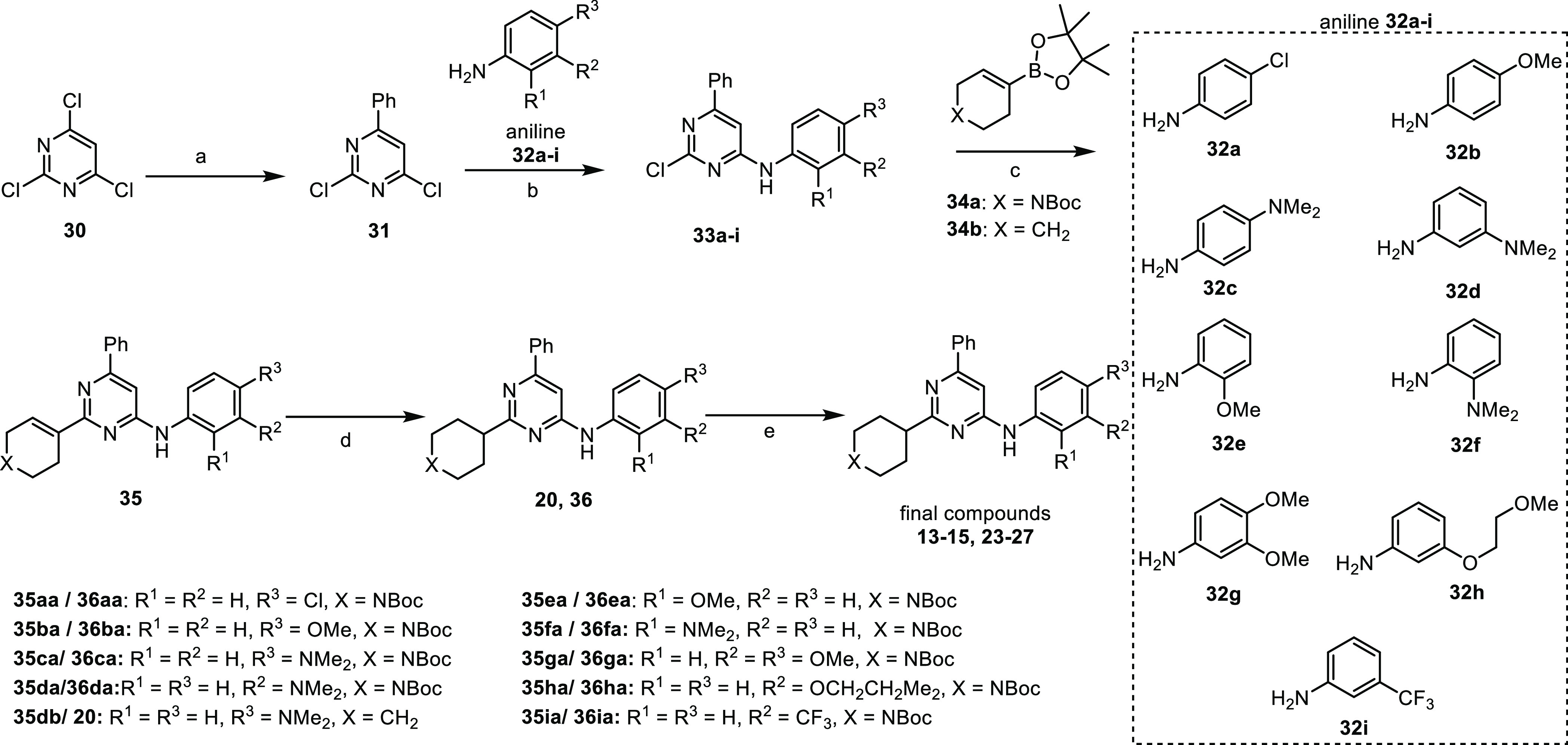
Synthesis of Additional Trisubstituted Pyrimidine Derivatives **13–15**, **20**, and **23–27** Reagents and conditions: (a) PdCl_2_(dppf)CH_2_Cl_2_, K_2_CO_3_ (2 M)_aq_, 1,4-dioxane dry, 60 °C MW; (b) (i) LiHMDS, aniline **32a–i**, THF dry, −60 to 0 °C or (ii) Pd(OAc)_2_, rac-(BINAP), Cs_2_CO_3_, 1,4-dioxane dry, 60 °C, MW; (c) **34a–b**, PdCl_2_(dppf)CH_2_Cl_2_, K_2_CO_3_ (2 M)_aq_, 1,4-dioxane dry, 120 °C MW; (d) (i) NH_4_COOH, Pd(OH)_2_/C, MeOH, 80–90 °C or (ii) Et_3_SiH, Pd/C, from −10 to 0 °C; and (e) HCl (4 M in dioxane), 1,4-dioxane.

We obtained the final targets **13–15**, **20**, and **23–27** using the five-step synthetic route outlined in Scheme 1. The first Suzuki cross-coupling reaction between commercial 2,4,6-trichloropyrimidine **30** and phenylboronic acid in the presence of PdCl_2_(dppf)CH_2_Cl_2_ and K_2_CO_3_ afforded intermediate **31** with the phenyl group on C6 with 72% yield. The regioselectivity of the reaction was driven using a mild temperature of 60 °C in 1,4-dioxane under microwave irradiation for 1 h. The yield of the transformation was partially affected by the formation with the time of the undesired 2,4-diphenylpyrimidine byproduct. The regioselective amination on C4 of dichloropyridine core **31** with suitable functionalized anilines **32a–i** was achieved through two different types of transformation: the C4-regioselective nucleophilic aromatic substitution employing LiHDMS as a strong base in tetrahydrofuran (THF) at −60 °C or Buchwald–Hartwig reaction using Pd(OAc)_2_, rac-(BINAP), and Cs_2_CO_3_ in 1,4-dioxane at 60 °C under microwave irradiation.^27^ The first procedure accessed di-substituted pyrimidine **33a** and **33e–i** with an excellent 80–92% range yield, while the second type of reaction generated compounds **33b−d** with a 63–67% range yield, as previously reported.^12^ The second Suzuki cross-coupling between suitable boronic esters **34a** and **34b** and the intermediate of type **33** in the presence of PdCl_2_(dppf)CH_2_Cl_2_ and K_2_CO_3_ in 1,4-dioxane at 120 °C in microwave yielded trisubstituted pyrimidine of type **35** with a very good 60–89% range yield. Finally, the reduction of the double C–C bond of cyclohexenyl or dihydropyridine moieties on C2 and final Boc deprotection get easy access to final desired compounds. Except for compound **35aa**, the double-bond reduction was reduced in the presence of ammonium formate and Pd(OH)_2_/C in MeOH at 80 °C. Since a dechlorination side reaction occurred under this reductive condition, a milder procedure with Et_3_SiH, Pd/C at −10 °C in a 3:3:1 mixture toluene/ethyl acetate/EtOH was used to obtain protected precursor **36aa** from **35aa** with 23% yield after chromatography purification. The low yield was due to the incomplete conversion of the starting material and the low formation of the undesired dechlorinated byproduct (5%). The final Boc deprotection with HCl (4 M in dioxane) afforded final derivatives **13–15** and **23–27** with 16–96% range yield.

**Scheme 2:**
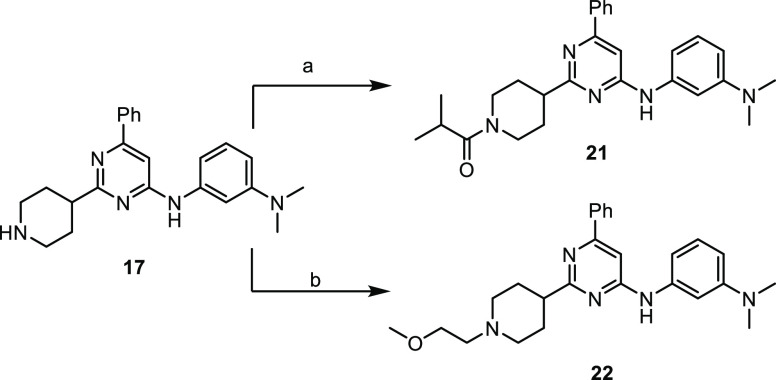
Synthesis of Final Compounds **21** and **22** Reagents and conditions: (a) IprCOOH, HATU, DIPEA, DMF and (b) BrCH_2_CH_2_OMe, DIPEA, CH_3_CN, 60 °C.

As shown in Scheme 2, derivatives **21** and **22** were obtained through direct functionalization of free piperidine of compound **17** via amide coupling with isobutyric acid and nucleophilic substitution on 2-bromoethyl methyl ether, respectively.

**Scheme 3:**
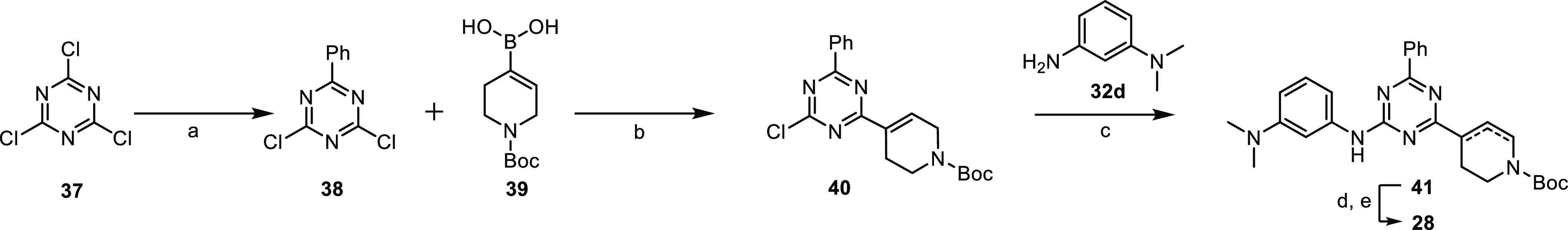
Synthesis of Triazine Analogue **28** Reagents and conditions: (a) Pd(PPh_3_)_2_Cl_2_, K_2_CO_3_, toluene dry, 60 °C; (b) Pd(PPh_3_)_2_Cl_2_, K_2_CO_3_, toluene dry, 80 °C; (c) aniline **32d**, DIPEA, THF dry, 80 °C; (d) i) NH_4_COOH, Pd(OH)_2_/C, MeOH, 80–90 °C; and (e) HCl (4 M in dioxane), 1,4-dioxane.

The preparation of the two triazine analogues required a different specific strategy due to the peculiar reactivity of cyanuric chloride (2,4,6-trichlorotriazine) **37** (Scheme 3). It is well known that the C–Cl bond reactivity of the triazine substrate subsequently decreases from cyanuric chloride **37** to monochlorotriazine toward both metal cross-coupling and aromatic nucleophilic substitution transformations.^28,29^ Accordingly, the orthogonal chemoselective substitution of thrichlorotriazine with the three specific functionalities of final compounds **28** and **29** was carefully evaluated, changing the reaction conditions and the order of synthetic sequence.

As depicted in Scheme 3, the synthesis toward triazine derivative **28** began from inexpensive cyanuric chloride **37**. The first Suzuki reaction with the simple boronic acid occurred in the presence of Pd(PPh_3_)_2_Cl_2_ and K_2_CO_3_ in toluene dry at 60 °C, yielding monophenyl-substituted **38** with 51% yield. At first, the second Suzuki between intermediate **38** and boronic ester **34a** was attempted, but the reaction did not give any conversion into desired product **40** regardless of the type of catalyst (Pd(dppf)Cl_2_·DCM, Pd(PPh)_3_Cl_2_, and Pd(PPh_3_)_4_), the base (K_2_CO_3_ and K_3_PO_4_), or the temperature (80, 100, and 120 °C) employed. Thus, we changed the intrinsic reactivity of the boronic partner, transforming the boronic ester **34a** into corresponding boronic acid **39** with NaIO_4_ and NH_4_OAc in the acetone/water mixture (see the Experimental Section). Notably, boronic acid **39** selectively and efficiently reacted with phenyl triazine **38** to give di-substituted product **40** by using PdCl_2_(PPh_3_)_2_ and K_2_CO_3_ in toluene at 80 °C with 71% yield. Finally, the aniline moiety was introduced by exploiting a nucleophilic aromatic substitution between aniline **32d** and monochloro triazine **40** with DIPEA in THF at 80 °C, affording trisubstituted triazine **41** as a mixture of isomers with 94% combined yield. Indeed, under these conditions, the double C–C bond underwent partial isomerization from C1′–C2′ positions to C2′–C3′ position, but this side reaction was irrelevant since both isomeric forms of **41** were reduced to related saturated derivatives with NH_4_COOH and Pd(OH)_2_/C. The final Boc deprotection gave desired analogue **28** with 51% yield after two steps.

**Scheme 4:**
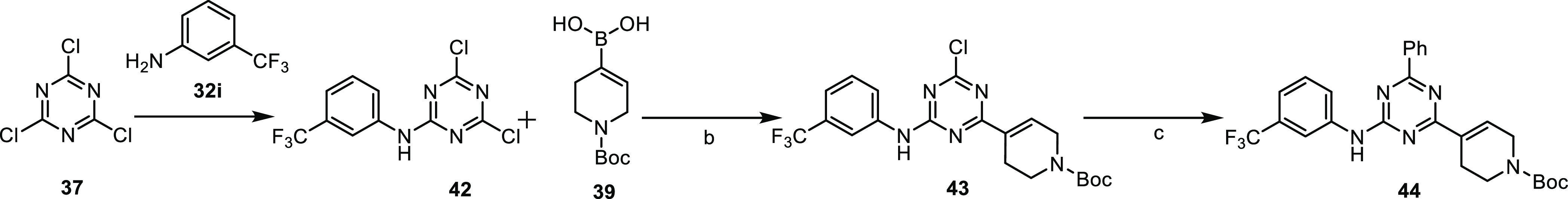
Synthesis of Intermediate **44** toward Final Compound **29** Reagents and conditions: (a) aniline **32i**, DIPEA, dichloromethane (DCM), 0 °C; (b) Pd(PPh_3_)_2_Cl_2_, K_2_CO_3_ (2 M)_aq_, toluene dry, 90 °C; and (c) phenyl boronic acid, Pd(PPh_3_)_2_Cl_2_, K_2_CO_3_ (2 M)_aq_, toluene dry, 120 °C.

**Scheme 5:**
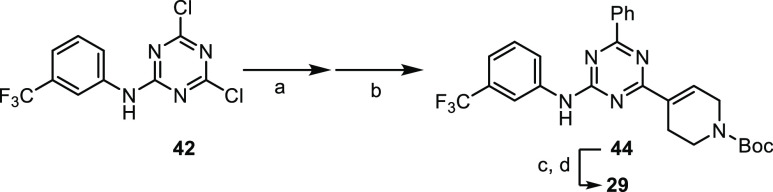
One-Pot Synthesis Optimization toward Compound **29** Reagents and conditions: (a) Pd(PPh_3_)_2_Cl_2_, K_2_CO_3_ (2 M)_aq_, toluene dry, 90 °C; then (b) phenyl boronic acid, Pd(PPh_3_)_2_Cl_2_, K_2_CO_3_ (2 M)_aq_, toluene dry, 120 °C; (c) (i) NH_4_COOH, Pd(OH)_2_/C, MeOH, 80–90 °C; and (d) HCl (4 M in dioxane), 1,4-dioxane.

Synthesis of analogue **29** required a different synthetic sequence due to the weaker nucleophilic nature of aniline **32i** compared to **32d**. Every attempt of aromatic nucleophilic substitution with **32i** on the advanced intermediate **40** failed in the presence of different bases (NaH, LiHMDS, and DIPEA) and solvents (acetone, THF, and DCM). The alternative Buchwald reaction with NaOtBu and Pd_2_(dba)_3_ gave impure conversion, too. To overcome these issues, we introduce aniline **32i** at the beginning of the synthetic route on trichlorotriazine **37**, which is more prone to undergo nucleophilic substitution than mono- and di-substituted triazine (Scheme 4). In confirmation of this, reaction between aniline **32i** and trichlorotrizine **37** under mild conditions (DIPEA and DCM at 0 °C) afforded desired substrate **42** with 24% yield. Then, two sequential Suzuki couplings with boronic acid **39** and phenyl boronic acid were successfully performed through a proper stoichiometry of the reagent and temperature in the presence of PdCl_2_(PPh_3_)_2_ and K_2_CO_3_, giving advanced intermediate **44** with 21% yield after two steps (Scheme 3). Given the high selectivity in stepwise functionalization of dichloro triazine, we developed a one-pot procedure for obtaining intermediate **44** starting by pure compound **42**, improving the two-step yield from 21 to 54% (Scheme 5). Finally, after the classic double C–C reduction and Boc removal, we obtained final compound **29**.

In summary, through the optimization of the key Suzuki and aromatic nucleophilic substitution reaction, we built up a simple and efficient five-step synthetic route to get access to a widely diversified class of new pyrimidine and triazine RHOJ/CDC42 inhibitors.

## Conclusions

Here, we have presented additional efforts of our drug discovery program targeting the CDC42 GTPase (RHOJ, CDC42, and RHOQ) proteins, which are overexpressed in multiple tumor types. Based on the recent discovery of a lead compound **17**, ARN22089,^12^ which has shown anticancer activity *in vivo*, we have expanded this new chemical class of RHOJ/CDC42 inhibitors. Importantly, we have identified and characterized two follow-up/back-up compounds, namely, **27** (ARN25062) and **28** (ARN24928), out of an SAR generated with ∼30 close analogues bearing different substituents at the pyrimidine and triazine core of this new chemical scaffold (Figure 2).

The resulting SAR elucidates the key structural features that enhance the potency and ameliorate the drug-like profile of our new trisubstituted pyrimidine/triazine derivatives. Indeed, we explored three points of modification of the pyrimidine class. The first subset includes compounds **1–5** with several substituents on C6; then, we explored the aniline moiety on C4, synthesizing **6–13** and **23–27**. We evaluated the effect of different aliphatic (hetero)cycles on C2 with compounds **14–22**. This study identified the best functionalities on a pyrimidine core that were also retained on the symmetric triazine core, like in **28–29**. The nineteen most potent compounds were further investigated for their kinetic solubility and *in vitro* plasma and metabolic stability.

The measured antiproliferative activity and *in vitro* binding data at the target, together with our extensive analyses of the drug-likeness profile, indicate the novel derivatives **27** (ARN25062) and **28** (ARN24928) as the most drug-like CDC42/RHOJ inhibitors of this novel chemical series, which are comparable to the previously identified drug candidate **17** (ARN22089). These back-up/follow-up compounds have also displayed a stable binding at the target pocket through MD simulations and a favorable PK profile comparable to **17** (ARN22089).

Notably, we also measured the *in vivo* efficacy of the two lead compounds **27** and **28**, with analogue **27** (ARN25062) that has exhibited a significant ability to inhibit tumor in PDXs *in vivo*, with an efficacy comparable to the lead **17**.^12^ These overall results and extended drug discovery effort further demonstrate the potential of our new chemical class of CDC42/RHOJ inhibitors, with lead compounds ready for advanced preclinical studies to develop new cancer treatments.

## Experimental Section

### Chemistry

#### Chemistry General Considerations

All the commercially available reagents and solvents were used as purchased from vendors without further purification. Dry solvents were purchased from Sigma-Aldrich. Automated column chromatography purifications were done using a Teledyne ISCO apparatus (CombiFlash Rf) with pre-packed silica gel columns of different sizes (from 4 g up to 24 g) and mixtures of increasing polarity of cyclohexane and ethyl acetate (EtOAc) or DCM and methanol (MeOH). NMR data were collected on 400 or 600 MHz (^1^H) and 100 or 150 MHz (^13^C). Spectra were acquired at 300 K, using deuterated dimethyl sulfoxide (DMSO-*d*_6_) or deuterated chloroform (CDCl_3_) as solvents. For ^1^H NMR, data are reported as follows: chemical shift, multiplicity (s = singlet, d = doublet, dd = double of doublets, t = triplet, q = quartet, and m = multiplet), coupling constants (Hz), and integration. Ultra-performance liquid chromatography-mass spectrometry (UPLC-MS) analyses were run on a Waters ACQUITY UPLC-MS instrument consisting of a single quadrupole detector (SQD) mass spectrometer. The analyses were performed on an ACQUITY UPLC BEH C_18_ column (50 × 2.1 mm ID and particle size 1.7 μm) with a VanGuard BEH C_18_ pre-column (5 × 2.1 mm ID and particle size 1.7 μm) (Log *D* > 1). The mobile phase was 10 mM NH_4_OAc in H_2_O at pH 5 adjusted with AcOH (A) and 10 mM NH_4_OAc in CH_3_CN–H_2_O (95:5) at pH 5 (B). Electrospray ionization in positive and negative modes was applied in the mass scan range 100–500 Da. Depending on the analysis method used, a different gradient increasing the proportion of mobile phase B was applied. For analysis method 1, the mobile phase B proportion increased from 5 to 95% in 2.5 min. For analysis method 2, the mobile phase B proportion increased from 50 to 100% in 2.5 min. For analysis method 3, the mobile phase B proportion increased from 70 to 100% in 2.5 min. High-resolution mass spectrometry (HRMS) was carried out on a Waters Synapt G2 Quadrupole-TOF instrument equipped with an ESI ion source. The analyses were run on an ACQUITY UPLC BEH C_18_ column (50 × 2.1 mm ID and particle size 1.7 μm), using H_2_O + 0.1% HCOOH (A) and CH_3_CN + 0.1% HCOOH as the mobile phase. The QC analysis was performed starting from a 10 mM stock solution of the test compound in DMSO-*d*_6_ and further diluted 20-fold with CH_3_CN–H_2_O (1:1) for analysis. The QC analyses were run on a Waters ACQUITY UPLC-MS system consisting of an SQD mass spectrometer equipped with an electrospray ionization interface and a photodiode array detector. Electrospray ionization in positive and negative modes was applied in the mass scan range 100–500 Da. The PDA range was 210–400 nm. The analyses were run on an ACQUITY UPLC BEH C_18_ column (100 × 2.1 mm ID and particle size 1.7 μm) with a VanGuard BEH C_18_ pre-column (5 × 2.1 mm ID and particle size 1.7 μm). The mobile phase was 10 mM NH_4_OAc in H_2_O at pH 5 adjusted with AcOH (A) and 10 mM NH_4_OAc in CH_3_CN–H_2_O (95:5) at pH 5 (B) with 0.5 mL/min as flow rate. A linear gradient was applied: 0–0.2 min: 10% B, 0.2–6.2 min: 10–90% B, 6.2–6.3 min: 90–100%, 6.3–7.0 min: 100% B (QC method). All final compounds displayed ≥95% purity as determined by NMR and UPLC-MS analysis (unless otherwise indicated). Compounds **1–8** were purchased from Sigma-Aldrich and used as such without further purification. Compounds **9–12** and **16–19** and intermediates **33b–d** and **35–36da** were synthesized as previously reported.^12^

#### General Procedure 1: Preparation of Compounds of Type 33 via Regioselective Nucleophilic Aromatic Sostitution

A solution (0.2 M) of 6-phenyl-2,4-dichloropyrimidine **30** (1.00 equiv) with a suitable aniline of type **32** (1.00 mmol) in THF was cooled to −60 °C. To this solution was added dropwise LiHMDS (1.0 M in THF, 2.5 equiv). After 1 h, water was added, and the mixture was extracted with EtOAc. The combined organic layers were washed with brine, dried (Na_2_SO_4_), and concentrated in vacuo. Final normal-phase chromatographic purification (cyclohexane/EtOAc) provided the desired product of type **33**.

#### General Procedure 2: Preparation of Compounds of Type 33 Via Buchwald–Hartwig Amination

A mixture of Pd(OAc)_2_ (0.05 equiv) and *rac*-BINAP (12.5 mg, 0.02 mmol, 0.05 equiv) in 1,4-dioxane (0.02 M solution) was stirred under Ar flushing for 10 min. Then, a solution (1 M) of intermediate **31** (1 equiv) and a solution (1 M) of suitable aniline of type **32** (1 equiv) in 1,4-dioxane (1 M mL) and Cs_2_CO_3_ (1.2 equiv) were stepwise added. The reaction mixture was stirred in a CEM microwave apparatus at 60 °C (200 W) for 4 h, filtrated through a Celite coarse patch, rinsed with DCM, and concentrated to dryness at low pressure. Final normal-phase chromatographic purification afforded the pure desired compound of type **33**.

#### General Procedure 3: Preparation of Compounds of Type 35 through Suzuki Cross-Coupling Reaction

A suspension of intermediate **33** (1 equiv), 4,4,5,5-tetramethylboronate of type **34** (1.2 equiv), dichloro[1,1′-bis(diphenylphosphino)ferrocene] palladium DCM complex (0.2 equiv), and K_2_CO_3_ 2 M solution (3 equiv) in 1,4-dioxane (0.04 M solution) was stirred in a CEM microwave apparatus at 120 °C (200 W) for 2 h. The resulting crude was portioned between DCM and NaHCO_3_ saturated solution, and the organic layer was dried over Na_2_SO_4_ and concentrated to dryness at low pressure. Final normal-phase chromatographic purification (cyclohexane/EtOAc) afforded the pure title compound of type **35**.

#### General Procedure 4. Preparation of Compounds of Type 36 or 20 after C=C Double-Bond Reduction

Under a N_2_ atmosphere, a suspension of the intermediate of type **35** (1 equiv), ammonium formate (4 equiv), and Pd(OH)_2_/C (20% of starting material weight) in MeOH dry (0.04 M solution) was stirred at reflux temperature until reaction completion. The catalyst was filtered off through a Celite coarse patch, and the resulting filtrate was concentrated to dryness at low pressure. Final chromatographic normal-phase purification (cyclohexane/EtOAc) afforded the pure desired compound of type **36**.

#### General Procedure 5: Final Boc Deprotection

To a 0 °C solution of the protected intermediate of type **36** (1 equiv) in 1,4-dioxane (0.02 M solution) was dropwise added a HCl (4 M) solution in 1,4-dioxane (10 equiv), and the reaction mixture was stirred at room temperature until completion of the conversion (from 4 to 24 h); then, the reaction crude was concentrated to dryness at low pressure, the resulting crude was portioned between EtOAc and NaOH 0.1 M, and the organic layer was dried over Na_2_SO_4_ and concentrated to dryness at low pressure. Final chromatographic purification by silica or alumina (DCM/MeOH NH_3_ 1 M) afforded pure final compounds.

##### 3-(4-((4-Chlorophenyl)amino)-6-(pyridin-4-yl)pyrimidin-2-yl)piperidin-1-ium Chloride (**1**)

Compound **1** was purchased from Aldrich. HRMS (ESI) *m*/*z* calcd for C_20_H_21_ClN_5_ [M + H]^+^, 366.1485; found, 366.1483. ^1^H NMR (400 MHz, DMSO-*d*_6_): δ 10.44 (s, 1H), 9.01 (bs, 2H), 9.00–8.94 (m, 2H), 8.44–8.34 (m, 2H), 7.83–7.73 (m, 2H), 7.46 (s, 1H), 7.44–7.40 (m, 2H), 3.73–3.61 (m, 2H), 3.33–3.21 (m, 2H), 2.93 (m, 1H), 2.25 (m, 1H), 1.95–1.67 (m, 3H).

##### ***N***-(4-Chlorophenyl)-2-(piperidin-3-yl)-6-(pyridin-3-yl)pyrimidin-4-amine (**2**)

Compound **2** was purchased from Aldrich. HRMS (ESI) *m*/*z*: calcd for C_20_H_21_ClN_5_ [M + H]^+^, 366.1485; found, 366.1481. ^1^H NMR (400 MHz, DMSO-*d*_6_): δ 9.85 (s, 1H), 9.18 (dd, *J* = 2.3, 0.9 Hz, 1H), 8.68 (dd, *J* = 4.8, 1.6 Hz, 1H), 8.37 (dt, *J* = 8.1, 1.9 Hz, 1H), 7.83–7.74 (m, 2H), 7.55 (ddd, *J* = 8.0, 4.8, 0.9 Hz, 1H), 7.43–7.34 (m, 2H), 7.10 (s, 1H), 3.22 (d, *J* = 10.8 Hz, 1H), 2.91 (d, *J* = 12.4 Hz, 1H), 2.88–2.70 (m, 2H), 2.10 (d, *J* = 12.8 Hz, 1H), 1.76 (qd, *J* = 12.6, 3.8 Hz, 1H), 1.70–1.61 (m, 1H), 1.48 (qt, *J* = 12.5, 3.6 Hz, 1H). Some signals overlap with water.

##### ***N***-(4-Chlorophenyl)-2-(piperidin-3-yl)-6-(pyridin-2-yl)pyrimidin-4-amine (**3**)

Compound **3** was purchased from Aldrich. HRMS (ESI) *m*/*z*: calcd for C_20_H_21_ClN_5_ [M + H]^+^, 366.1485; found, 366.1483. [M + H]^+^ calcd. ^1^H NMR (600 MHz, DMSO-*d*_6_): δ 9.85 (s, 1H), 8.70 (ddd, *J* = 4.8, 1.8, 0.9 Hz, 1H), 8.39 (dt, *J* = 7.9, 1.1 Hz, 1H), 7.98 (td, *J* = 7.7, 1.8 Hz, 1H), 7.82–7.75 (m, 2H), 7.64 (s, 1H), 7.52 (ddd, *J* = 7.5, 4.7, 1.2 Hz, 1H), 7.42–7.37 (m, 2H), 3.27 (d, *J* = 11.2 Hz, 1H), 2.95 (dd, *J* = 12.4, 3.8 Hz, 1H), 2.86 (tt, *J* = 10.4, 3.4 Hz, 1H), 2.83–2.76 (m, 1H), 2.54 (dd, *J* = 12.0, 3.0 Hz, 1H), 2.15–2.11 (m, 1H), 1.82–1.76 (m, 1H), 1.69 (dp, *J* = 13.4, 3.4 Hz, 1H), 1.52 (qt, *J* = 12.7, 3.9 Hz, 1H). ^13^C NMR (151 MHz, DMSO-*d*_6_): δ 171.8 (Cq), 161.6 (Cq), 161.0 (Cq), 154.4 (Cq), 150.0 (CH), 139.6 (Cq), 137.9 (CH), 129.1 (CH, 2C), 126.1 (Cq), 125.8 (CH), 121.5 (CH, 2C), 121.4 (CH), 100.8 (CH), 51.4 (CH_2_), 46.5 (CH_2_), 46.3 (CH), 30.3 (CH_2_), 26.3 (CH_2_).

##### ***N***-(4-Chlorophenyl)-6-phenyl-2-(piperidin-3-yl)pyrimidin-4-amine (**4**)

Compound **4** was purchased from Aldrich. HRMS (ESI) *m*/*z*: calcd for C_21_H_22_ClN_4_ [M + H]^+^, 365.1533; found, 365.1529. ^1^H NMR (400 MHz, DMSO-*d*_6_): δ 9.78 (s, 1H), 0.8.04–8.02 (m, 2H), 7.84–7.75 (m, 2H), 7.53–7.51 (m, 3H), 7.42–7.34 (m, 2H), 7.08 (s, 1H), 3.35–3.29 (m, 1H), 3.01 (d, *J* = 12.3 Hz, 1H), 2.94–2.84 (m, 2H), 2.60 (td, *J* = 12.0, 2.6 Hz, 1H), 2.15–2.12 (m, 1H), 1.83–1.67 (m, 2H), 1.56 (qt, *J* = 12.3, 3.4 Hz, 1H). ^13^C NMR (101 MHz, DMSO-*d*_6_): δ 170.8 (Cq), 161.4 (Cq), 161.0 (Cq), 139.2 (Cq), 137.2 (Cq), 130.3 (CH), 128.9 (CH, 2C), 128.6 (CH, 2C), 126.5 (CH, 2C), 125.5 (Cq), 121.0 (CH, 2C), 99.9 (CH), 50.2 (CH_2_), 45.6 (CH_2_), 45.1 (CH), 29.4 (CH_2_), 25.1 (CH_2_).

##### *N*-(4-Chlorophenyl)-6-(4-methoxyphenyl)-2-(piperidin-3-yl)pyrimidin-4-amine (**5**)

Compound **5** was purchased from Aldrich. HRMS (ESI) *m*/*z*: calcd for C_22_H_24_N_4_OCl [M + H]^+^, 395.1635; found, 395.1639. ^1^H NMR (600 MHz, DMSO-*d*_6_): δ 9.71 (s, 1H), 8.05–7.95 (m, 2H), 7.80–7.74 (m, 2H), 7.41–7.34 (m, 2H), 7.10–7.04 (m, 2H), 7.01 (s, 1H), 3.83 (s, 3H), 3.36–3.32 (m, 1H), 3.04 (td, *J* = 12.4, 3.5 Hz, 1H), 2.94–2.85 (m, 2H), 2.64 (td, *J* = 12.1, 3.0 Hz, 1H), 2.17–2.09 (m, 1H), 1.82–1.70 (m, 2H), 1.63–1.50 (qt, *J* = 12.4, 3.7 Hz, 1H). ^13^C NMR (151 MHz, DMSO-*d*_6_): δ 170.4 (Cq), 161.1 (Cq), 161.0 (Cq), 160.9 (Cq), 139.2 (Cq), 129.4 (Cq), 128.6 (CH, 2C), 128.0 (CH, 2C), 125.4 (Cq), 120.9 (CH, 2C), 114.2 (CH, 2C), 98.7 (CH), 55.3 (CH_3_), 49.8 (CH_2_), 45.3 (CH_2_), 44.7 (CH), 29.2 (CH_2_), 24.7 (CH_2_).

##### *N*-(3-Chlorophenyl)-2-(piperidin-3-yl)-6-(pyridin-4-yl)pyrimidin-4-amine (**6**)

Compound **6** was purchased from Aldrich. HRMS (ESI) *m*/*z*: calcd for C_20_H_21_N_5_Cl [M + H]^+^, 366.1485; found, 366.1488. ^1^H NMR (400 MHz, DMSO-*d*_6_): δ 10.02 (s, 1H), 8.79–8.70 (m, 2H), 8.13 (t, *J* = 2.1 Hz, 1H), 7.97–7.92 (m, 2H), 7.59 (dd, *J* = 8.0, 2.0 Hz, 1H), 7.37 (t, *J* = 8.1 Hz, 1H), 7.20 (s, 1H), 7.06 (m, *J* = 7.9, 2.0 Hz, 1H), 3.32 (d, *J* = 10.6 Hz, 1H), 2.99 (d, *J* = 12.4 Hz, 1H), 2.95–2.81 (m, 2H), 2.56 (t, *J* = 10.2 Hz, 1H), 2.16–2.13 (m, 1H), 1.88–1.74 (m, 1H), 1.74–1.66 (m, 1H), 1.54 (qd, *J* = 12.3, 3.9 Hz, 1H). ^13^C NMR (101 MHz, DMSO-*d*_6_): δ 171.5 (Cq), 161.0 (Cq), 159.2 (Cq), 150.5 (CH, 2C), 144.3 (Cq), 141.5 (Cq), 133.1 (Cq), 130.4 (CH), 121.7 (CH), 120.6 (CH, 2C), 119.0 (CH), 117.7 (CH), 101.4 (CH), 50.5 (CH_2_), 45.83 (CH_2_), 45.5 (CH), 29.54 (CH_2_), 25.39 (CH_2_).

##### *N*-(4-Fluorophenyl)-2-(piperidin-3-yl)-6-(pyridin-4-yl)pyrimidin-4-amine (**7**)

Compound **7** was purchased from Aldrich. HRMS (ESI) *m*/*z*: calcd for C_20_H_21_N_5_F [M + H]^+^, 350.1781; found, 350.1785. ^1^H NMR (600 MHz, DMSO-*d*_6_): δ 9.81 (s, 1H), 8.75–8.72 (m, 2H), 7.97–7.93 (m, 2H), 7.77–7.72 (m, 2H), 7.22–7.17 (m, 2H), 7.12 (s, 1H), 3.30 (d, *J* = 9.1 Hz, 2H), 2.99 (dt, *J* = 12.7, 3.6 Hz, 1H), 2.91–2.82 (m, 2H), 2.58 (td, *J* = 11.9, 2.8 Hz, 1H), 2.15–2.09 (m, 1H), 1.81–1.66 (m, 2H), 1.55 (qt, *J* = 12.3, 3.8 Hz, 1H). ^13^C NMR (151 MHz, DMSO-*d*_6_): δ 171.4 (Cq), 161.3 (Cq), 159.1, 153.8 (d, ^1^*J*_CF_ = 239.8 Hz, Cq), 150.6 (CH, 2C), 144.5 (Cq), 136.2 (Cq), 121.7 (CH, 2C), 120.8 (CH, 2C), 115.5 (d, ^2^*J*_CF_ = 22.3 Hz, CH, 2C), 100.8 (CH), 50.1 (CH_2_), 45.6 (CH_2_), 45.1 (CH), 29.5 (CH_2_), 25.1 (CH_2_).

##### *N*-Phenyl-2-(piperidin-3-yl)-6-(pyridin-4-yl)pyrimidin-4-amine (**8**)

Compound **8** was purchased from Aldrich. HRMS (ESI) *m*/*z*: calcd for C_20_H_22_N_5_ [M + H]^+^, 332.1875; found, 332.1865. ^1^H NMR (400 MHz, DMSO-*d*_6_): δ 9.80 (s, 1H), 8.77–8.73 (m, 2H), 7.97–7.93 (m, 2H), 7.77–7.73 (m, 2H), 7.40–7.34 (m, 2H), 7.18 (s, 1H), 7.05 (tt, *J* = 7.4, 1.2 Hz, 1H), 3.04 (d, *J* = 12.8 Hz, 1H), 2.93 (m, 2H), 2.64 (dd, *J* = 12.0, 2.4 Hz, 1H), 2.15 (d, *J* = 13.1 Hz, 1H), 1.86–1.68 (m, 2H), 1.64–1.58 (m, 1H) (one aliphatic signal of 1H overlaps with water).

##### *N*-(4-Chlorophenyl)-6-phenyl-2-(piperidin-4-yl)pyrimidin-4-amine (**13**)

Title compound **13** was prepared according to general procedure 5 using intermediate **36aa** (45 mg, 0.10 mmol) and HCl (4 M in 1,4-dioxane) (0.25 mL, 1.00 mmol) in 1,4-dioxane dry (1.0 mL). Final purification by neutral alumina (DCM/MeOH from 100/0 to 90/10) afforded pure title compound **13** (15 mg, 42% yield). UPLC-MS: *R*_*t*_ = 1.99 min (method 1). MS (ESI) *m*/*z*: 365.5 [M + H]^+^, C_21_H_22_ClN_4_ [M + H]^+^ calcd, 365.9. HRMS (ESI) *m*/*z*: calcd for C_21_H_22_N_4_Cl [M + H]^+^, 365.1533; found, 365.1524. ^1^H NMR (400 MHz, DMSO-*d*_6_): δ 9.75 (s, 1H), 8.08–7.98 (m, 2H), 7.85–7.76 (m, 2H), 7.57–7.44 (m, 3H), 7.43–7.31 (m, 2H), 7.07 (s, 1H), 3.07 (d, *J* = 12.3 Hz, 2H), 2.86–2.80 (m, 1H), 2.66 (t, *J* = 11.8 Hz, 2H), 1.94 (d, *J* = 12.8 Hz, 2H), 1.75 (qd, *J* = 12.2, 4.0 Hz, 2H). ^13^C NMR (101 MHz, DMSO-*d*_6_): δ 172.1 (Cq), 161.5 (Cq), 161.1 (Cq), 139.3 (Cq), 137.2 (Cq), 130.3 (CH), 128.9 (CH, 2C), 128.6 (CH, 2C), 126.5 (CH, 2C), 125.5 (Cq), 121.0 (CH, 2C), 99.9 (CH), 45.3 (CH_2_, 2C), 44.6 (CH), 30.5 (CH_2_, 2C).

##### *N*-(4-Methoxyphenyl)-6-phenyl-2-(piperidin-4-yl)pyrimidin-4-amine (**14**)

Title compound **14** was prepared according to general procedure 5 using intermediate **36ba** (106 mg, 0.23 mmol) and HCl (4 M in 1,4-dioxane) (0.58 mL, 2.30 mmol) in 1,4-dioxane dry (2.3 mL). Final normal-phase purification (DCM/DCM/NH_3_ 1 M MeOH 4:1 from 85/15 to 50/50) afforded pure title compound **14** (53 mg, yield 64%). UPLC-MS: *R*_*t*_ = 1.87 min (method 1). MS (ESI) *m*/*z*: 361.5 [M + H]^+^, C_22_H_25_N_4_O [M + H]^+^ calcd, 361.5. HRMS (ESI) *m*/*z*: calcd for C_22_H_25_N_4_O [M + H]^+^, 361.2028; found, 361.2029. ^1^H NMR (400 MHz, DMSO-*d*_6_): δ 9.44 (s, 1H), 8.14–7.89 (m, 2H), 7.64 (d, *J* = 8.9 Hz, 2H), 7.58–7.38 (m, 3H), 6.99 (s, 1H), 6.96–6.91 (m, 2H), 3.75 (s, 3H), 3.13 (d, *J* = 12.4 Hz, 2H), 2.83 (tt, *J* = 11.4, 3.8 Hz, 1H), 2.73 (td, *J* = 12.2, 2.7 Hz, 2H), 1.97 (dd, *J* = 12.8, 3.4 Hz, 2H), 1.81 (qd, *J* = 12.2, 4.0 Hz, 2H). ^13^C NMR (101 MHz, DMSO-*d*_6_): δ 172.0 (Cq), 161.4 (Cq), 161.0 (Cq), 154.7 (Cq), 137.5 (Cq), 133.2 (Cq), 130.0 (CH), 128.7 (CH, 2C), 126.4 (CH, 2C), 121.5 (CH, 2C), 114.0 (CH, 2C), 99.0 (CH), 55.2 (CH_3_), 45.2 (CH_2_, 2C), 44.4 (CH), 30.4 (CH_2_, 2C).

##### *N*1,*N*1-Dimethyl-*N*4-(6-phenyl-2-(piperidin-4-yl)pyrimidin-4-yl)benzene-1,4-diamine (**15**)

Title compound **14** was prepared according to general procedure 5 using intermediate **36ca** (109 mg, 0.23 mmol) and HCl (4 M in 1,4-dioxane) (0.58 mL, 2.30 mmol) in 1,4-dioxane dry (2.3 mL). Final normal-phase purification (DCM/DCM/NH_3_ 1 M MeOH 4:1 from 85/15 to 50/50) afforded pure title compound **14** (54 mg, yield 63%). UPLC-MS: *R*_*t*_ = 1.98 min (method 1). MS (ESI) *m*/*z*: 374.2 [M + H]^+^, C_23_H_28_N_5_ [M + H]^+^ calcd, 374.5. HRMS (ESI) *m*/*z*: calcd for C_23_H_28_N_5_ [M + H]^+^, 374.2345; found, 374.2341. ^1^H NMR (400 MHz, DMSO-*d*_6_): δ 9.25 (s, 1H), 8.17–7.82 (m, 2H), 7.69–7.37 (m, 5H), 6.93 (s, 1H), 6.83–6.66 (m, 2H), 3.08 (dt, *J* = 12.2, 3.4 Hz, 2H), 2.87 (s, 6H), 2.77 (tt, *J* = 11.5, 3.8 Hz, 1H), 2.66 (td, *J* = 12.1, 2.6 Hz, 2H), 1.93 (dd, *J* = 13.4, 3.5 Hz, 2H), 1.77 (qd, *J* = 13.0, 12.5, 4.0 Hz, 2H). ^13^C NMR (101 MHz, DMSO-*d*_6_): δ 172.2 (Cq), 161.6 (Cq), 160.9 (Cq), 146.7 (Cq), 137.6 (Cq), 129.9 (CH), 128.7 (CH, 2C), 126.3 (CH, 2C), 121.7 (CH, 2C), 113.0 (CH, 2C), 98.4 (CH), 45.6 (CH_2_, 2C), 44.9 (CH), 40.6 (CH_3_, 2C), 31.0 (CH_2_, 2C).

##### ***N***1-(2-Cyclohexyl-6-phenylpyrimidin-4-yl)-*N*3,*N*3-dimethylbenzene-1,3-diamine (**20**)

Title compound **14** was prepared according to general procedure 4 using intermediate **35db** (60 mg, 0.16 mmol), NH_4_COOH (61 mg, 0.96 mmol), and Pd(OH)_2_/C (12 mg) in MeOH dry (2.7 mL). Final normal-phase purification (Cyclohexane/EtOAc from 100/0 to 90/10) afforded pure title compound **20** (35 mg, yield 58%). UPLC-MS: *R*_*t*_ = 1.45 min (method 3). MS (ESI) *m*/*z*: 373.3 [M + H]^+^, C_24_H_29_N_4_ [M + H]^+^ calcd, 373.5. HRMS (ESI) *m*/*z*: calcd for C_24_H_29_N_4_ [M + H]^+^, 373.2392; found, 373.2393. ^1^H NMR (400 MHz, DMSO-*d*_6_) 9.40 (s, 1H), 8.06–7.95 (m, 2H), 7.56–7.44 (m, 4H), 7.12 (t, *J* = 8.1 Hz, 1H), 7.05 (s, 1H), 6.86 (dd, *J* = 7.8, 1.4 Hz, 1H), 6.40 (dd, *J* = 8.1, 2.4 Hz, 1H), 2.93 (s, 6H), 2.72 (tt, *J* = 11.6, 3.5 Hz, 1H), 2.06–1.96 (m, 2H), 1.81 (dt, *J* = 12.6, 3.4 Hz, 2H), 1.76–1.57 (m, 3H), 1.47–1.32 (m, 2H), 1.24 (tt, *J* = 12.7, 3.5 Hz, 1H). ^13^C NMR (101 MHz, DMSO-*d*_6_): δ 173.1 (Cq), 161.4 (Cq), 161.1 (Cq), 150.9 (Cq), 141.0 (Cq), 137.5 (Cq), 130.0 (CH), 129.0 (CH), 128.8 (CH, 2C), 126.4 (CH, 2C), 107.7 (CH), 106.6 (CH), 103.9 (CH), 99.6 (CH), 46.9 (CH), 40.2 (CH_3_, 2C), 31.6 (CH_2_, 2C), 25.8 (CH_2_), 25.8 (CH_2,_ 2C).

##### 1-(4-(4-((3-(Dimethylamino)phenyl)amino)-6-phenylpyrimidin-2-yl)piperidin-1-yl)-2-methylpropan-1-one (**21**)

To a solution of compound 17 (30 mg, 0.08 mmol) in DMF dry (1 mL) were added isobutyric acid (8 μL, 0.09 mmol), HATU (37 mg, 0.1 mmol), and DIPEA (0.04 mL, 0.24 mmol) under argon. The reaction mixture was stirred at room temperature for 2 h; after that, water (2 mL) was added, and the aqueous layer was extracted with EtOAc (3 × 3 mL). The collected organic layer was washed with NaHCO_3_ (2 mL), dried over Na_2_SO_4_, filtered, and concentrated under vacuum. Final normal-phase purification (cyclohexane/EtOAc from 100/0 to 60/40) afforded pure title compound **21** (21 mg, 59% yield). UPLC-MS: *R*_*t*_ = 1.53 min (method 2), no ionization. HRMS (ESI) *m*/*z*: calcd for C_27_H_34_N_5_O [M + H]^+^, 444.2763; found, 444.2765. ^1^H NMR (400 MHz, CDCl_3_): δ 7.99–7.94 (m, 2H), 7.46–7.42 (m, 3H), 7.24 (t, *J* = 8.1 Hz, 1H), 7.01 (s, 1H), 6.99 (bs, 1H), 6.80 (t, *J* = 2.2 Hz, 1H), 6.70 (dd, *J* = 7.6, 2.0 Hz, 1H), 6.56 (dd, *J* = 8.3, 2.5 Hz, 1H), 4.73 (d, *J* = 13.2 Hz, 1H), 4.06 (d, *J* = 13.6 Hz, 1H), 3.20 (t, *J* = 12.7 Hz, 1H), 3.04 (tt, *J* = 11.5, 3.9 Hz, 1H), 2.98 (s, 6H), 2.86 (p, *J* = 6.7 Hz, 1H), 2.82–2.71 (m, 1H), 2.20–2.06 (m, 2H), 1.98–2.87 (m, 2H), 1.16 (t, *J* = 7.8 Hz, 6H). ^13^C NMR (101 MHz, CDCl_3_): δ 175.4 (Cq), 172.1 (Cq), 163.9 (Cq), 162.1 (Cq), 151.7 (Cq), 139.4 (Cq), 137.9 (Cq), 130.3 (CH), 130.1 (CH), 128.8 (CH, 2C), 127.2 (CH, 2C), 110.4 (CH), 109.2 (CH), 106.4 (CH), 97.8 (CH), 45.6 (CH_2_), 45.3 (CH), 42.0 (CH_2_), 40.7 (CH_3_, 2C), 30.7 (CH_2_, 2C), 30.3 (CH), 19.8 (CH_3_, 2C).

##### *N*1-(2-(1-(2-Methoxyethyl)piperidin-4-yl)-6-phenylpyrimidin-4-yl)-*N*3,*N*3-dimethylbenzene-1,3-diamine (**22**)

DIPEA (0.03 mL, 0.16 mmol) and 1-bromo-3-methoxypropane (14 μL, 0.14 mmol) were added to a solution of compound 17 (50 mg, 0.13 mmol) in CH_3_CN dry (1.3 mL) under argon. The reaction mixture was stirred at 60 °C for 2 days. After that, water (2 mL) was added, and the aqueous layer was extracted with EtOAc (2 mL × 3). Collected organic layers were dried over Na_2_SO_4_, filtered, and concentrated under vacuum. Final normal-phase purification (cyclohexane/EtOAc from 100/0 to 40/60) afforded pure title compound **22** (22 mg, 38% yield). UPLC-MS: *R*_*t*_ = 2.09 min (method 1), MS (ESI) *m*/*z*: 432.3 [M + H]^+^, C_26_H_34_N_5_O [M + H]^+^ calcd, 432.6. HRMS (ESI) *m*/*z*: calcd for C_26_H_34_N_5_O [M + H]^+^, 432.2763; found, 432.2752. ^1^H NMR (400 MHz, DMSO-*d*_6_): δ 9.59 (bs, 1H), 8.08–7.96 (m, 2H), 7.58–7.44 (m, 3H), 7.19 (s, 1H), 7.17–7.10 (m, 2H), 7.04 (d, *J* = 8.1 Hz, 1H), 6.42 (dd, *J* = 8.3, 2.5 Hz, 1H), 3.71–3.62 (bs, 3H), 3.32 (s, 3H), 3.28–2.97 (m, 6H), 2.92 (s, 6H), 2.28–2.08 (m, 4H). ^13^C NMR (101 MHz, DMSO-*d*_6_): δ 161.5 (Cq), 161.1 (Cq), 151.0 (Cq), 140.7 (Cq), 137.2 (Cq), 130.3 (CH), 129.2 (CH), 128.9 (CH, 2C), 126.4 (CH, 2C), 108.1 (CH), 107.1 (CH), 104.0 (CH), 100.0 (CH), 66.0 (CH_2_), 58.2 (CH_3_), 55.3 (CH_2_, 2C, recovered from HSQC), 52.6 (CH_2_, recovered from HSQC), 40.3 (CH_3_, 2C), 27.6 (CH_2_, 2C, recovered from HSQC).

##### ***N***-(2-Methoxyphenyl)-6-phenyl-2-(piperidin-4-yl)pyrimidin-4-amine (**23**)

Title compound **23** was prepared according to general procedure 5 using intermediate **36ea** (60 mg, 0.13 mmol) and HCl (4 M in 1,4-dioxane) (0.33 mL, 1.30 mmol) in 1,4-dioxane dry (1.3 mL). Final purification by alumina (DCM/MeOH·NH_3_ 1 M from 100/0 to 95/5) afforded pure title compound **23** (25 mg, yield 53%). UPLC-MS: *R*_*t*_ = 1.92 min (method 1). MS (ESI) *m*/*z*: 361.3 [M + H]^+^, C_22_H_25_N_4_O [M + H]^+^ calcd, 361.5. HRMS (ESI) *m*/*z*: calcd for C_22_H_25_N_4_O [M + H]^+^, 361.2028; found, 361.2016. ^1^H NMR (400 MHz, DMSO-*d*_6_): δ 8.70 (s, 1H), 8.26 (d, *J* = 7.9 Hz, 1H), 8.08–7.99 (m, 2H), 7.57–7.45 (m, 3H), 7.30 (s, 1H), 7.11–7.01 (m, 2H), 7.00–6.96 (m, 1H), 3.88 (s, 3H), 3.09–2.98 (m, 2H), 2.76 (dt, *J* = 12.1, 3.0 Hz, 1H), 2.60 (tt, *J* = 12.1, 2.6 Hz, 2H), 1.89 (d, *J* = 12.8 Hz, 2H), 1.71 (qd, *J* = 12.2, 4.0 Hz, 2H). ^13^C NMR (101 MHz, DMSO-*d*_6_): δ 172.4 (Cq), 161.6 (Cq), 161.2 (Cq), 149.8 (Cq), 137.5 (Cq), 130.0 (CH), 128.7 (CH, 2C), 128.5 (Cq), 126.4 (CH, 2C), 123.3 (CH), 121.7 (CH), 120.4 (CH), 111.2 (CH), 99.8 (CH), 55.7 (CH_3_), 46.1 (CH_2_, 2C), 45.6 (CH), 31.7 (CH_2_, 2C).

##### *N*1,*N*1-Dimethyl-*N*2-(6-phenyl-2-(piperidin-4-yl)pyrimidin-4-yl)benzene-1,2-diamine (**24**)

Title compound **24** was prepared according to general procedure 5 using intermediate **36fa** (100 mg, 0.21 mmol) and HCl (4 M in 1,4-dioxane) (0.53 mL, 2.1 mmol) in 1,4-dioxane dry (2.1 mL). Final normal-phase purification (DCM/MeOH·NH_3_ 1 M from 100/0 to 95/5) afforded pure title compound **24** (13 mg, 16% yield). UPLC-MS: *R*_*t*_ = 2.06 min (method 1). MS (ESI) *m*/*z*: 374.3 [M + H]^+^, C_23_H_28_N_5_ [M + H]^+^ calcd, 374.5. HRMS (ESI) *m*/*z*: calcd for C_23_H_28_N_5_ [M + H]^+^, 374.2345; found, 374.2341.^1^H NMR (400 MHz, DMSO-*d*_6_): δ 8.64 (s, 1H), 8.25–8.14 (m, 1H), 8.10–8.00 (m, 2H), 7.55–7.44 (m, 3H), 7.36 (s, 1H), 7.18 (td, *J* = 7.7, 2.0 Hz, 1H), 7.07 (td, *J* = 7.5, 5.5 Hz, 1H), 7.03 (td, *J* = 7.6, 1.9 Hz, 1H), 3.00 (dt, *J* = 12.2, 2.0 Hz, 2H), 2.76 (tt, *J* = 11.4, 3.7 Hz, 1H), 2.65 (s, 6H), 2.63–2.56 (m, 2H), 1.90 (d, *J* = 12.7 Hz, 2H), 1.72 (qd, *J* = 12.2, 4.0 Hz, 2H). ^13^C NMR (151 MHz, DMSO-*d*_6_): δ 172.2 (Cq), 161.4 (Cq), 161.3 (Cq), 144.5 (Cq), 137.5 (Cq), 133.2 (Cq), 130.1 (CH), 128.8 (2C), 126.5 (CH, 2C), 123.2 (CH), 123.1 (CH), 121.7 (CH), 119.3 (CH), 99.9 (CH), 45.7 (CH_2_, 2C), 45.1 (CH), 43.8 (CH_3_, 2C), 31.2 (CH_2_, 2C).

##### *N*-(3,4-Dimethoxyphenyl)-6-phenyl-2-(piperidin-4-yl)pyrimidin-4-amine (**25**)

Title compound **25** was prepared according to general procedure 5 using intermediate **36ga** (40 mg, 0.08 mmol) and HCl (4 M in 1,4-dioxane) (0.20 mL, 0.8 mmol) in 1,4-dioxane dry (0.8 mL). Final purification by alumina (DCM/MeOH·NH_3_ 1 M from 100/0 to 98/2) afforded pure title compound **25** (20 mg, 63% yield). UPLC-MS: *R*_*t*_ = 1.79 min (method 1). MS (ESI) *m*/*z*: 391.2 [M + H]^+^, C_23_H_27_N_4_O_2_ [M + H]^+^ calcd, 391.5. HRMS (ESI) *m*/*z*: calcd for C_23_H_27_N_4_O_2_ [M + H]^+^, 391.2134; found, 391.2136.^1^H NMR (400 MHz, DMSO-*d*_6_): δ 8.05–7.95 (m, 2H), 7.62 (s, 1H), 7.55–7.43 (m, 3H), 7.14 (dd, *J* = 8.6, 2.5 Hz, 1H), 6.98 (s, 1H), 6.93 (d, *J* = 8.7 Hz, 1H), 3.80 (s, 3H), 3.74 (s, 3H), 3.04 (dt, *J* = 11.9, 2.7 Hz, 2H), 2.77 (tt, *J* = 11.5, 3.8 Hz, 1H), 2.61 (td, *J* = 12.1, 2.5 Hz, 2H), 1.93 (d, *J* = 12.8 Hz, 2H), 1.74 (qd, *J* = 12.2, 4.0 Hz, 2H). ^13^C NMR (101 MHz, DMSO-*d*_6_): δ 172.1 (Cq), 161.3 (Cq), 161.1 (Cq), 148.7 (Cq), 144.2 (Cq), 137.5 (Cq), 133.8 (Cq), 130.1 (CH), 128.8 (CH, 2C), 126.4 (CH, 2C), 112.4 (CH), 111.5 (CH), 105.2 (CH), 99.2 (CH), 55.8 (CH_3_), 55.4 (CH_3_), 45.3 (CH_2_, 2C), 44.7 (CH), 30.8 (CH_2_, 2C).

##### *N*-(3-(2-Methoxyethoxy)phenyl)-6-phenyl-2-(piperidin-4-yl)pyrimidin-4-amine (**26**)

Title compound **26** was prepared according to general procedure 5 using intermediate **36ha** (51 mg, 0.1 mmol) and HCl (4 M in 1,4-dioxane) (0.25 mL, 1.0 mmol) in 1,4-dioxane dry (1.0 mL). Final purification by alumina (DCM/MeOH·NH_3_ 1 N from 100/0 to 98/2) afforded pure title compound **26** (12 mg, 30% yield). TLC: *R*_*f*_ = 0.3 (DCM/MeOH·NH_3_ 1 N, 98:2). HRMS (ESI) *m*/*z*: calcd for C_24_H_29_N_4_O_2_ [M + H]^+^, 405.2291; found, 405.2291.^1^H NMR (400 MHz, DMSO-*d*_6_) 9.59 (s, 1H), 8.09–7.98 (m, 2H), 7.66 (d, *J* = 2.3 Hz, 1H), 7.57–7.45 (m, 3H), 7.28–7.15 (m, 2H), 7.06 (s, 1H), 6.66–6.51 (m, 1H), 4.16–4.06 (m, 2H), 3.72–3.64 (m, 2H), 3.03 (dt, *J* = 12.2, 3.4 Hz, 2H), 2.79 (tt, *J* = 11.5, 3.8 Hz, 1H), 2.61 (td, *J* = 12.1, 2.5 Hz, 2H), 1.93 (d, *J* = 12.1 Hz, 2H), 1.74 (qd, *J* = 12.2, 4.0 Hz, 2H). ^13^C NMR (101 MHz, DMSO-*d*_6_): δ 172.6 (Cq), 161.3 (Cq), 161.3 (Cq), 158.8 (Cq), 141.6 (Cq), 137.4 (Cq), 130.2 (CH), 129.5 (CH), 128.8 (CH, 2C), 126.5 (CH, 2C), 111.7 (CH), 108.5 (CH), 105.4 (CH), 99.8 (CH), 70.4 (CH_2_), 66.9 (CH_2_), 58.2 (CH_3_), 46.1 (CH_2_, 2C), 45.6 (CH), 31.8 (CH_2_, 2C).

##### 6-Phenyl-2-(piperidin-4-yl)-*N*-(3-(trifluoromethyl)phenyl)pyrimidin-4-amine (**27**)

Title compound **27** was prepared according to general procedure 5 using intermediate **36ia** (90 mg, 0.18 mmol) and HCl (4 M in 1,4-dioxane) (0.45 mL, 1.8 mmol) in 1,4-dioxane dry (1.8 mL). Final purification by alumina (DCM/MeOH·NH_3_ 1 N from 100/0 to 95/5) afforded pure title compound **27** (69 mg, 96% yield). UPLC-MS: *R*_*t*_ = 2.04 min (method 1). MS (ESI) *m*/*z*: 399.2 [M + H]^+^, C_22_H_22_F_3_N_4_ [M + H]^+^ calcd, 399.4. HRMS (ESI) *m*/*z*: calcd for C_22_H_22_N_4_F_3_ [M + H]^+^, 399.1797; found, 399.1801.^1^H NMR (400 MHz, DMSO-*d*_6_) 9.98 (s, 1H), 8.53 (d, *J* = 2.3 Hz, 1H), 8.12–7.99 (m, 2H), 7.87 (dd, *J* = 8.5, 2.2 Hz, 1H), 7.61–7.46 (m, 4H), 7.32 (d, *J* = 7.7 Hz, 1H), 7.10 (s, 1H), 3.03 (dt, *J* = 12.0, 3.3 Hz, 2H), 2.82 (tt, *J* = 11.6, 3.7 Hz, 1H), 2.62 (td, *J* = 12.0, 2.5 Hz, 2H), 1.97 (dd, *J* = 13.5, 3.6 Hz, 2H), 1.72 (qd, *J* = 12.1, 4.0 Hz, 2H). ^13^C NMR (101 MHz, DMSO-*d*_6_): δ 172.5 (Cq), 161.7 (Cq), 161.0 (Cq), 141.2 (Cq), 137.2 (Cq), 130.3 (CH), 129.8 (CH), 129.5 (q, ^2^*J*_CF_ = 31.2 Hz, Cq), 128.8 (CH, 2C), 126.5 (CH, 2C), 124.3 (q, ^1^*J*_CF_ = 276.2 Hz, Cq), 122.5 (CH), 117.8 (q, ^3^*J*_CF_ = 3.7 Hz, CH), 115.4 (q, ^3^*J*_CF_ = 4.0 Hz, CH), 100.0 (CH), 46.2 (CH_2_, 2C), 45.4 (CH), 31.8 (CH_2_, 2C).

##### *N*1,*N*1-Dimethyl-*N*3-(4-phenyl-6-(piperidin-4-yl)-1,3,5-triazin-2-yl)benzene-1,3-diamine (**28**)

Title compound **28** was prepared after two steps from intermediate **41**. Step 1. The double C=C bond was reduced following general procedure 4 using intermediate **41** (70 mg, 0.15 mmol), NH_4_COOH (57 mg, 0.90 mmol), and Pd(OH)_2_/C (14 mg). Final normal-phase purification (cyclohexane/EtOAc from 100/0 to 75/25) afforded compound *tert*-butyl 4-(4-((3-(dimethylamino)phenyl)amino)-6-phenyl-1,3,5-triazin-2-yl)piperidine-1-carboxylate (49 mg, 70% yield). UPLC-MS: *R*_*t*_ = 2.42 min (method 2). MS (ESI) *m*/*z*: 475.4 [M + H]^+^, C_27_H_35_N_6_O_2_ [M + H]^+^ calcd, 475.6. ^1^H NMR (400 MHz, CDCl_3_): δ 8.54–7.47 (m, 2H), 7.57–7.51 (m, 1H), 7.49–7.43 (m, 2H), 7.35 (t, *J* = 2.2 Hz, 1H), 7.31 (s, 1H), 7.23 (t, *J* = 8.1 Hz, 1H), 6.91 (dd, *J* = 7.9, 2.0 Hz, 1H), 6.51 (dd, *J* = 8.3, 2.5 Hz, 1H), 4.21 (bs, 2H), 3.01 (s, 6H), 2.96–2.79 (m, 3H), 1.90 (qd, *J* = 11.5, 3.6 Hz, 2H), 1.49 (s, 9H). Step 2. The Boc-protective group was removed following general procedure 5 using *tert*-butyl 4-(4-((3-(dimethylamino)phenyl)amino)-6-phenyl-1,3,5-triazin-2-yl)piperidine-1-carboxylate (50 mg, 0.11 mmol) and HCl (4 M in 1,4-dioxane) (0.27 mL, 1.1 mmol) in 1,4-dioxane dry (1.1 mL). Final purification by alumina (DCM/MeOH from 100/0 to 95/5) afforded pure title compound **28** (30 mg, 73% yield). UPLC-MS: *R*_*t*_ = 2.10 min (method 1). MS (ESI) *m*/*z*: 375.3 [M + H]^+^, C_22_H_27_N_6_ [M + H]^+^ calcd, 375.5. HRMS (ESI) *m*/*z*: calcd for C_22_H_27_N_6_ [M + H]^+^, 375.2297; found, 375.2293.^1^H NMR (400 MHz, DMSO-*d*_6_): δ 9.97 (s, 1H), 8.50–8.32 (m, 2H), 7.64–7.58 (m, 1H), 7.58–7.50 (m, 2H), 7.44 (t, *J* = 2.2 Hz, 1H), 7.15 (t, *J* = 8.0 Hz, 1H), 7.07 (dt, *J* = 8.3, 1.2 Hz, 1H), 6.46 (dd, *J* = 8.0, 2.5 Hz, 1H), 3.03 (dt, *J* = 12.3, 3.4 Hz, 2H), 2.93 (s, 6H), 2.80–2.68 (m, 1H), 2.59 (td, *J* = 12.1, 2.5 Hz, 2H), 1.91 (d, *J* = 12.6 Hz, 2H), 1.72 (qd, *J* = 12.1, 4.0 Hz, 2H). ^13^C NMR (101 MHz, DMSO-*d*_6_): δ 170.1 (Cq), 164.2 (Cq), 150.8 (Cq), 139.8 (Cq), 136.1 (Cq), 132.2 (CH), 128.9 (CH), 128.6 (CH, 2C), 128.1 (CH, 2C), 108.6 (CH), 107.5 (CH), 104.6 (CH), 45.9 (CH_2_, 2C), 45.0 (CH), 40.2 (CH_3_), 31.0 (CH_2_, 2C).

##### 4-Phenyl-6-(piperidin-4-yl)-*N*-(3-(trifluoromethyl)phenyl)-1,3,5-triazin-2-amine (**29**)

Title compound **29** was prepared after two steps from intermediate **44**. Step 1. The double C=C bond was reduced following general procedure 4 using intermediate **44** (130 mg, 0.26 mmol), NH_4_COOH (98 mg, 1.57 mmol), and Pd(OH)_2_/C (26 mg). Final normal-phase purification (cyclohexane/EtOAc from 100/0 to 80/20) afforded compound *tert*-butyl 4-(4-phenyl-6-((3-(trifluoromethyl)phenyl)amino)-1,3,5-triazin-2-yl)piperidine-1-carboxylate (113 mg, 87% yield). UPLC-MS: *R*_*t*_ = 2.52 min (method 2). MS (ESI) *m*/*z*: 500.3 [M + H]^+^, C_26_H_29_F_3_N_5_O_2_ [M + H]^+^ calcd, 500.5. ^1^H NMR (400 MHz, CDCl_3_): δ 8.48 (d, *J* = 7.7 Hz, 2H), 8.30 (s, 1H), 7.75 (d, *J* = 8.1 Hz, 1H), 7.59–7.54 (m, 1H), 7.53–7.48 (m, 3H), 7.44 (s, 1H), 7.38 (d, *J* = 7.8 Hz, 1H), 4.22 (bs, 2H), 2.94–2.91 (m, 3H), 2.08 (d, *J* = 13.1 Hz, 2H), 1.95–1.82 (m, 2H), 1.49 (s, 9H). Step 2. The Boc-protective group was removed following general procedure 5 using *tert*-butyl 4-(4-phenyl-6-((3-(trifluoromethyl)phenyl)amino)-1,3,5-triazin-2-yl)piperidine-1-carboxylate (74 mg, 0.15 mmol) and HCl (4 M in 1,4-dioxane) (0.38 mL, 1.5 mmol) in 1,4-dioxane dry (1.5 mL). Final purification by alumina (DCM/MeOH·NH_3_ 1 N from 100/0 to 90/10) afforded pure title compound **29** (43 mg, 71% yield). UPLC-MS: *R*_*t*_ = 2.19 min (method 1). MS (ESI) *m*/*z*: 400.2 [M + H]^+^, C_21_H_21_F_3_N_5_ [M + H]^+^ calcd, 500.5. HRMS (ESI) *m*/*z*: calcd for C_21_H_21_N_5_F_3_ [M + H]^+^, 400.1749; found, 400.1747.^1^H NMR (400 MHz, DMSO-*d*_6_): δ 10.53 (s, 1H), 8.48 (d, *J* = 2.6 Hz, 1H), 8.45–8.35 (m, 2H), 8.00 (dd, *J* = 8.1, 2.1 Hz, 1H), 7.68–7.53 (m, 4H), 7.41 (d, *J* = 7.7 Hz, 1H), 3.04 (dt, *J* = 12.3, 3.4 Hz, 2H), 2.85–2.71 (m, 1H), 2.61 (td, *J* = 12.1, 2.5 Hz, 2H), 1.95 (d, *J* = 12.5 Hz, 2H), 1.80–1.65 (m, 2H). ^13^C NMR (101 MHz, DMSO-*d*_6_): δ 181.6 (Cq), 170.4 (Cq), 164.3 (Cq), 140.0 (Cq), 135.8 (Cq), 132.4 (CH), 129.9 (CH), 129.5 (q, ^2^*J*_CF_ = 32.1, Cq), 128.7 (CH, 2C), 128.2 (CH, 2C), 124.3 (q, ^1^*J*_CF_ = 272.9 Hz, Cq) 123.6 (CH), 119.0 (CH), 116.4 (CH), 45.8 (CH_2_, 2C), 44.9 (CH), 30.9 (CH_2_, 2C).

##### 3-(2-Methoxyethoxy)aniline (**32h**)

NaH (60% dispersion in mineral oil) (121.0 mg, 3.01 mmol) was added to a solution of 3-aminophenol (220.0 mg, 2.01 mmol) in THF dry (4.0 mL) under argon at room temperature. After 10 min, 1-bromo-3-methoxypropane (0.23 mL, 2.61 mmol) was slowly added, and the reaction mixture was stirred for 2 days. After that, NH_4_Cl aqueous sat solution (4 mL) was slowly added, and the aqueous layer was extracted with EtOAc (5 mL × 2). Collected organic layers were dried over Na_2_SO_4_, filtered, and concentrated under vacuum. Final normal-phase purification (cyclohexane/EtOAc from 100/0 to 70/30) afforded pure title compound **32h** (270.0 mg, 80% yield). UPLC-MS: *R*_*t*_ = 1.34 min (method 1). MS (ESI) *m*/*z*: 168.1 [M + H]^+^, C_9_H_14_NO_2_ [M + H]^+^ calcd, 168.2. ^1^H NMR (400 MHz, CDCl_3_): δ 7.05 (t, *J* = 7.8 Hz, 1H), 6.39–6.27 (m, 3H), 4.10–4.05 (m, 2H), 3.75–3.69 (m, 2H).

##### 2-Chloro-*N*-(4-chlorophenyl)-6-phenylpyrimidin-4-amine (**33a**)

Title compound **33a** was prepared according to general procedure 1 using intermediate **31** (245 mg, 1.09 mmol) and 4-chloroaniline **32a** (151.0 mg, 1.20 mmol). Final normal-phase purification (cyclohexane/EtOAc from 100/0 to 85/15) afforded pure title compound **33a** (310.0 mg, 90% yield). UPLC-MS: *R*_*t*_ = 1.96 min (method 2). MS (ESI) *m*/*z*: 316.1 [M + H]^+^, C_17_H_11_Cl_2_N_3_ [M + H]^+^ calcd, 317.2. ^1^H NMR (400 MHz, CDCl_3_): δ 7.97–7.85 (m, 2H), 7.52–7.36 (m, 5H), 7.33 (d, *J* = 8.8 Hz, 2H), 7.22 (s, 1H), 6.90 (s, 1H). ^13^C NMR (101 MHz, CDCl_3_): δ 166.6 (Cq), 163.2 (Cq), 161.2 (Cq), 136.1 (Cq, 2C), 131.2 (CH), 131.2 (Cq), 129.9 (CH, 2C), 129.0 (CH, 2C), 127.3 (CH, 2C), 124.5 (CH, 2C), 98.0 (CH).

##### 2-Chloro-*N*-(4-methoxyphenyl)-6-phenylpyrimidin-4-amine (**33b**)

Title compound **33b** was prepared according to general procedure 2 using intermediate **31** (100 mg, 0.44 mmol) and *p*-methoxyaniline **32b** (55.2 mg, 0.44 mmol). Final normal-phase purification (cyclohexane/TBME from 95/5 to 75/25) afforded pure title compound **33b** (86.8 mg, 63% yield). UPLC-MS: *R*_*t*_ = 1.39 min (method 2); MS (ESI) *m*/*z*: 312.1 [M – H]^+^, [M – H]^+^ calcd: 312.1. 1H NMR (400 MHz, DMSO-*d*_6_): δ 9.91 (s, 1H), 8.06–7.84 (m, 2H), 7.65–7.37 (m, 5H), 7.06 (s, 1H), 7.02–6.92 (m, 2H), 3.76 (s, 3H).

##### ***N***1-(2-Chloro-6-phenylpyrimidin-4-yl)-*N*4,*N*4-dimethylbenzene-1,4-diamine (**33c**)

Titled compound **33c** was prepared according to general procedure 2 using intermediate **31** (300 mg, 1.33 mmol) and *N*1,*N*1-dimethylbenzene-1,4-diamine **32c** (191.1 mg, 1.33 mmol). Final normal-phase purification (cyclohexane/TBME from 100/0 to 80/20) afforded pure title compound **33c** (272 mg, 63% yield). UPLC-MS: *R*_*t*_ = 1.56 min (method 2); MS (ESI) *m*/*z*: 325.1 [M + H]^+^, [M + H]^+^ calcd: 325.1. ^1^H NMR (400 MHz, DMSO-*d*_6_): δ 9.76 (s, 1H), 7.93 (dd, *J* = 6.7, 3.0 Hz, 2H), 7.52 (dd, *J* = 4.6, 2.4 Hz, 3H), 7.36 (s, 2H), 7.00 (s, 1H), 6.87–6.64 (m, 2H), 2.89 (s, 6H).

##### *N*1-(2-Chloro-6-phenylpyrimidin-4-yl)-*N*3,*N*3-dimethylbenzene-1,3-diamine (**33d**)

Titled compound **33d** was prepared according to general procedure 2 using intermediate **31** (300 mg, 1.13 mmol) and N1,N1-dimethylbenzene-1,3-diamine **32d** (181.5 mg, 1.33 mmol). Final normal-phase purification (cyclohexane/TBME from 100/0 to 80/20) afforded pure title compound **33d** (246 mg, 67% yield). UPLC-MS: *R*_*t*_ = 1.73 min (method 2); MS (ESI) *m*/*z*: 325.1 [M + H]^+^, [M + H]^+^ calcd: 325.1. 1H NMR (400 MHz, DMSO-*d*_6_): δ 9.93 (s, 1H), 8.14–7.82 (m, 2H), 7.60–7.47 (m, 3H), 7.29–7.09 (m, 2H), 7.04 (s, 1H), 6.99–6.85 (m, 1H), 6.51 (ddd, *J* = 8.4, 2.5, 0.8 Hz, 1H), 2.92 (s, 6H).

##### 2-Chloro-*N*-(2-methoxyphenyl)-6-phenylpyrimidin-4-amine (**33e**)

Title compound **33e** was prepared according to general procedure 1 using intermediate **31** (180 mg, 0.80 mmol) and 2-methoxyaniline **32e** (0.07 mL, 0.55 mmol). Final normal-phase purification (cyclohexane/EtOAc from 100/0 to 85/15) afforded pure title compound **33e** (230 mg, 91% yield). UPLC-MS: *R*_*t*_ = 1.57 min (method 2). MS (ESI) *m*/*z*: 312.1 [M + H]^+^, C_17_H_15_ClN_3_O [M + H]^+^ calcd, 312.8. ^1^H NMR (400 MHz, CDCl_3_): δ 7.96 (m, 2H), 7.91 (d, *J* = 7.9 Hz, 1H), 7.51–7.41 (m, 3H), 7.32 (s, 1H), 7.15 (td, *J* = 7.8, 1.6 Hz, 1H), 7.04 (td, *J* = 7.7, 1.4 Hz, 1H), 7.00 (s, 1H), 6.97 (dd, *J* = 8.1, 1.4 Hz, 1H), 3.90 (s, 3H). ^13^C NMR (101 MHz, CDCl_3_): δ 166.0 (Cq), 162.6 (Cq), 161.2 (Cq), 150.3 (Cq), 136.4 (Cq), 131.0 (Cq), 128.9 (CH, 2C), 127.3 (CH, 2C), 125.1 (CH), 121.6 (CH), 121.2 (CH), 111.1 (CH), 99.0 (CH), 55.9 (CH_3_).

##### *N*1-(2-Chloro-6-phenylpyrimidin-4-yl)-*N*2,*N*2-dimethylbenzene-1,2-diamine (**33f**)

Title compound **33f** was prepared according to general procedure 1 using intermediate **31** (112 mg, 0.50 mmol) and *N*1,*N*1-dimethylbenzene-1,2-diamine **32f** (0.10 mL, 0.55 mmol). Final normal-phase purification (cyclohexane/EtOAc from 100/0 to 80/20) afforded pure title compound **33f** (149 mg, 92% yield). UPLC-MS: *R*_*t*_ = 1.91 min (method 2). MS (ESI) *m*/*z*: 323.3 [M – H]^−^, C_17_H_17_ClN_4_ [M – H]^−^ calcd, 323.8. ^1^H NMR (400 MHz, CDCl_3_): δ 8.03–7.96 (m, 2H), 7.95–7.92 (m, 2H), 7.47 (m, 3H), 7.22–7.10 (m, 3H), 7.09 (s, 1H), 2.68 (s, 6H). ^13^C NMR (101 MHz, CDCl_3_): δ 166.0 (Cq), 162.2 (Cq), 161.3 (Cq), 144.9 (Cq), 136.4 (Cq), 132.8 (Cq), 131.0 (CH), 128.9 (CH, 2C), 127.3 (CH, 2C), 124.6 (CH), 124.5 (CH), 120.5 (CH), 120.3 (CH), 99.1 (CH), 44.5 (CH_3_, 2C).

##### 2-Chloro-*N*-(3,4-dimethoxyphenyl)-6-phenylpyrimidin-4-amine (**33g**)

Title compound **33g** was prepared according to general procedure 1 using intermediate **31** (107 mg, 0.47 mmol) and 3,4-dimethoxyaniline **32g** (64 mg, 0.41 mmol). Final normal-phase purification (cyclohexane/EtOAc from 100/0 to 70/30) afforded pure title compound **33f** (149 mg, 92% yield). UPLC-MS: *R*_*t*_ = 1.54 min (method 2). MS (ESI) *m*/*z*: 340.2 [M – H]^−^, C_17_H_16_ClN_3_O_2_ [M – H]^−^ calcd, 340.8. ^1^H NMR (400 MHz, CDCl_3_): δ 7.97–7.83 (m, 2H), 7.49–7.38 (m, 3H), 7.07 (s, 1H), 6.93–6.88 (m, 3H), 6.81 (s, 1H), 3.93 (s, 3H), 3.89 (s, 3H).

##### 2-Chloro-*N*-(3-(2-methoxyethoxy)phenyl)-6-phenylpyrimidin-4-amine (**33h**)

Title compound **33h** was prepared according to general procedure 1 using intermediate **31** (85 mg, 0.38 mmol) and 3,4-dimethoxyaniline **32h** (68 mg, 0.41 mmol). Final normal-phase purification (cyclohexane/EtOAc from 100/0 to 65/35) afforded pure title compound **33h** (108 mg, 80% yield). UPLC-MS: *R*_*t*_ = 1.36 min (method 2). MS (ESI) *m*/*z*: 356.2 [M + H]^+^, C_19_H_19_ClN_3_O_2_ [M + H]^+^ calcd, 356.8. ^1^H NMR (400 MHz, CDCl_3_): δ 7.97–7.88 (m, 2H), 7.50–7.40 (m, 3H), 7.32 (t, *J* = 8.1 Hz, 1H), 7.15 (s, 1H), 7.00 (s, 1H), 6.97 (t, *J* = 2.3 Hz, 1H), 6.93 (dd, *J* = 7.8, 1.7 Hz, 1H), 6.82 (ddd, *J* = 8.4, 2.5 Hz, 1H), 4.16–4.13 (m, 2H), 3.79–3.74 (m, 2H), 3.45 (s, 3H).

##### 2-Chloro-6-phenyl-*N*-(3-(trifluoromethyl)phenyl)pyrimidin-4-amine (**33i**)

Title compound **33i** was prepared according to general procedure 1 using intermediate **31** (180 mg, 0.80 mmol) and 3,4-dimethoxyaniline **32i** (0.11 mL, 0.88 mmol). Final normal-phase purification (cyclohexane/EtOAc from 100/0 to 90/10) afforded pure title compound **33i** (200 mg, 71% yield). UPLC-MS: *R*_*t*_ = 1.87 min (method 2). MS (ESI) *m*/*z*: 348.3 [M – H]^−^, C_17_H_10_ClF_3_N_3_ [M – H]^−^ calcd, 348.7. ^1^H NMR (400 MHz, CDCl_3_): δ 8.00–7.89 (m, 2H), 7.70–7.64 (m, 2H), 7.56 (s, 1H), 7.52–7.42 (m, 4H), 7.09 (s, 1H), 6.96 (s, 1H).

##### *tert*-Butyl 4-(4-((4-Chlorophenyl)amino)-6-phenylpyrimidin-2-yl)-3,6-dihydropyridine-1(2*H*)-carboxylate (**35aa**)

Title compound **35aa** was prepared according to general procedure 3 using intermediate **33a** (310 mg, 0.98 mmol) and boronic ester **34a** (368 mg, 1.19 mmol). Final normal-phase purification (cyclohexane/EtOAc from 100/0 to 85/15) afforded pure title compound **35aa** (380 mg, 83% yield). UPLC-MS: *R*_*t*_ = 2.50 min (method 2). MS (ESI) *m*/*z*: 461.6 [M – H]^−^, C_26_H_26_ClN_4_O_2_ [M – H]^−^ calcd, 462.0. ^1^H NMR (400 MHz, DMSO-*d*_6_): δ 9.77 (s, 1H), 8.15–8.03 (m, 2H), 7.80 (d, *J* = 8.5 Hz, 2H), 7.56–7.50 (m, 3H), 7.41 (d, *J* = 8.5 Hz, 2H), 7.20 (s, 1H), 7.10 (s, 1H), 4.13 (s, 2H), 3.57 (t, *J* = 5.0 Hz, 2H), 2.69 (s, 2H), 1.44 (s, 9H).

##### *tert*-Butyl 4-(4-((4-Methoxyphenyl)amino)-6-phenylpyrimidin-2-yl)-3,6-dihydropyridine-1(2*H*)-carboxylate (**35ba**)

Title compound **35ba** was prepared according to general procedure 3 using intermediate **33b** (80 mg, 0.26 mmol), boronic ester **34a** (98.1 mg, 0.31 mmol), Pd(dppf)Cl_2_·DCM (42 mg, 0.05 mmol), and K_2_CO_3_ (2 M)_aq_ (0.39 mL, 0.78 mmol) in 1,4-dioxane dry (2.6 mL). Final normal-phase purification (cyclohexane/EtOAc from 95/5 to 75/25) afforded pure title compound **35ba** (104.7 mg, 89% yield). UPLC-MS: *R*_*t*_ = 2.24 min (method 2). MS (ESI) *m*/*z*: 459.3 [M + H]^+^, C_27_H_31_N_4_O_3_ [M + H]^+^ calcd, 459.6. ^1^H NMR (400 MHz, DMSO-*d*_6_): δ 9.44 (s, 1H), 8.12–8.02 (m, 2H), 7.68–7.60 (m, 2H), 7.58–7.46 (m, 3H), 7.17 (s, 1H), 7.01 (s, 1H), 6.99–6.92 (m, 2H), 4.12 (s, 2H), 3.75 (s, 3H), 3.56 (t, *J* = 5.7 Hz, 2H), 2.68 (q, *J* = 4.0, 3.4 Hz, 2H), 1.44 (s, 9H).

##### *tert*-Butyl 4-(4-((4-(Dimethylamino)phenyl)amino)-6-phenylpyrimidin-2-yl)-3,6-dihydropyridine-1(2*H*)-carboxylate (**35ca**)

Title compound **35ca** was prepared according to general procedure 3 using intermediate **33c** (100 mg, 0.35 mmol), boronic ester **34a** (117.8 mg, 0.37 mmol), Pd(dppf)Cl_2_·DCM (57 mg, 0.07 mmol), and K_2_CO_3_ (2 M)_aq_ (0.52 mL, 1.05 mmol) in 1,4-dioxane dry (3.5 mL). Final normal-phase purification (cyclohexane/EtOAc from 95/5 to 75/25) afforded pure title compound **35ca** (107.4 mg, 65% yield). UPLC-MS: *R*_*t*_ = 2.58 min (method 2). MS (ESI) *m*/*z*: 472.3 [M + H]^+^, C_28_H_34_N_5_O_2_ [M + H]^+^ calcd, 472.6. ^1^H NMR (400 MHz, DMSO-*d*_6_): δ 9.28 (s, 1H), 8.12–7.96 (m, 2H), 7.64–7.42 (m, 5H), 7.15 (s, 1H), 6.96 (s, 1H), 6.86–6.69 (m, 2H), 4.11 (d, *J* = 4.0 Hz, 2H), 3.55 (t, *J* = 5.7 Hz, 2H), 2.87 (s, 6H), 2.67 (d, *J* = 7.0 Hz, 2H), 1.44 (s, 9H).

##### *tert*-Butyl 4-(4-((3-(Dimethylamino)phenyl)amino)-6-phenylpyrimidin-2-yl)-3,6-dihydropyridine-1(2*H*)-carboxylate (**35da**)

Title compound **35da** was prepared according to general procedure 3 using intermediate **33d** (120 mg, 0.37 mmol), boronic ester **34a** (129.5 mg, 0.41 mmol), Pd(dppf)Cl_2_·DCM (60 mg, 0.07 mmol), and K_2_CO_3_ (2 M)_aq_ (0.55 mL, 1.11 mmol) in 1,4-dioxane dry (3.7 mL). Final normal-phase purification (cyclohexane/EtOAc from 100/0 to 80/20) afforded pure title compound **35da** (155.1 mg, 89% yield). UPLC-MS: *R*_*t*_ = 2.58 min (method 2). MS (ESI) *m*/*z*: 472.3 [M + H]^+^, C_28_H_34_N_5_O_2_ [M + H]^+^ calcd, 472.6. ^1^H NMR (400 MHz, DMSO-*d*_6_): δ 9.44 (s, 1H), 8.17–7.95 (m, 2H), 7.67–7.41 (m, 3H), 7.31 (s, 1H), 7.21 (s, 1H), 7.14 (t, *J* = 8.1 Hz, 1H), 7.11 (s, 1H), 7.03–6.93 (m, 1H), 6.42 (ddd, *J* = 8.3, 2.7, 0.8 Hz, 1H), 4.11 (s, 2H), 3.56 (t, *J* = 5.6 Hz, 2H), 2.93 (s, 6H), 2.81–2.62 (m, 2H), 1.44 (s, 9H).

##### *N*1-(2-(Cyclohex-1-en-1-yl)-6-phenylpyrimidin-4-yl)-*N*3,*N*3-dimethylbenzene-1,3-diamine (**35db**)

Title compound **35db** was prepared according to general procedure 3 using intermediate **33d** (70 mg, 0.22 mmol), boronic ester **34b** (67 mg, 0.32 mmol), Pd(dppf)Cl_2_·DCM (36 mg, 0.04 mmol), and K_2_CO_3_ (2 M)_aq_ (0.33 mL, 0.66 mmol) in 1,4-dioxane dry (2.2 mL). Final normal-phase purification (cyclohexane/EtOAc from 100/0 to 90/10) afforded pure title compound **35db** (65 mg, 80% yield). UPLC-MS: *R*_*t*_ = 1.55 min (method 3). MS (ESI) *m*/*z*: 371.2 [M + H]^+^, C_24_H_27_N_4_ [M + H]^+^ calcd, 370.5. ^1^H NMR (400 MHz, CDCl3): δ 8.07–8.00 (m, 2H), 7.51–7.42 (m, 3H), 7.35 (s, 1H), 7.24 (d, *J* = 8.0 Hz, 1H), 7.02 (d, *J* = 1.1 Hz, 1H), 6.84 (s, 1H), 6.70 (dd, *J* = 7.7, 2.0 Hz, 1H), 6.56 (d, *J* = 8.3 Hz, 1H), 2.99 (s, 6H), 2.71–2.67 (m, 2H), 2.36–2.32 (m, 2H), 1.87–1.75 (m, 2H), 1.75–1.66 (m, 2H).

##### *tert*-Butyl 4-(4-((2-Methoxyphenyl)amino)-6-phenylpyrimidin-2-yl)-3,6-dihydropyridine-1(2*H*)-carboxylate (**35ea**)

Title compound **35ea** was prepared according to general procedure 3 using intermediate **33e** (190 mg, 0.57 mmol), boronic ester **34a** (211 mg, 0.68 mmol), Pd(dppf)Cl_2_·DCM (93 mg, 0.11 mmol), and K_2_CO_3_ (2 M)_aq_ (0.86 mL, 1.7 mmol) in 1,4-dioxane dry (5.7 mL). Final normal-phase purification (cyclohexane/EtOAc from 100/0 to 85/15) afforded pure title compound **35ea** (230 mg, 88% yield). UPLC-MS: *R*_*t*_ = 2.47 min (method 2). MS (ESI) *m*/*z*: 459.3 [M + H]^+^, C_27_H_31_N_4_O_3_ [M + H]^+^ calcd, 458.6. ^1^H NMR (400 MHz, CDCl_3_): δ 8.26 (dd, *J* = 7.0, 2.5 Hz, 1H), 8.10–8.00 (m, 2H), 7.48–7.44 (m, 3H), 7.24 (bs, 2H), 7.07–7.03 (m, 2H), 6.96–6.93 (m, 2H), 4.21 (q, *J* = 3.0 Hz, 2H), 3.91 (s, 3H), 3.67 (t, *J* = 5.8 Hz, 2H), 2.84 (bs, 2H), 1.51 (s, 9H).

##### *tert*-Butyl 4-(4-((2-(Dimethylamino)phenyl)amino)-6-phenylpyrimidin-2-yl)-3,6-dihydropyridine-1(2*H*)-carboxylate (**35fa**)

Title compound **35fa** was prepared according to general procedure 3 using intermediate **33f** (150 mg, 0.46 mmol), boronic ester **34a** (171 mg, 0.55 mmol), Pd(dppf)Cl_2_·DCM (75 mg, 0.09 mmol), and K_2_CO_3_ (2 M)_aq_ (0.69 mL, 1.38 mmol) in 1,4-dioxane dry (4.6 mL). Final normal-phase purification (cyclohexane/EtOAc from 100/0 to 95/5) afforded pure title compound **35fa** (134.7 mg, 62% yield). UPLC-MS: *R*_*t*_ = 1.54 min (method 3). MS (ESI) *m*/*z*: 472.3 [M + H]^+^, C_28_H_34_N_5_O_2_ [M + H]^+^ calcd, 472.6. ^1^H NMR (400 MHz, CDCl_3_): δ 8.28 (d, *J* = 8.0 Hz, 1H), 8.14–8.04 (m, 2H), 7.85 (s, 1H), 7.50–7.43 (m, 3H), 7.20 (dd, *J* = 7.8, 1.5 Hz, 1H), 7.16 (dd, *J* = 7.8, 1.6 Hz, 1H), 7.06 (td, *J* = 7.7, 1.5 Hz, 1H), 7.00 (s, 1H), 4.22 (q, *J* = 3.0 Hz, 2H), 3.68 (t, *J* = 5.7 Hz, 2H), 2.86 (bs, 2H), 2.70 (s, 6H), 1.51 (s, 9H).

##### *tert*-Butyl 4-(4-((3,4-Dimethoxyphenyl)amino)-6-phenylpyrimidin-2-yl)-3,6-dihydropyridine-1(2*H*)-carboxylate (**35ga**)

Title compound **35ga** was prepared according to general procedure 3 using intermediate **33g** (100 mg, 0.29 mmol), boronic ester **34a** (108 mg, 0.35 mmol), Pd(dppf)Cl_2_·DCM (47 mg, 0.06 mmol), and K_2_CO_3_ (2 M)_aq_ (0.43 mL, 0.87 mmol) in 1,4-dioxane dry (2.9 mL). Final normal-phase purification (cyclohexane/EtOAc from 100/0 to 95/5) afforded pure title compound **35ga** (85 mg, 60% yield). UPLC-MS: *R*_*t*_ = 2.05 min (method 2). MS (ESI) *m*/*z*: 489.3 [M + H]^+^, C_28_H_33_N_4_O_4_ [M + H]^+^ calcd, 489.6. ^1^H NMR (400 MHz, CDCl_3_): δ 8.03–7.95 (m, 2H), 7.50–7.42 (m, 3H), 7.23 (s, 1H), 7.05 (s, 1H), 6.95–6.89 (m, 2H), 6.83 (bs, 1H), 4.18 (s, 2H), 3.91 (s, 3H), 3.87 (s, 3H), 3.64 (s, 2H), 2.80 (s, 2H), 1.49 (s, 9H).

##### *tert*-Butyl 4-(4-((3-(2-methoxyethoxy)phenyl)amino)-6-phenylpyrimidin-2-yl)-3,6-dihydropyridine-1(2*H*)-carboxylate (**35ha**)

Title compound **35ha** was prepared according to general procedure 3 using intermediate **33h** (70 mg, 0.20 mmol), boronic ester **34a** (74 mg, 0.24 mmol), Pd(dppf)Cl_2_·DCM (33 mg, 0.04 mmol), and K_2_CO_3_ (2 M)_aq_ (0.30 mL, 0.60 mmol) in 1,4-dioxane dry (2.0 mL). Final normal-phase purification (cyclohexane/EtOAc from 100/0 to 85/15) afforded pure title compound **35ha** (70 mg, 70% yield). UPLC-MS: *R*_*t*_ = 2.11 min (method 2). MS (ESI) *m*/*z*: 503.3 [M + H]^+^, C_29_H_35_N_4_O_4_ [M + H]^+^ calcd, 502.6. ^1^H NMR (400 MHz, CDCl_3_): δ 7.96–7.88 (m, 2H), 7.51–7.40 (m, 3H), 7.32 (td, *J* = 8.1, 1.1 Hz, 1H), 7.25 (bs, 1H), 7.19 (s, 1H), 7.00 (d, *J* = 0.9 Hz, 1H), 6.97 (t, *J* = 2.3 Hz, 1H), 6.93 (dd, *J* = 7.9, 1.5 Hz, 1H), 6.84–6.81 (m, 1H), 4.17–4.12 (m, 2H), 3.81–3.72 (m, 2H), 3.45 (s, 3H).

##### *tert*-Butyl 4-(4-Phenyl-6-((3-(trifluoromethyl)phenyl)amino)pyrimidin-2-yl)-3,6-dihydropyridine-1(2*H*)-carboxylate (**35ia**)

Title compound **35ia** was prepared according to general procedure 3 using intermediate **33i** (185 mg, 0.53 mmol), boronic ester **34a** (197 mg, 0.64 mmol), Pd(dppf)Cl_2_·DCM (86 mg, 0.10 mmol), and K_2_CO_3_ (2 M)_aq_ (0.79 mL, 1.59 mmol) in 1,4-dioxane dry (5.3 mL). Final normal-phase purification (cyclohexane/EtOAc from 100/0 to 85/15) afforded pure title compound **35ia** (220 mg, 84% yield). UPLC-MS: *R*_*t*_ = 2.59 min (method 2). MS (ESI) *m*/*z*: 495.4 [M + H]^+^, C_27_H_28_F_3_N_4_O_2_ [M + H]^+^ calcd, 496.5. ^1^H NMR (400 MHz, CDCl_3_): δ 8.09 (s, 1H), 8.07–7.99 (m, 2H), 7.62 (d, *J* = 8.1 Hz, 1H), 7.51–7.43 (m, 4H), 7.36 (d, *J* = 7.7 Hz, 1H), 7.26 (bs, 1H), 6.91 (s, 2H), 4.21 (q, *J* = 3.9 Hz, 2H), 3.67 (t, *J* = 5.7 Hz, 2H), 2.82 (b, 2H), 1.51 (s, 9H).

##### *tert*-Butyl 4-(4-((4-chlorophenyl)amino)-6-phenylpyrimidin-2-yl)piperidine-1-carboxylate (**36aa**)

Et_3_SiH (1.38 mL, 8.66 mmol) and Pd/C (200 mg) were added to a solution of compound **35aa** (200 mg, 0.43 mmol) in a 1:1:1 mixture of toluene/EtOAc/EtOH (6 mL) at 10 °C under argon. The reaction mixture was stirred for 30 min and filtered through a Celite pad. The residue was concentrated under vacuum and subjected to the first trituration with a 1:1 mixture of DCM/EtOAc, the resulting solid was filtered, and the mother liquor was concentrated under vacuum. Final normal-phase purification (toluene/EtOAc from 100/0 to 85/15) afforded compound **36aa** (45 mg, 23% yield) impure of 3% of dechlorinated byproduct. UPLC-MS: *R*_*t*_ = 2.37 min (method 2). MS (ESI) *m*/*z*: 463.4 [M – H]^−^, C_26_H_28_ClN_4_O_2_ [M – H]^−^ calcd, 464.0. ^1^H NMR (400 MHz, CDCl_3_): δ 1H NMR (400 MHz, CDCl_3_): δ 8.01–7.92 (m, 2H), 7.49–7.43 (m, 3H), 7.43–7.39 (m, 2H), 7.37–7.32 (m, 2H), 6.88 (s, 2H), 4.22 (bs, 2H), 2.95 (tt, *J* = 11.5, 3.7 Hz, 1H), 2.93–2.88 (m, 2H), 2.06 (d, *J* = 11.2 Hz, 2H), 1.89 (qd, *J* = 12.2, 4.3 Hz, 2H), 1.49 (s, 9H).

##### *tert*-Butyl 4-(4-((4-Methoxyphenyl)amino)-6-phenylpyrimidin-2-yl)piperidine-1-carboxylate (**36ba**)

Title compound **36ba** was prepared according to general procedure 4 using intermediate **35ba** (120 mg), NH_4_CO_2_H (65 mg, 1 mmol), and Pd(OH)_2_/C (46 mg) in MeOH dry (6.2 mL). The crude was used as such without further purification. UPLC-MS: *R*_*t*_ = 1.94 min (method 2). MS (ESI) *m*/*z*: 461.2 [M + H]^+^, C_27_H_33_N_4_O_3_ [M + H]^+^ calcd, 461.6.

##### *tert*-Butyl 4-(4-((4-(Dimethylamino)phenyl)amino)-6-phenylpyrimidin-2-yl)piperidine-1-carboxylate (**36ca**)

Title compound **36ca** was prepared according to general procedure 4 using intermediate **35ca** (120 mg, 0.26 mmol), NH_4_CO_2_H (65 mg, 1 mmol), and Pd(OH)_2_/C (46 mg) in MeOH dry (6.5 mL). The crude was used as such without further purification. UPLC-MS: *R*_*t*_ = 2.23 min (method 2). MS (ESI) *m*/*z*: 472.6 [M – H]^−^, C_28_H_34_N_5_O_2_ [M – H]^−^ calcd, 472.3.

##### *tert*-Butyl 4-(4-((2-Methoxyphenyl)amino)-6-phenylpyrimidin-2-yl)piperidine-1-carboxylate (**36ea**)

Title compound **36ea** was prepared according to general procedure 4 using intermediate **35ea** (230 mg, 0.50 mmol), NH_4_CO_2_H (189 mg, 3.00 mmol), and Pd(OH)_2_/C (46 mg) in MeOH dry (8 mL). Final normal-phase purification (cyclohexane/EtOAc from 100/0 to 70/30) afforded pure title compound **36ea** (191 mg, 83% yield). UPLC-MS: *R*_*t*_ = 2.30 min (method 2). MS (ESI) *m*/*z*: 459.5 [M – H]^−^, C_27_H_31_N_4_O_3_ [M – H]^−^ calcd, 459.6. ^1^H NMR (400 MHz, CDCl_3_): δ 8.19 (dd, *J* = 7.6, 1.9 Hz, 1H), 8.04–7.96 (m, 2H), 7.50–7.40 (m, 3H), 7.21 (s, 1H), 7.08 (td, *J* = 7.5, 1.9 Hz, 1H), 7.03 (td, *J* = 7.5, 1.8 Hz, 1H), 6.97–6.91 (m, 2H), 4.23 (bs, 2H), 2.99 (tt, *J* = 11.5, 3.8 Hz, 1H), 2.96–2.86 (m, 2H), 2.09 (d, *J* = 13.1 Hz, 2H), 1.89 (qd, *J* = 12.1, 4.0 Hz, 2H), 1.49 (s, 9H).

##### *tert*-Butyl 4-(4-((2-(Dimethylamino)phenyl)amino)-6-phenylpyrimidin-2-yl)piperidine-1-carboxylate (**36fa**)

Title compound **36fa** was prepared according to general procedure 4 using intermediate **35fa** (135 mg, 0.29 mmol), NH_4_CO_2_H (110 mg, 1.74 mmol), and Pd(OH)_2_/C (27 mg) in MeOH dry (4.8 mL). Final normal-phase purification (cyclohexane/EtOAc from 100/0 to 85/15) afforded pure title compound **36fa** (110 mg, 80% yield). UPLC-MS: *R*_*t*_ = 1.35 min (method 3). MS (ESI) *m*/*z*: 472.4 [M – H]^−^, C_28_H_34_N_5_O_2_ [M – H]^−^ calcd, 472.6. ^1^H NMR (400 MHz, CDCl_3_): δ 8.22 (dd, *J* = 8.0, 1.5 Hz, 1H), 8.07–7.99 (m, 2H), 7.83 (s, 1H), 7.48–7.43 (m, 3H), 7.19 (dd, *J* = 7.8, 1.5 Hz, 1H), 7.16 (td, *J* = 7.8, 1.6 Hz, 1H), 7.05 (td, *J* = 7.6, 1.5 Hz, 1H), 6.99 (s, 1H), 4.24 (bs, 2H), 2.99 (tt, *J* = 11.5, 3.8 Hz, 1H), 2.95–2.85 (m, 2H), 2.69 (s, 6H), 2.10 (d, *J* = 13.0 Hz, 2H), 1.95 (qd, *J* = 12.3, 4.3 Hz, 2H), 1.50 (s, 9H).

##### *tert*-Butyl 4-(6-((3,4-Dimethoxyphenyl)amino)-4-phenyl-4,5-dihydropyrimidin-2-yl)piperidine-1-carboxylate (**36ga**)

Title compound **36ga** was prepared according to general procedure 4 using intermediate **35ga** (80 mg, 0.16 mmol), NH_4_CO_2_H (61 mg, 0.96 mmol), and Pd(OH)_2_/C (20 mg) in MeOH dry (2.7 mL). Final normal-phase purification (cyclohexane/EtOAc from 100/0 to 45/55) afforded pure title compound **36ga** (41 mg, 52% yield). UPLC-MS: *R*_*t*_ = 1.88 min (method 2). MS (ESI) *m*/*z*: 491.4 [M + H]^+^, C_28_H_37_N_4_O_4_ [M + H]^+^ calcd, 491.6. NMR (400 MHz, CDCl_3_): δ 7.95–7.93 (dd, *J* = 6.7, 3.0 Hz, 2H), 7.44–7.43 (m, 3H), 7.03 (s, 1H), 6.90 (bs, 3H), 6.80 (s, 1H), 4.22 (bs, 2H), 3.92 (s, 3H), 3.89 (s, 3H), 2.98–2.83 (m, 3H), 2.05 (d, *J* = 12.7 Hz, 2H), 1.91 (qd, *J* = 12.2, 4.2 Hz, 2H), 1.48 (s, 9H).

##### *tert*-Butyl 4-(4-((3-(2-Methoxyethoxy)phenyl)amino)-6-phenylpyrimidin-2-yl)piperidine-1-carboxylate (**36ha**)

Title compound **36ha** was prepared according to general procedure 4 using intermediate **35ha** (100 mg, 0.20 mmol), NH_4_CO_2_H (76 mg, 1.20 mmol), and Pd(OH)_2_/C (20 mg) in MeOH dry (3.3 mL). Final normal-phase purification (cyclohexane/EtOAc from 100/0 to 70/30) afforded pure title compound **36ha** (65 mg, 64% yield). TLC: *R*_*f*_ = 0.3 (75/25 cyclohexane/EtOAc). ^1^H NMR (400 MHz, CDCl_3_): δ 1H NMR (400 MHz, CDCl_3_): δ 7.99–7.95 (m, 2H), 7.49–7.42 (m, 3H), 7.29 (td, *J* = 8.2, 2.0 Hz, 1H), 7.08–7.06 (m, 1H), 7.05–6.93 (m, 3H), 6.80–6.71 (m, 1H), 4.31–4.18 (m, 2H), 4.17–4.12 (m, 2H), 3.78–3.75 (m, 2H), 3.45 (s, 3H), 2.98–2.93 (m, 3H), 2.06 (d, *J* = 12.6 Hz, 2H), 1.90 (qd, *J* = 12.3, 4.3 Hz, 3H), 1.48 (s, 9H).

##### *tert*-Butyl 4-(4-Phenyl-6-((3-(trifluoromethyl)phenyl)amino)pyrimidin-2-yl)piperidine-1-carboxylate (**36ia**)

Title compound **36ia** was prepared according to general procedure 4 using intermediate **35ia** (220 mg, 0.44 mmol), NH_4_CO_2_H (167 mg, 2.64 mmol), and Pd(OH)_2_/C (44 mg) in MeOH dry (7.3 mL). Final normal-phase purification (cyclohexane/EtOAc from 100/0 to 75/25) afforded pure title compound **36ia** (189 mg, 86% yield). UPLC-MS: *R*_*t*_ = 2.44 min (method 2). MS (ESI) *m*/*z*: 497.4 [M – H]^−^, C_27_H_28_F_3_N_4_O_2_ [M – H]^−^ calcd, 497.5. ^1^H NMR (400 MHz, CDCl_3_): δ 8.03–7.93 (m, 3H), 7.65 (d, *J* = 8.2 Hz, 1H), 7.53–7.41 (m, 4H), 7.37 (d, *J* = 7.7 Hz, 1H), 6.91 (s, 2H), 4.18 (bs, 2H), 2.99 (tt, *J* = 11.5, 3.8 Hz, 1H), 2.96–2.91 (m, 2H), 2.10 (d, *J* = 13.0 Hz, 2H), 1.90 (qd, *J* = 11.8, 4.3 Hz, 2H), 1.49 (s, 9H).

##### 2,4-Dichloro-6-phenyl-1,3,5-triazine (**38**)

Phenyl boronic acid (44 mg, 0.36 mmol), Pd(PPh_3_)_2_Cl_2_ (12.7 mg, 0.02 mmol), and K_2_CO_3_ (200 mg, 1.44 mmol) were added to a solution of cyanuric chloride **37** (100 mg, 0.54 mmol) in toluene (1.8 mL) under argon. The reaction mixture stirred at 70 °C for 2 h. After that, the reaction mixture was diluted with EtOAc (2 mL) and water (3 mL), and the organic layer was divided, dried over Na_2_SO_4_, filtered, and concentrated under vacuum. Purification by silica (cyclohexane/EtOAc from 100 to 98/2) afforded pure compound **38** as a white solid (42 mg, 51% yield). UPLC-MS: *R*_*t*_ = 1.48 min (method 2), no ionization. ^1^H NMR (400 MHz, CDCl_3_): δ 8.55–8.46 (m, 2H), 7.66 (tt, *J* = 7.4, 1.4 Hz, 1H), 7.56–7.51 (m, 2H).

##### (1-(*tert*-Butoxycarbonyl)-1,2,3,6-tetrahydropyridin-4-yl)boronic Acid (**39**)

Boronic ester **34a** (155 mg, 0.50 mmol), NaIO_4_ (385 mg, 1.80 mmol), and NH_4_OAc (139 mg, 1.8 mmol) were suspended in an acetone/water 1:1 mixture (5 mL). The reaction mixture was stirred for 24 h at room temperature. The resulting precipitate was filtered, and acetone was concentrated under vacuum. The aqueous solution was extracted with Et_2_O (4 × 3 mL). Collected organic layers were dried over Na_2_SO_4_, filtered, and concentrated under vacuum. The resulting crude was used as such without further purification. UPLC-MS: *R*_*t*_ = 1.62 min (method 2). MS (ESI) *m*/*z*: 172.0 [M + H]^+^, C_10_H_19_BNO_4_ [M + H]^+^ calcd, 172.1. ^1^H NMR (400 MHz, CDCl_3_): δ 6.43 (bs, 1H), 4.09–3.89 (m, 2H), 3.46 (q, *J* = 6.2 Hz, 2H), 2.34–2.26 (n, 2H), 1.47 (s, 9H). ^1^H NMR analysis taken at different times (0, 18 h) showed an equilibrium between two species.

##### *tert*-Butyl 4-(4-Chloro-6-phenyl-1,3,5-triazin-2-yl)-3,6-dihydropyridine-1(2*H*)-carboxylate (**40**)

K_2_CO_3_ (2 M)_aq_ (1.1 mL, 2.21 mmol), PdCl_2_(PPh_3_)_2_ (15.5 mg, 0.02 mmol), and boronic acid **39** (250 mg, 1.11 mmol) were added to a degassed solution of compound **38** (250 mg, 1.11 mmol) in toluene dry (5 mL) under argon. The reaction mixture was stirred at 80 °C for 2 h. After that, water (3 mL), NaCl sat sol (2 mL), and DCM (5 mL) were added, and the collected organic layer was dried over Na_2_SO_4_, filtered, and concentrated under vacuum. Final normal-phase purification (cyclohexane/EtOAc from 100/0 to 90/10) afforded pure title compound **40** (295 mg, 71% yield). ^1^H NMR (400 MHz, CDCl_3_): δ 8.57–8.46 (m, 2H), 7.62 (bs, 1H), 7.61 (tt, *J* = 7.3, 1.4 Hz, 1H), 7.53–7.49 (m, 2H), 4.33–4.17 (m, 2H), 3.65 (q, *J* = 5.7 Hz, 2H), 2.76 (bs, 2H), 1.50 (s, 9H).

##### *tert*-Butyl 4-(4-((3-(Dimethylamino)phenyl)amino)-6-phenyl-1,3,5-triazin-2-yl)-3,6-dihydropyridine-1(2*H*)-carboxylate (**41a**) and *tert*-Butyl 4-(4-((3-(Dimethylamino)phenyl)amino)-6-phenyl-1,3,5-triazin-2-yl)-3,4-dihydropyridine-1(2*H*)-carboxylate (**41b**)

Aniline **32d** (84 mg, 0.40 mmol) and DIPEA (0.38 mL, 2.15 mmol) were added to a solution of compound **40** (100 mg, 0.27 mmol) under argon. The reaction mixture was stirred for 3 h at 80 °C until complete consumption of the starting material. After that, the crude was concentrated under vacuum, the residue was risen with water (2 mL) and DCM (3 mL), and the organic layer was collected through a phase separator and concentrated under vacuum. Final normal-phase purification (cyclohexane/EtOAc from 100/0 to 70/30) afforded a mixture of **41a** and **41b** (with isomerized double C=C bond) in a ratio of 1:0.4 (120 mg, 94% combined yield). ^1^H NMR of major isomer **41a** (400 MHz, CDCl_3_): δ 8.51 (d, *J* = 7.7 Hz, 2H), 7.58–7.43 (m, 4H), 7.34 (s, 1H), 7.31 (s, 1H), 7.23 (d, *J* = 8.1 Hz, 1H), 6.94 (dd, *J* = 7.9, 2.0 Hz, 1H), 6.52 (dd, *J* = 8.3, 2.5 Hz, 1H), 4.22 (q, *J* = 3.8 Hz, 2H), 3.65 (t, *J* = 5.7 Hz, 2H), 3.01 (s, 6H), 2.77 (bs, 2H), 1.51 (s, 9H). ^1^H NMR of minor isomer **41b** (400 MHz, CDCl_3_): δ 8.49 (d, *J* = 7.7 Hz, 2H), 7.57–7.52 (m, 1H), 7.52–7.45 (m, 2H), 7.39 (bs, 1H), 7.31 (bs, 1H), 7.23 (t, *J* = 8.1 Hz, 1H), 7.07 (d, *J* = 8.4 Hz, 1H), 6.94 (d, *J* = 8.3 Hz, 1H), 6.88 (d, *J* = 7.8 Hz, 1H), 6.52 (dd, *J* = 8.3, 2.5 Hz, 1H), 5.26–5.16 (m, 1H), 3.92–3.84 (m, 1H), 3.64 (bs, 2H), 3.01 (s, 6H), 2.36–2.27 (m, 2H), 1.52 (s, 9H).

##### 4,6-Dichloro-*N*-(3-(trifluoromethyl)phenyl)-1,3,5-triazin-2-amine (**42**)

Aniline **32i** (0.07 mL, 0.55 mmol) and DIPEA (0.11 mL, 0.61 mmol) were added to a solution of cyanuric chloride **37** (100 mg, 0.55 mmol) in DCM dry (2 mL) at 0 °C under argon. After 1 h, water (2 mL) and DCM (2 mL) were added, and the organic layer was collected through a phase separator and concentrated under vacuum. Final normal-phase purification (cyclohexane/EtOAc from 100/0 to 80/20) afforded compound **42** (41 mg, 24% yield). UPLC-MS: *R*_*t*_ = 1.25 min (method 2). MS (ESI) *m*/*z*: 307.0/309.0 [M – H]^−^, C_10_H_4_Cl_2_F_3_N_4_ [M – H]^−^ calculated, 308.1. ^1^H NMR (400 MHz, CDCl_3_): δ 7.84 (s, 1H), 7.78 (d, *J* = 8.0, 1H), 7.68 (bs, 1H) 7.55 (t, *J* = 7.9 Hz, 1H), 7.48 (d, *J* = 7.8 Hz, 1H).

##### *tert*-Butyl 4-(4-Chloro-6-((3-(trifluoromethyl)phenyl)amino)-1,3,5-triazin-2-yl)-3,6-dihydropyridine-1(2*H*)-carboxylate (**43**)

K_2_CO_3_ (2 M)_aq_ (0.16 mL, 0.32 mmol) was added to a degassed mixture of compound **42** (50 mg, 0.16 mmol), boronic acid **39** (44 mg, 0.19 mmol), and PdCl_2_(PPh_3_)_2_ (6 mg, 0.008 mmol) in toluene dry (1 mL) under argon. The reaction mixture was stirred at 90 °C for 2 h. After that, water (2 mL) was added, and the aqueous layer was extracted with EtOAc (3 × 2 mL). Collected organic layers were dried over Na_2_SO_4_, filtered, and concentrated under vacuum. Final normal-phase purification (cyclohexane/EtOAc from 100/0 to 90/10) afforded compound **43** (40 mg, 55% yield). *R*_*f*_ = 0.40 (8/2 cyclohexane/EtOAc). ^1^H NMR (400 MHz, CDCl_3_): δ 8.17 (bs, 1H), 7.63 (bs, 1H), 7.55–7.31 (m, 4H), 4.27–4.16 (m, 2H), 3.61 (t, *J* = 5.7 Hz, 2H), 2.63 (bs, 2H), 1.49 (s, 9H).

##### *tert*-Butyl 4-(4-Phenyl-6-((3-(trifluoromethyl)phenyl)amino)-1,3,5-triazin-2-yl)-3,6-dihydropyridine-1(2*H*)-carboxylate (**44**)

K_2_CO_3_ (2 M)_aq_ (0.60 mL, 0.12 mmol) was added to a degassed mixture of compound **43** (36 mg, 0.08 mmol), phenylboronic acid (12 mg, 0.10 mmol), and PdCl_2_(PPh_3_)_2_ (3 mg, 0.004 mmol) in toluene dry (0.4 mL) under argon. The reaction mixture was stirred at 120 °C for 2 h. After that, water (2 mL) was added, and the aqueous layer was extracted with EtOAc (3 × 2 mL). Collected organic layers were dried over Na_2_SO_4_, filtered, and concentrated under vacuum. Final normal-phase purification (cyclohexane/EtOAc from 100/0 to 90/10) afforded compound **44** (15 mg, 38% yield). *R*_f_ = 0.50 (75/25 cyclohexane/EtOAc). ^1^H NMR (400 MHz, CDCl_3_): δ 8.54–8.46 (m, 2H), 8.35 (s, 1H), 7.71 (d, *J* = 8.1 Hz, 1H), 7.63 (s, 1H), 7.60–7.54 (m, 1H), 7.54–7.44 (m, 4H), 7.38 (d, *J* = 7.9 Hz, 1H), 4.24 (q, *J* = 3.6 Hz, 2H), 3.66 (t, *J* = 5.8 Hz, 2H), 2.76 (bs, 2H), 1.51 (s, 9H).

## *In Vitro* and *In Vivo* Experiments

### Cell Viability

Cell viability assay was performed as previously described.^12^ Cancer cell lines were cultured according to the method described in Jahid et al., 2022.^12^

### *In Vivo* Efficacy

*In vivo* efficacy test was performed as described previously with a slight modification.^12^ After tumors reached an initial size range of 150–250 mm^3^, mice were administrated 10 mg/kg ARN25062, ARN24928, or vehicle via tail vein injection for 1–2 weeks daily. Tumors were measured every other day with a caliper, and tumor volume was calculated ((length × width × width)/2). At the end of 2 weeks, tumors were extracted and measured to determine volume (length × width × height). GraphPad Prism9 software was used to generate line and bar graphs and determine two-way ANOVA and two-tailed *T*-test. Equal numbers of males and females were used in the experiments.

## Computational Methods

### Molecular Docking

In order to predict and evaluate the interaction between CDC42 and ARN24928/ARN25062 compounds, we first performed molecular docking. As a receptor structure, we employed the CDC42 protein in complex with the CRIB domain of PAK6 (PDB code 2ODB, resolution of 2.4 Å) where we formerly identified a previously unappreciated allosteric pocket at the protein–protein interface.^12^ The structure was refined by using the Protein Preparation Wizard^30^ workflow implemented in Maestro Release 2021-3. Specifically, hydrogen atoms were added, and charges and protonation states were assigned, titrating the protein at physiologic pH. The steric clashes were relieved by performing a small number of minimization steps, until the rmsd of the non-hydrogen atoms reached 0.30 Å. The formerly identified pocket was used to center the grid. Precisely, the cubic grid box of 26 × 26 × 26 Å^3^ was centered on the previously identified hit compound of the CDC42-ligand complex.^12^ The compound was prepared using LigPrep software implemented in Maestro. First, we added hydrogens and generated ionization states at pH 7.4 ± 0.5. Then, we generated tautomers and all stereochemical isomers. Finally, we used Glide^31−33^ to perform the molecular docking, using Extra Precision and retaining maximum 20 poses. The best docking pose was chosen for further investigation through classical MD simulation.

### MD Simulations

MD simulations were performed on the protein–ligand complexes obtained from our docking calculations. The GTP substrate as well as the catalytic Mg^2+^ ion in the active site of the proteins were considered. The systems were hydrated with a 14 Å layer of TIP3P water molecules^34^ from the protein center. The coordinates of the water molecules at the catalytic center were taken from the PDB X-ray structure 2ODB. Sodium ions were added to neutralize the charge of the systems. The final models are enclosed in a box of ∼89 × 89 × 89 Å^3^, containing ∼18,800 water molecules, resulting in ∼59,000 atoms for each system. The AMBER-ff14SB force field^35^ was used for the parametrization of the protein. The parameters for the ligands ARN24928 and ARN25062 were determined via Hartree–Fock calculation, with the 6-31G* basis set, convergence criterium SCF = tight after structure optimization (DFT B3LYP functional; 6-31G* basis set). The Merz-Singh-Kollman scheme^36^ was used for the atomic charge assignment. The GTP and the Mg^2+^ were parametrized according to Meagher et al. and Allner et al. respectively.^37,38^ Joung–Chetham parameters were used for monovalent ions.^39^ All MD simulations were performed with Amber,^40^ and all the systems were the object of the following equilibration protocol. To relax the water molecule and the ions, we performed an energy minimization, imposing a harmonic potential of 300 kcal/mol Å^2^ on the backbone, the GTP, and the docked compounds. Then, two consecutive MD simulations in *NVT* and *NPT* ensembles (1 and 10 ns, respectively) were carried out, imposing the previous positional restraints. To relax the solute, two additional energy minimization steps were performed, imposing positional restraints of 20 kcal/mol Å^2^ and without any restraints, respectively. Such minimized systems were heated up to 303 K with four consecutive MD simulations in *NVT* (∼0.1 ns, 100 K) and *NPT* ensembles (∼0.1 ns, 100 K; ∼0.1 ns, 200 K; and ∼0.2 ns, 303 K), imposing the previous positional restraints of 20 kcal/mol Å^2^. We used the Andersen-like temperature-coupling scheme,^41^ while pressure control was achieved with the Monte Carlo barostat at a reference pressure of 1 atm. Long-range electrostatics were treated with the particle mesh Ewald method. We performed an additional MD simulation (∼1.5 ns) in the *NPT* ensemble at 303 K without any restraint to relax the system at such temperature. Finally, multiple replicas of 500 ns were performed in the *NPT* ensemble for each system with an integration time step of 2 fs.

## Biophysical Methods

### His-CDC42 Production and Purification

The His-CDC42 wild type (amino acids Ile4-Pro182) was expressed from the pET28a+ vector in *Escherichia coli* BL21 (DE3) cells and purified, GppNHp or GDP-bound, as previously described.^12^

### Binding Check of Hit Derivatives by Microscale Thermophoresis

MicroScale Thermophoresis experiments were performed according to the NanoTemper Technologies protocols in a Monolith NT.115 Pico (Pico Red/Nano Blue—NanoTemper Technologies). His-CDC42 was RED–NHS-labeled and used at a concentration of 10 nM. Compound concentration was 50 μM throughout all the experiments. DMSO was also constant across samples at 0.5% v/v. Solutions were prepared in 100 mM Trizma base (Sigma) pH 7.5, 40 mM NaCl, 0.05% v/v Tween 20 or PBS, 0.05% v/v Tween 20 and incubated 5 min before loading on Premium Capillaries and analysis. Binding was detected at 24 °C, MST power high, and 20% light-emitting diode power. The MST traces were recorded as follows: 3 s MST power off, 20 s MST power on, and 1 s MST power off. The difference in normalized fluorescence (Δ*F*_norm_ [‰] = *F*_hot_/*F*_cold_) between the protein/compound sample and a protein-only sample at 1.5–2.5 s is calculated and plotted through MO.Affinity analysis v2.3 (NanoTemper Technologies) and GraphPad Prism 8.0.0 (GraphPad Software, San Diego, California USA). The signal-to-noise ratio and response amplitude were used to evaluate the quality of the binding data according to NanoPedia instructions (NanoTemper Technologies). Only a signal-to-noise ratio of more than 5 and a response amplitude of more than 1.5 were considered acceptable, while a signal-to-noise of more than 12 was considered excellent.

### NMR Confirmation of Target Engagement

All the NMR experiments were recorded at 25 °C using a NMR Bruker 600 MHz NEO equipped with a 5 mm CryoProbe QCI ^1^H/^19^F−^13^C/^15^N−D quadruple resonance, shielded z-gradient coil, and an automatic sample changer SampleJet system with temperature control. For all samples, a 1D ^1^H NMR experiment was recorded, and the water suppression was obtained using the standard NOESY (nuclear Overhauser effect spectroscopy) presat Bruker pulse sequence, with 64 k data points, a spectral width (sw) of 30 ppm, 64 scans, acquisition time (aq) of 1.835 s, a relaxation delay (d1) of 4 s, and a mixing time of 10 ms. ^1^H T_2_ filter experiments were recorded using the CPMG^42^ spin-echo train sequence with a total echo time to 750 ms consisting of 150 repetitions with a τ time of 5 m and a 180° pulse of approximately 27 μs, 128 scans, d1 4 s, sw 30 ppm, aq 1.835 s. The WaterLOGSY experiments were achieved with a 7.5 ms-long 180° Gaussian-shaped pulse, aq 0.852 s, mixing time of 1.7 s, relaxation delay of 2 s, 512 scans. ^19^F T_2_ filter experiments were recorded using the CPMG spin-echo scheme with a 50 ms time interval between the 180 pulses and different total lengths (200 and 400 ms, respectively), 64 scans, sw 40 ppm, aq 0.72 s and d1 5 s. The data were multiplied with an exponential window function with 1 Hz line broadening prior to Fourier transformation for ^1^H 1D, ^1^H T_2_, and ^19^F T_2_ filter experiments and 2 Hz line broadening for the WaterLOGSY experiments. The solubility of the compounds were evaluated by ^1^H 1D experiments and aggregation by WaterLOGSY, testing the compounds in the binding assay buffer at theoretical concentrations of 10, 50, and 100 μM in the presence of 400 μM 4-trifluoromethyl benzoic acid (internal reference).

To evaluate the stability and the aggregation state of both activated and inactive forms of His-CDC42, a mixture of 35 fluorinated fragments (soluble and not aggregating) was tested at 20 μM in the absence and in the presence of the proteins just after purification (*t*_0_) and 24 h later (*t*_1_) by ^19^F T_2_ filter experiments.

For the binding experiments, all the compounds were tested at 50 μM in 20 mM Trizma base (Sigma) pH 7.5, 40 mM NaCl, 5 mM MgCl_2_, 5 μM ethylenediaminetetraacetic acid, 10% D_2_O (for the lock signal) and in the presence of 2 μM GppNHp or GDP and 2 μM His-CDC42 (loaded with GDP) or His-CDC42 (loaded with GppNHp). The total amount of DMSO-*d*_6_ in all sample was 1%. All fluorine chemical shifts were referred to the CFCl_3_ signal in water.

## Supporting Information Available

^1^H, ^13^C, and ^19^F NMR spectral data and chromatography analysis of key compounds; MST and NMR analyses for binding evaluation; molecular docking of compounds **17**, **27**, and **28**; procedures for aqueous kinetic solubility, *in vitro* metabolic stability, and *in vitro* plasmatic stability; and PK studies (PDF)
Molecular formula strings (CSV)

## Abbreviations

DCM: dichloromethane
DMF: *N*,*N*-dimethylformamide
DMSO: dimethyl sulfoxide
EtOAc: ethyl acetate
MST: microscale thermophoresis
THF: tetrahydrofuran
