# Supplementary material for: Design, Synthesis, In Vitro and In Vivo Characterization of CDC42 GTPase Interaction Inhibitors for the Treatment of Cancer
Source: J Med Chem. 2023 Apr 7;66(8):5981–6001. doi: 10.1021/acs.jmedchem.3c00276 (PMC10150367; doi:10.1021/acs.jmedchem.3c00276)
Supplement: Supplementary file 1 — jm3c00276_si_001.pdf [file jm3c00276_si_001.pdf]

SUPPORTING INFORMATION

Design, Synthesis, In Vitro, and In Vivo Characterization of CDC42 GTPase Interaction Inhibitors for the Treatment of Cancer

Nicoletta Brindani,[#,1], Linh M. Vuong,[#,2] Isabella Maria Acquistapace,[#,1] Maria Antonietta La Serra,[1] José Antonio Ortega,[1] Marina Veronesi,[4] Sine Mandrup Bertozzi,[3] Maria Summa,[5] Stefania Girotto,[4] Rosalia Bertorelli,[5] Andrea Armirotti,[3] Anand K. Ganesan[*,2] Marco De Vivo[*,1]

1. Molecular Modeling and Drug Discovery Lab, Istituto Italiano di Tecnologia, via Morego 30, 16163 Genova, Italy

2. Department of Dermatology, University of California, Irvine, CA 92697, USA

3. Analytical Chemistry Facility, Istituto Italiano di Tecnologia, via Morego 30, 16163 Genova, Italy

4. Structural Biophysics Facility, Istituto Italiano di Tecnologia, via Morego 30, 16163 Genova, Italy

5. Translational Pharmacology Facility, Istituto Italiano di Tecnologia, via Morego 30, 16163, Genova, Italy

Corresponding author:

Dr. Marco De Vivo – Email: marco.devivo@iit.it

Dr. Anand K. Ganesan- Email: aganesan@uci.edu

#Equally contributed

*co- corresponding authors

Table of contents

- 1. ^{1}H-NMR, ^{13}C-NMR and ^{19}F-NMR spectra S2
- 2. Chromatography analysis of representative compounds S57
- 3. Table S1 S72
- 4. Figure S1 S74
- 5. Figure S2 S75
- 6. Figure S3 S76
- 7. *In vitro* metabolic stability S77
- 8. Aqueous kinetic solubility S77
- 9. Pharmacokinetic studies S78

1. ^{1}H, ^{13}C, ^{19}F NMR Spectra

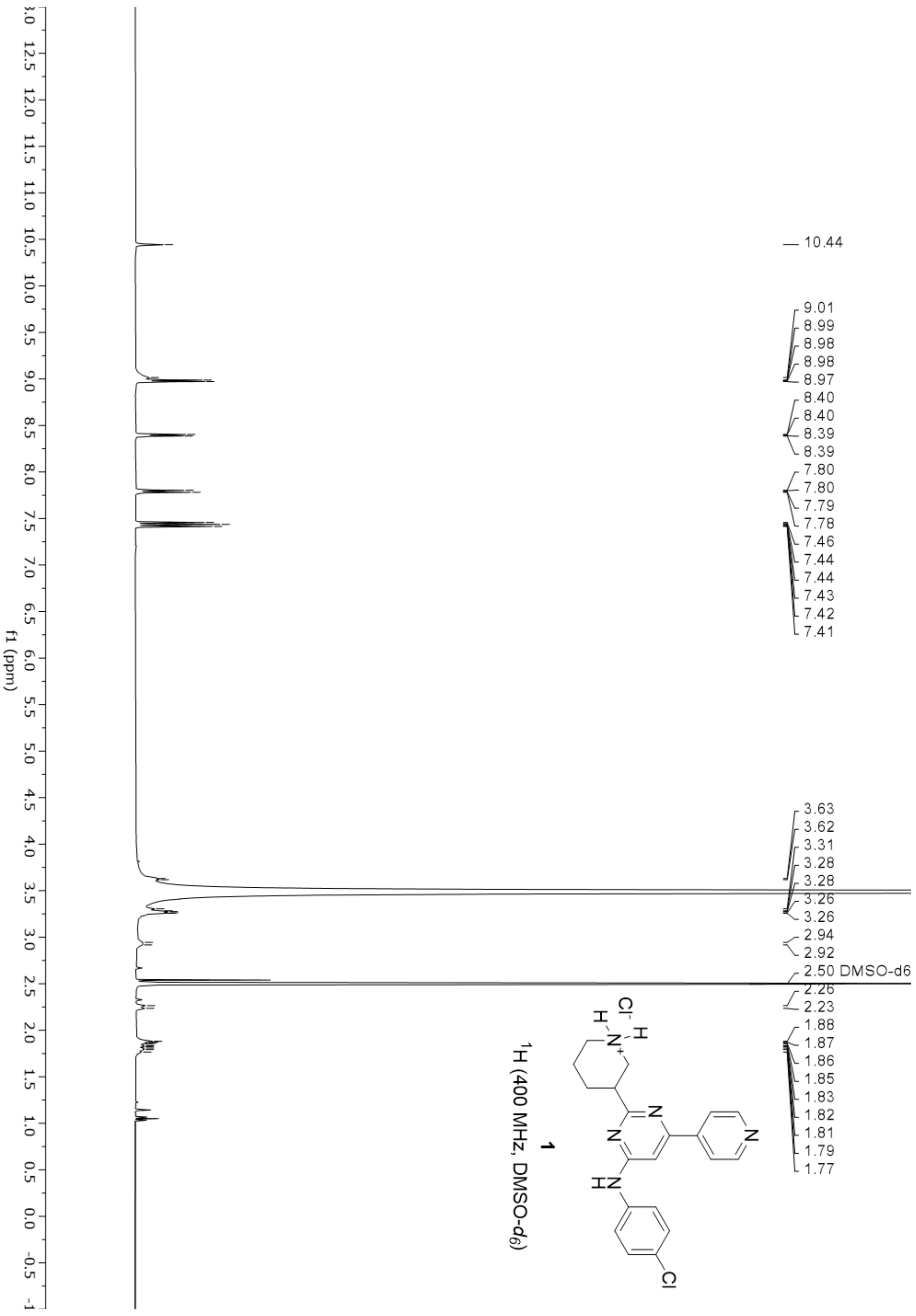

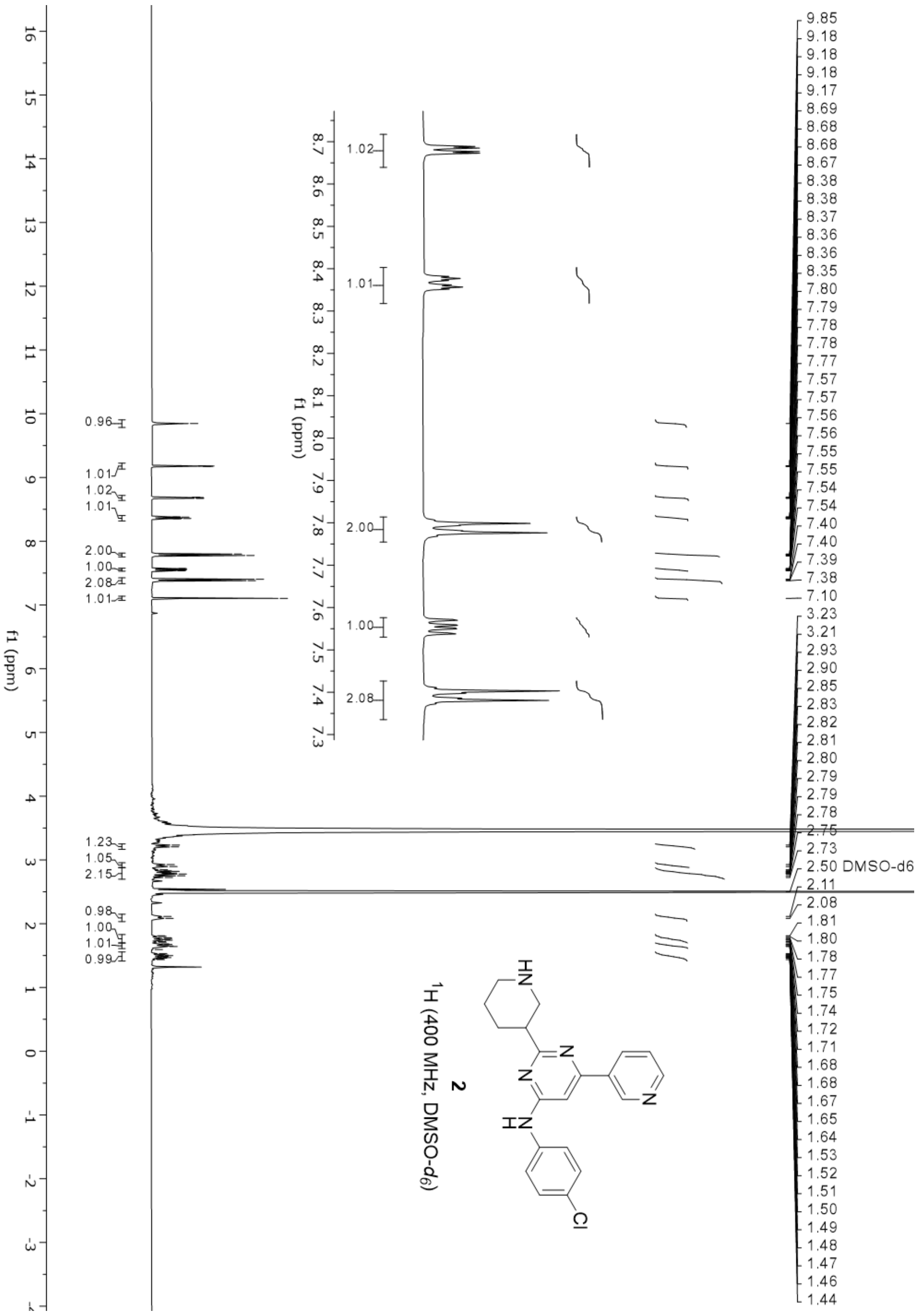
9.85
9.18
9.18
9.18
9.17
8.69
8.68
8.68
8.67
8.38
8.38
8.37
8.36
8.36
8.35
7.80
7.79
7.78
7.78
7.77
7.57
7.57
7.56
7.56
7.55
7.55
7.54
7.54
7.40
7.40
7.39
7.38
7.10
3.23
3.21
2.93
2.90
2.85
2.83
2.82
2.81
2.80
2.79
2.79
2.78
2.75
2.73
2.50 DMSO-d6
2.11
2.08
1.81
1.80
1.78
1.77
1.75
1.74
1.72
1.71
1.68
1.68
1.67
1.65
1.64
1.53
1.52
1.51
1.50
1.49
1.48
1.47
1.46
1.44
f1 (ppm)
2
1H (400 MHz, DMSO-d6)

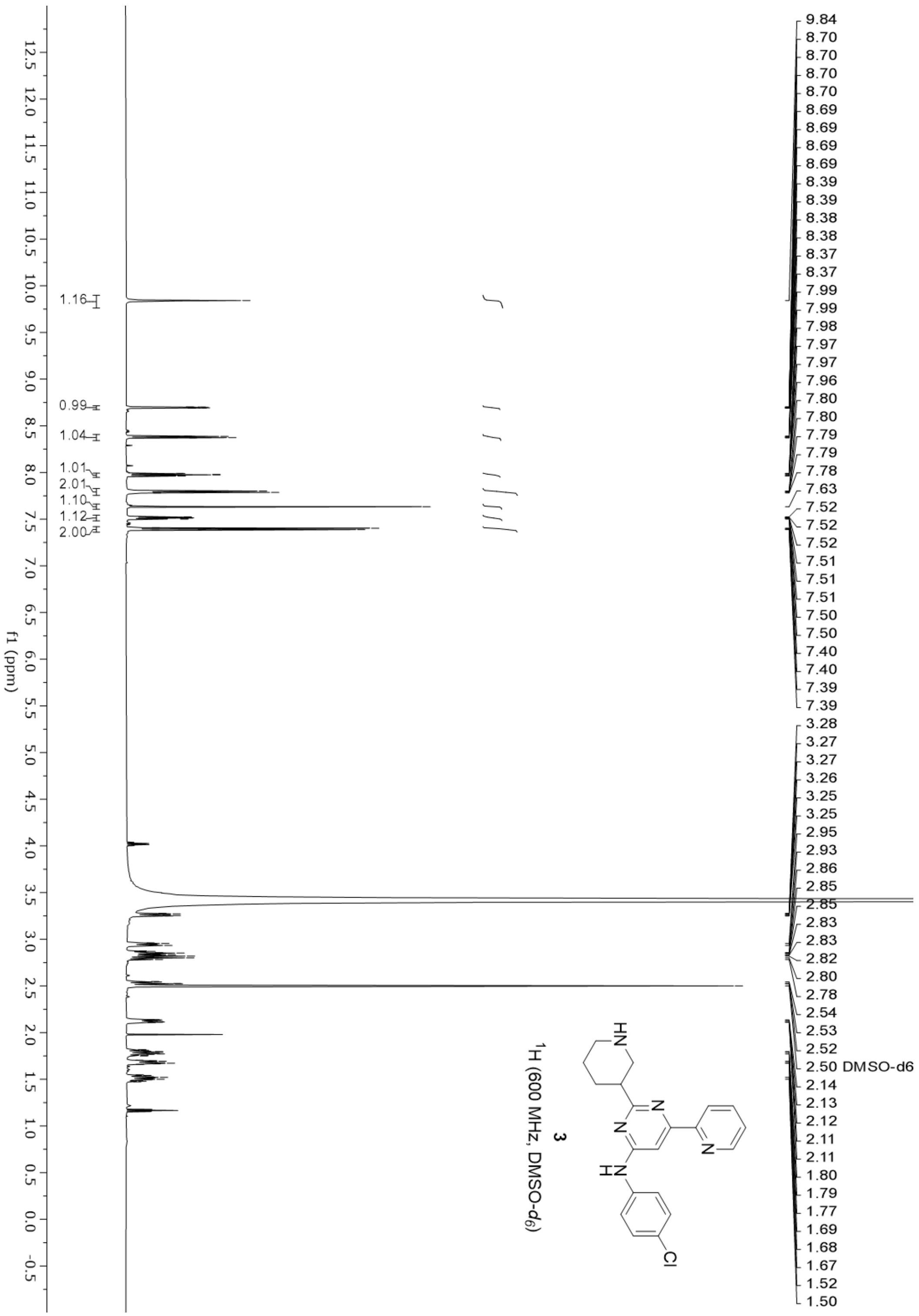
9.84
8.70
8.70
8.70
8.70
8.69
8.69
8.69
8.69
8.39
8.39
8.38
8.38
8.37
8.37
7.99
7.99
7.98
7.97
7.97
7.96
7.80
7.80
7.79
7.79
7.78
7.63
7.52
7.52
7.52
7.51
7.51
7.51
7.50
7.50
7.40
7.40
7.39
7.39
3.28
3.27
3.27
3.26
3.25
3.25
2.95
2.93
2.86
2.85
2.85
2.83
2.83
2.82
2.80
2.78
2.54
2.53
2.52
2.50 DMSO-d6
2.14
2.13
2.12
2.11
2.11
1.80
1.79
1.77
1.69
1.68
1.67
1.52
1.50
1.16
0.99
1.04
1.01
2.01
1.10
1.12
2.00
f1 (ppm)
3
1H (600 MHz, DMSO-d6)

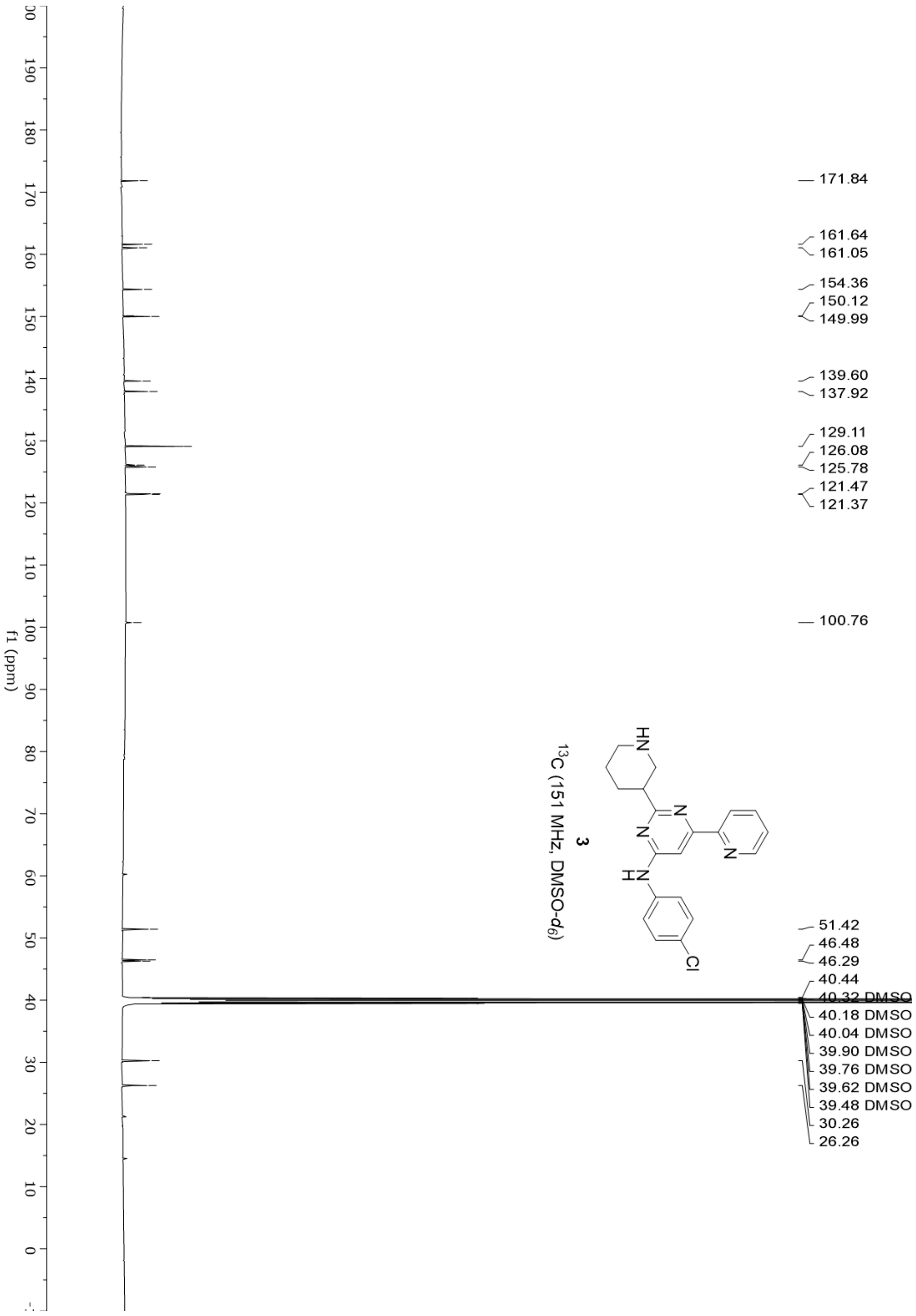
171.84
161.64
161.05
154.36
150.12
149.99
139.60
137.92
129.11
126.08
125.78
121.47
121.37
100.76
51.42
46.48
46.29
40.44
40.32 DMSO
40.18 DMSO
40.04 DMSO
39.90 DMSO
39.76 DMSO
39.62 DMSO
39.48 DMSO
30.26
26.26
3
^{13}C (151 MHz, DMSO-d_6)
f1 (ppm)
HN
N
N
N
H
Cl

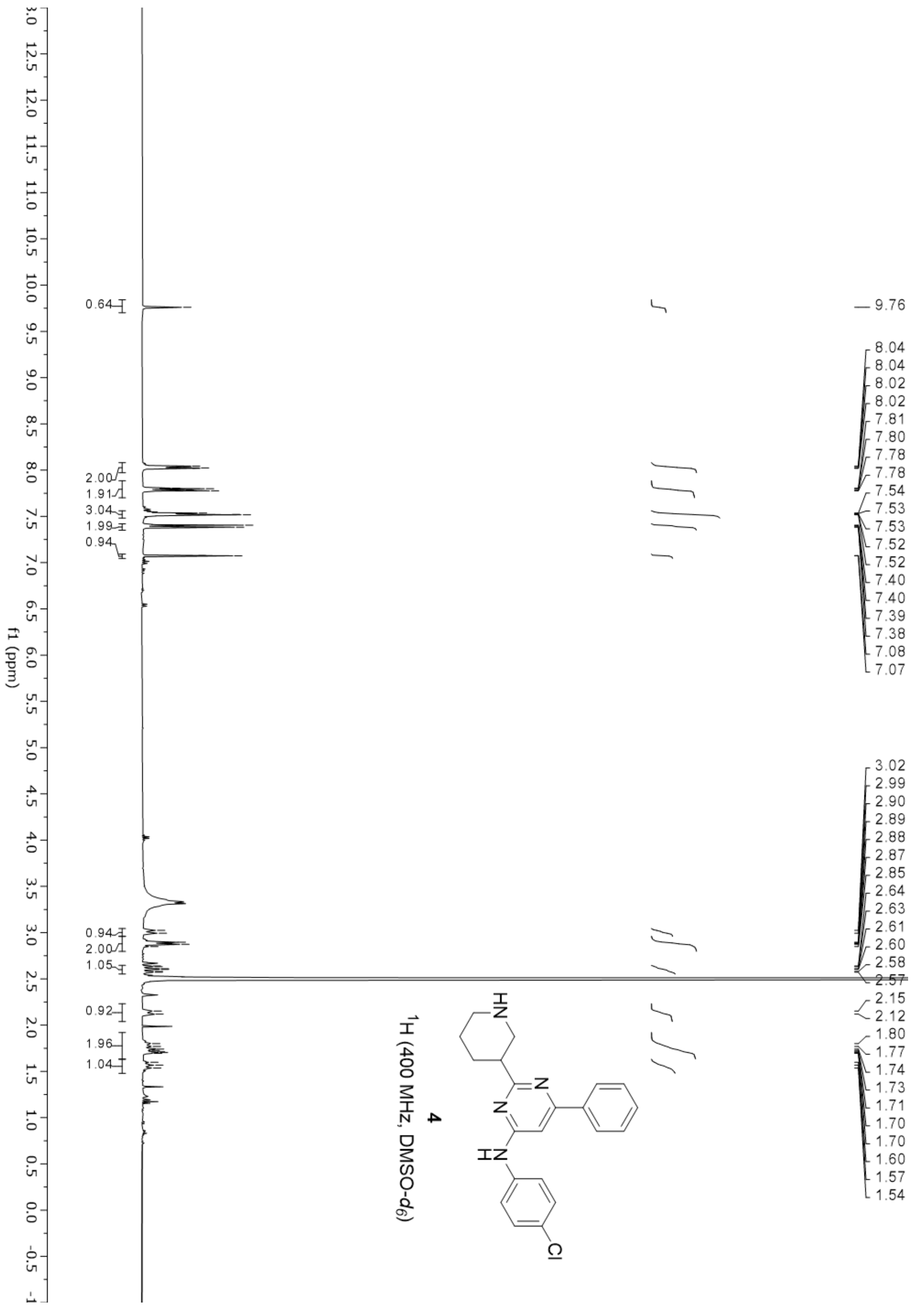
9.76
8.04
8.04
8.02
8.02
7.81
7.80
7.78
7.78
7.54
7.53
7.53
7.52
7.52
7.40
7.40
7.39
7.38
7.08
7.07
3.02
2.99
2.90
2.89
2.88
2.87
2.85
2.64
2.63
2.61
2.60
2.58
2.57
2.15
2.12
1.80
1.77
1.74
1.73
1.71
1.70
1.70
1.60
1.57
1.54
0.64
2.00
1.91
3.04
1.99
0.94
0.94
2.00
1.05
0.92
1.96
1.04
f1 (ppm)
HN
N
N
N
H
Cl
4
1H (400 MHz, DMSO-d6)

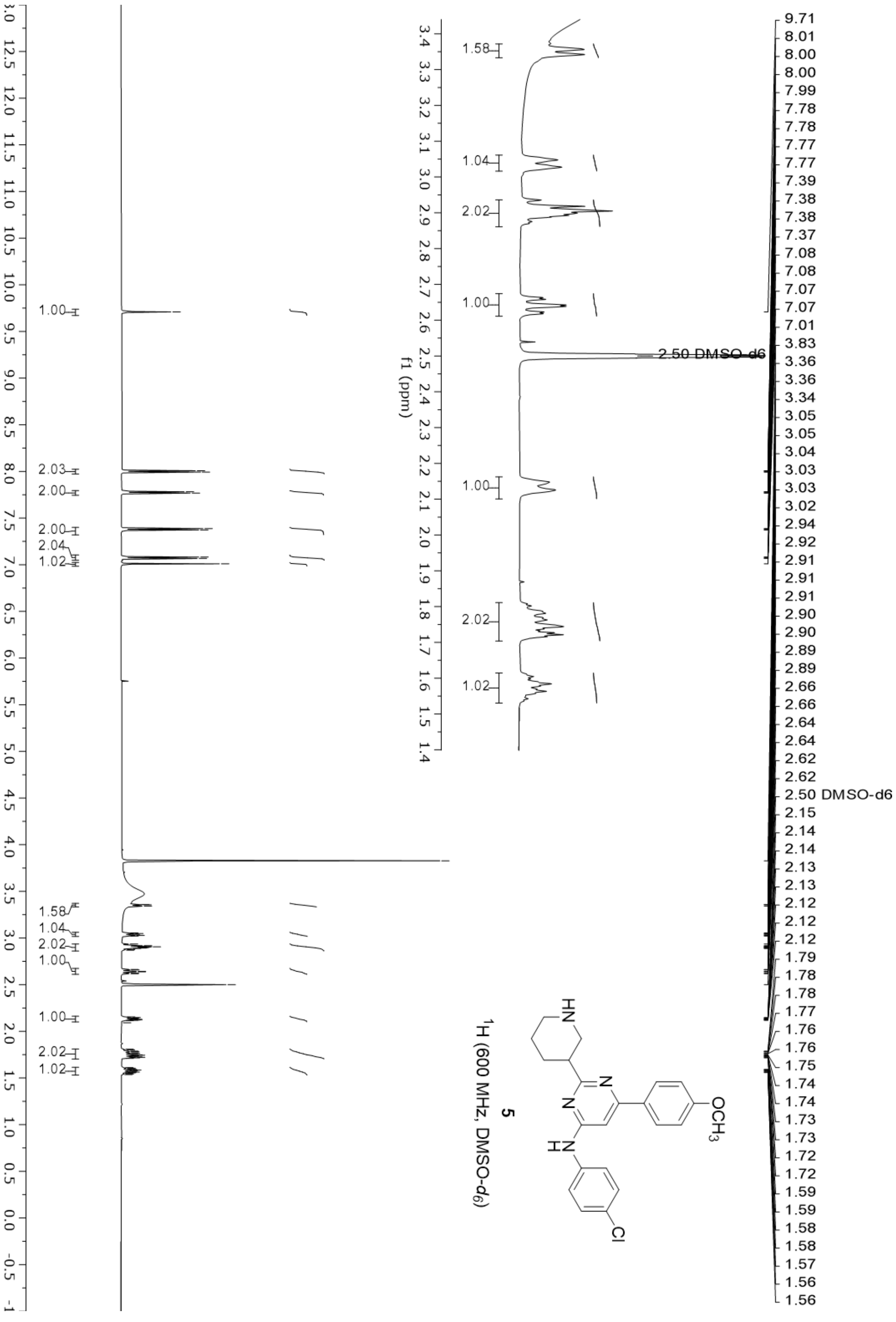
9.71
8.01
8.00
8.00
7.99
7.78
7.78
7.77
7.77
7.39
7.38
7.38
7.37
7.08
7.08
7.07
7.07
7.01
3.83
3.36
3.36
3.34
3.05
3.05
3.04
3.03
3.03
3.02
2.94
2.92
2.91
2.91
2.91
2.90
2.90
2.89
2.89
2.66
2.66
2.64
2.64
2.62
2.62
2.50 DMSO-d6
2.15
2.14
2.14
2.13
2.13
2.12
2.12
2.12
1.79
1.78
1.78
1.77
1.76
1.76
1.75
1.74
1.74
1.73
1.73
1.72
1.72
1.59
1.59
1.58
1.58
1.57
1.56
1.56
OCH3
HN
N
N
H
Cl
5
1H (600 MHz, DMSO-d6)
f1 (ppm)

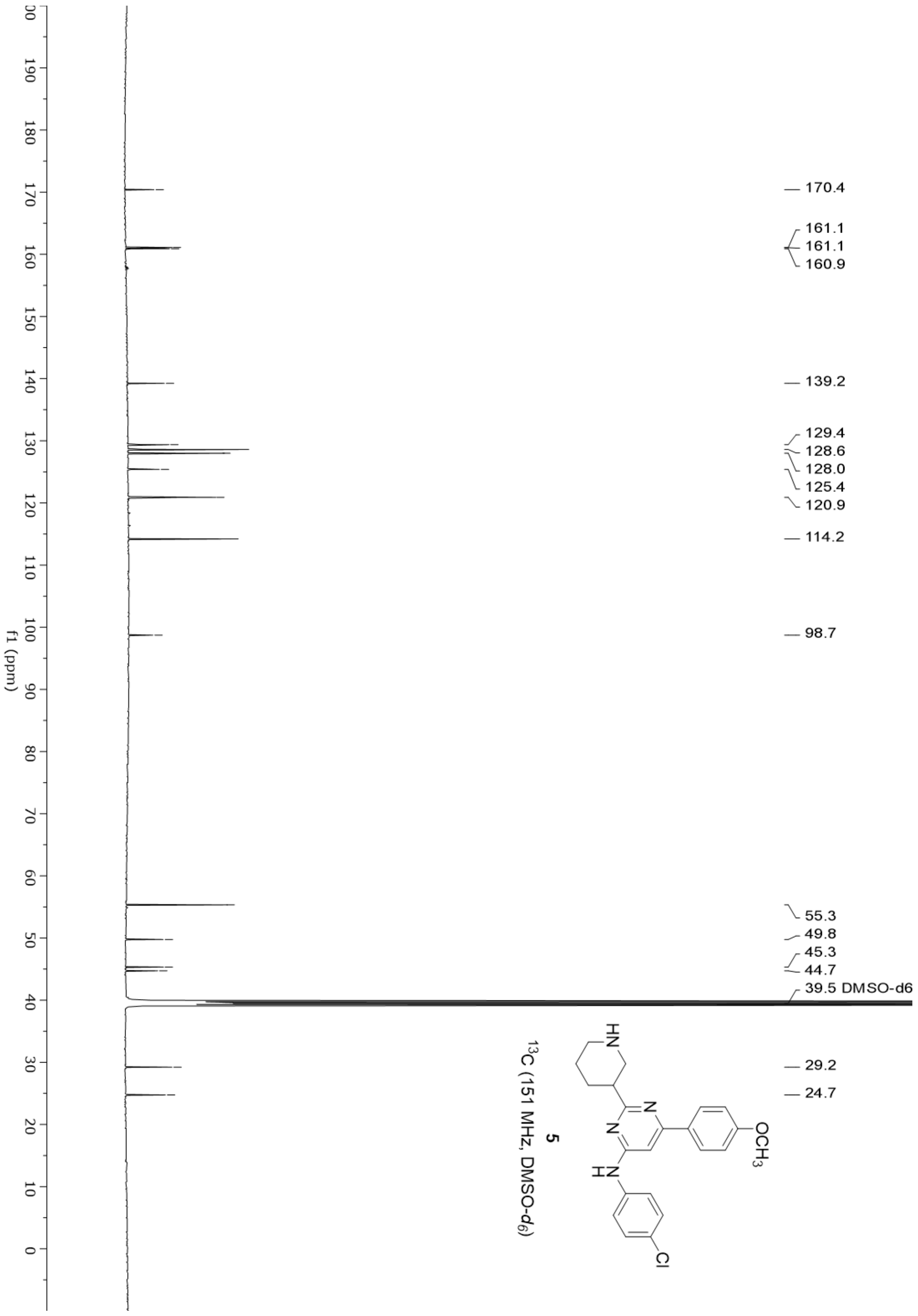
170.4
161.1
161.1
160.9
139.2
129.4
128.6
128.0
125.4
120.9
114.2
98.7
55.3
49.8
45.3
44.7
39.5 DMSO-d6
29.2
24.7
f1 (ppm)
HN
N
N
N
H
OCH3
Cl
5
13C (151 MHz, DMSO-d6)

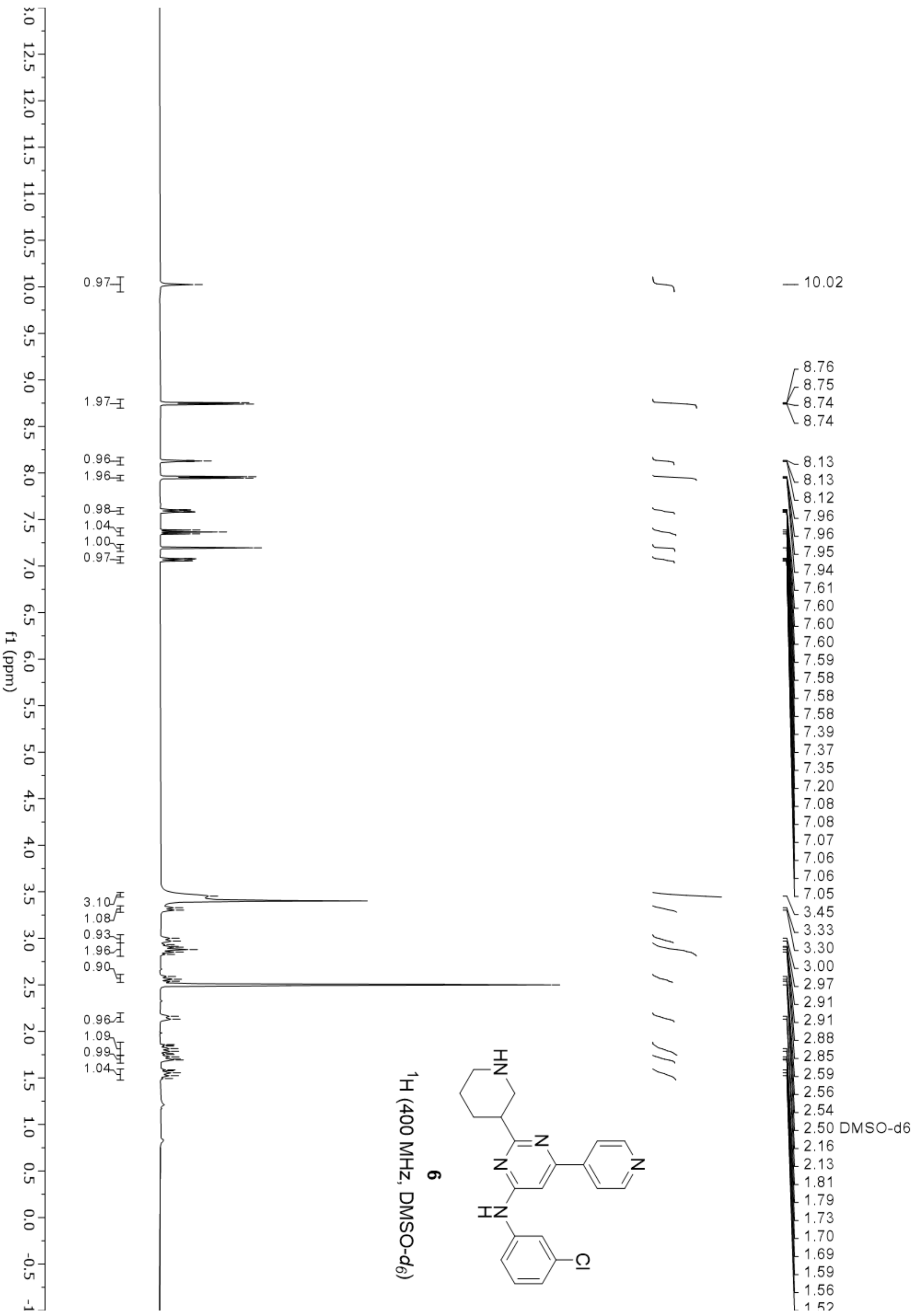
10.02
8.76
8.75
8.74
8.74
8.13
8.13
8.12
7.96
7.96
7.95
7.94
7.61
7.60
7.60
7.60
7.59
7.58
7.58
7.58
7.39
7.37
7.35
7.20
7.08
7.08
7.07
7.06
7.06
7.05
3.45
3.33
3.30
3.00
2.97
2.91
2.91
2.88
2.85
2.59
2.56
2.54
2.50 DMSO-d6
2.16
2.13
1.81
1.79
1.73
1.70
1.69
1.59
1.56
0.97
1.97
0.96
1.96
0.98
1.04
1.00
0.97
3.10
1.08
0.93
1.96
0.90
0.96
1.09
0.99
1.04
f1 (ppm)
6
1H (400 MHz, DMSO-d6)

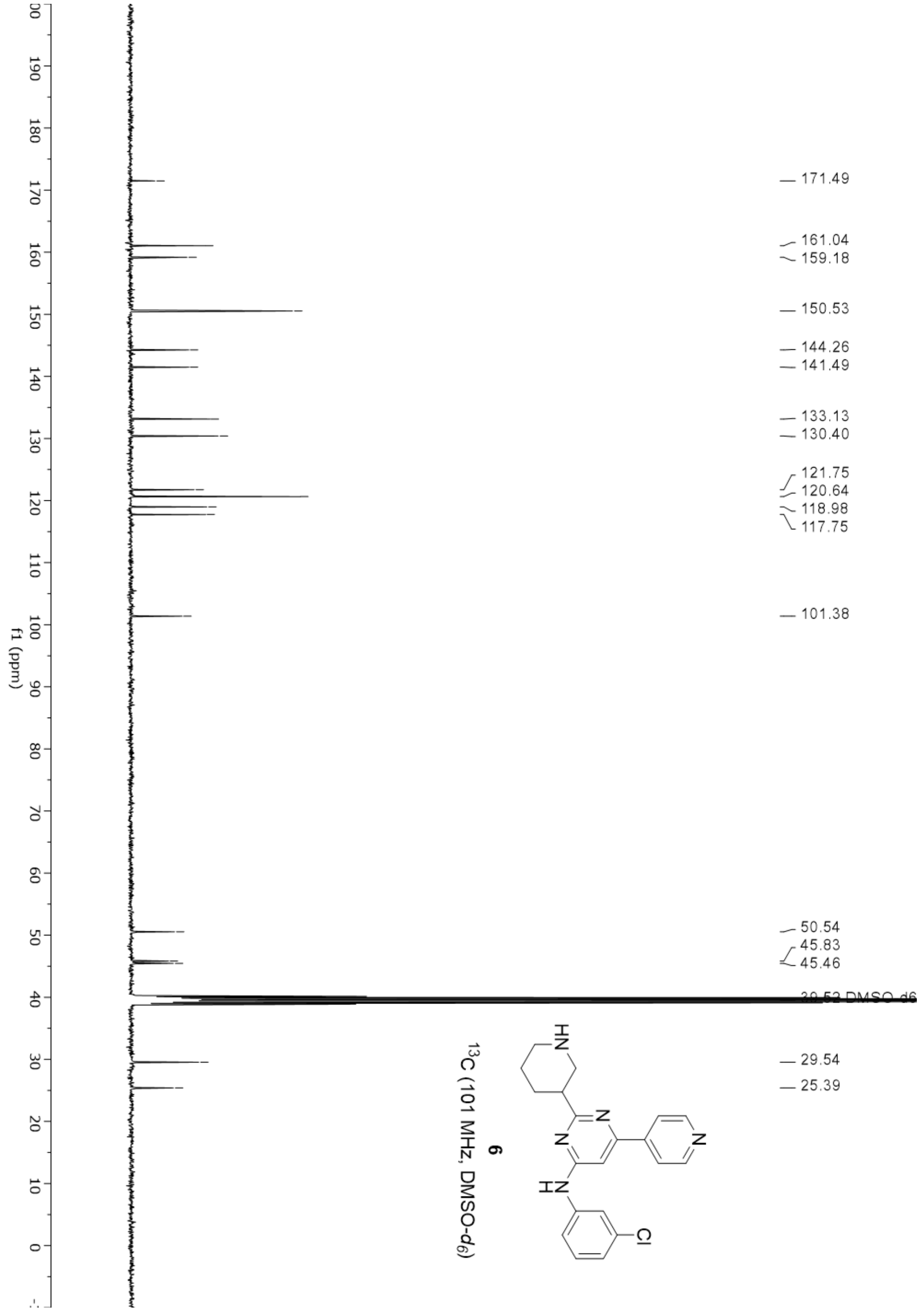

6

^{13}C (101 MHz, DMSO-d_6)

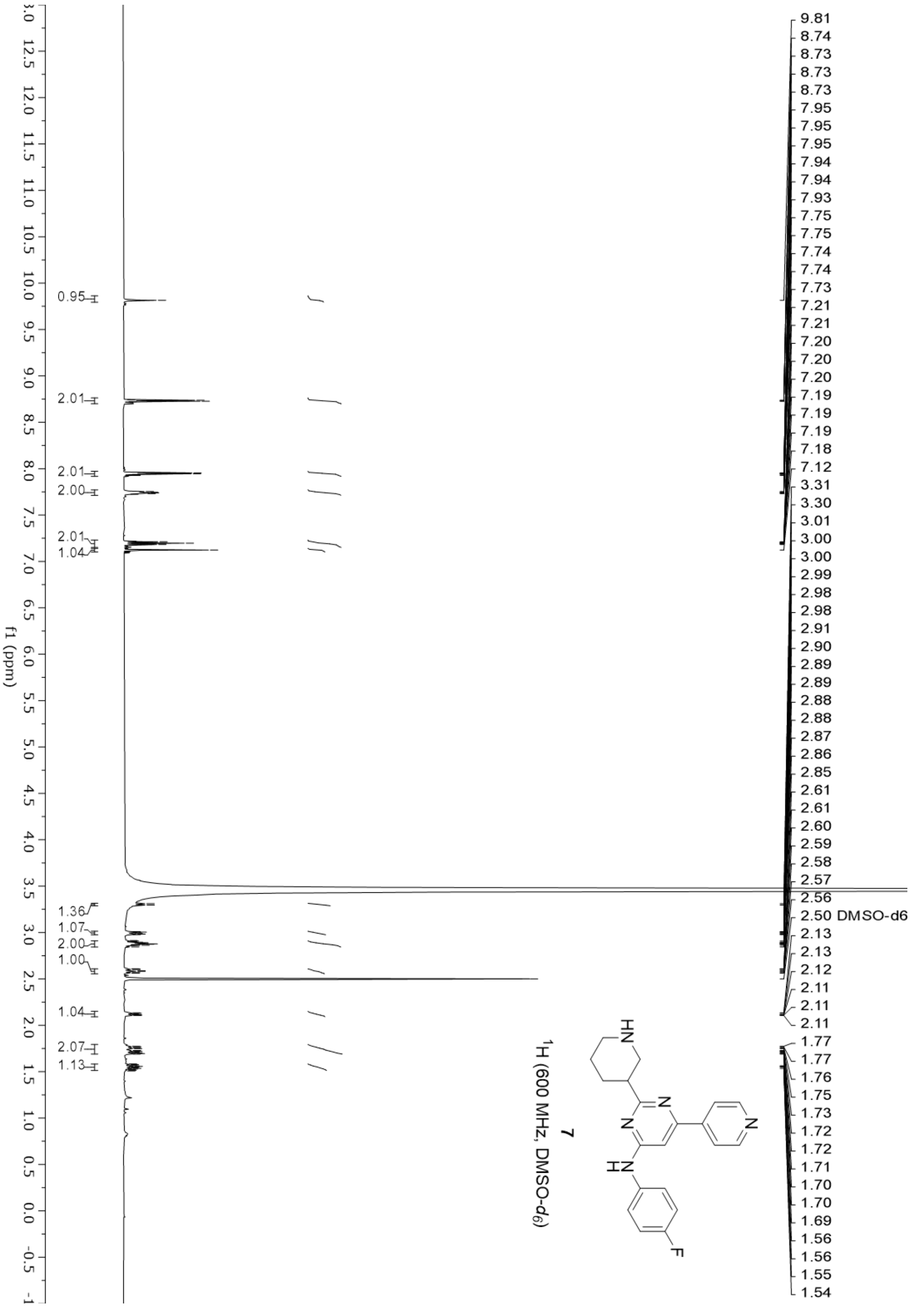
9.81
8.74
8.73
8.73
8.73
7.95
7.95
7.95
7.94
7.94
7.93
7.75
7.75
7.74
7.74
7.73
7.21
7.21
7.20
7.20
7.20
7.19
7.19
7.19
7.18
7.12
3.31
3.30
3.01
3.00
3.00
2.99
2.98
2.98
2.91
2.90
2.89
2.89
2.88
2.88
2.87
2.86
2.85
2.61
2.61
2.60
2.59
2.58
2.57
2.56
2.50 DMSO-d6
2.13
2.13
2.12
2.11
2.11
2.11
1.77
1.77
1.76
1.75
1.73
1.72
1.72
1.71
1.70
1.70
1.69
1.56
1.56
1.55
1.54
0.95
2.01
2.01
2.00
2.01
1.04
1.36
1.07
2.00
1.00
1.04
2.07
1.13
f1 (ppm)
7
1H (600 MHz, DMSO-d6)

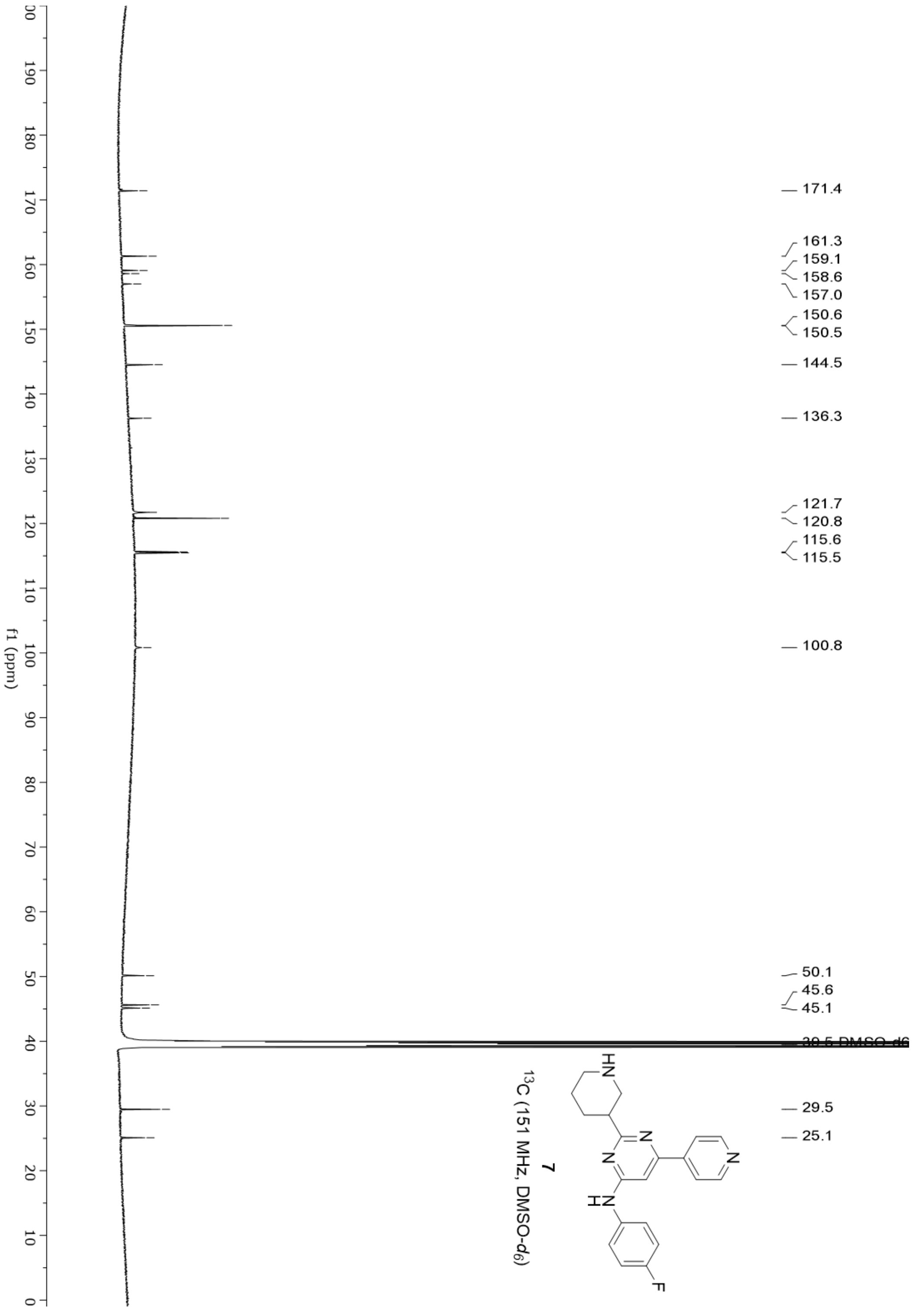
171.4
161.3
159.1
158.6
157.0
150.6
150.5
144.5
136.3
121.7
120.8
115.6
115.5
100.8
50.1
45.6
45.1
39.5 DMSO-d6
29.5
25.1
f1 (ppm)
7
13C (151 MHz, DMSO-d6)

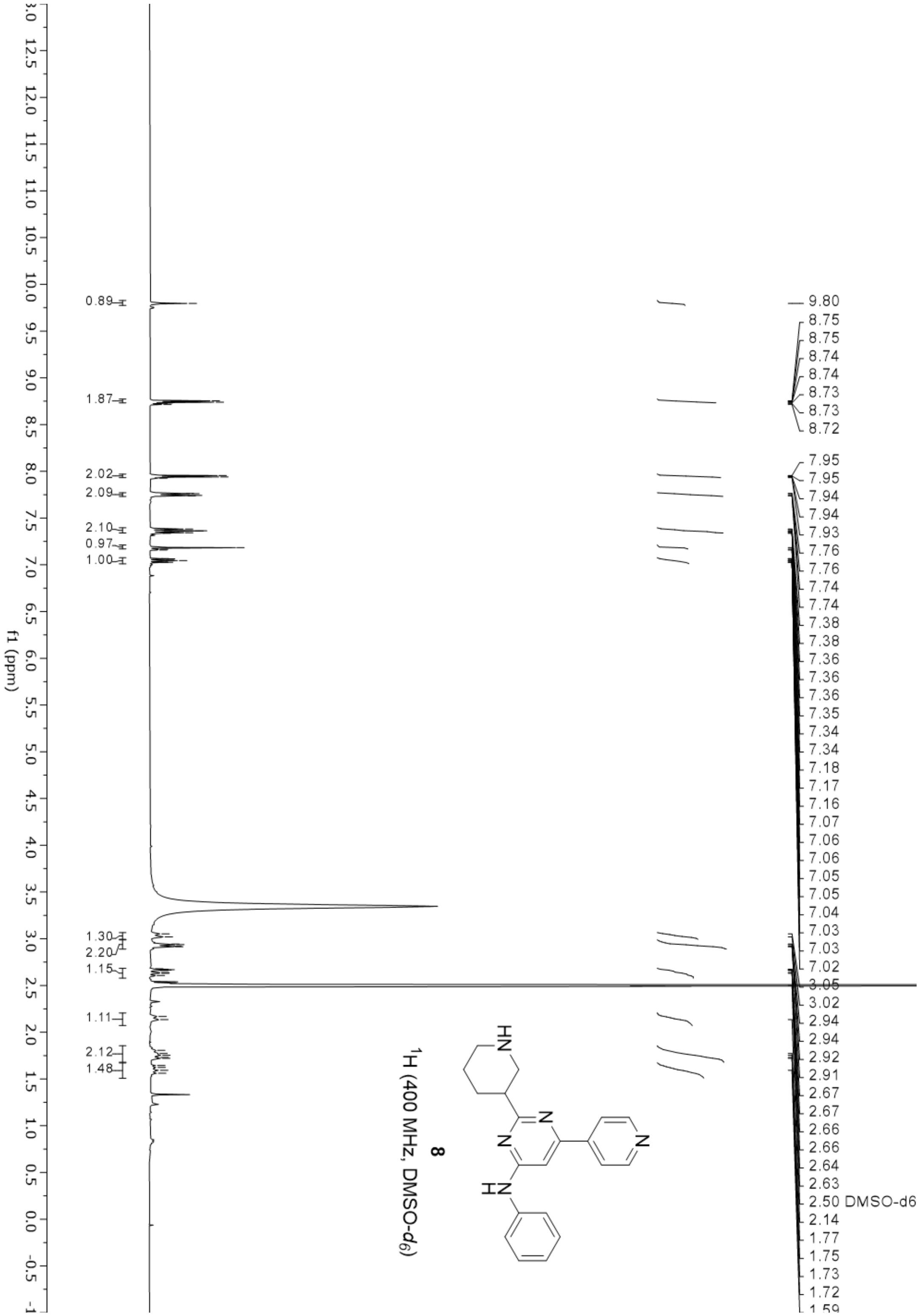
9.80
8.75
8.75
8.74
8.74
8.73
8.73
8.72
7.95
7.95
7.94
7.94
7.93
7.76
7.76
7.74
7.74
7.38
7.38
7.36
7.36
7.36
7.35
7.34
7.34
7.18
7.17
7.16
7.07
7.06
7.06
7.05
7.05
7.04
7.03
7.03
7.02
3.05
3.02
2.94
2.94
2.92
2.91
2.67
2.67
2.66
2.66
2.64
2.63
2.50 DMSO-d6
2.14
1.77
1.75
1.73
1.72
f1 (ppm)
8
^{1}H (400 MHz, DMSO-d_6)

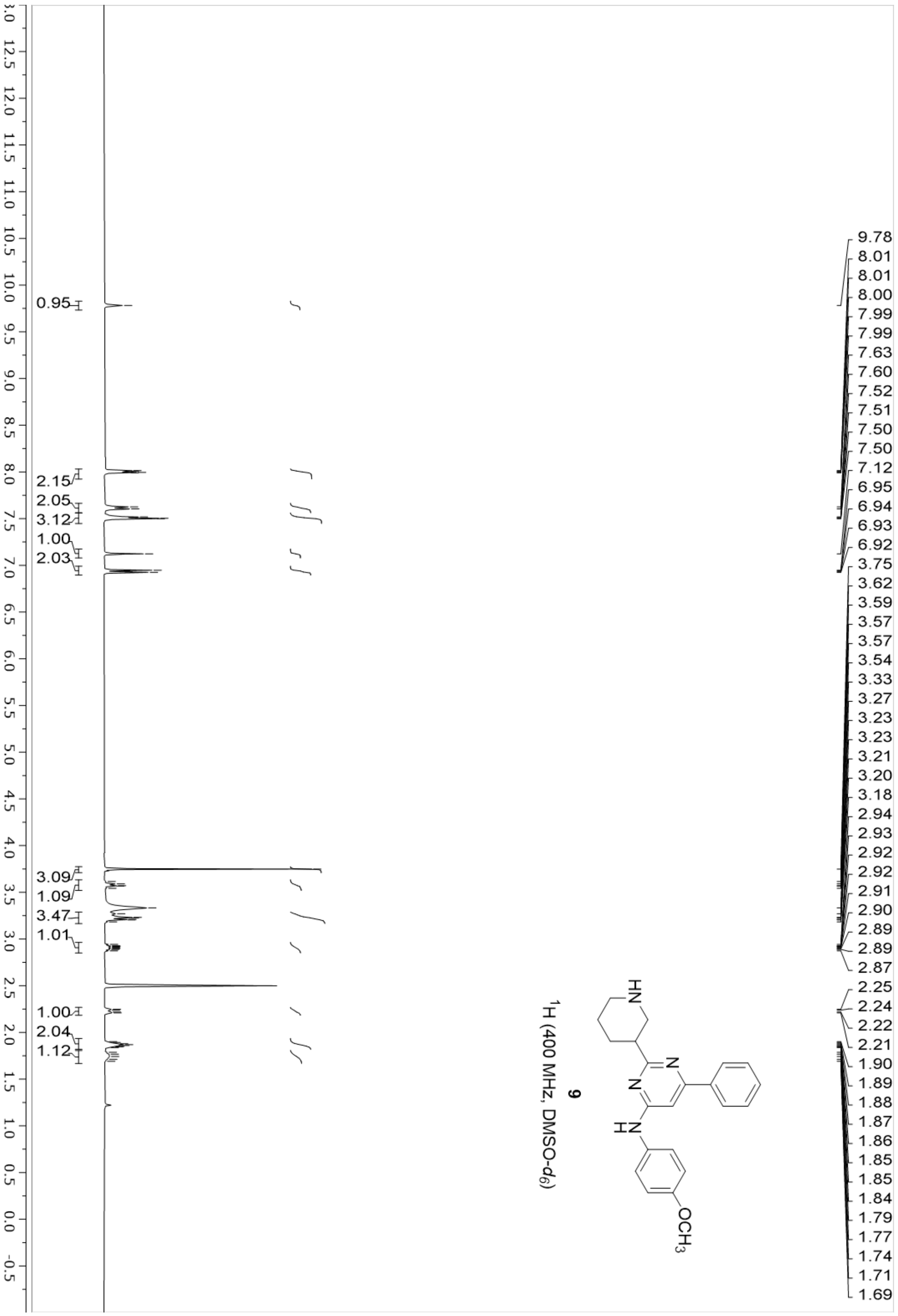
9.78
8.01
8.01
8.00
7.99
7.99
7.63
7.60
7.52
7.51
7.50
7.50
7.12
6.95
6.94
6.93
6.92
3.75
3.62
3.59
3.57
3.57
3.54
3.33
3.27
3.23
3.23
3.21
3.20
3.18
2.94
2.93
2.92
2.92
2.91
2.90
2.89
2.89
2.87
2.25
2.24
2.22
2.21
1.90
1.89
1.88
1.87
1.86
1.85
1.85
1.84
1.79
1.77
1.74
1.71
1.69
0.95
2.15
2.05
3.12
1.00
2.03
3.09
1.09
3.47
1.01
1.00
2.04
1.12
HN
N
N
N
H
OCH3
9
1H (400 MHz, DMSO-d6)
f1 (ppm)

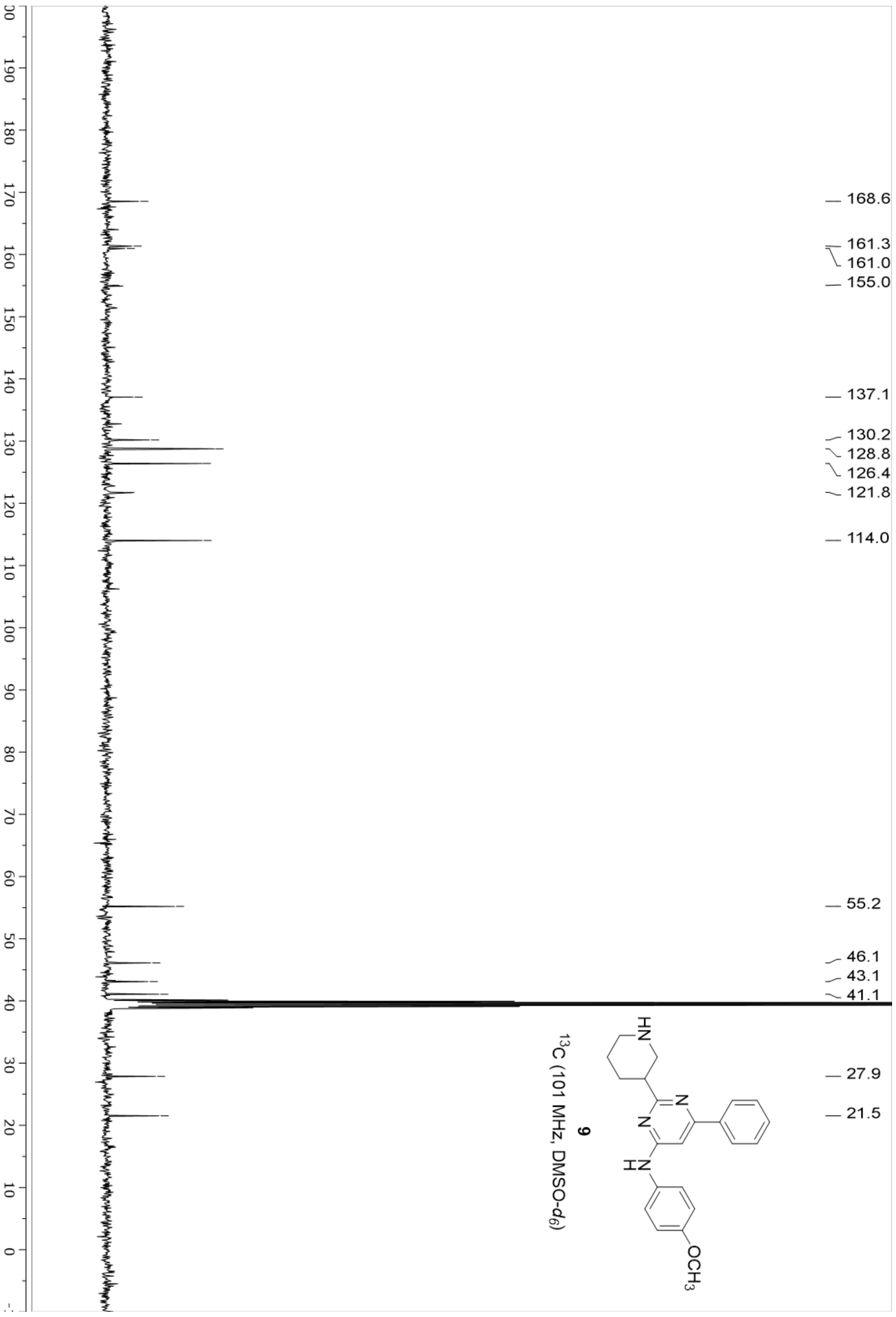

168.6
161.3
161.0
155.0
137.1
130.2
128.8
126.4
121.8
114.0
55.2
46.1
43.1
41.1
27.9
21.5
f1 (ppm)
OCH3
9
13C (101 MHz, DMSO-d6)

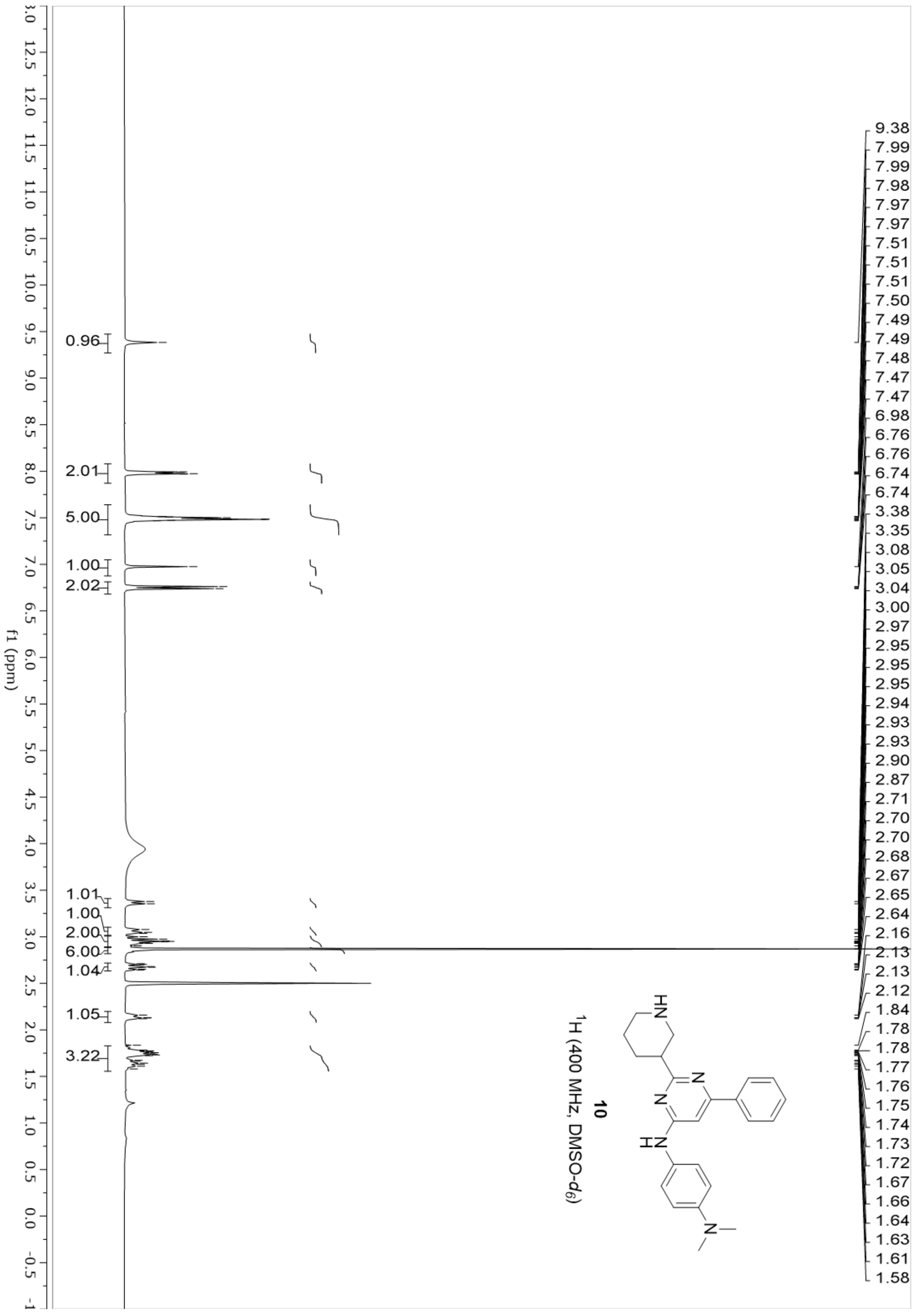
10
1H (400 MHz, DMSO-d6)
f1 (ppm)
0.96
2.01
5.00
1.00
2.02
1.01
1.00
2.00
6.00
1.04
1.05
3.22
9.38
7.99
7.99
7.98
7.97
7.97
7.51
7.51
7.51
7.50
7.49
7.49
7.48
7.47
7.47
6.98
6.76
6.76
6.74
6.74
3.38
3.35
3.08
3.05
3.04
3.00
2.97
2.95
2.95
2.95
2.94
2.93
2.93
2.90
2.87
2.71
2.70
2.70
2.68
2.67
2.65
2.64
2.16
2.13
2.13
2.12
1.84
1.78
1.78
1.77
1.76
1.75
1.74
1.73
1.72
1.67
1.66
1.64
1.63
1.61
1.58

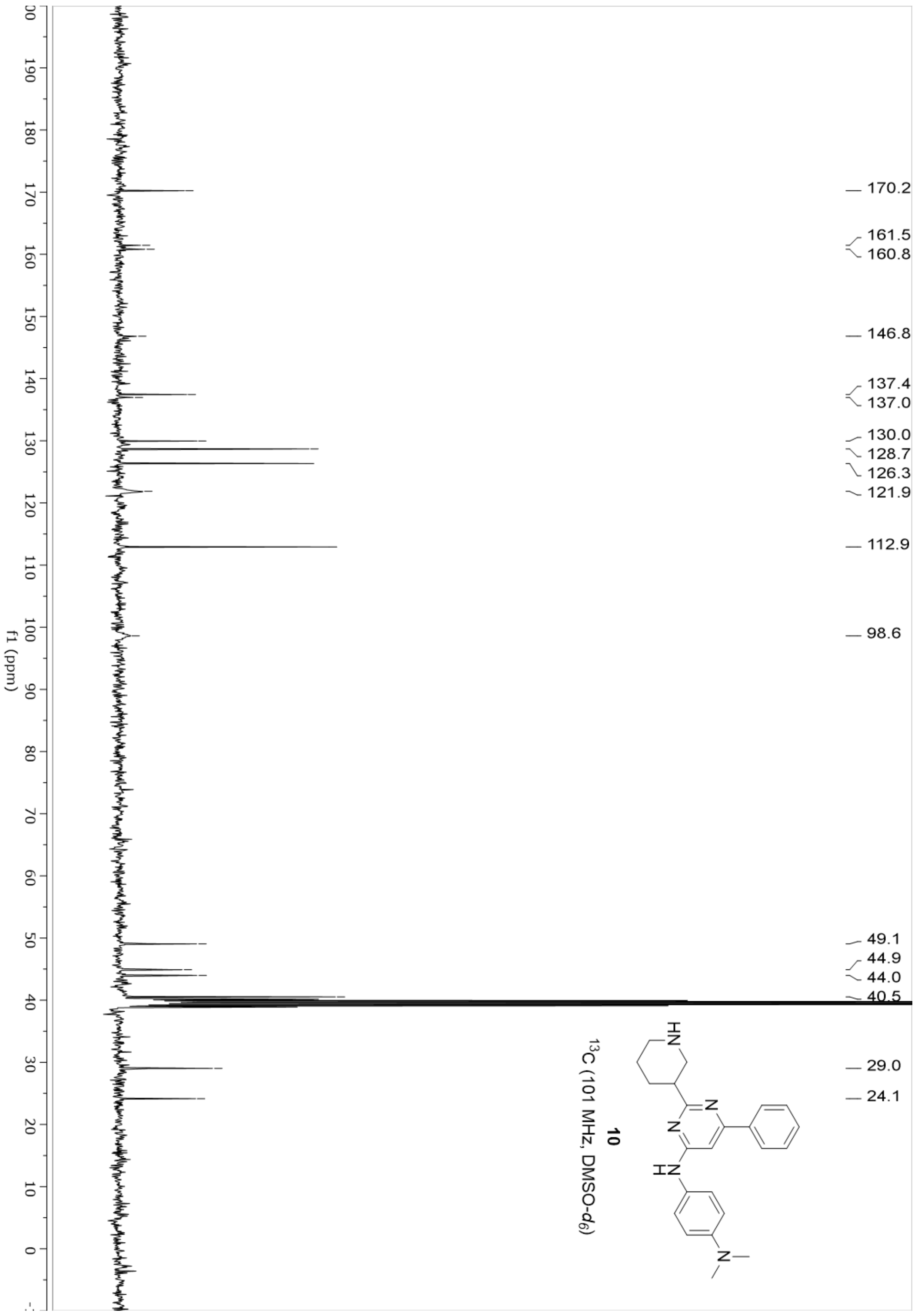
170.2
161.5
160.8
146.8
137.4
137.0
130.0
128.7
126.3
121.9
112.9
98.6
49.1
44.9
44.0
40.5
29.0
24.1
f1 (ppm)
10
13C (101 MHz, DMSO-d6)

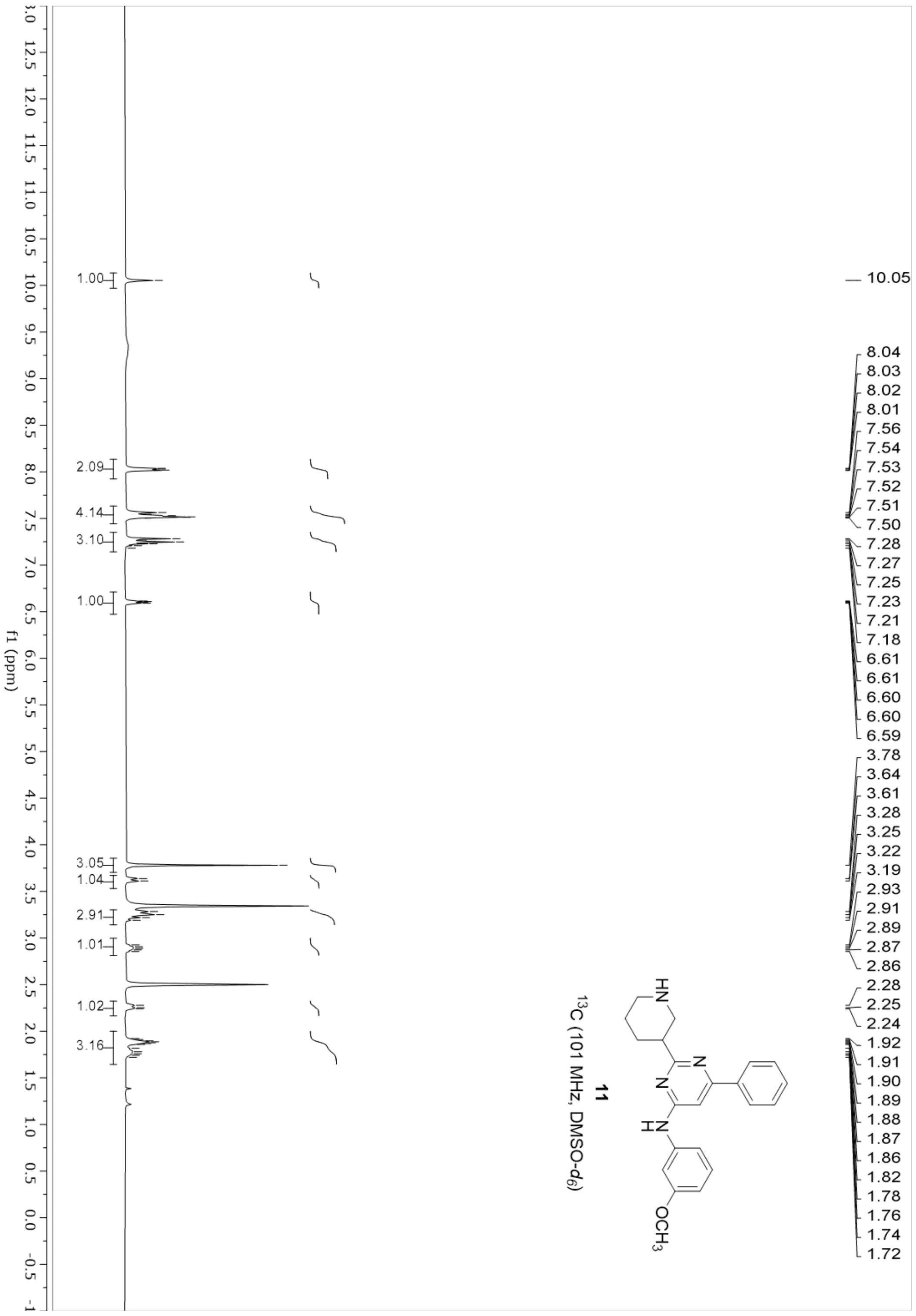
11
13C (101 MHz, DMSO-d6)
HN
N
N
N
H
OCH3
f1 (ppm)
1.00
2.09
4.14
3.10
1.00
3.05
1.04
2.91
1.01
1.02
3.16
10.05
8.04
8.03
8.02
8.01
7.56
7.54
7.53
7.52
7.51
7.50
7.28
7.27
7.25
7.23
7.21
7.18
6.61
6.61
6.60
6.60
6.59
3.78
3.64
3.61
3.28
3.25
3.22
3.19
2.93
2.91
2.89
2.87
2.86
2.28
2.25
2.24
1.92
1.91
1.90
1.89
1.88
1.87
1.86
1.82
1.78
1.76
1.74
1.72

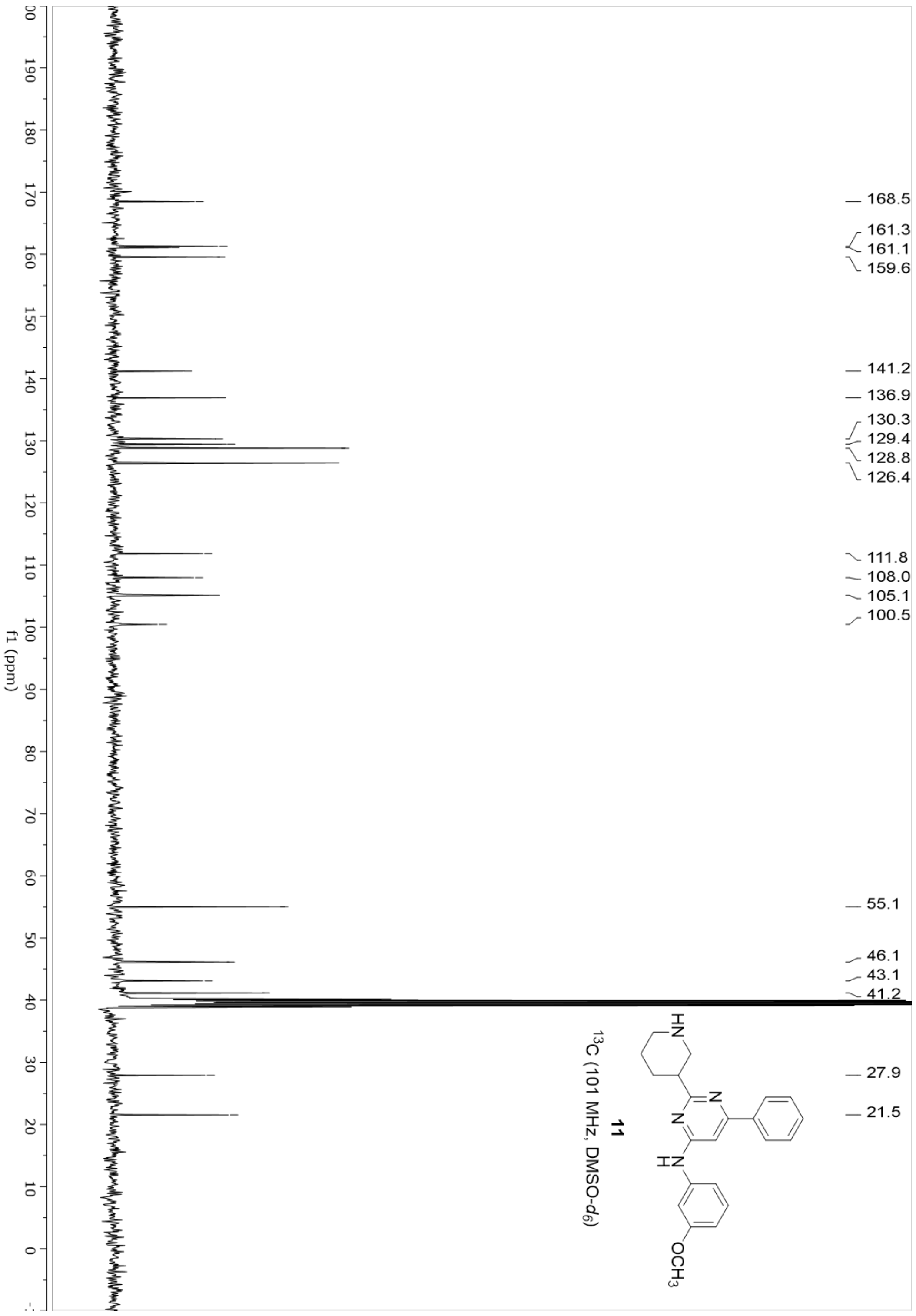
168.5
161.3
161.1
159.6
141.2
136.9
130.3
129.4
128.8
126.4
111.8
108.0
105.1
100.5
55.1
46.1
43.1
41.2
27.9
21.5
f1 (ppm)
11
^{13}C (101 MHz, DMSO-d_6)
HN
N
H
OCH_3

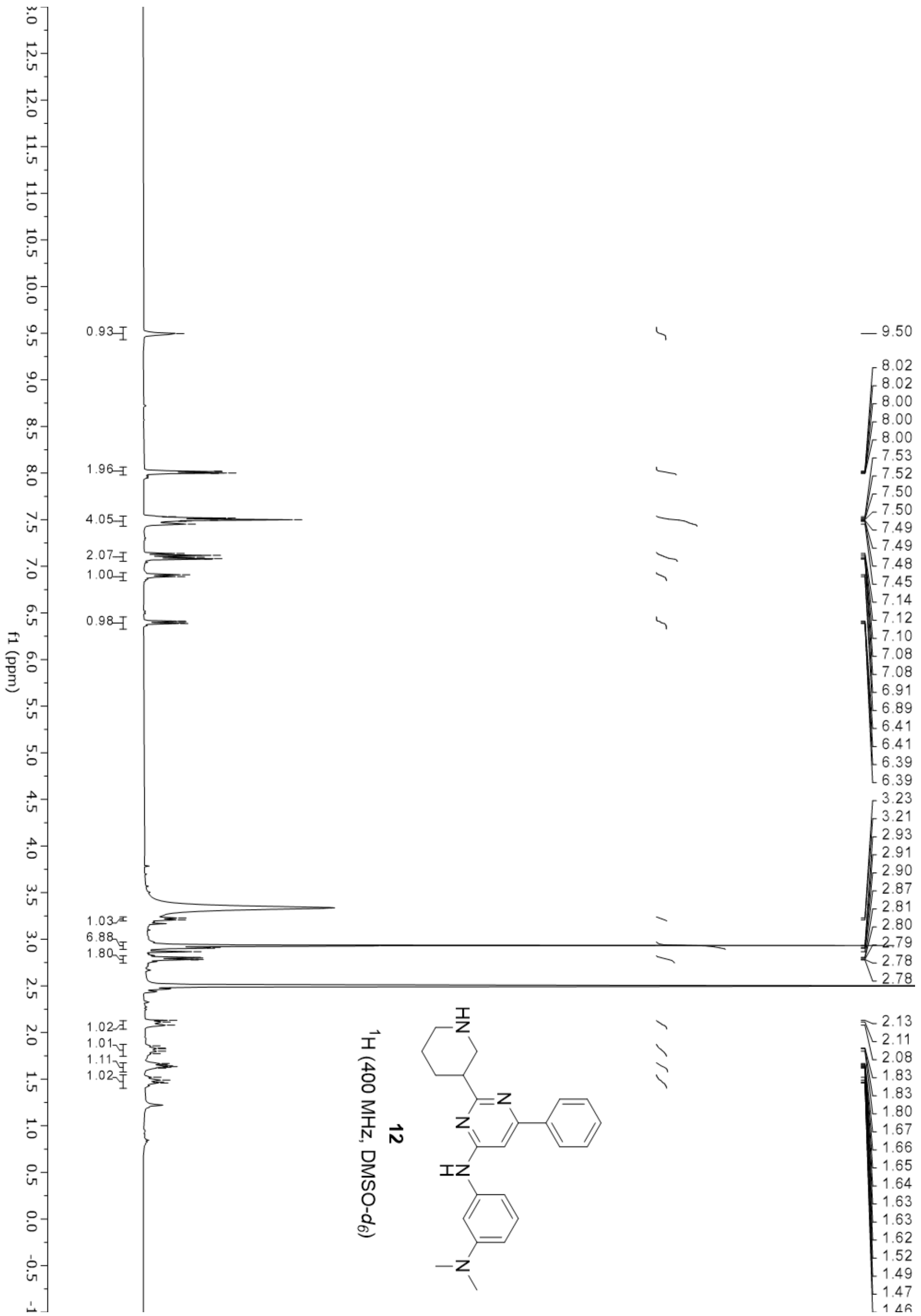

12
1H (400 MHz, DMSO-d6)
f1 (ppm)
9.50
8.02 8.02 8.00 8.00 8.00 7.53 7.52 7.50 7.50 7.49 7.49 7.48 7.45 7.14 7.12 7.10 7.08 7.08 6.91 6.89 6.41 6.41 6.39 6.39
3.23 3.21 2.93 2.91 2.90 2.87 2.81 2.80 2.79 2.78 2.78
2.13 2.11 2.08 1.83 1.83 1.80 1.67 1.66 1.65 1.64 1.63 1.63 1.62 1.52 1.49 1.47 1.46
0.93 1.96 4.05 2.07 1.00 0.98 1.03 6.88 1.80 1.02 1.01 1.11 1.02

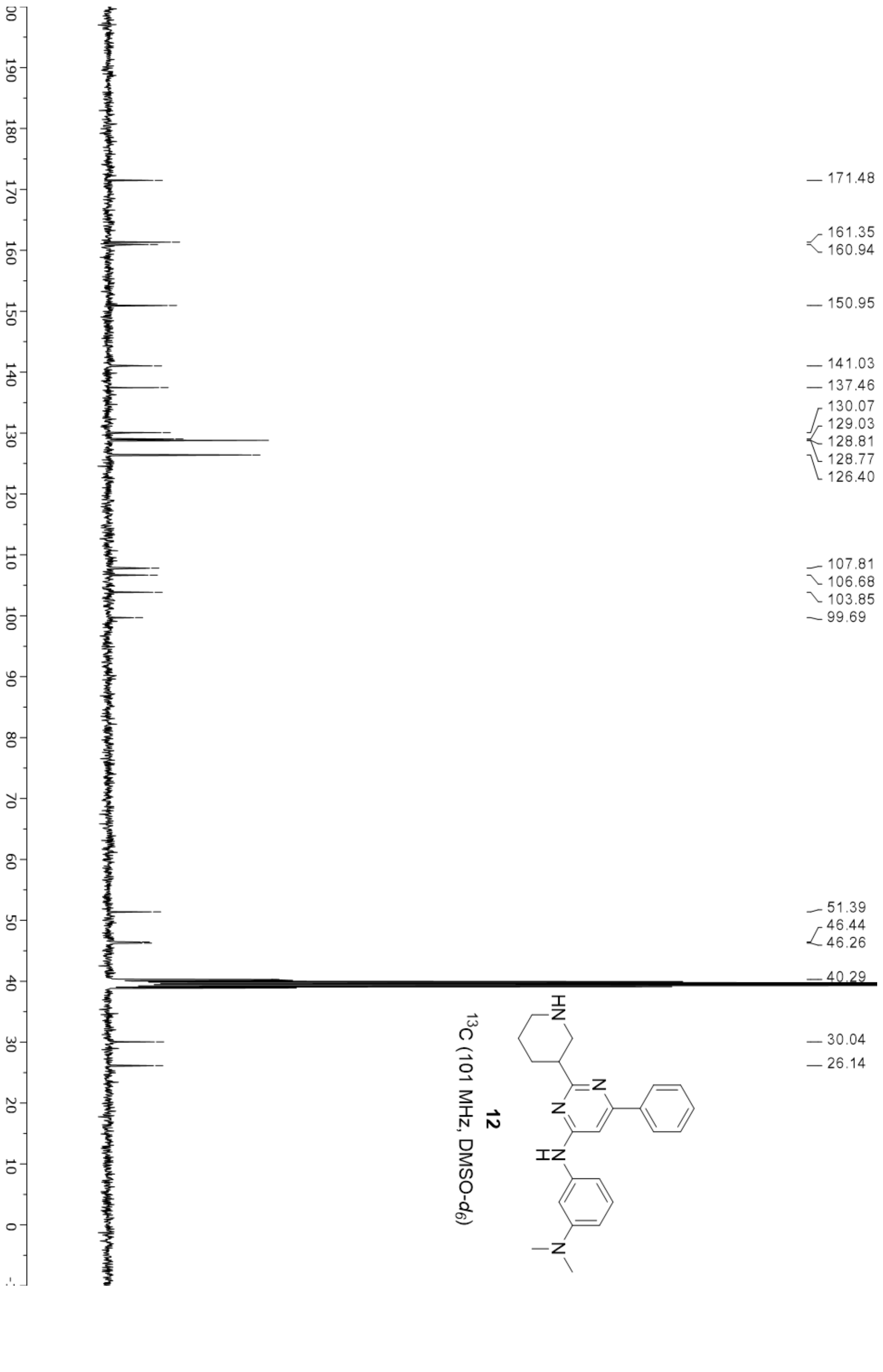

171.48
161.35
160.94
150.95
141.03
137.46
130.07
129.03
128.81
128.77
126.40
107.81
106.68
103.85
99.69
51.39
46.44
46.26
40.29
30.04
26.14
f1 (ppm)
HN
N
N
H
12
^{13}C (101 MHz, DMSO-d_6)

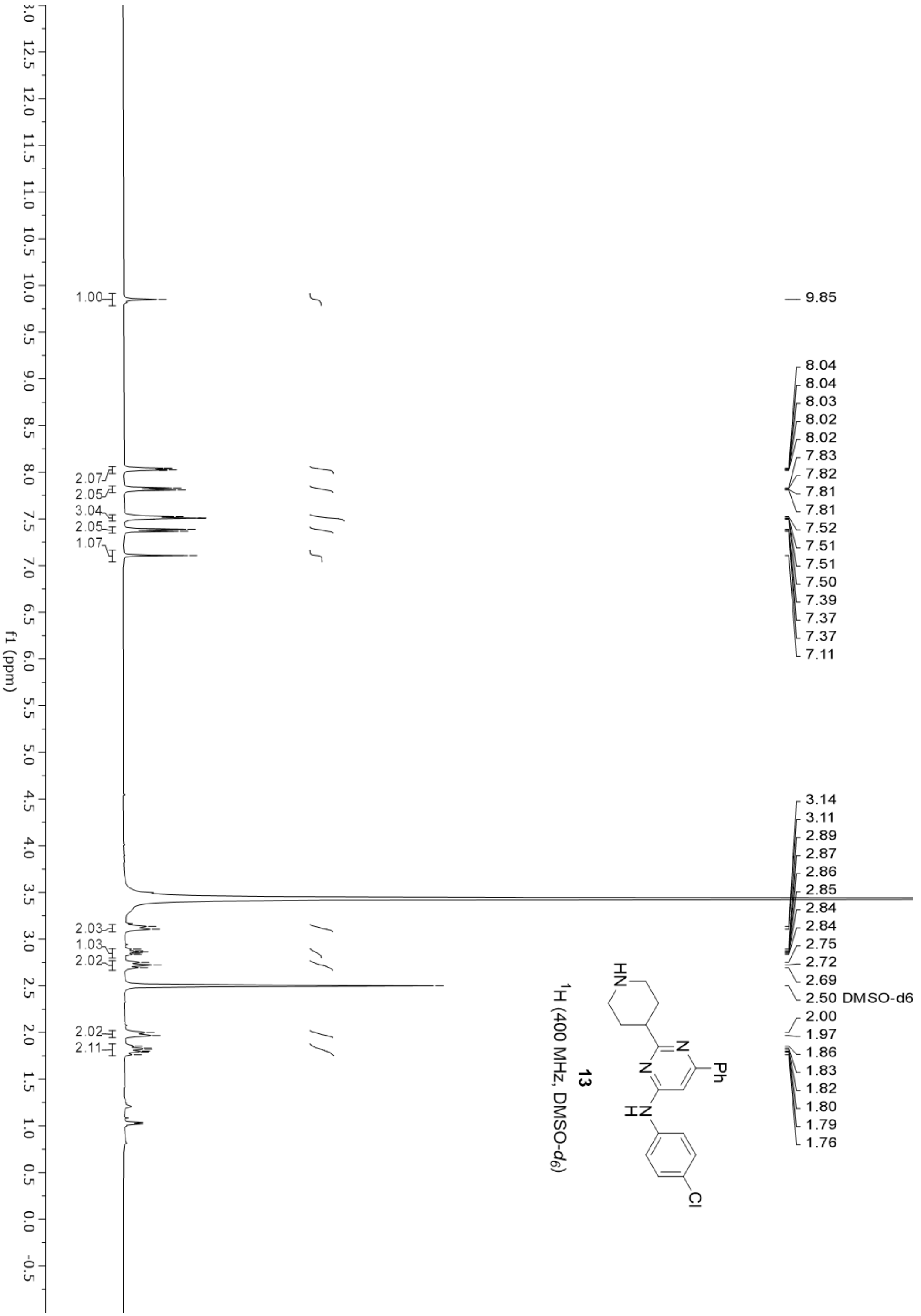
9.85
8.04
8.04
8.03
8.02
8.02
7.83
7.82
7.81
7.81
7.52
7.51
7.51
7.50
7.39
7.37
7.37
7.11
3.14
3.11
2.89
2.87
2.86
2.85
2.84
2.84
2.75
2.72
2.69
2.50 DMSO-d6
2.00
1.97
1.86
1.83
1.82
1.80
1.79
1.76
1.00
2.07
2.05
3.04
2.05
1.07
2.03
1.03
2.02
2.02
2.11
f1 (ppm)
HN
N
N
Ph
N
H
Cl
13
1H (400 MHz, DMSO-d6)

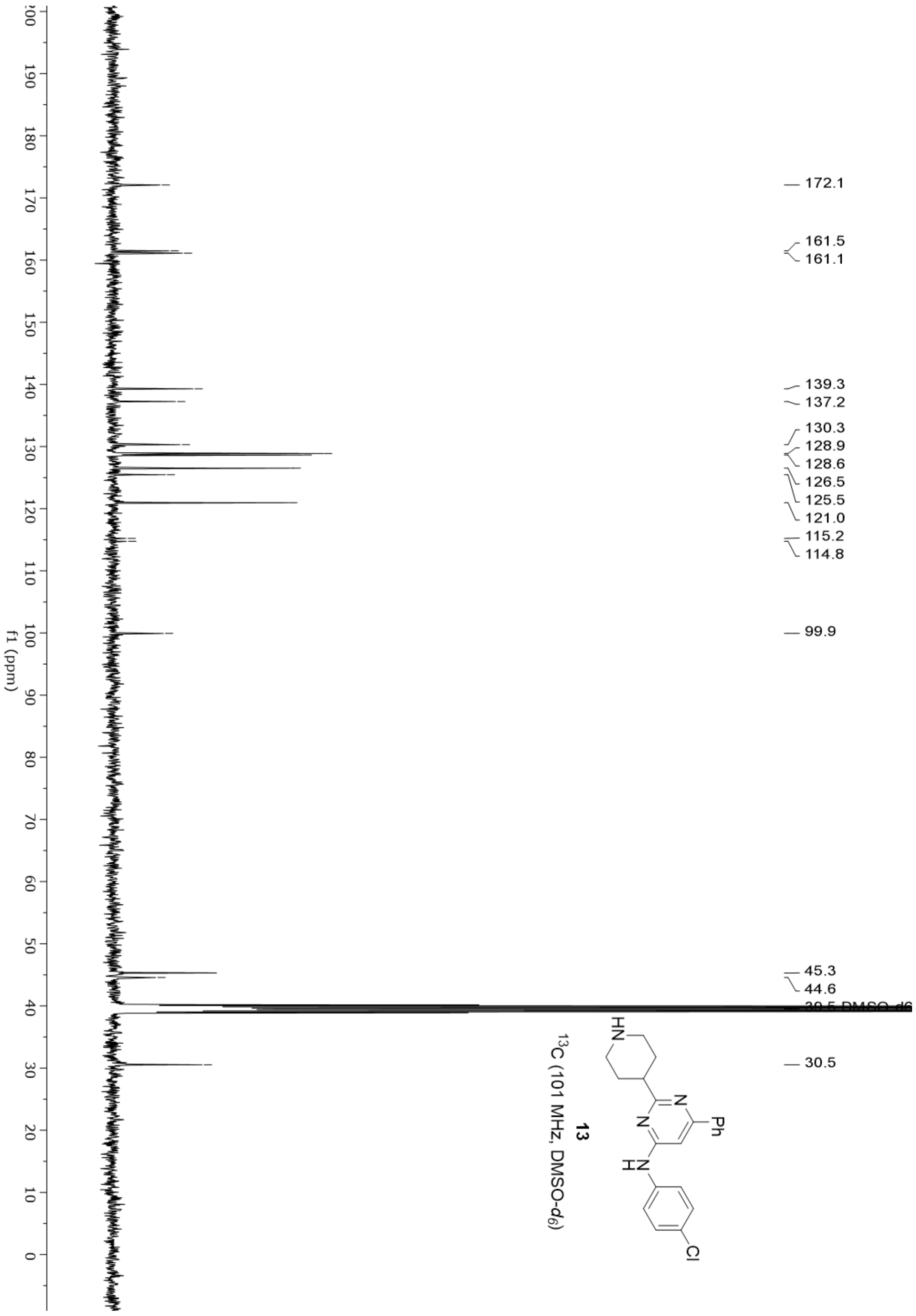
172.1
161.5
161.1
139.3
137.2
130.3
128.9
128.6
126.5
125.5
121.0
115.2
114.8
99.9
45.3
44.6
39.5 DMSO-d6
30.5
f1 (ppm)
13
^{13}C (101 MHz, DMSO-d_6)

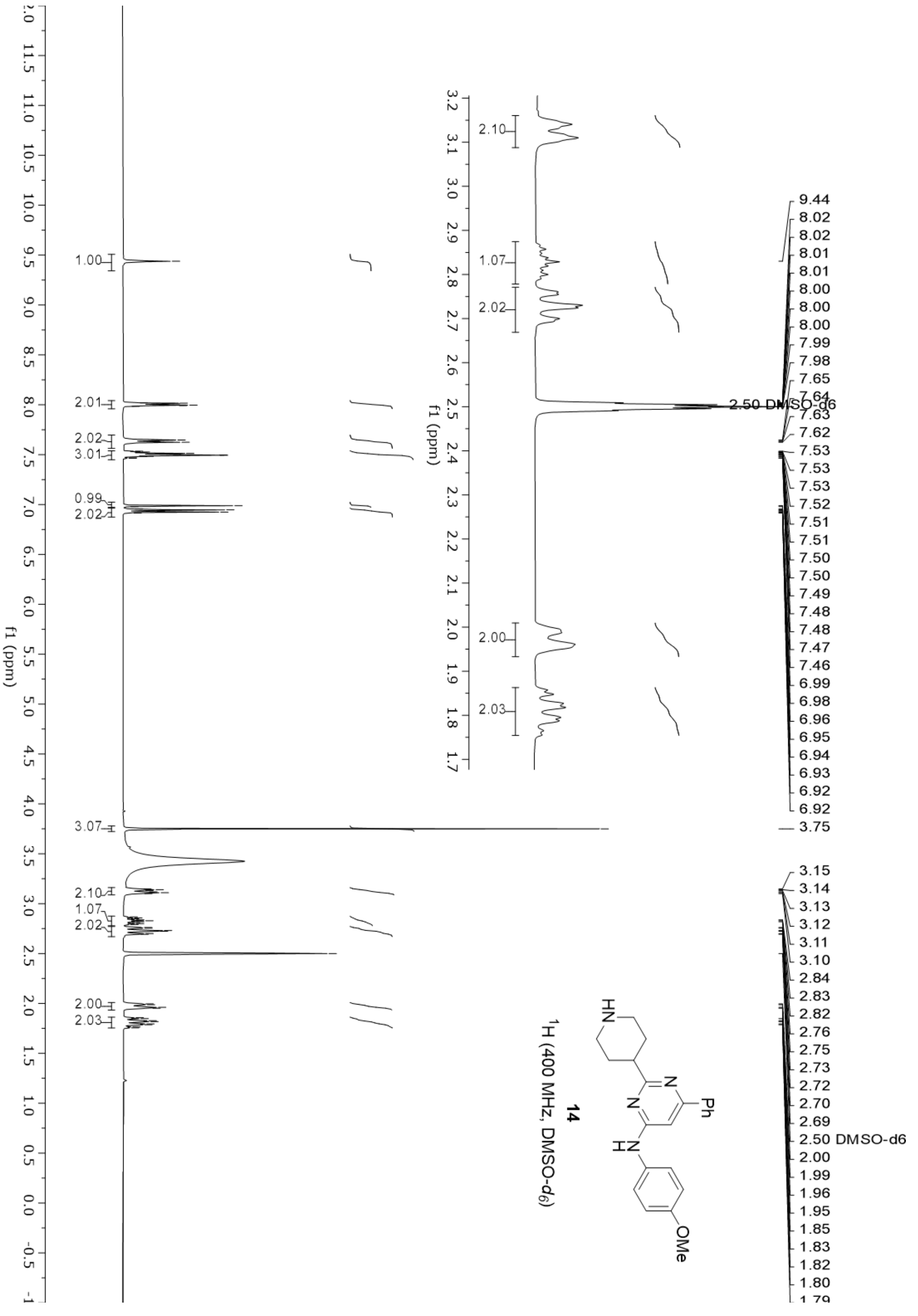
14
1H (400 MHz, DMSO-d6)
Ph
OMe
f1 (ppm)
2.50 DMSO-d6

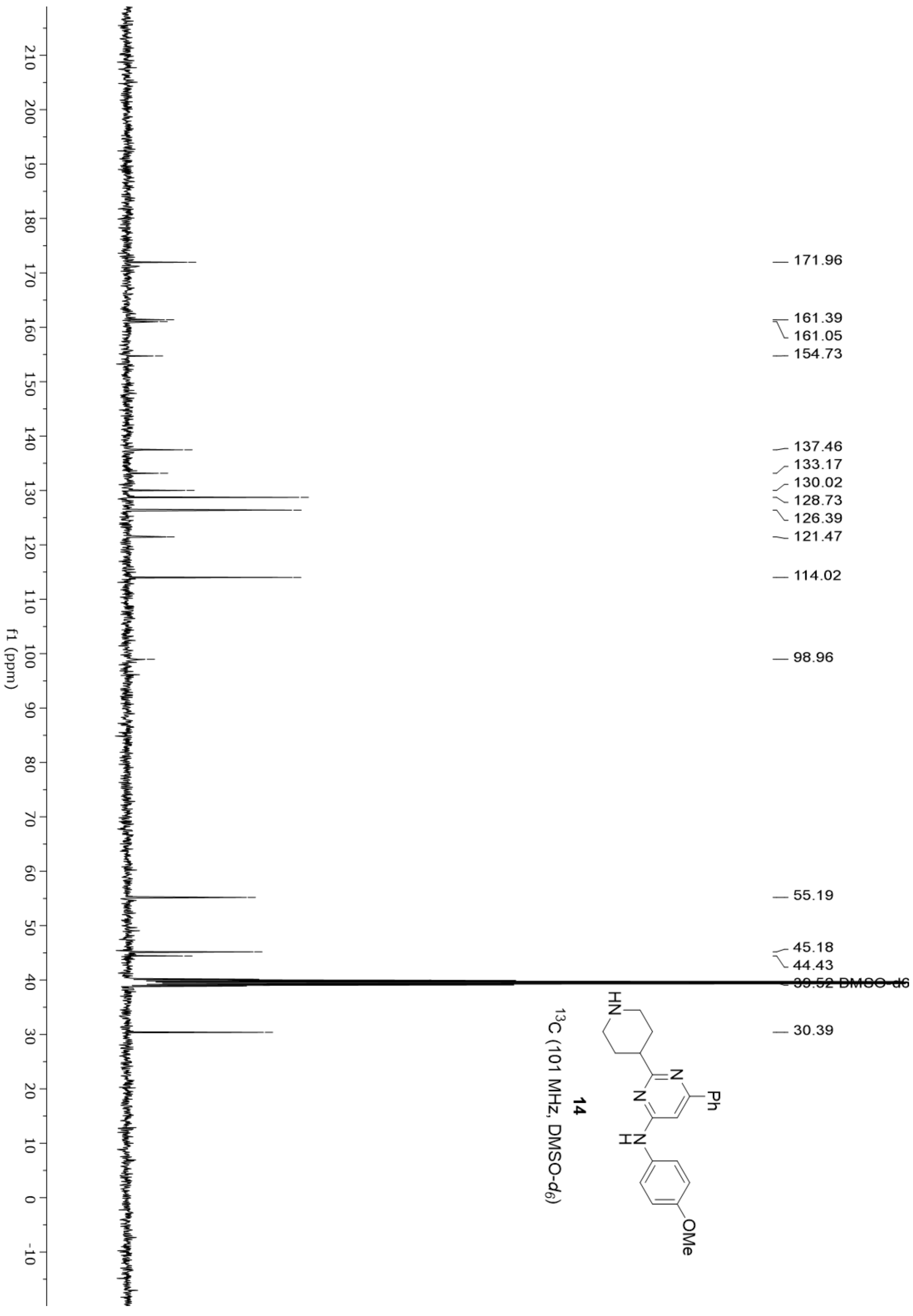

171.96
161.39
161.05
154.73
137.46
133.17
130.02
128.73
126.39
121.47
114.02
98.96
55.19
45.18
44.43
39.52 DMSO-d6
30.39
f1 (ppm)
HN
N
N
Ph
N
H
OMe
14
13C (101 MHz, DMSO-d6)

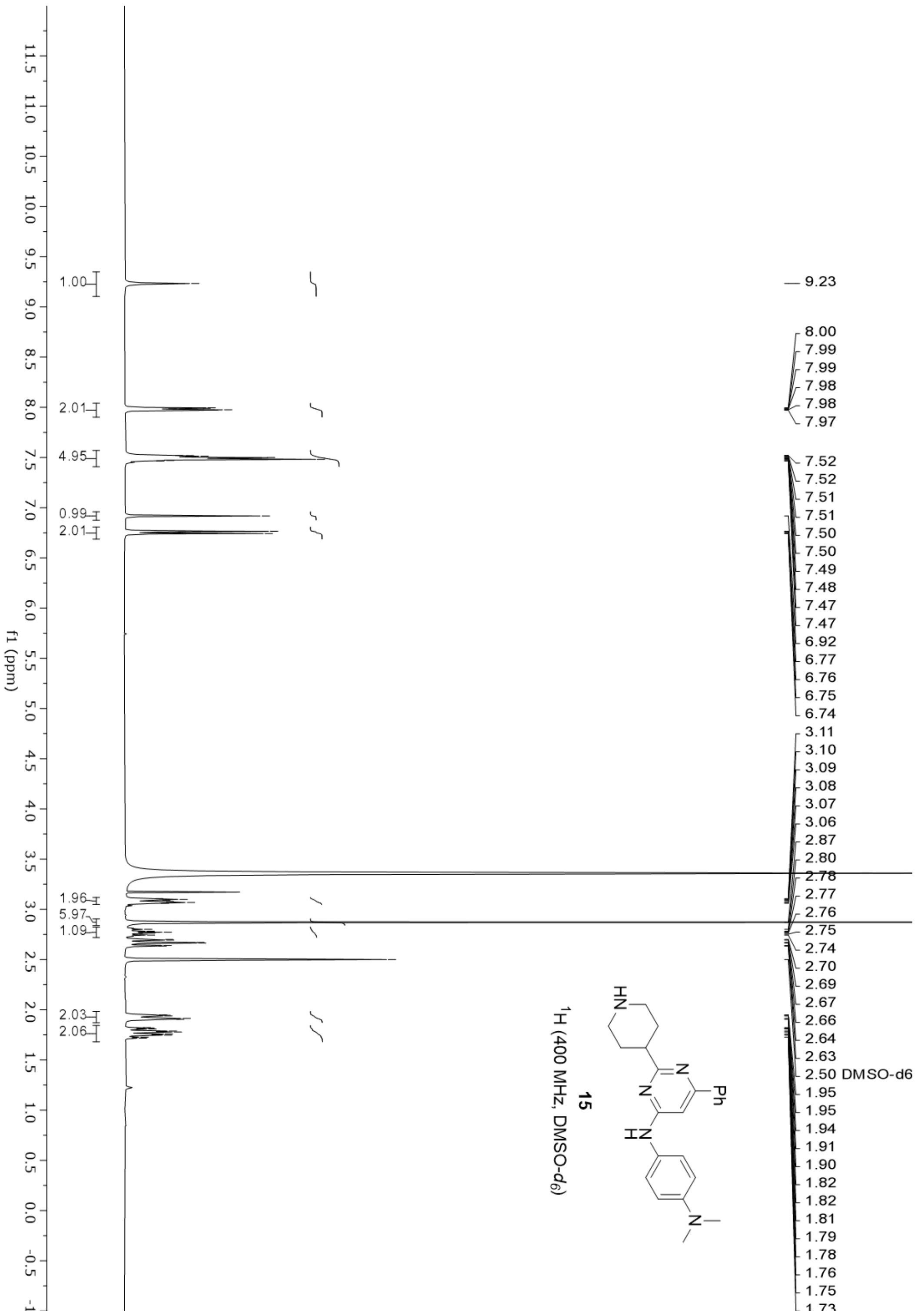
f1 (ppm)
15
1H (400 MHz, DMSO-d6)
2.50 DMSO-d6

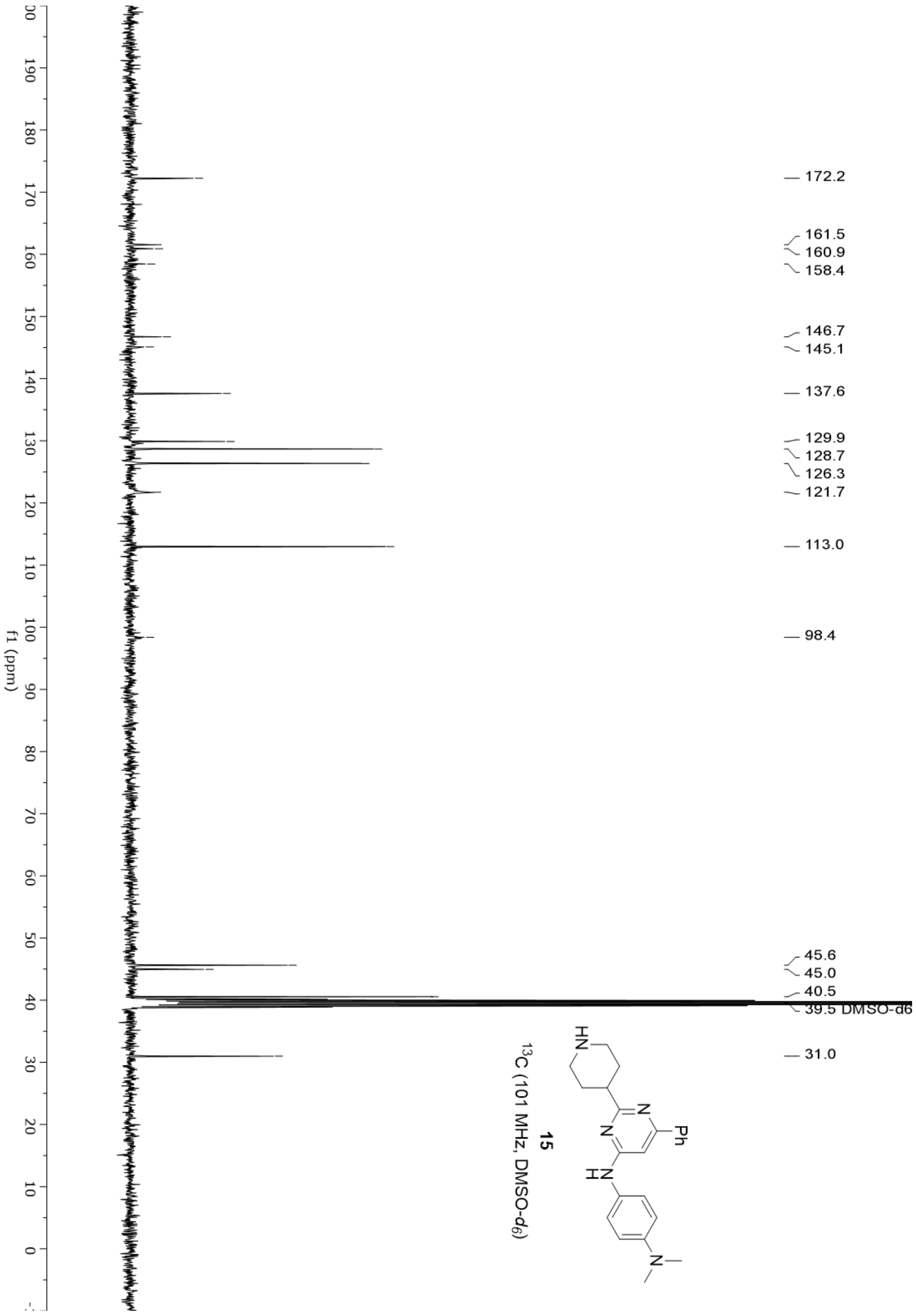

15

^{13}C (101 MHz, DMSO-d_6)

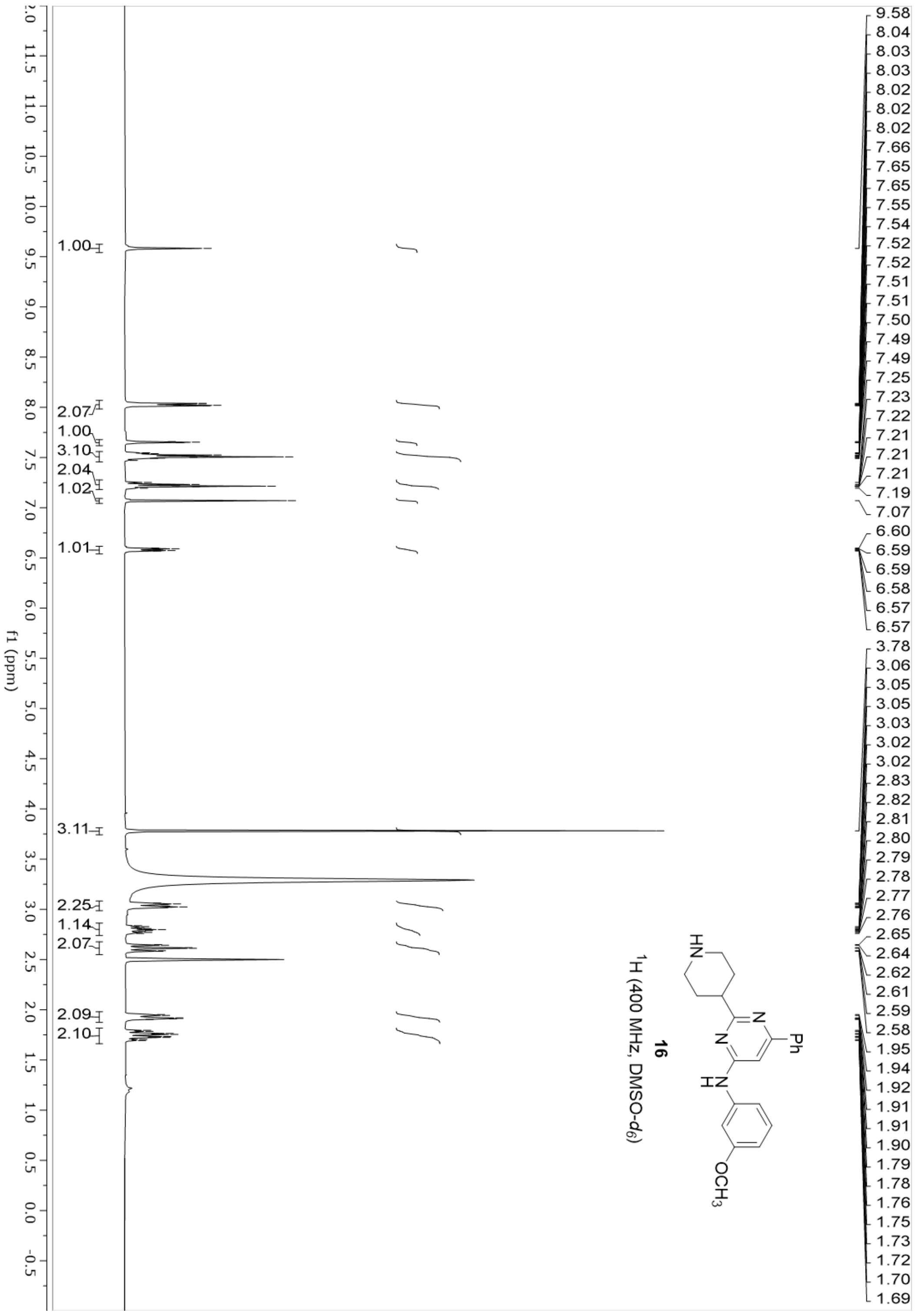
16
1H (400 MHz, DMSO-d6)
Ph
OCH3
f1 (ppm)

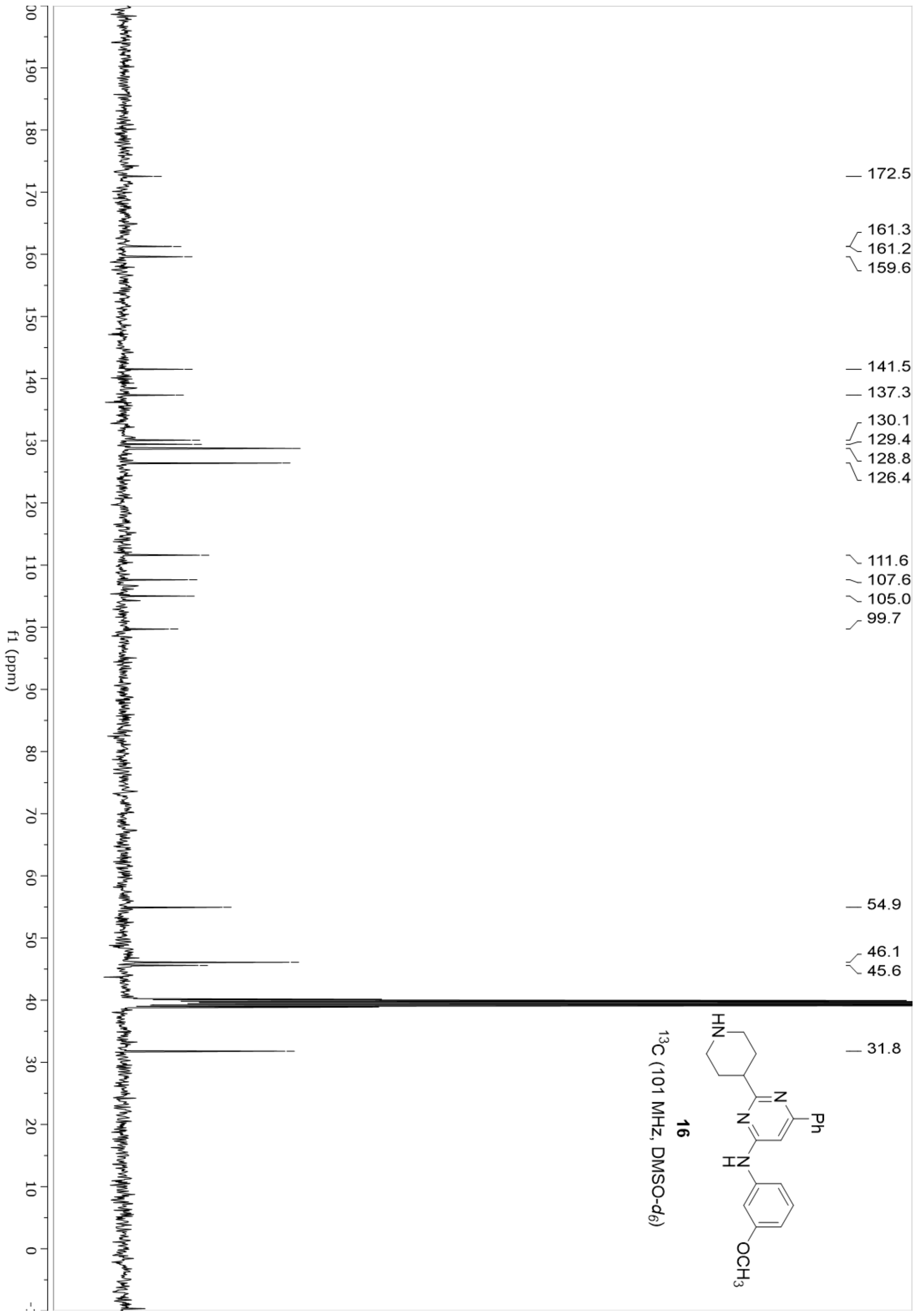
172.5
161.3
161.2
159.6
141.5
137.3
130.1
129.4
128.8
126.4
111.6
107.6
105.0
99.7
54.9
46.1
45.6
31.8
f1 (ppm)
HN
N
N
Ph
N
H
OCH3
16
13C (101 MHz, DMSO-d6)

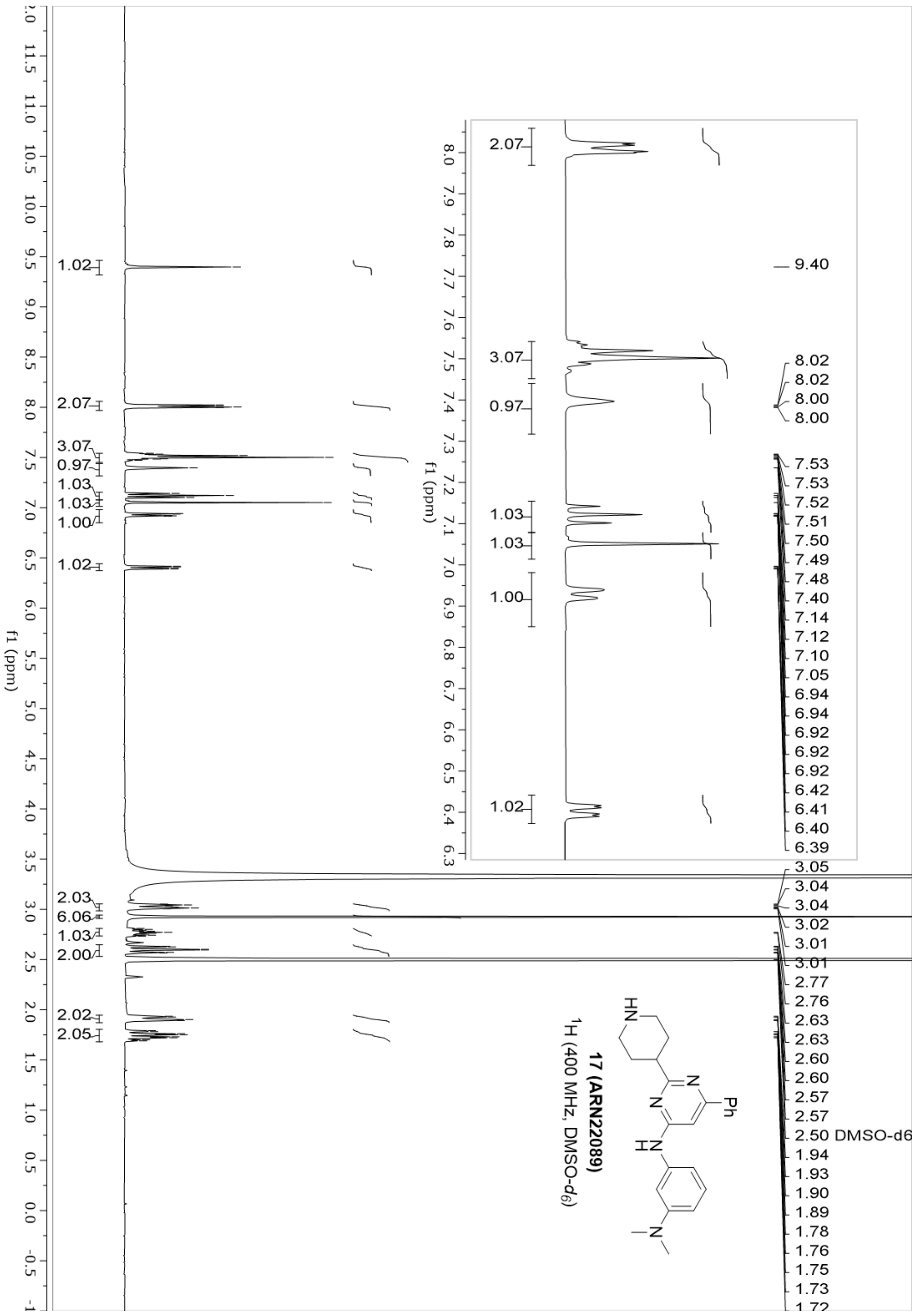
17 (ARN22089)
1H (400 MHz, DMSO-d6)

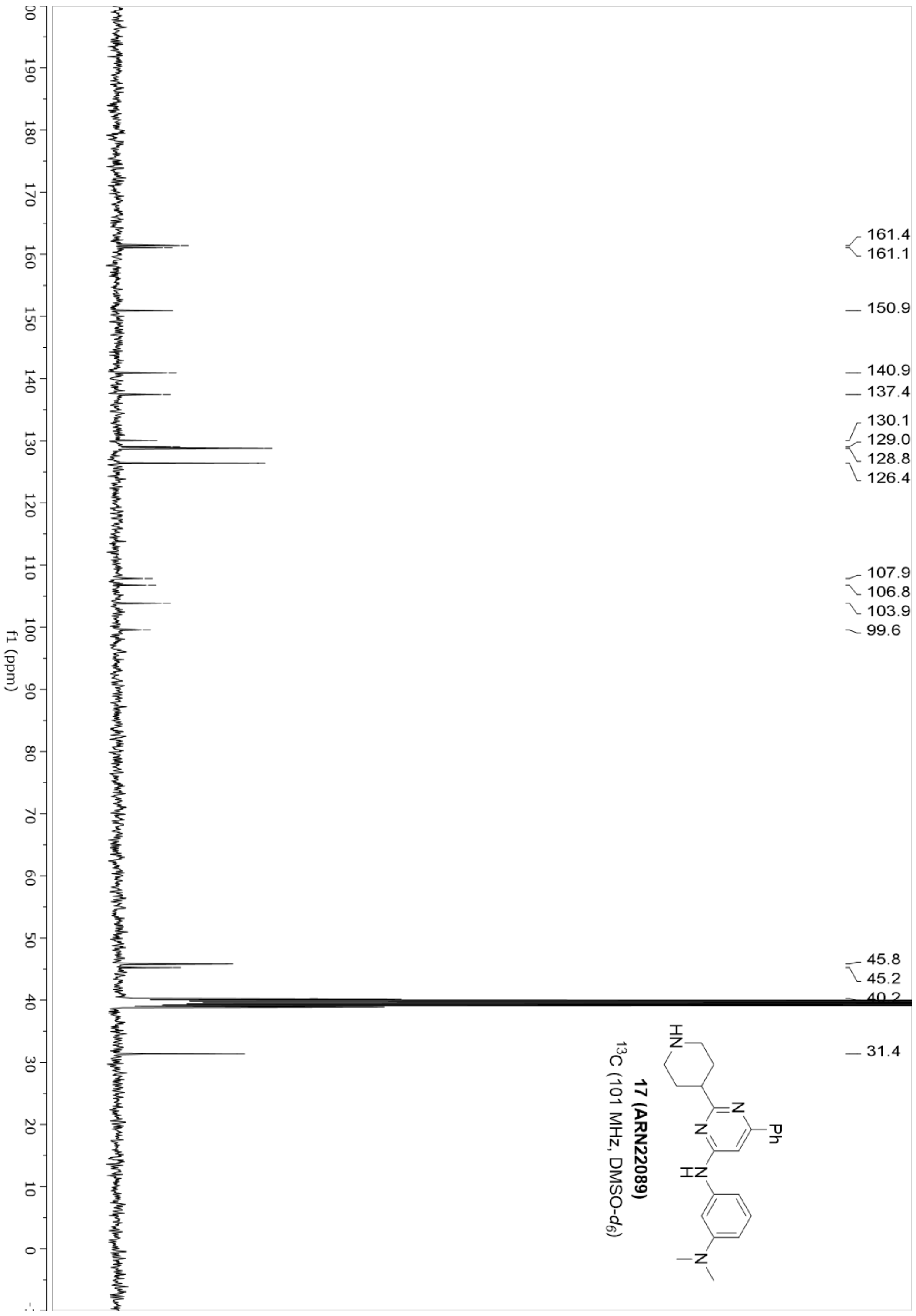
161.4
161.1
150.9
140.9
137.4
130.1
129.0
128.8
126.4
107.9
106.8
103.9
99.6
45.8
45.2
40.2
31.4
f1 (ppm)
17 (ARN22089)
^{13}C (101 MHz, DMSO-d_6)

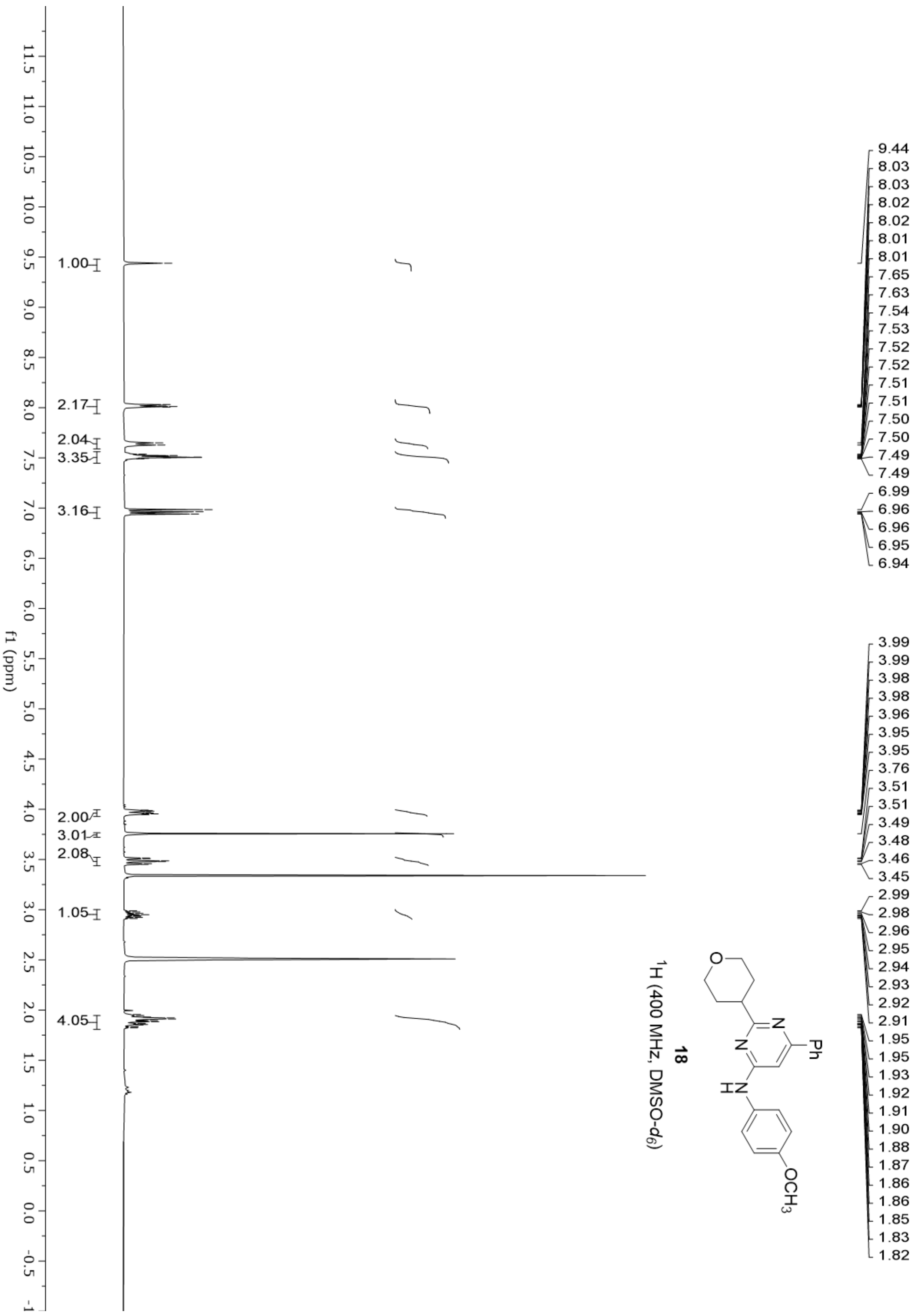

18
^{1}H (400 MHz, DMSO-d_6)
f1 (ppm)

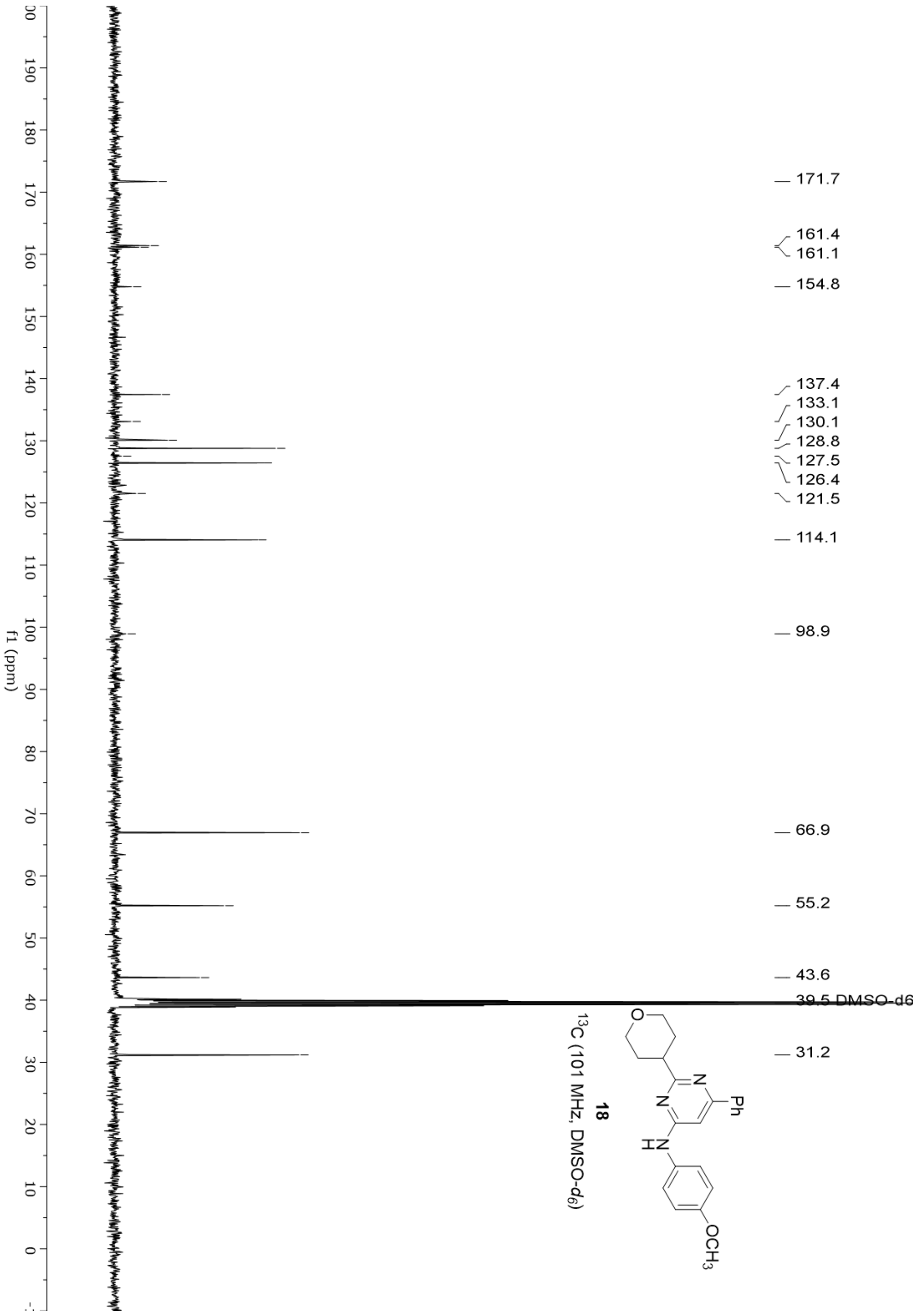
171.7
161.4
161.1
154.8
137.4
133.1
130.1
128.8
127.5
126.4
121.5
114.1
98.9
66.9
55.2
43.6
39.5 DMSO-d6
31.2
f1 (ppm)
Ph
N
N
O
N
H
OCH3
18
13C (101 MHz, DMSO-d6)

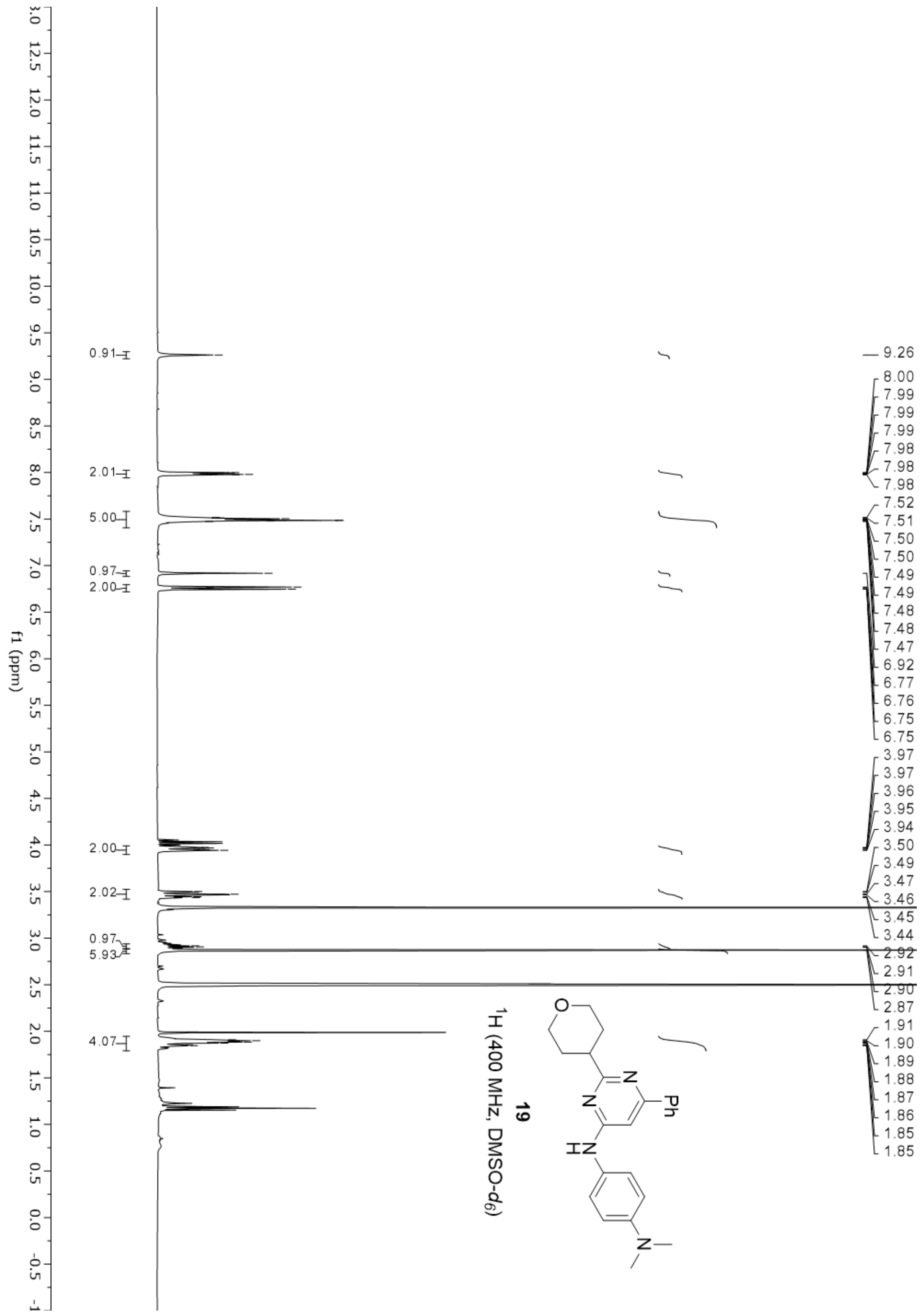

19
1H (400 MHz, DMSO-d6)
f1 (ppm)
Ph

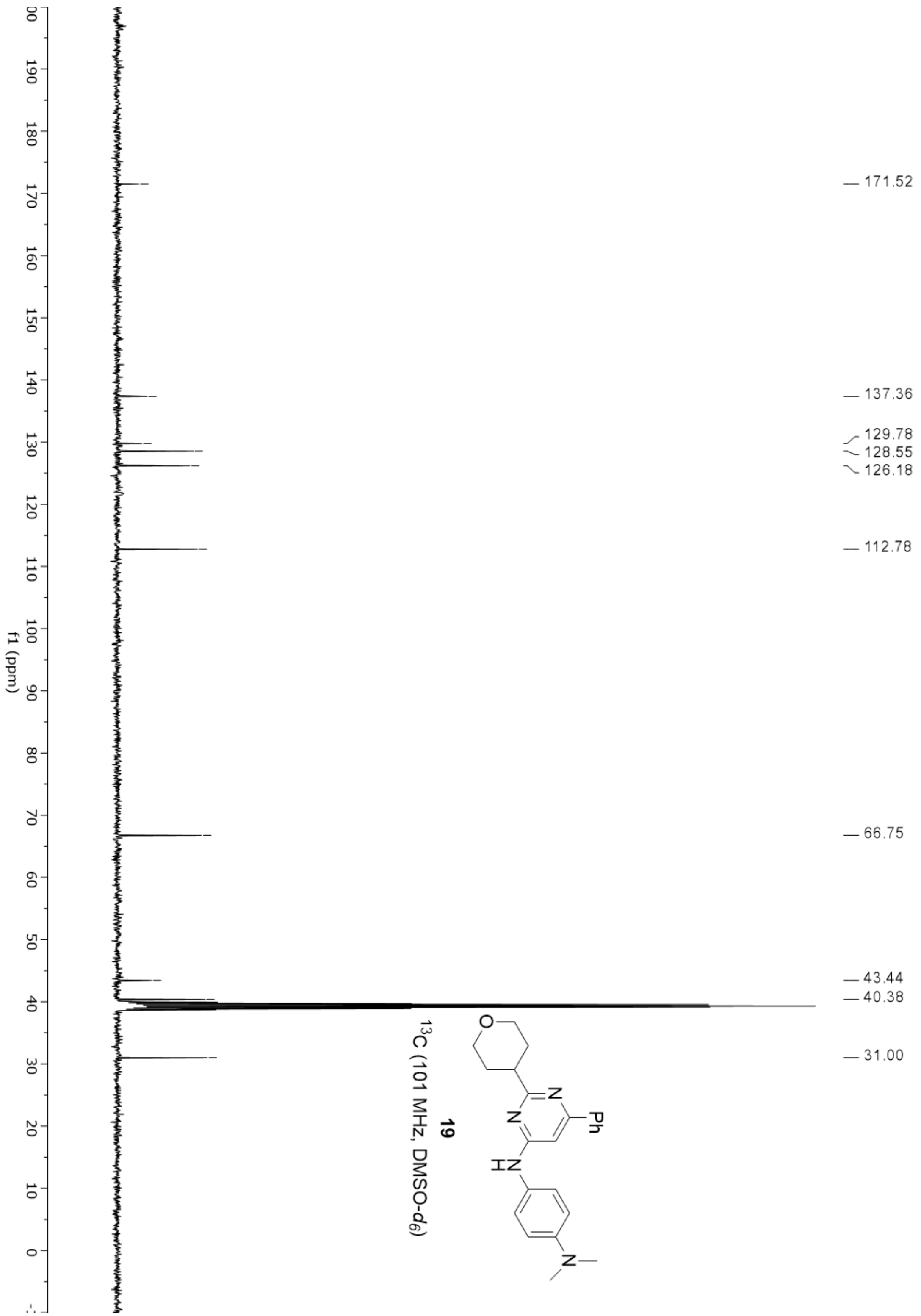

171.52
137.36
129.78
128.55
126.18
112.78
66.75
43.44
40.38
31.00
f1 (ppm)
Ph
N
N
H
O
19
13C (101 MHz, DMSO-d6)

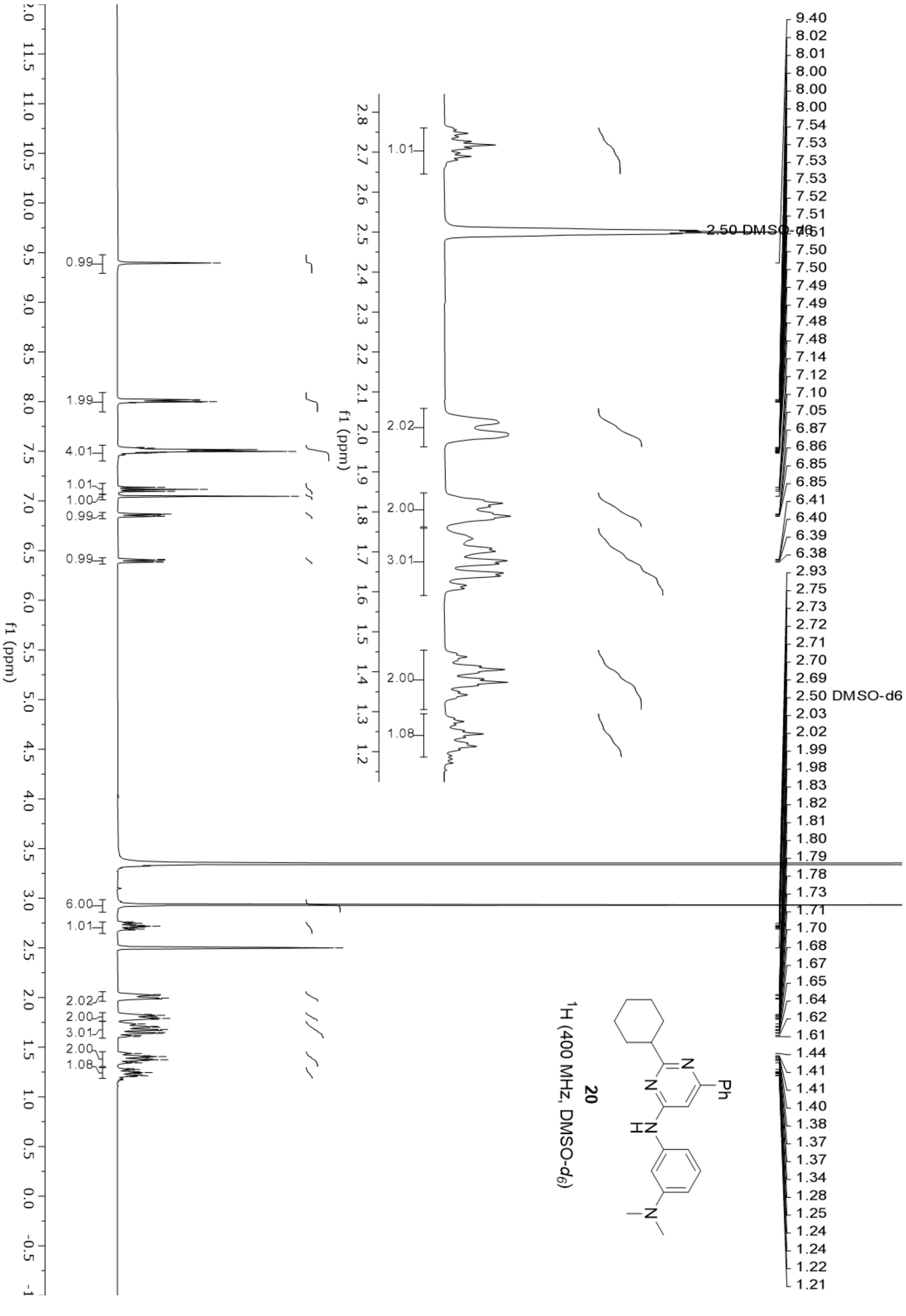
9.40
8.02
8.01
8.00
8.00
8.00
7.54
7.53
7.53
7.53
7.52
7.51
7.51
7.50
7.50
7.49
7.49
7.48
7.48
7.14
7.12
7.10
7.05
6.87
6.86
6.85
6.85
6.41
6.40
6.39
6.38
2.93
2.75
2.73
2.72
2.71
2.70
2.69
2.50 DMSO-d6
2.03
2.02
1.99
1.98
1.83
1.82
1.81
1.80
1.79
1.78
1.73
1.71
1.70
1.68
1.67
1.65
1.64
1.62
1.61
1.44
1.41
1.41
1.40
1.38
1.37
1.37
1.34
1.28
1.25
1.24
1.24
1.22
1.21
2.50 DMSO-d6
Ph
N
N
N
H
N
20
1H (400 MHz, DMSO-d6)
f1 (ppm)
f1 (ppm)

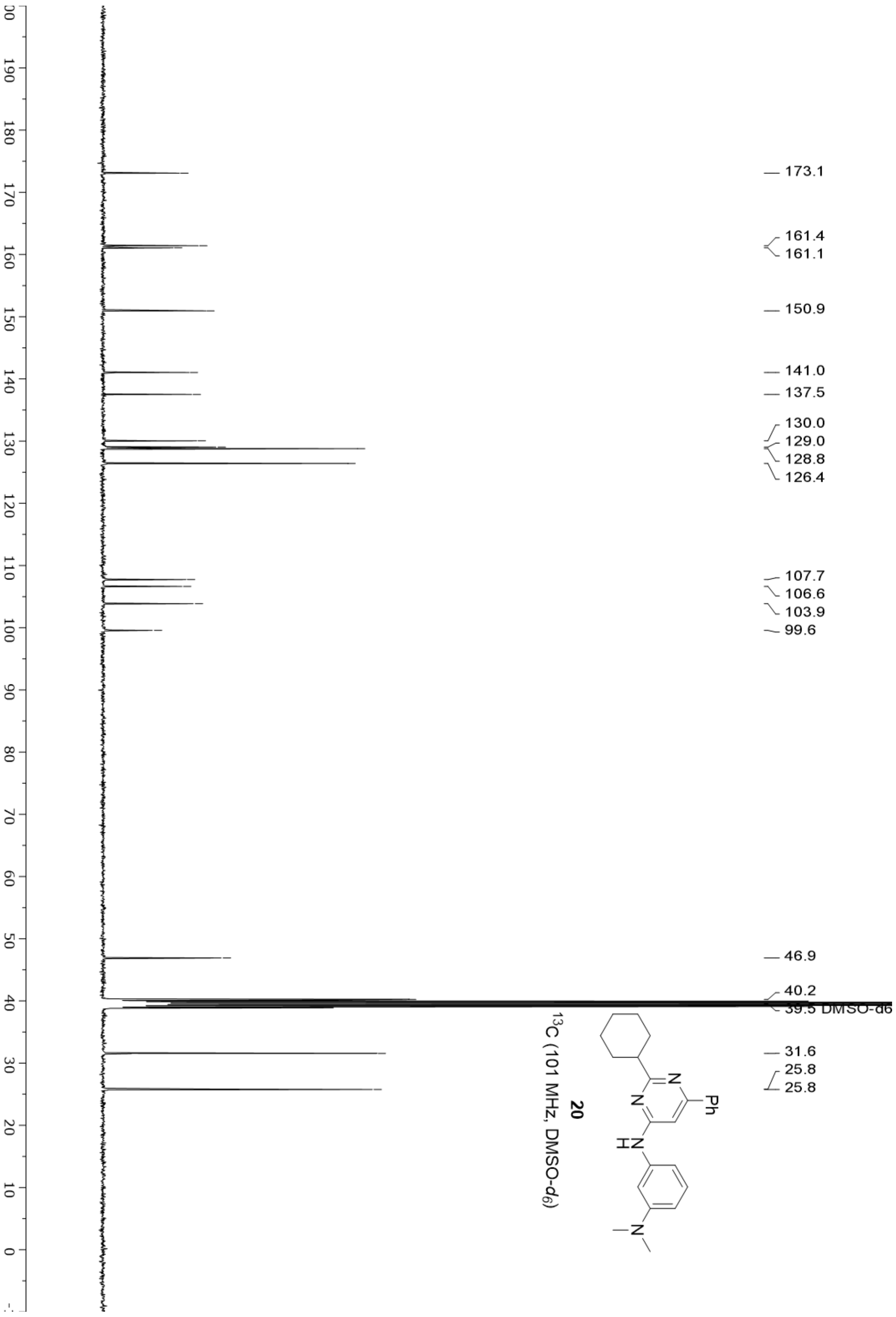
173.1
161.4
161.1
150.9
141.0
137.5
130.0
129.0
128.8
126.4
107.7
106.6
103.9
99.6
46.9
40.2
39.5 DMSO-d6
31.6
25.8
25.8
Ph
N
N
N
H
20
13C (101 MHz, DMSO-d6)
f1 (ppm)

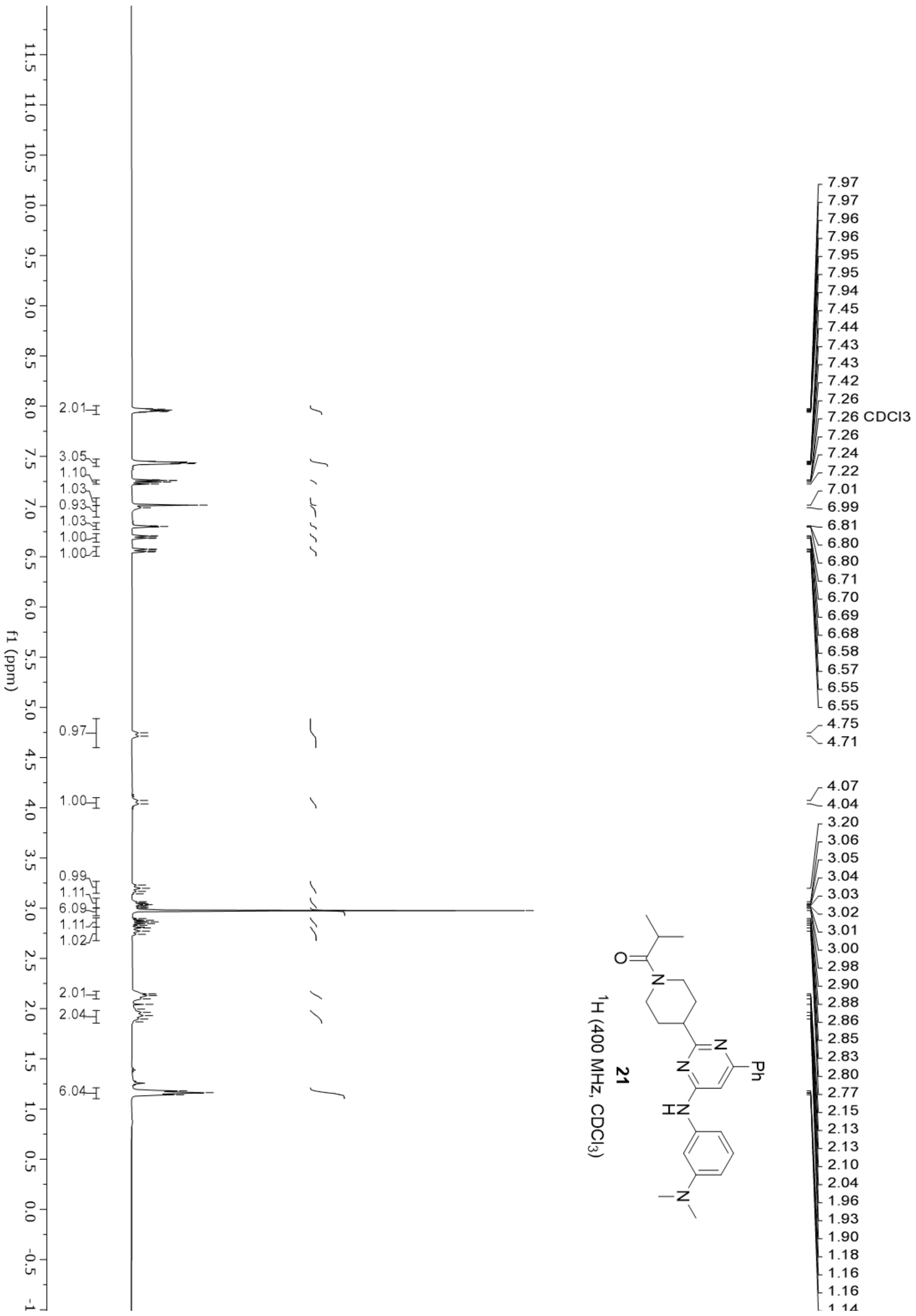

21

^1H (400 MHz, $CDCl_3$)

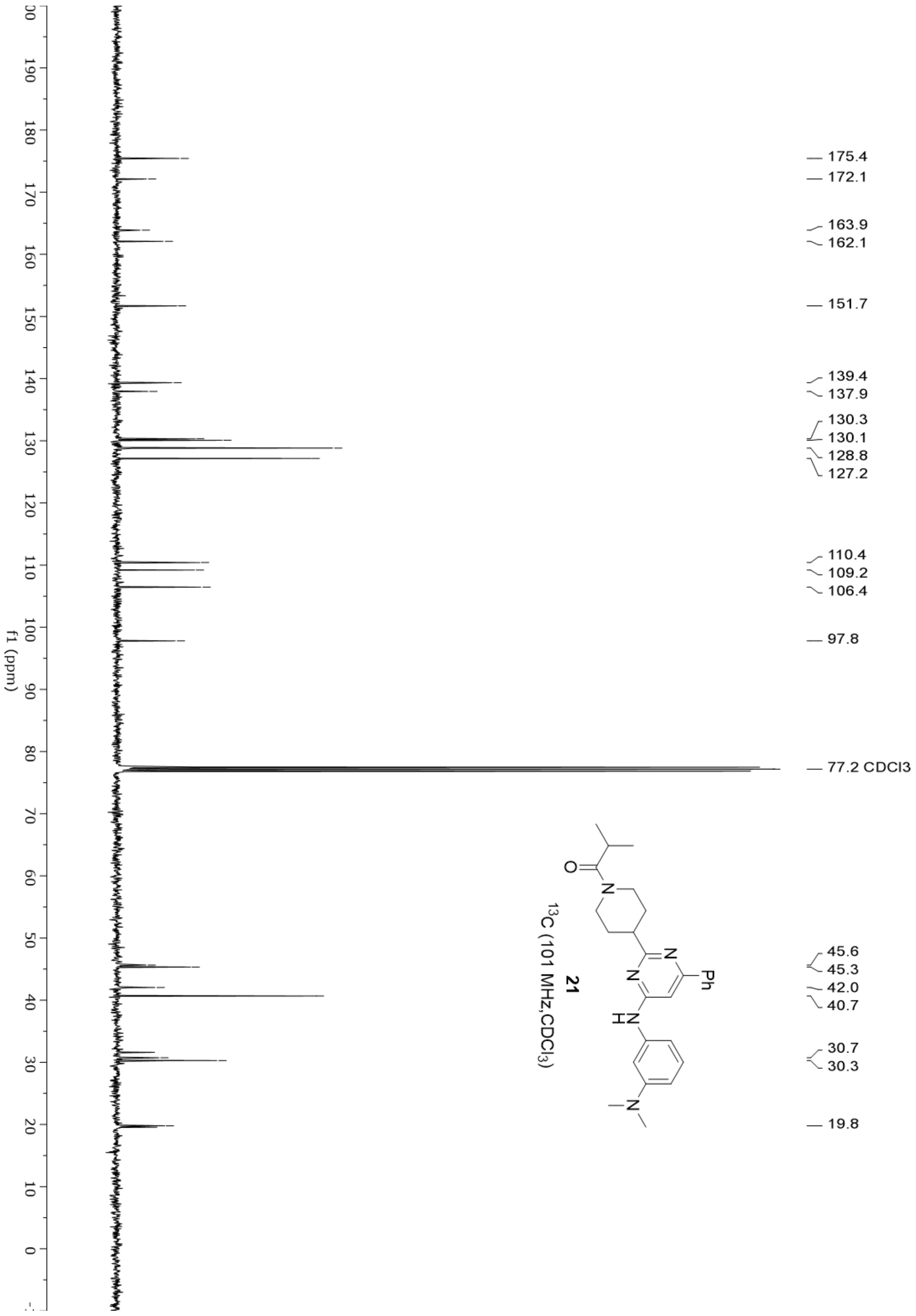
175.4
172.1
163.9
162.1
151.7
139.4
137.9
130.3
130.1
128.8
127.2
110.4
109.2
106.4
97.8
77.2 CDCl3
45.6
45.3
42.0
40.7
30.7
30.3
19.8
f1 (ppm)
21
13C (101 MHz,CDCl3)
Ph
N
H

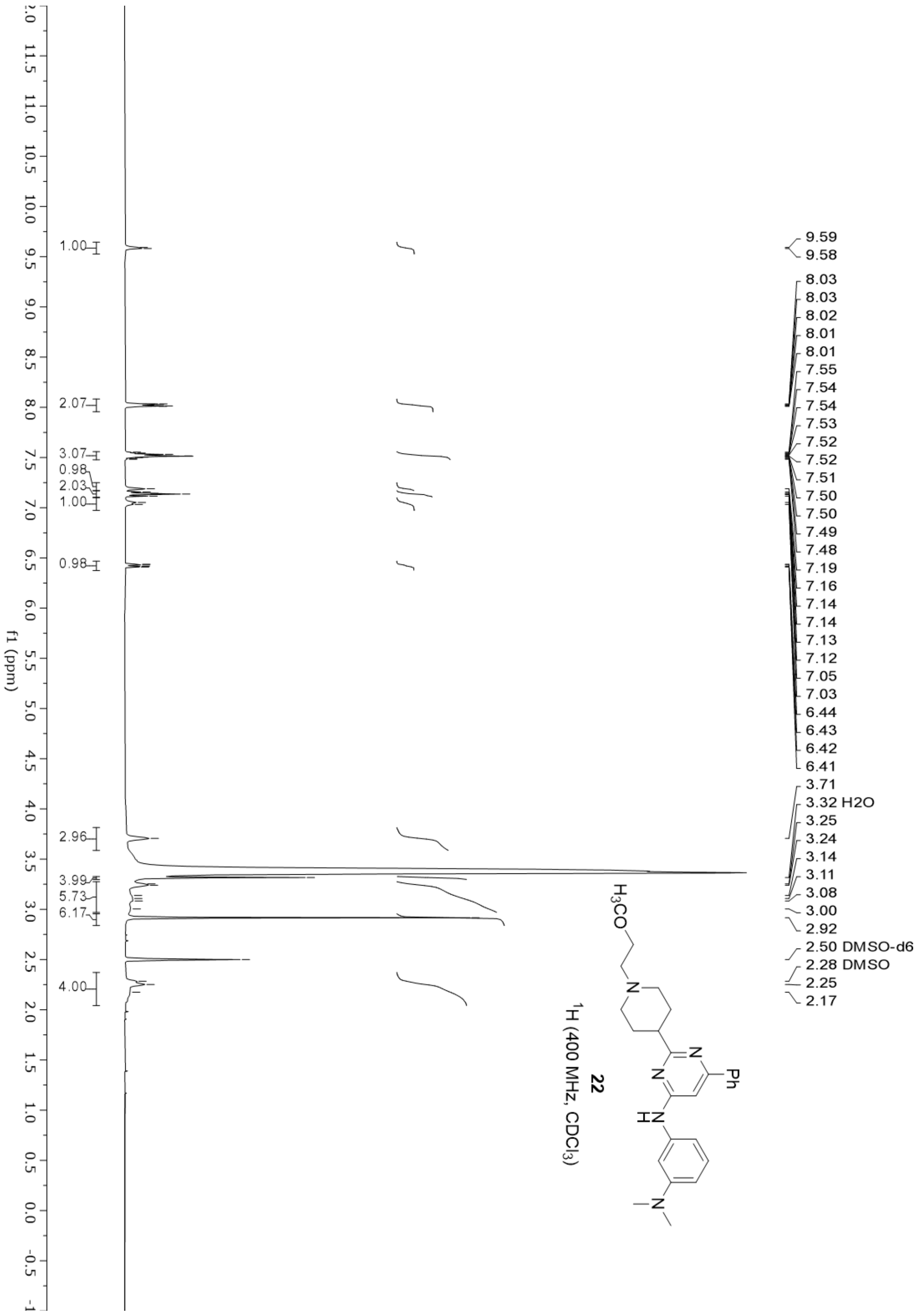

22
^{1}H (400 MHz, $CDCl_3$)

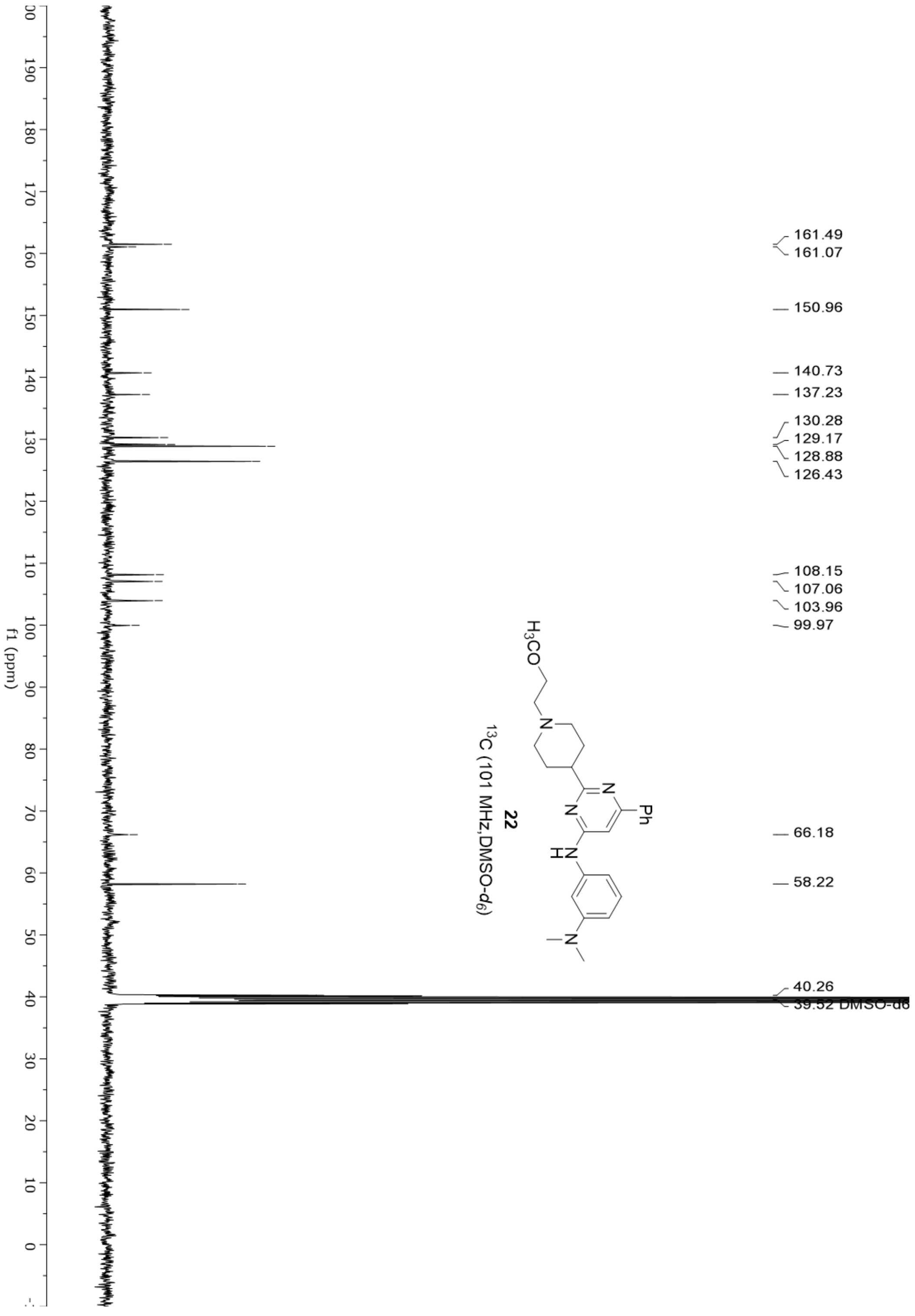
161.49
161.07
150.96
140.73
137.23
130.28
129.17
128.88
126.43
108.15
107.06
103.96
99.97
66.18
58.22
40.26
39.52 DMSO-d6
f1 (ppm)
H3CO
N
N
N
Ph
H
N
22
13C (101 MHz,DMSO-d6)

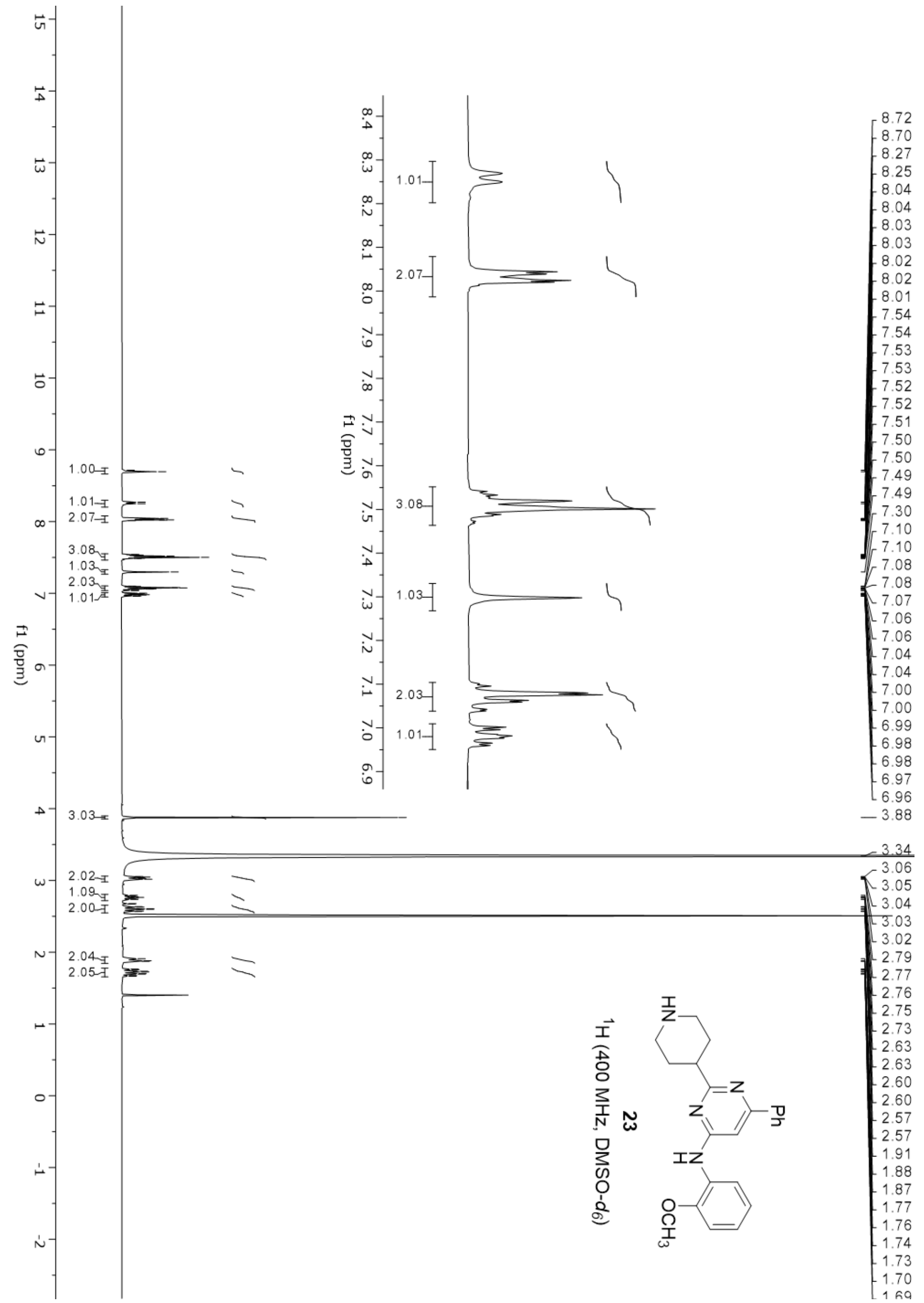

23

^{1}H (400 MHz, DMSO-d_6)

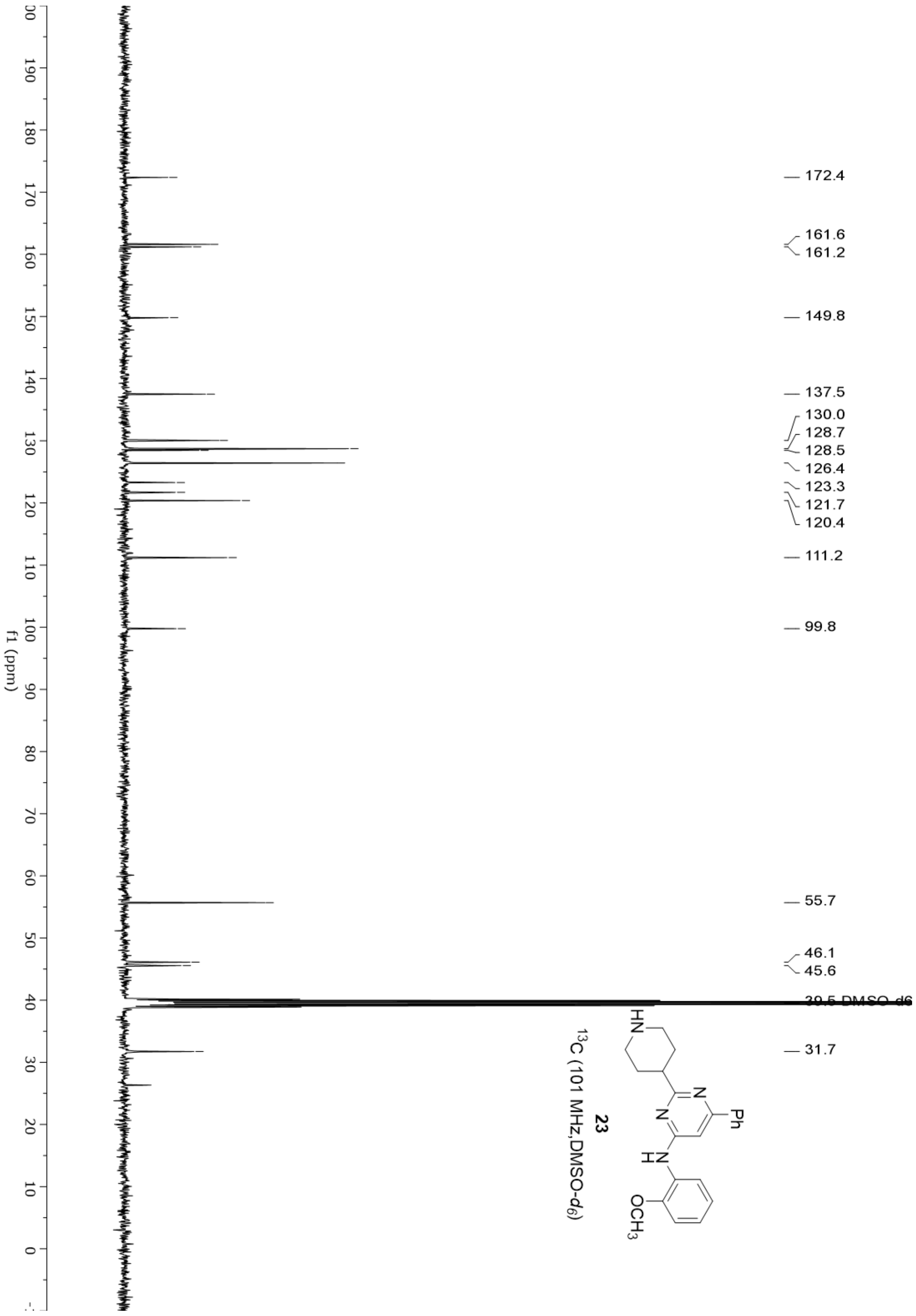
172.4
161.6
161.2
149.8
137.5
130.0
128.7
128.5
126.4
123.3
121.7
120.4
111.2
99.8
55.7
46.1
45.6
39.5 DMSO-d6
31.7
f1 (ppm)
HN
N
N
Ph
N
H
OCH3
23
13C (101 MHz,DMSO-d6)

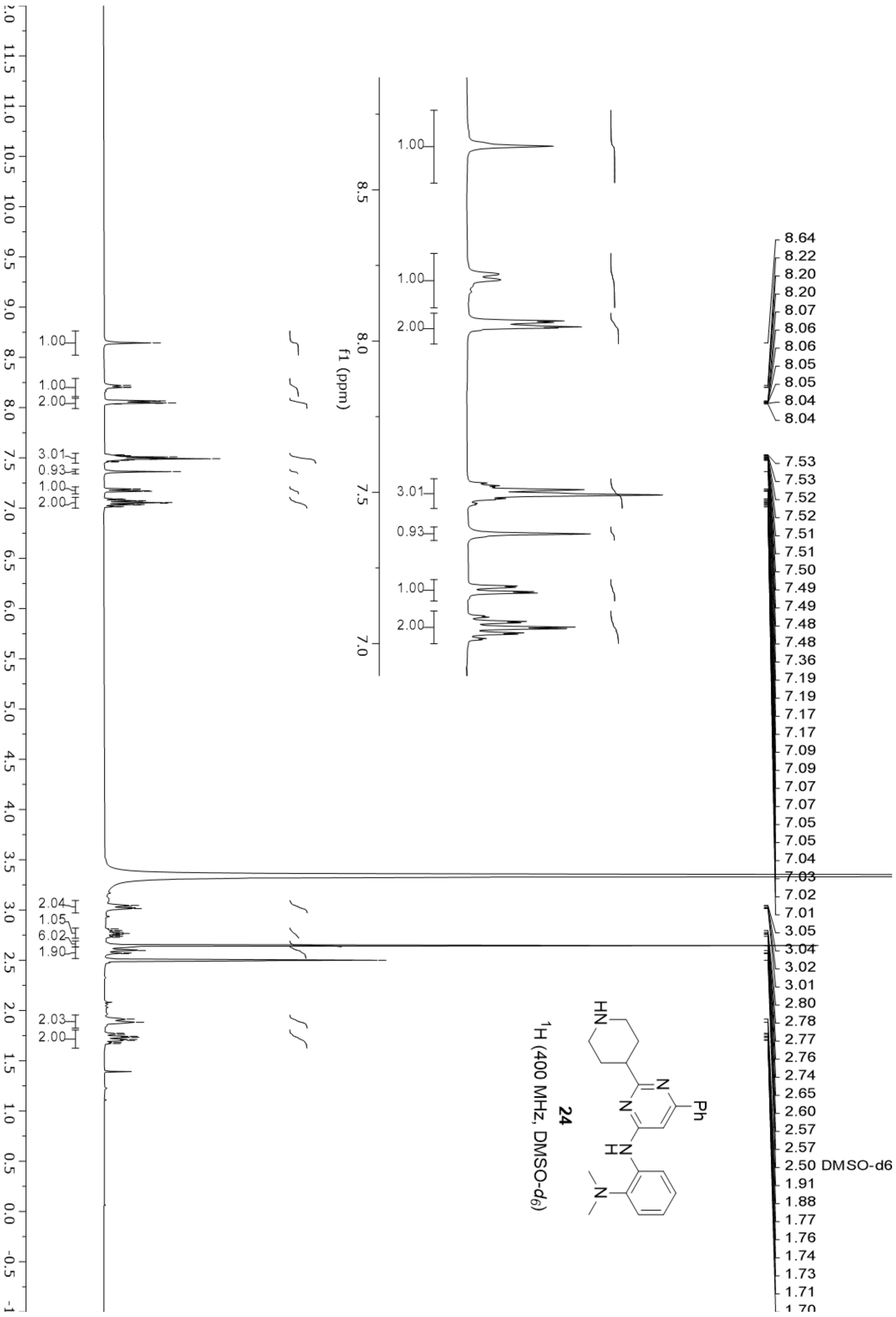
24
^{1}H (400 MHz, DMSO-d_6)

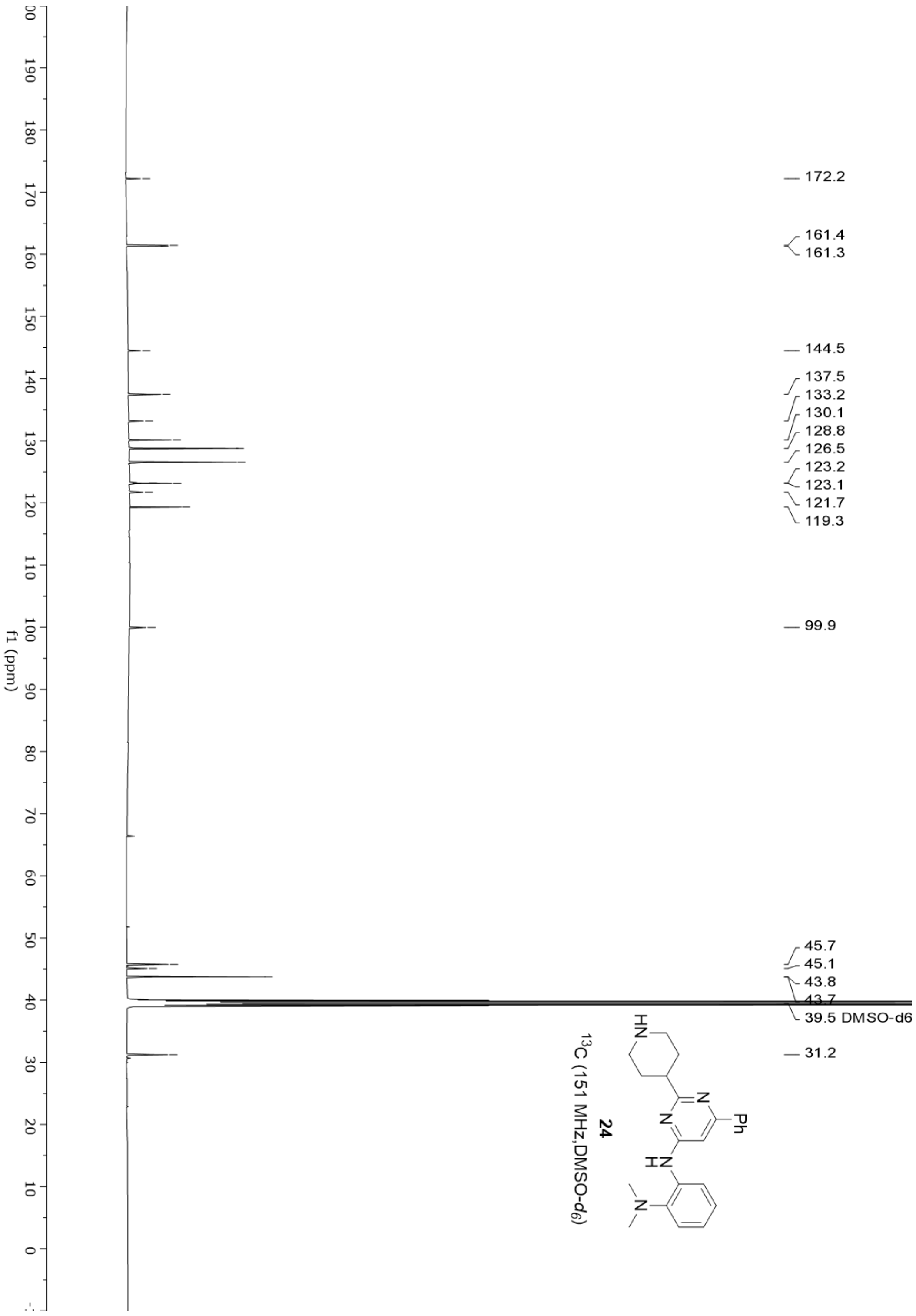
172.2
161.4
161.3
144.5
137.5
133.2
130.1
128.8
126.5
123.2
123.1
121.7
119.3
99.9
45.7
45.1
43.8
43.7
39.5 DMSO-d6
31.2
f1 (ppm)
24
^{13}C (151 MHz,DMSO-d_6)

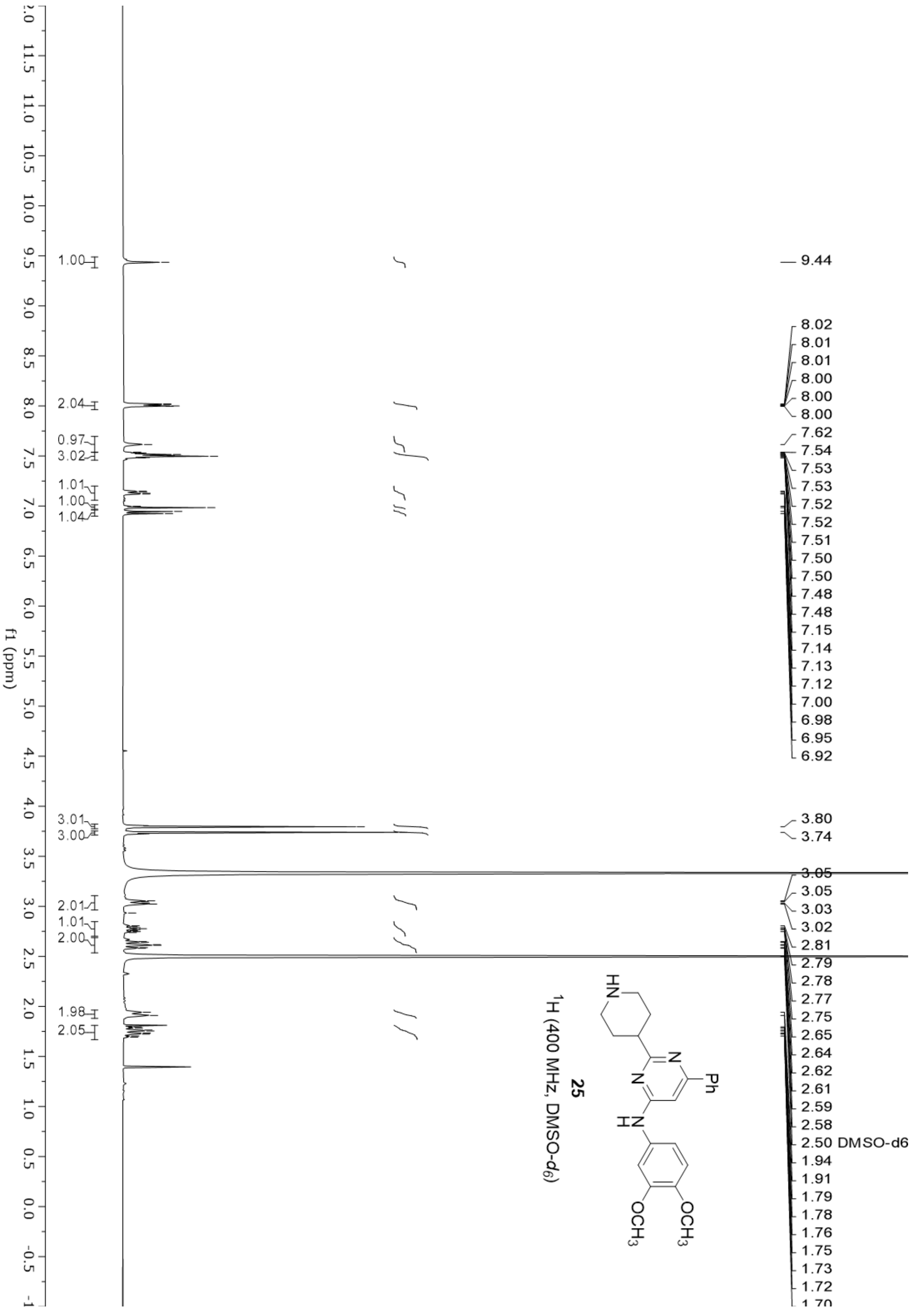

25
¹H (400 MHz, DMSO-d6)
HN
N
N
Ph
H
OCH3
OCH3
f1 (ppm)
9.44
8.02
8.01
8.01
8.00
8.00
8.00
7.62
7.54
7.53
7.53
7.52
7.52
7.51
7.50
7.50
7.48
7.48
7.15
7.14
7.13
7.12
7.00
6.98
6.95
6.92
3.80
3.74
3.05
3.05
3.03
3.02
2.81
2.79
2.78
2.77
2.75
2.65
2.64
2.62
2.61
2.59
2.58
2.50 DMSO-d6
1.94
1.91
1.79
1.78
1.76
1.75
1.73
1.72
1.00
2.04
0.97
3.02
1.01
1.00
1.04
3.01
3.00
2.01
1.01
2.00
1.98
2.05

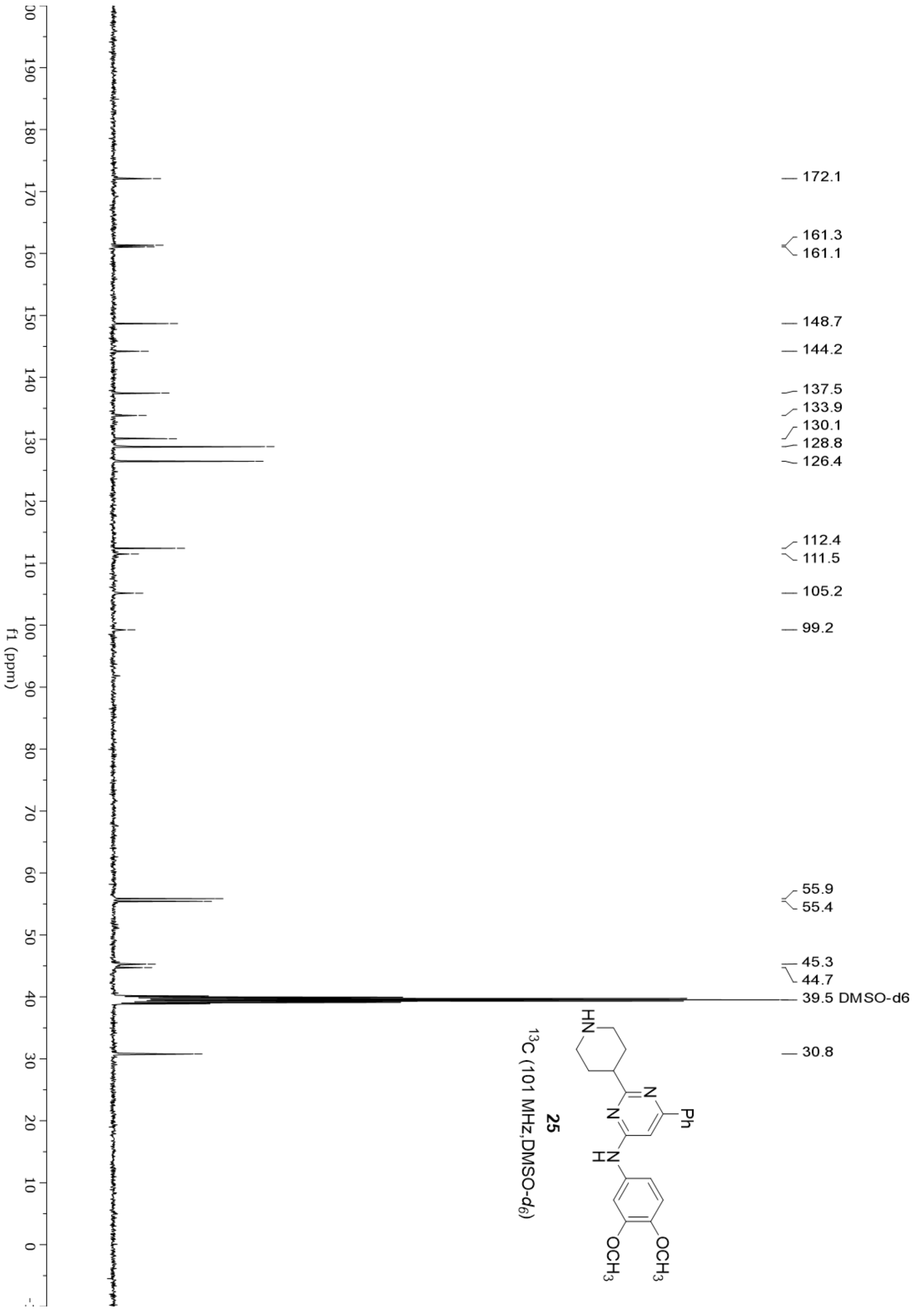

172.1
161.3
161.1
148.7
144.2
137.5
133.9
130.1
128.8
126.4
112.4
111.5
105.2
99.2
55.9
55.4
45.3
44.7
39.5 DMSO-d6
30.8
f1 (ppm)
HN
N
N
Ph
N
H
OCH3
OCH3
25
13C (101 MHz,DMSO-d6)

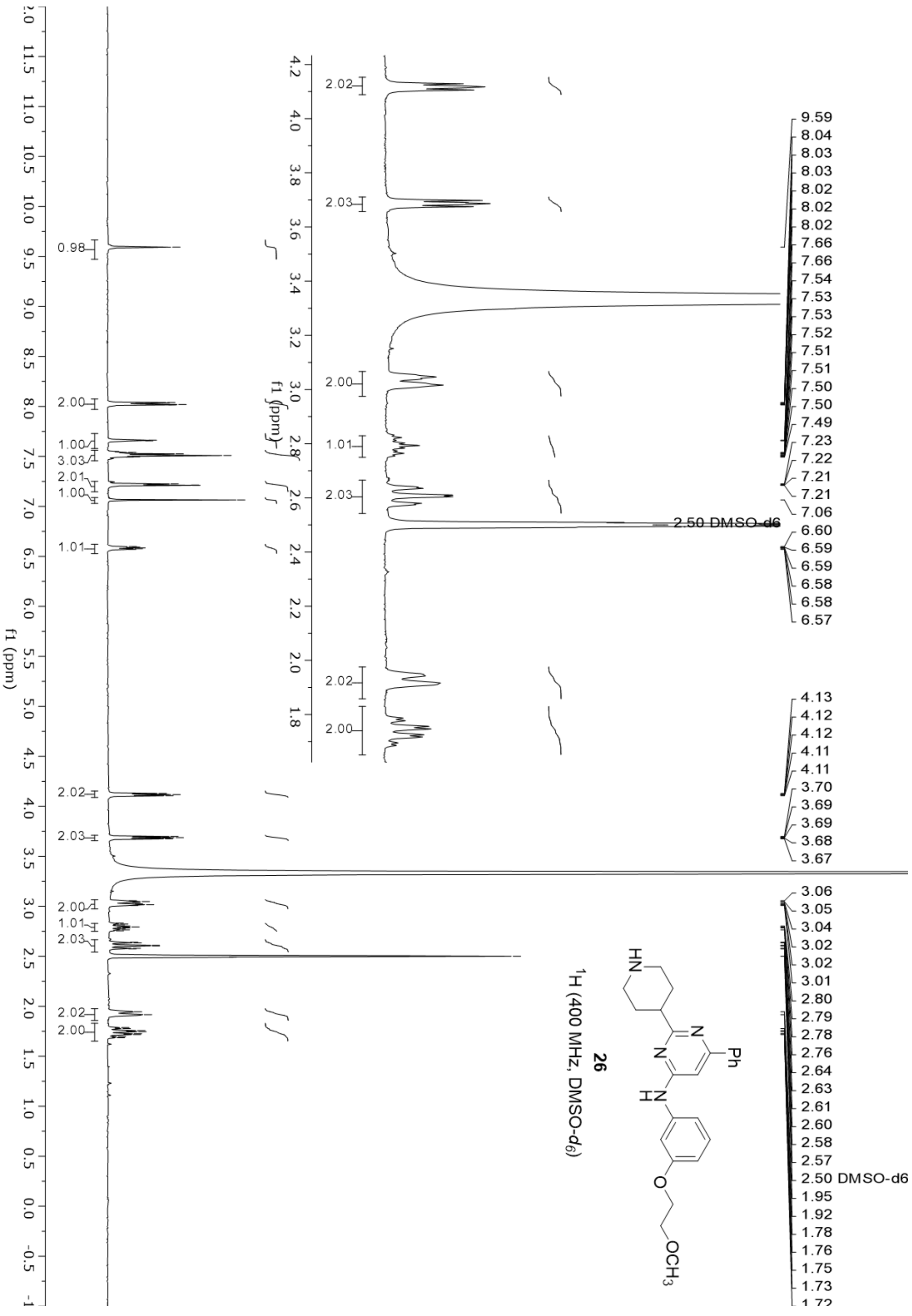
26
^{1}H (400 MHz, DMSO-d_6)
2.50 DMSO-d6
f1 (ppm)

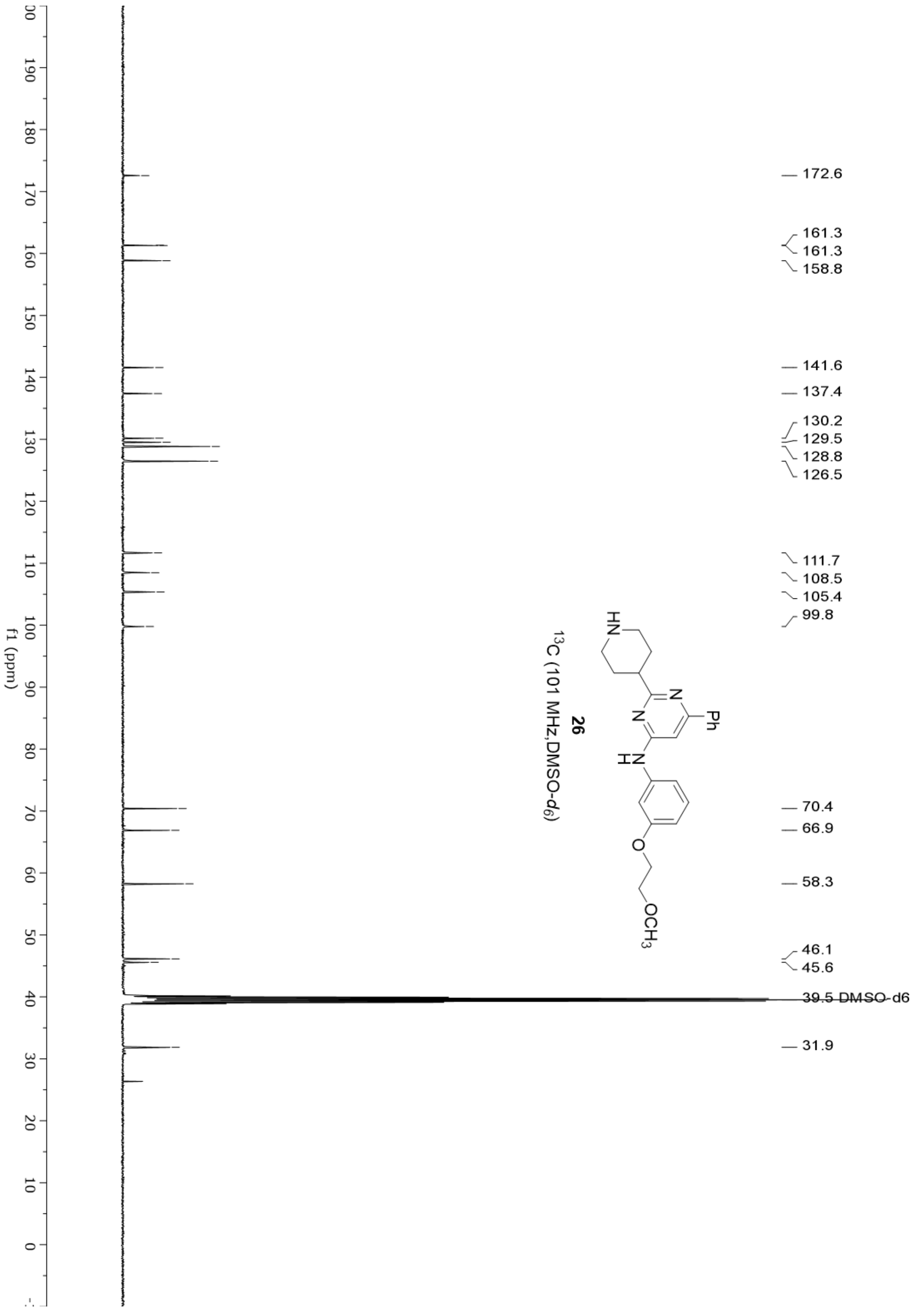

26
^{13}C (101 MHz,DMSO-d_6)

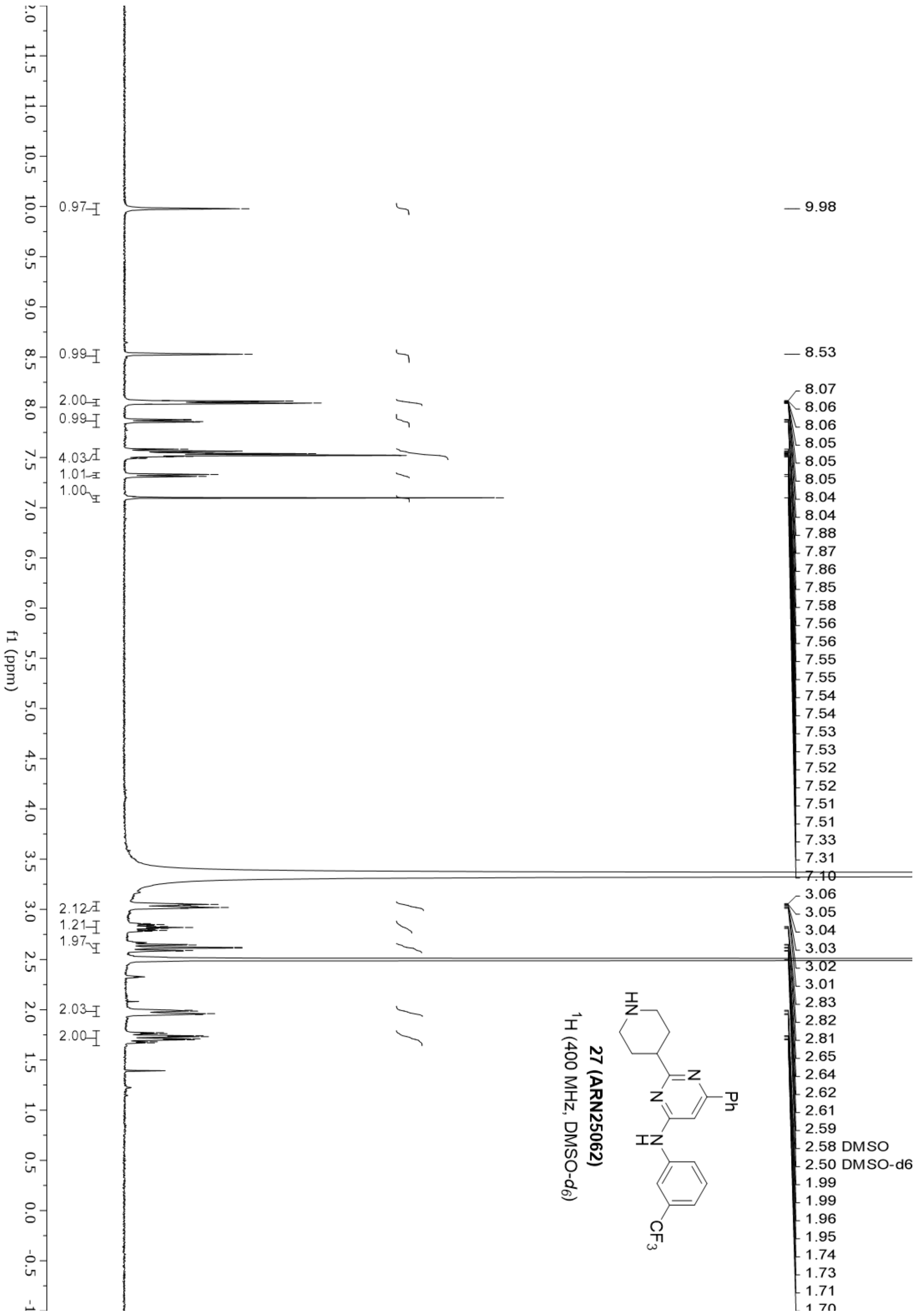
27 (ARN25062)
^{1}H (400 MHz, DMSO-d_6)
Ph
CF3
HN
f1 (ppm)
9.98
8.53
8.07
8.06
8.06
8.05
8.05
8.05
8.04
8.04
7.88
7.87
7.86
7.85
7.58
7.56
7.56
7.55
7.55
7.54
7.54
7.53
7.53
7.52
7.52
7.51
7.51
7.33
7.31
7.10
3.06
3.05
3.04
3.03
3.02
3.01
2.83
2.82
2.81
2.65
2.64
2.62
2.61
2.59
2.58 DMSO
2.50 DMSO-d6
1.99
1.99
1.96
1.95
1.74
1.73
1.71
1.70
0.97
0.99
2.00
0.99
4.03
1.01
1.00
2.12
1.21
1.97
2.03
2.00

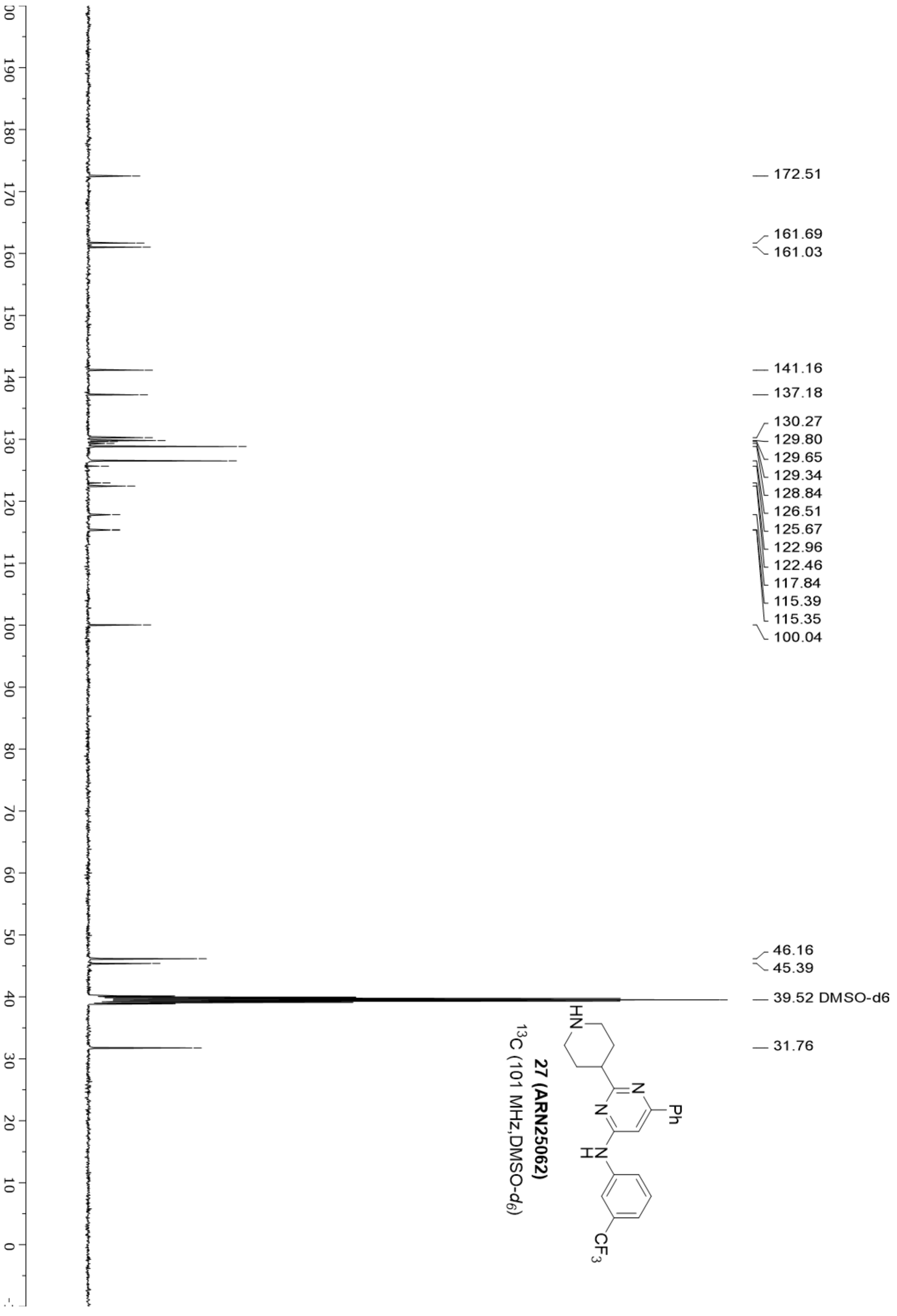
172.51
161.69
161.03
141.16
137.18
130.27
129.80
129.65
129.34
128.84
126.51
125.67
122.96
122.46
117.84
115.39
115.35
100.04
46.16
45.39
39.52 DMSO-d6
31.76
f1 (ppm)
HN
N
N
Ph
N
H
CF3
27 (ARN25062)
13C (101 MHz,DMSO-d6)

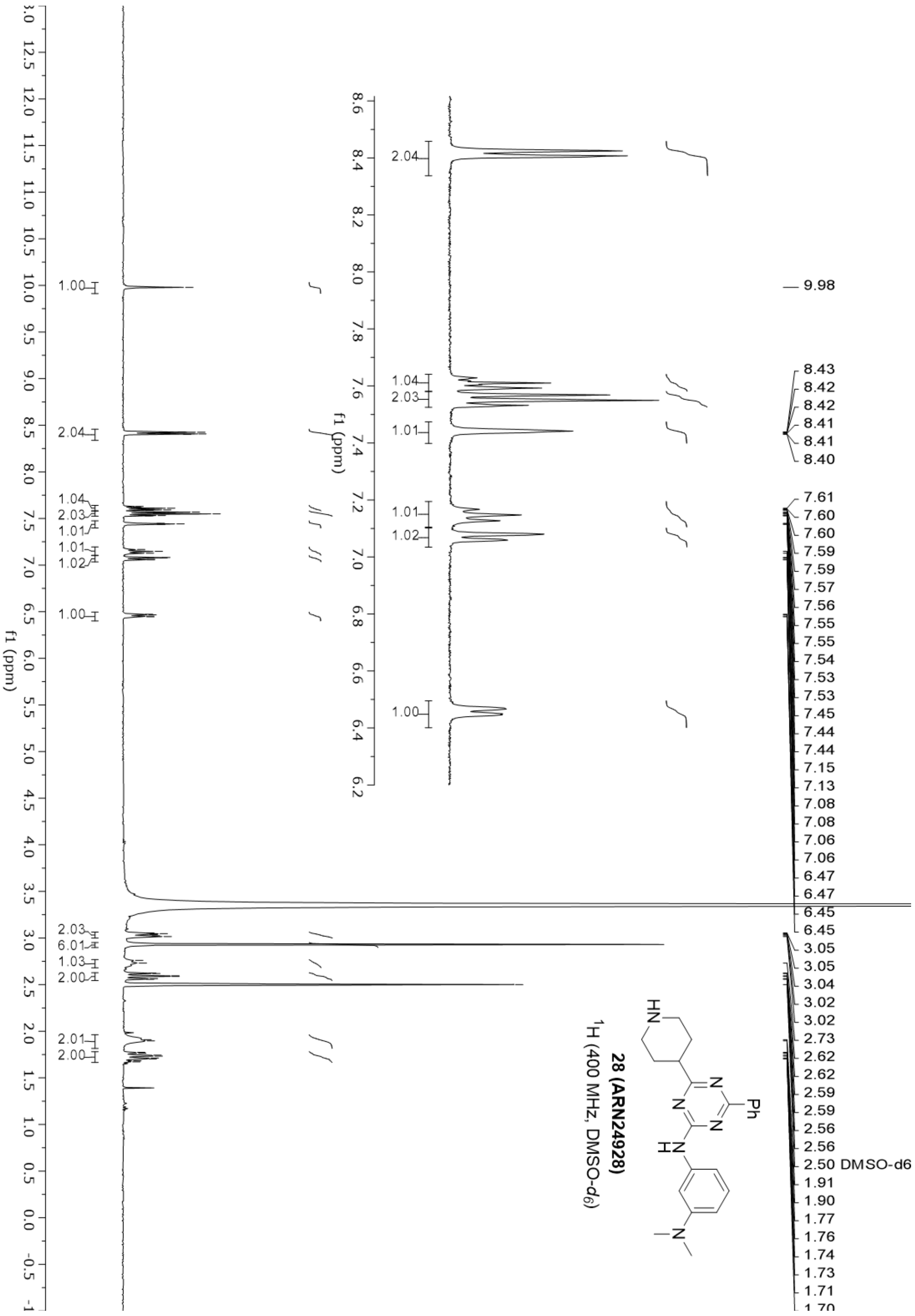
28 (ARN24928)
1H (400 MHz, DMSO-d6)
f1 (ppm)
2.50 DMSO-d6

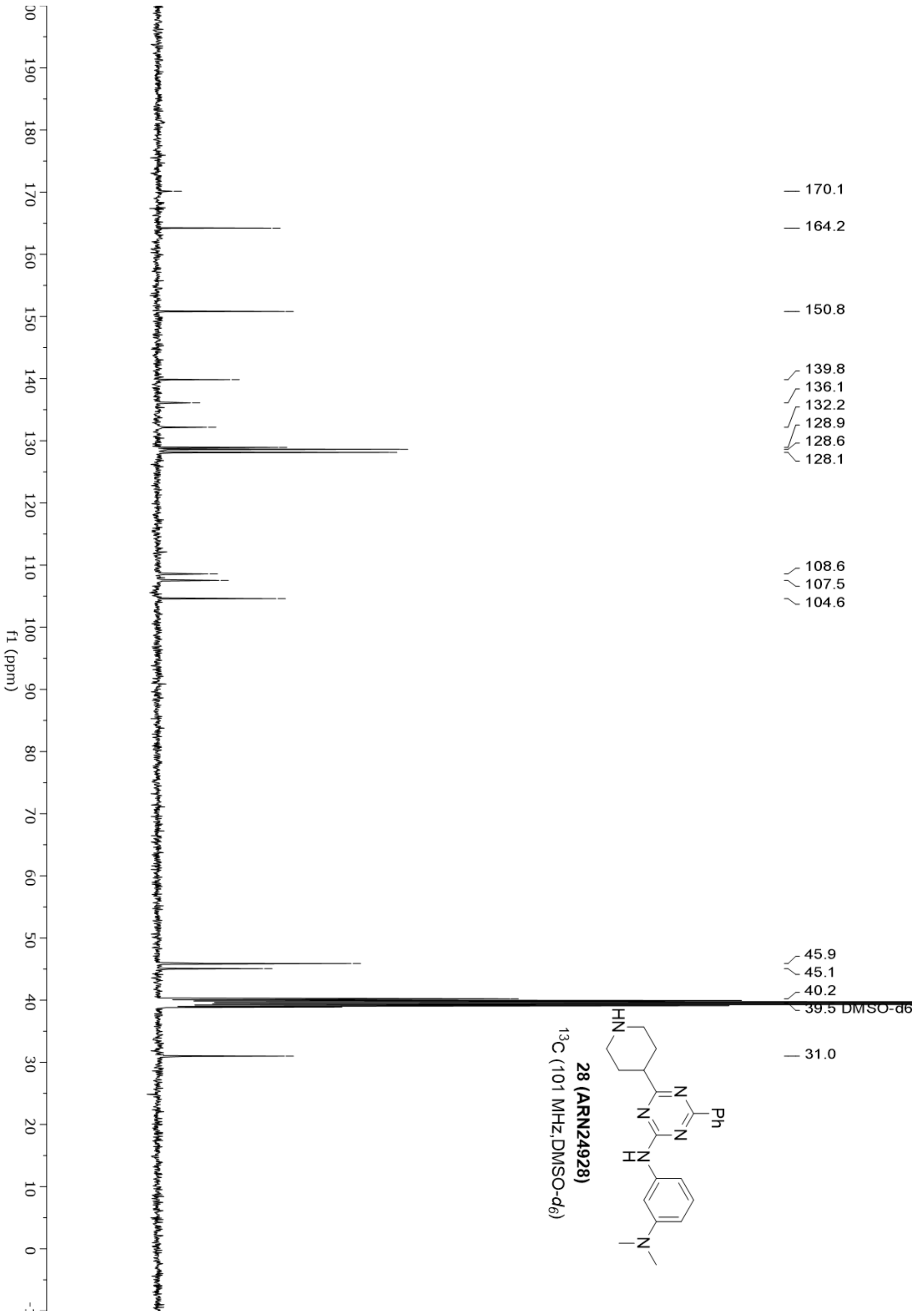

170.1
164.2
150.8
139.8
136.1
132.2
128.9
128.6
128.1
108.6
107.5
104.6
45.9
45.1
40.2
39.5 DMSO-d6
31.0
f1 (ppm)
HN
N
N
N
Ph
N
H
N
28 (ARN24928)
^{13}C (101 MHz,DMSO-d_6)

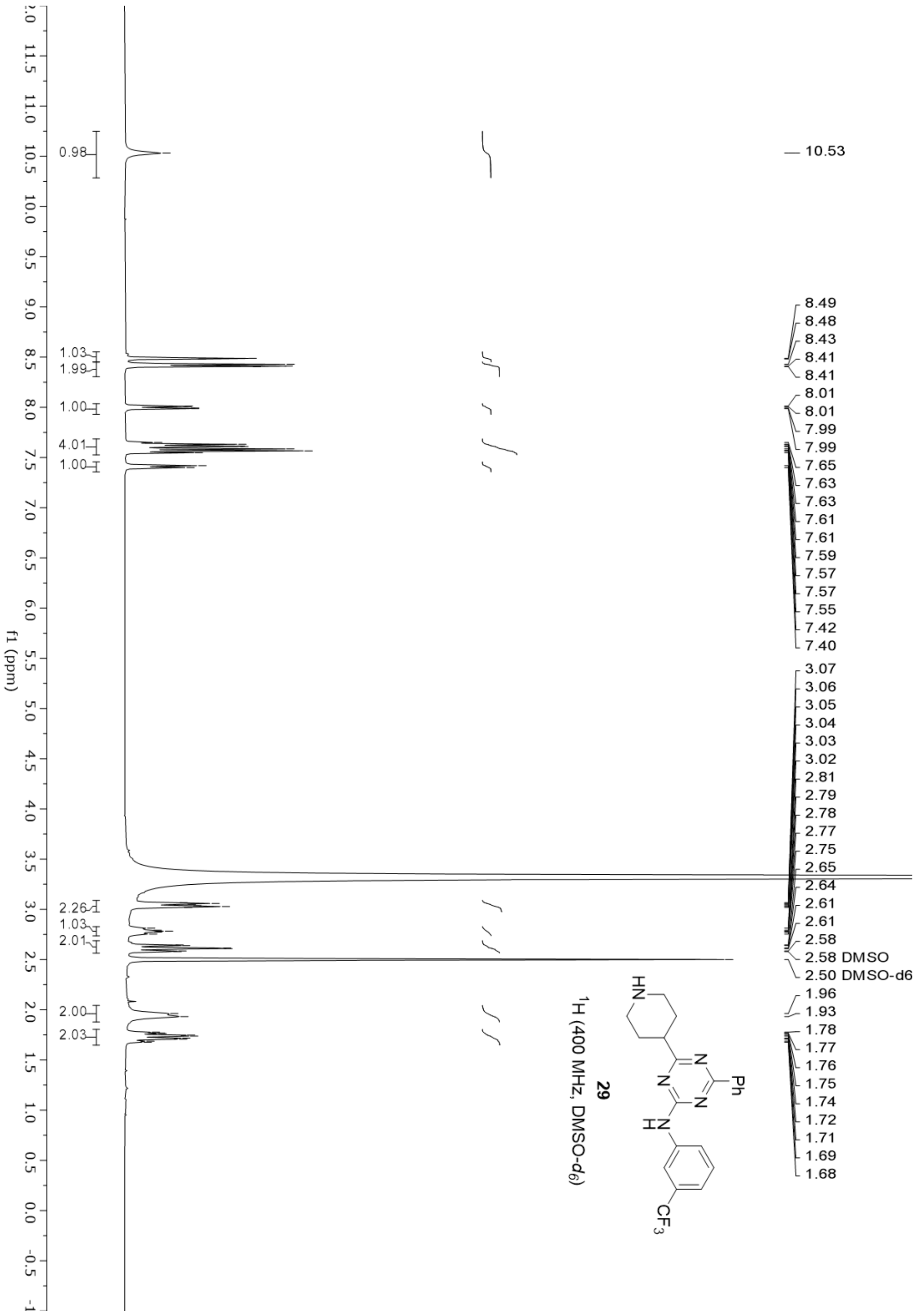

10.53
8.49
8.48
8.43
8.41
8.41
8.01
8.01
7.99
7.99
7.65
7.63
7.63
7.61
7.61
7.59
7.57
7.57
7.55
7.42
7.40
3.07
3.06
3.05
3.04
3.03
3.02
2.81
2.79
2.78
2.77
2.75
2.65
2.64
2.61
2.61
2.58
2.58 DMSO
2.50 DMSO-d6
1.96
1.93
1.78
1.77
1.76
1.75
1.74
1.72
1.71
1.69
1.68
0.98
1.03
1.99
1.00
4.01
1.00
2.26
1.03
2.01
2.00
2.03
f1 (ppm)
Ph
N
N
N
HN
N
H
CF3
29
1H (400 MHz, DMSO-d6)

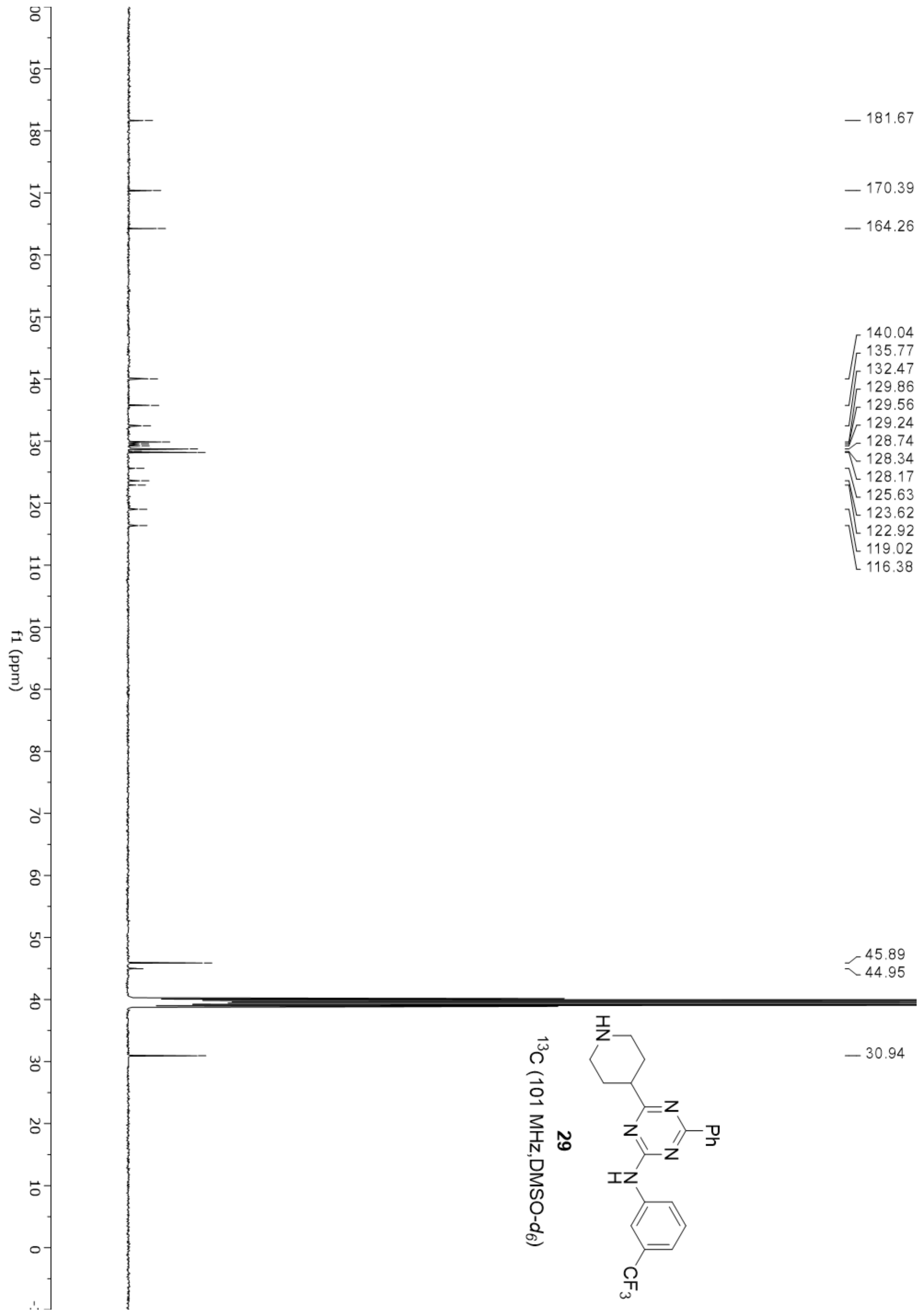
181.67
170.39
164.26
140.04
135.77
132.47
129.86
129.56
129.24
128.74
128.34
128.17
125.63
123.62
122.92
119.02
116.38
45.89
44.95
30.94
f1 (ppm)
HN
N
N
N
Ph
N
H
CF3
29
13C (101 MHz,DMSO-d6)

2. Chromatographic analysis of key compounds

After their synthesis, all the compounds underwent UPLC- MS quality control analysis using reversed phase chromatography. Analyses were conducted on a Waters Acquity UPLC-MS system consisting of a single quadrupole detector (SQD) mass spectrometer equipped with an electrospray ionization interface and a photodiode array detector (PDA) from Waters Inc. (Milford, MA, USA). The scan range was set to 110–650 *m*/*z* for both polarities (ESI+ and ESI−). The PDA wavelength range was 210–400 nm (bottom image), and the UV purity was determined at the specific wavelength of 215 nm (upper image). The analyses were performed on an Acquity UPLC BEH C_{18} column (100x2.1mmID, particle size 1.7µm) with a VanGuard BEH C_{18} pre-column (5x2.1mmID, particle size 1.7µm) using 10 mM NH_4OAc in H_2O at pH 5 adjusted with AcOH and 10 mM NH_4OAc in CH_3CN-H_2O (95:5) at pH 5 (B) as mobile phase. After an initial hold for 0.2 min at 10% B, a linear gradient was applied to 90% B in 6 min, then from 90 to 100% B in 0.1 min, followed by a hold at 100% for 0.4 min. A 10 mM stock solution in dry DMSO was prepared for each test compound, and further diluted 20× in CH_3CN–H_2O (1:1) prior to analysis.
As showed by chromatograms below, all compounds displayed an UV purity > 95%, with the exception of compounds **4** (UV purity > 93%), **7** (UV purity = 90%), **12** (UV purity > 91%), and **25** (UV purity > 93%).

Compound **1**.

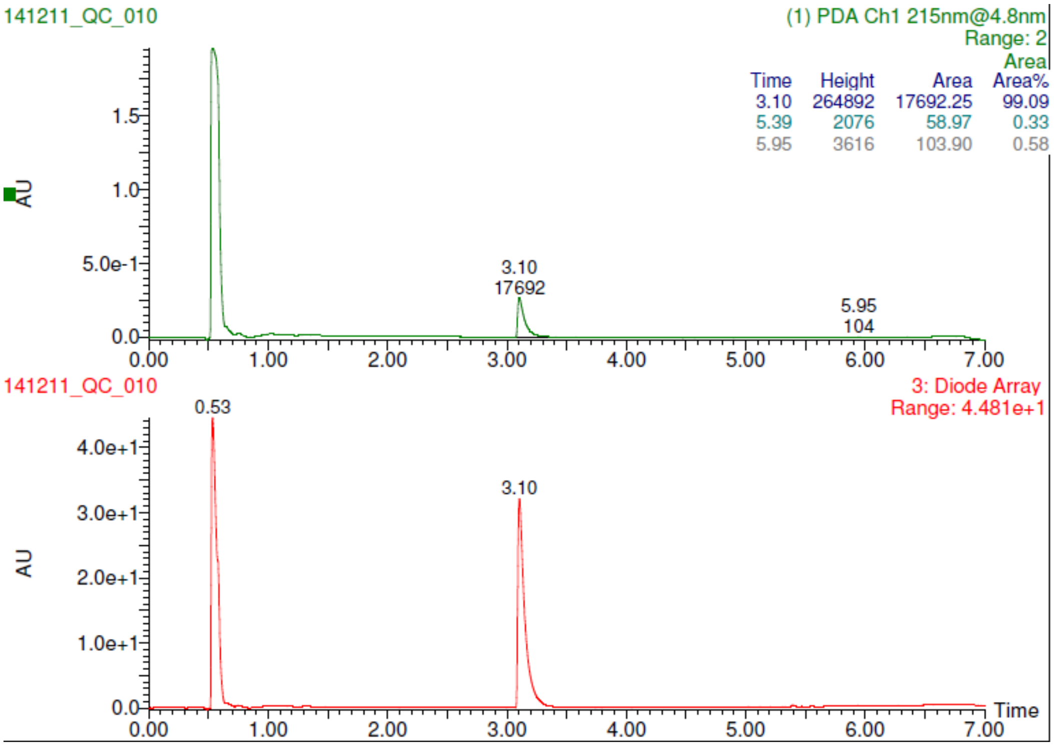

Compound **2**.

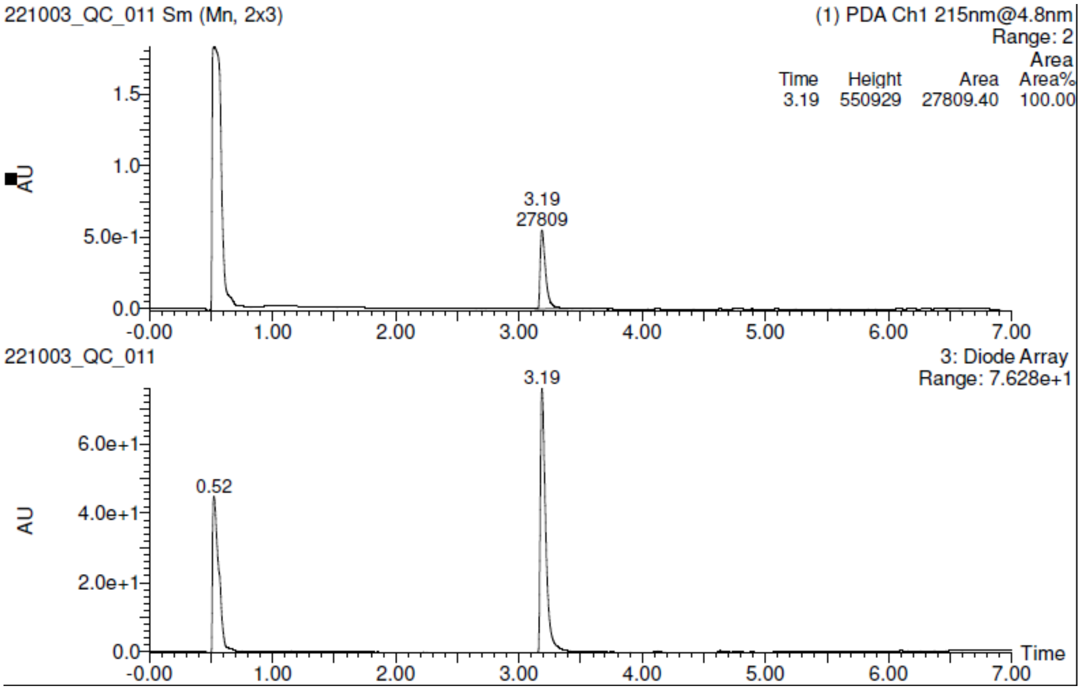

Compound **3**.

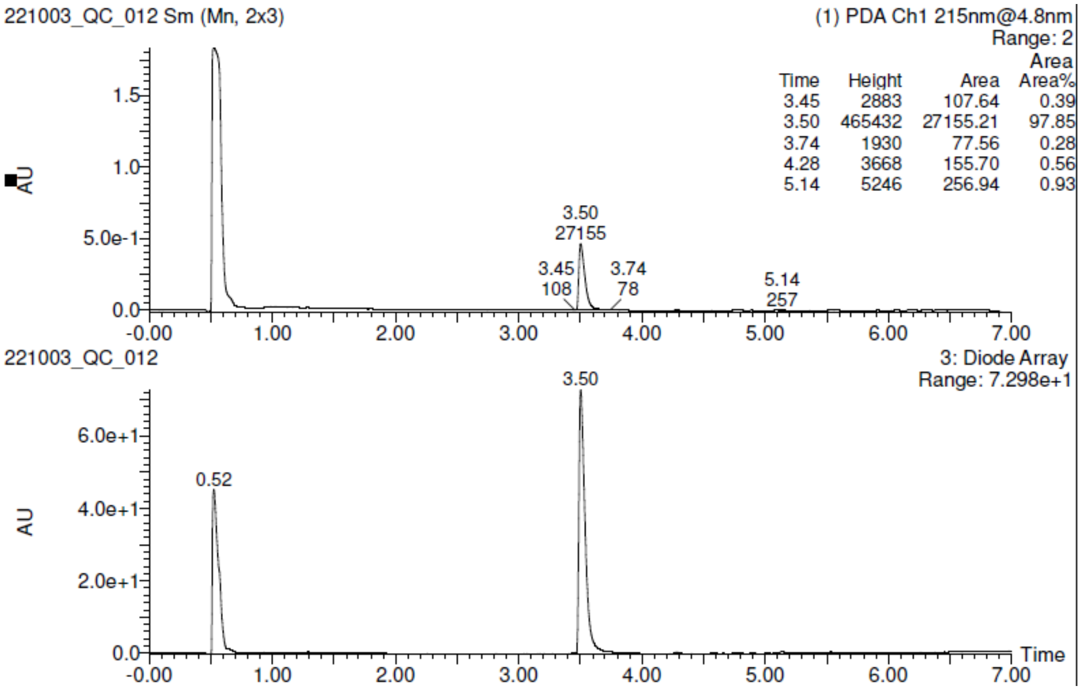

Compound **4**.

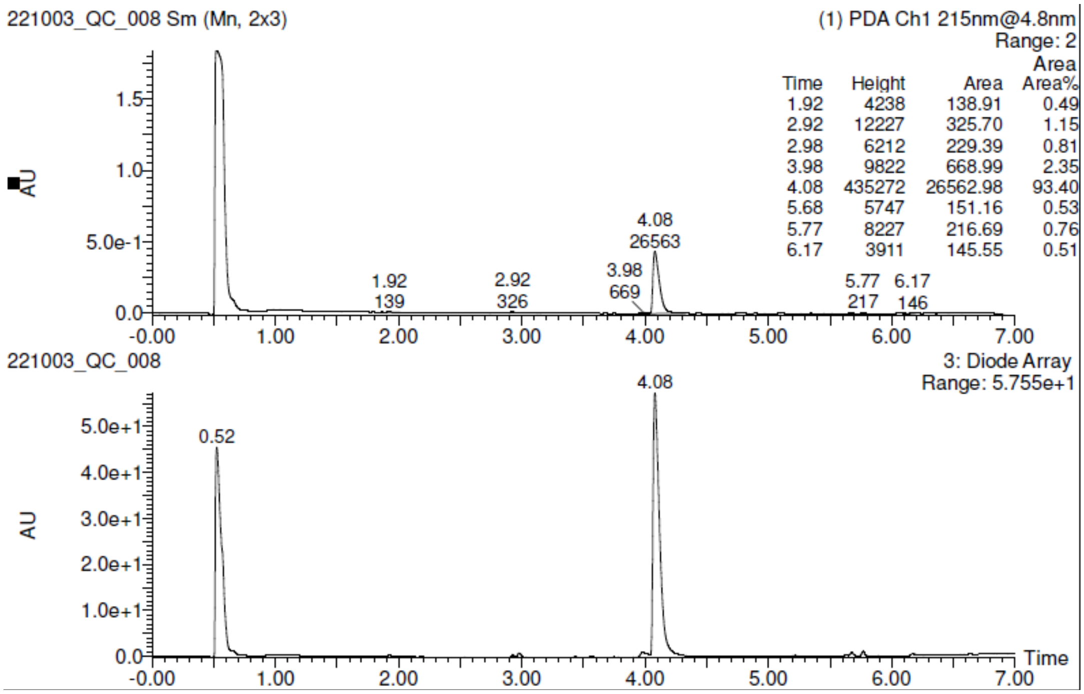

Compound **5**.

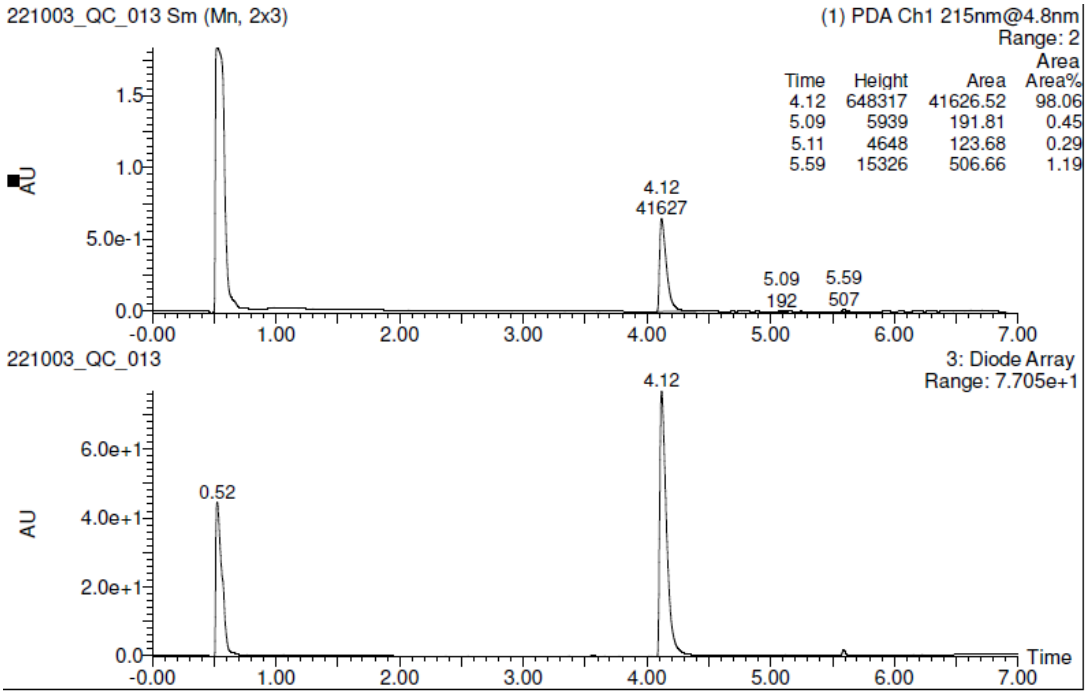

Compound **6**.

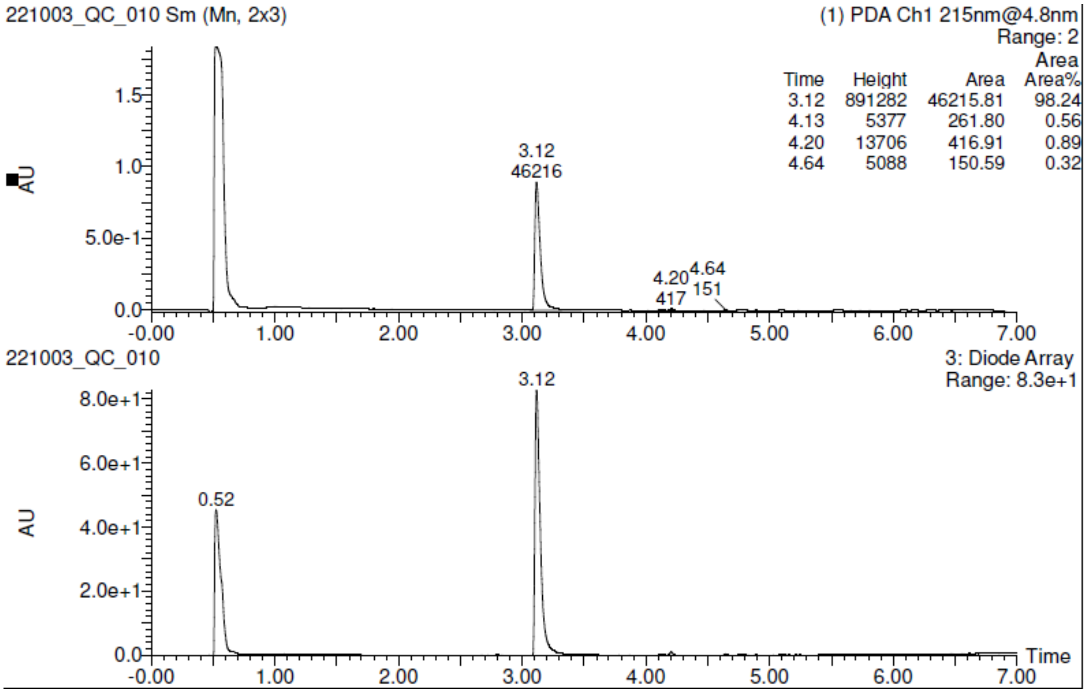

Compound **7**.

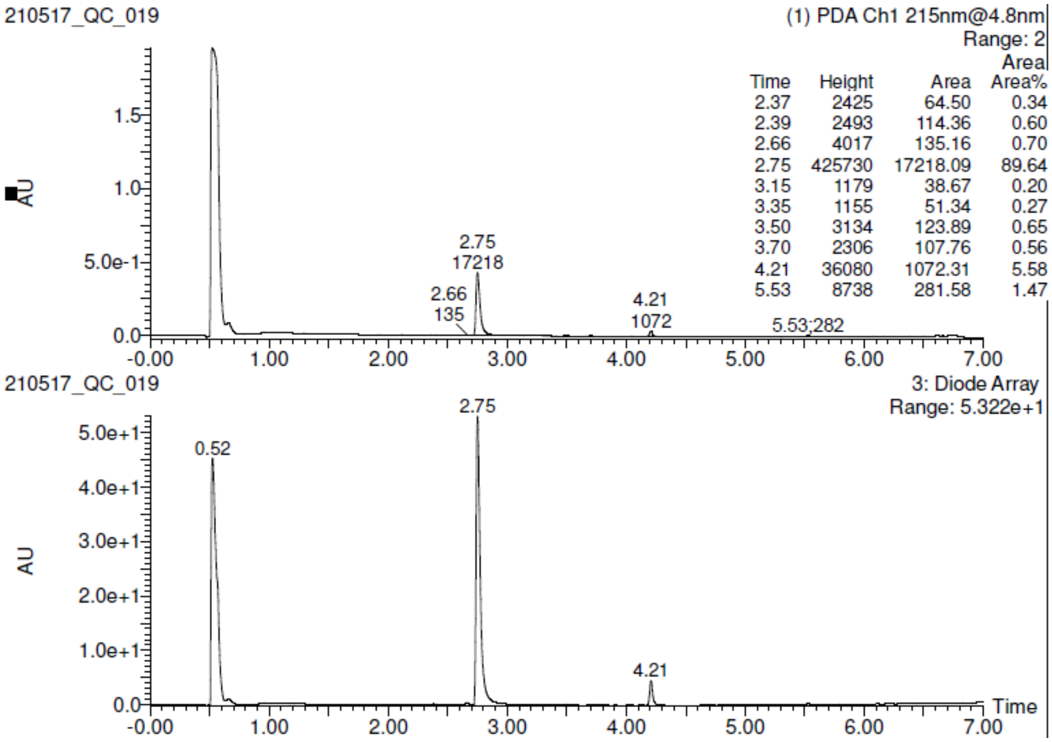

Compound **8**.

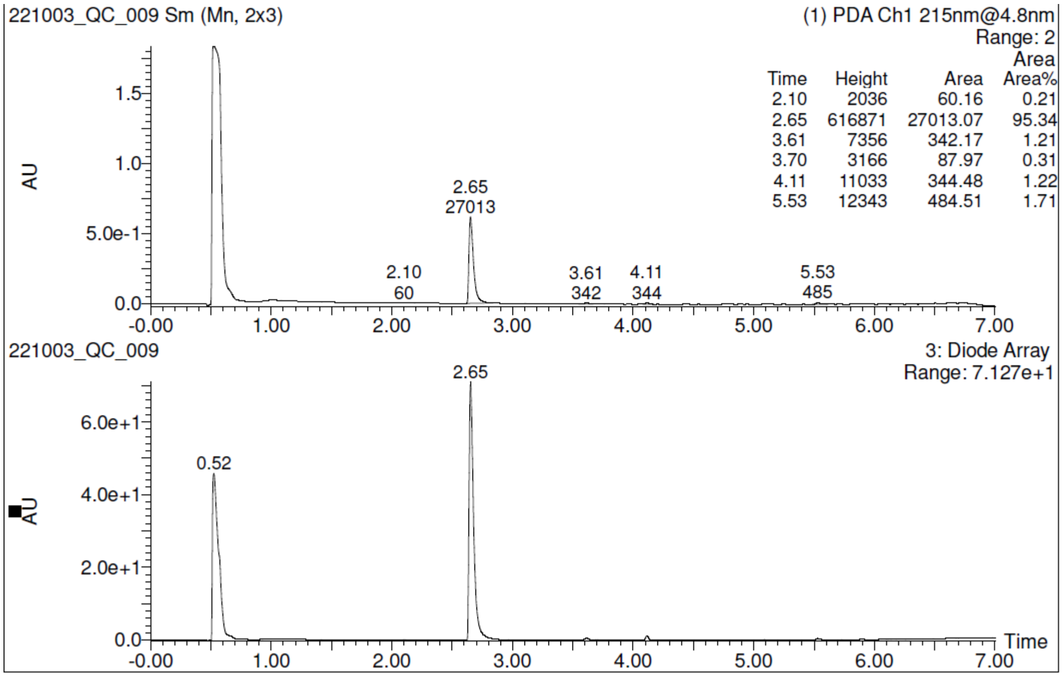

Compound **9**.

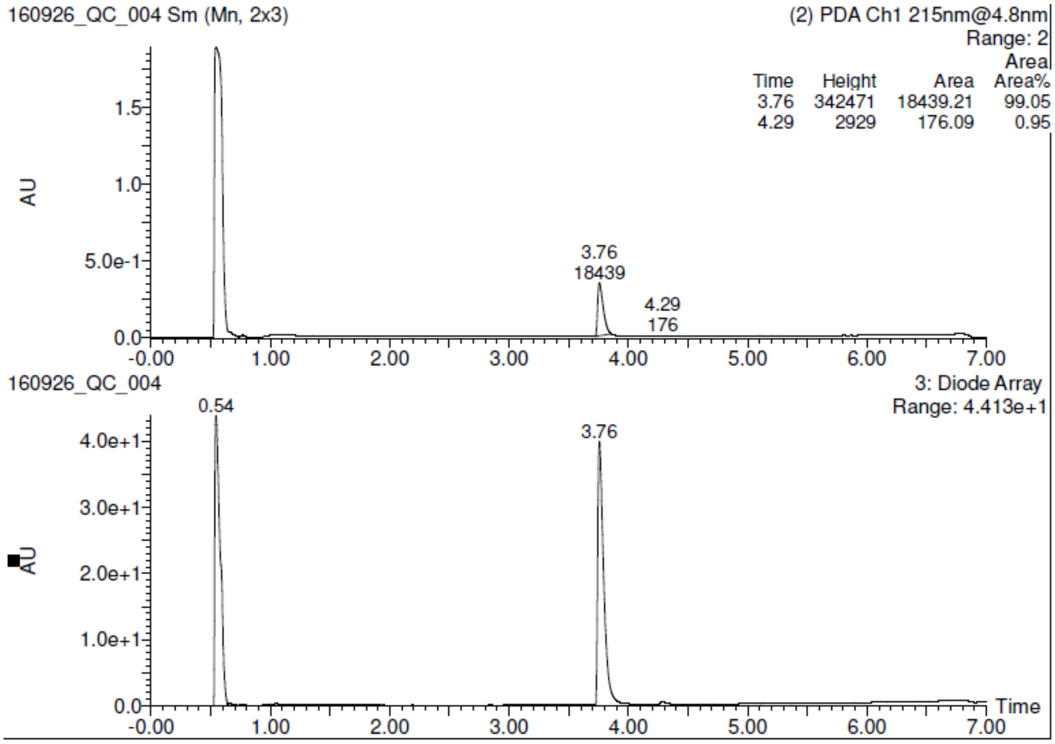

Compound **10**.

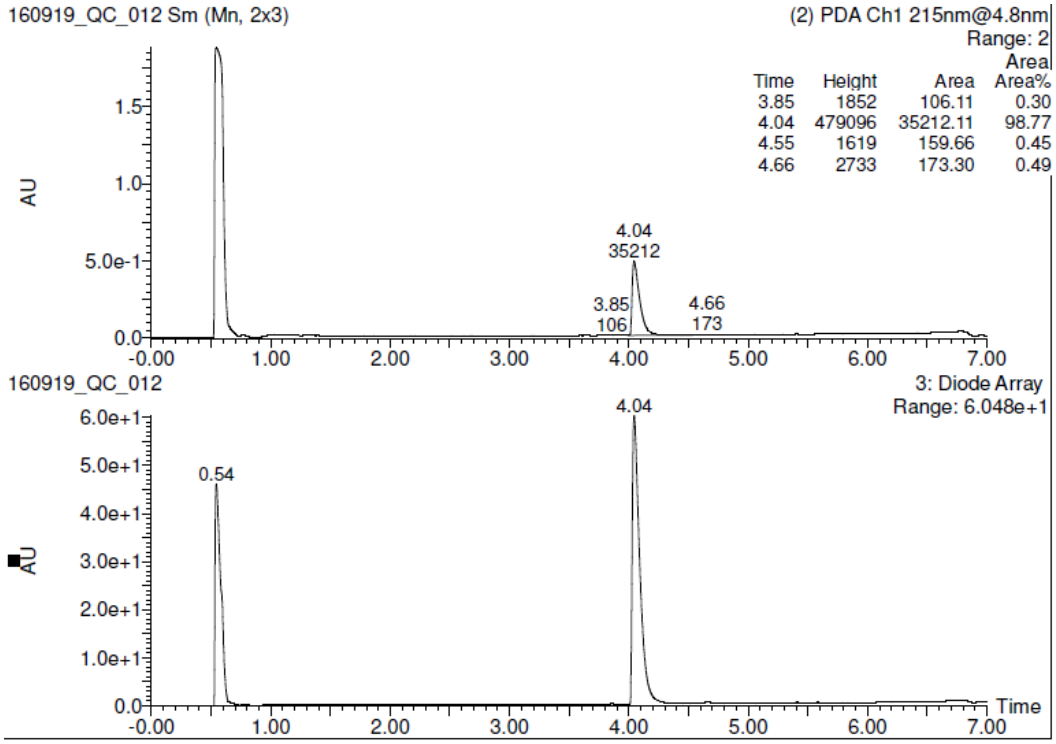

Compound **11**.

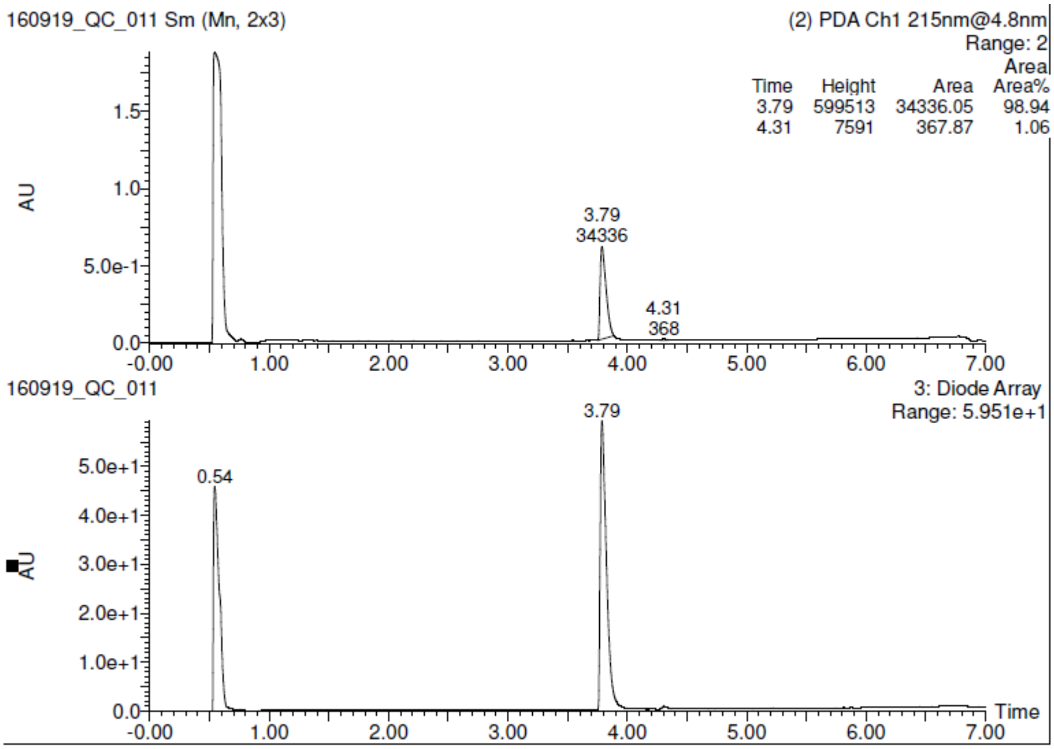

Compound **12**.

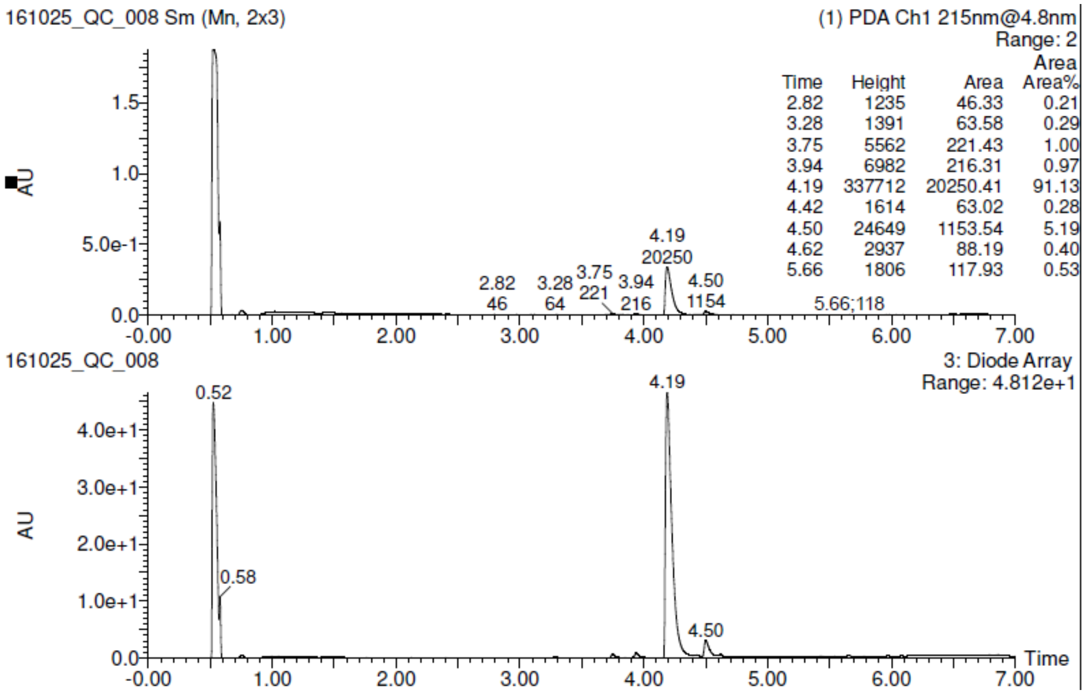

Compound **13**.

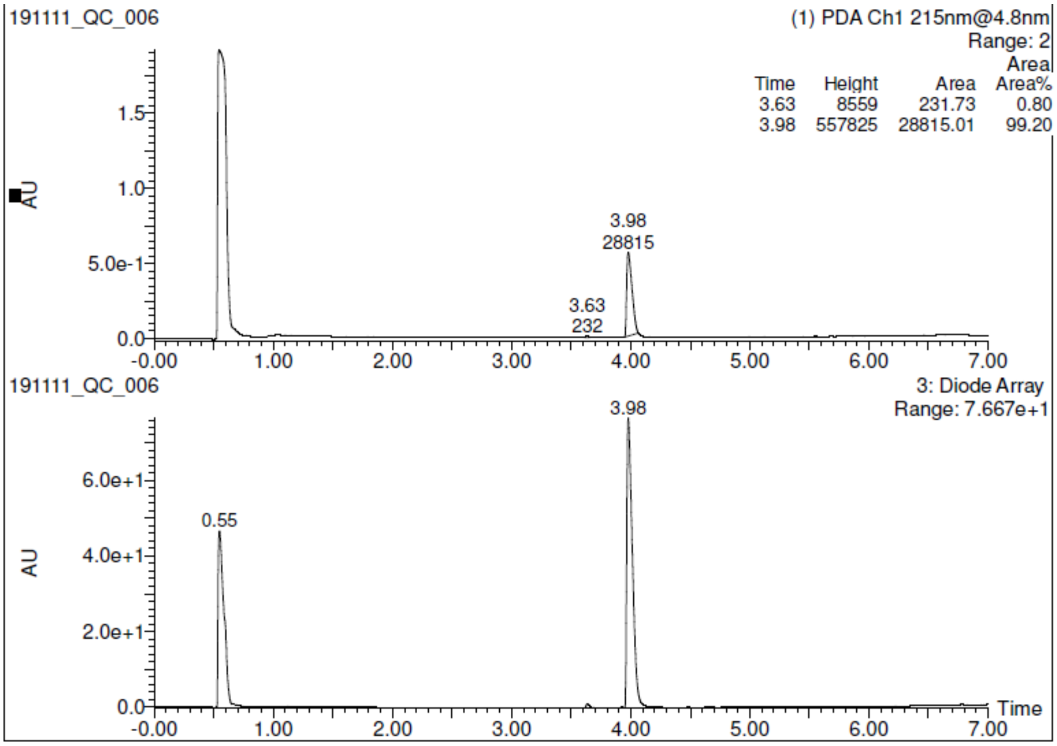

Compound **14**.

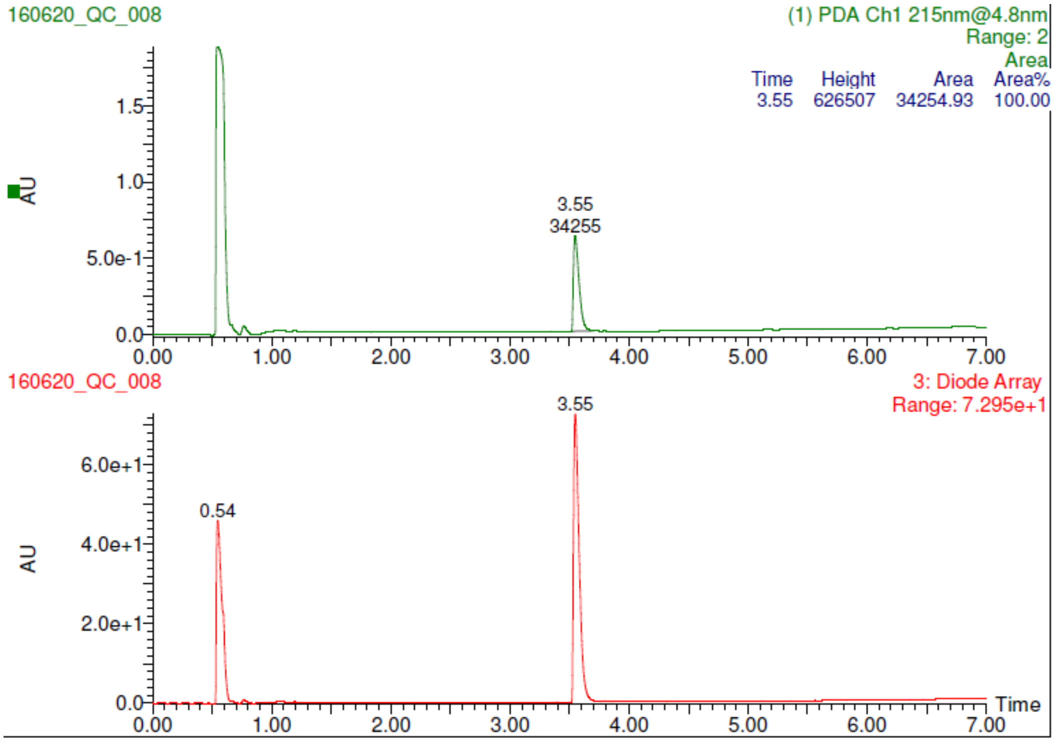

Compound **15**.

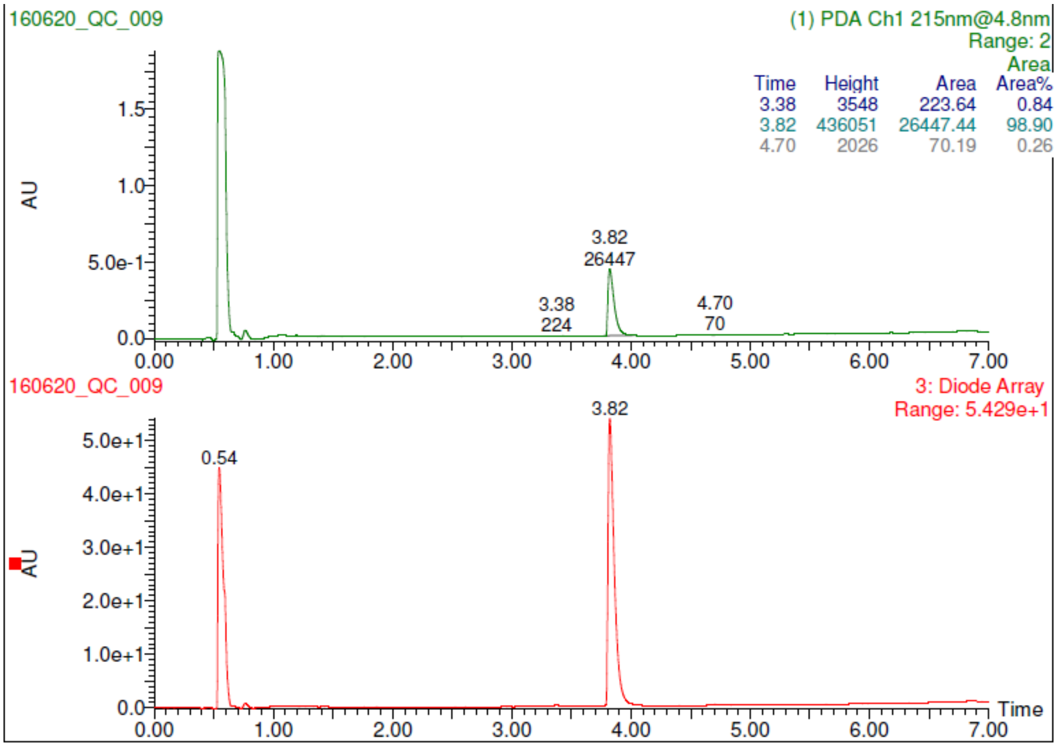

Compound **16**.

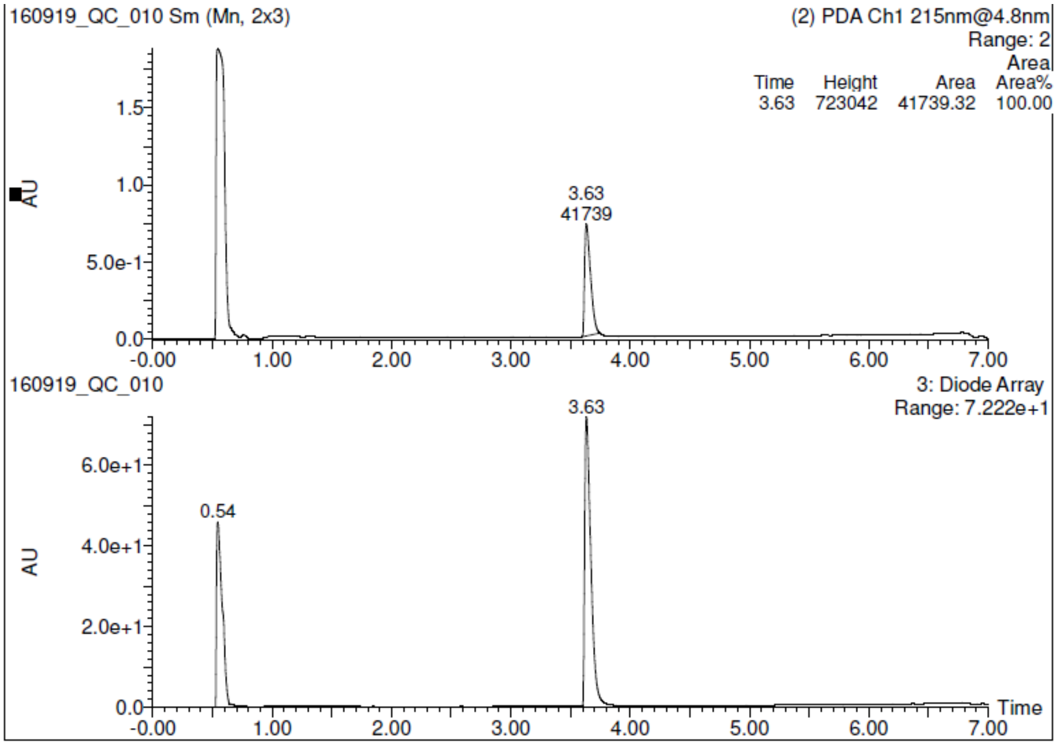

Compound **17**.

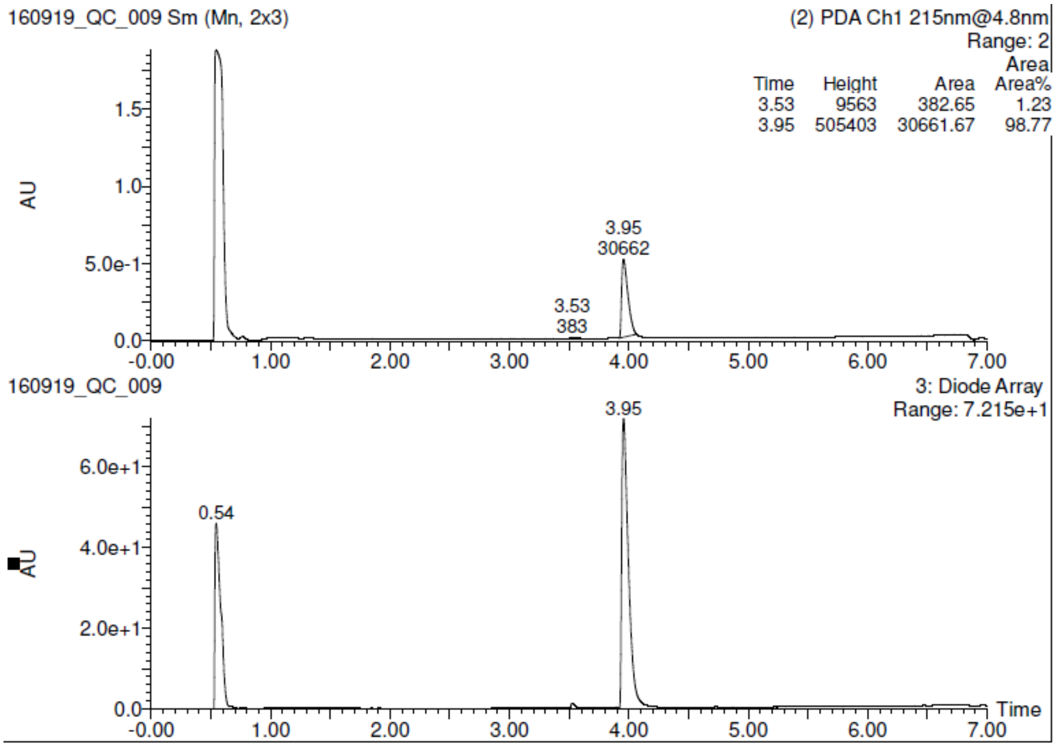

Compound **18**.

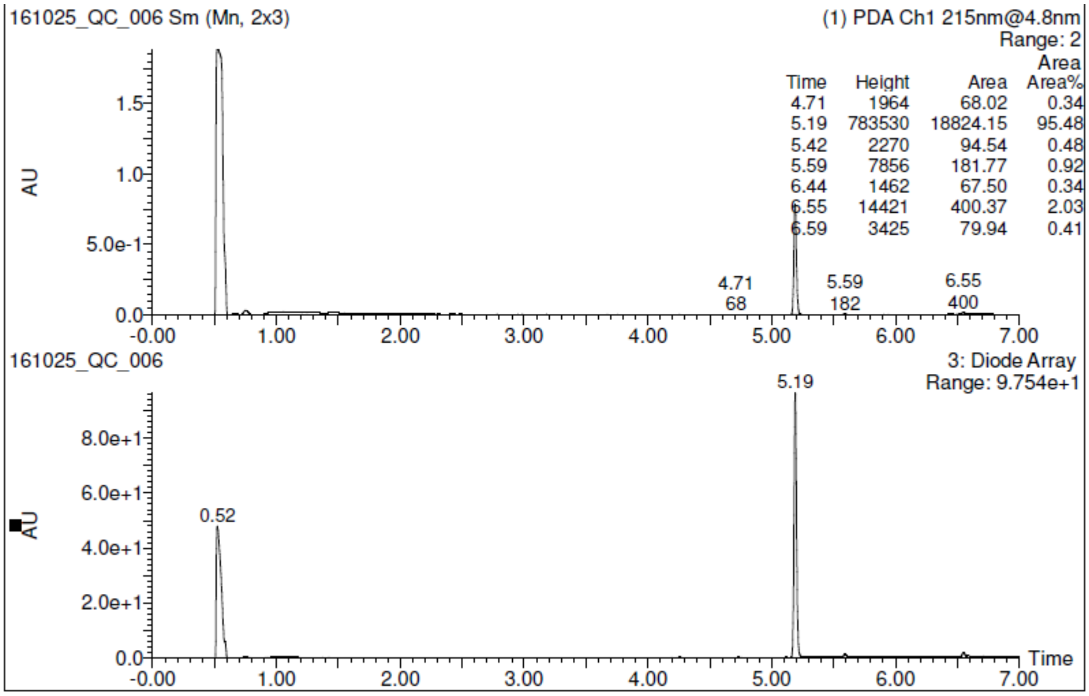

Compound **19**.

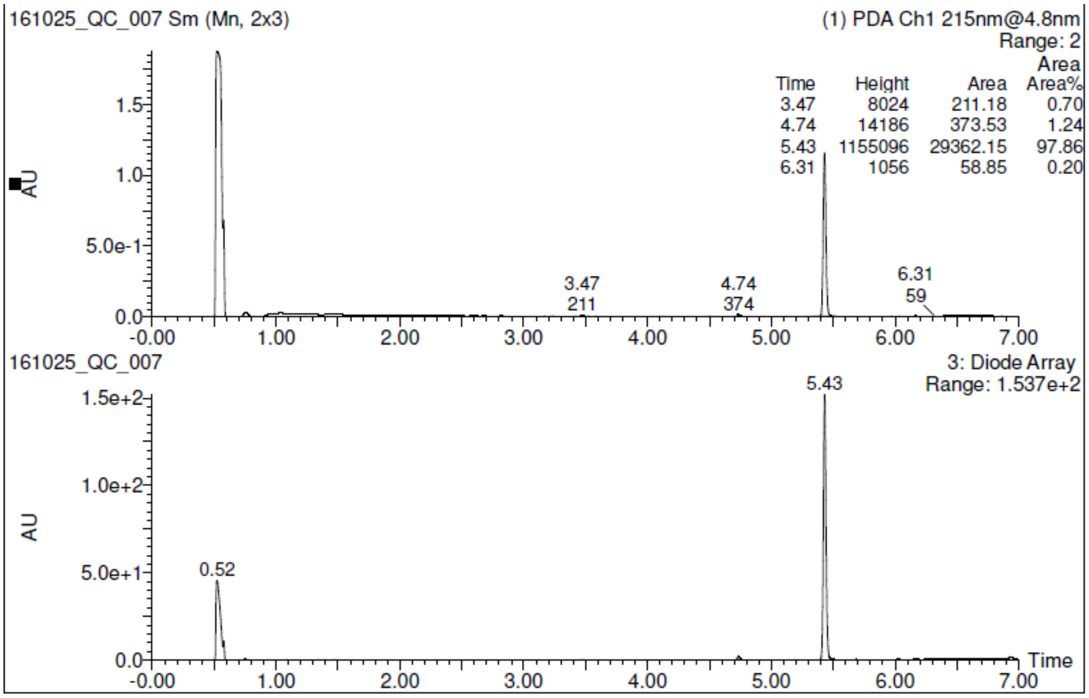

Compound **20**.

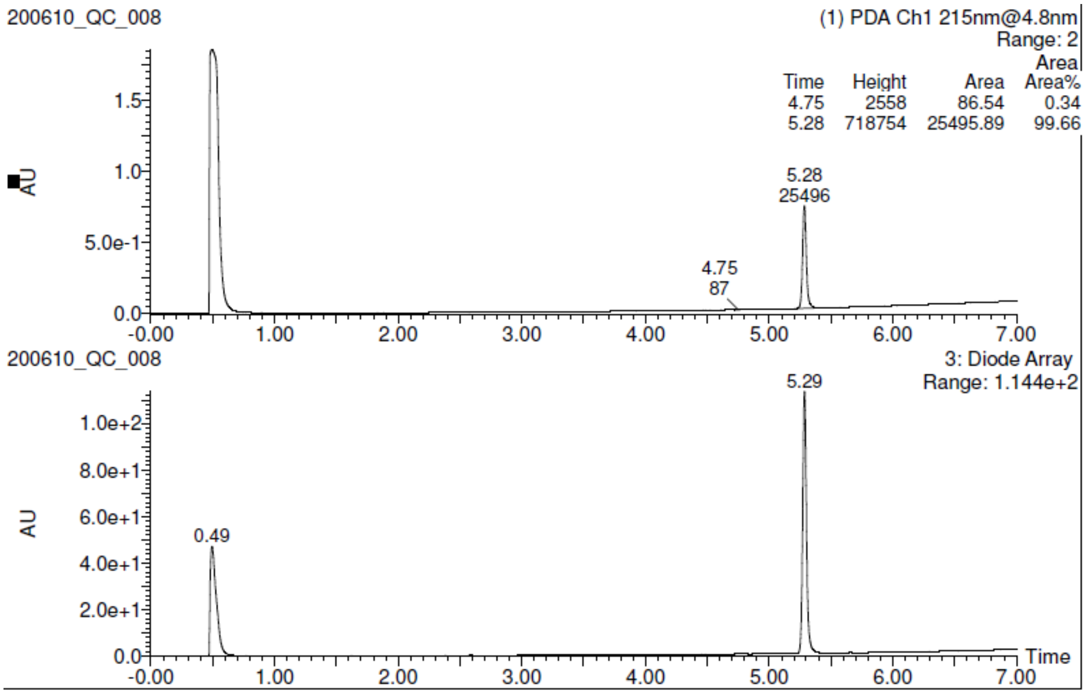

Compound **21**.

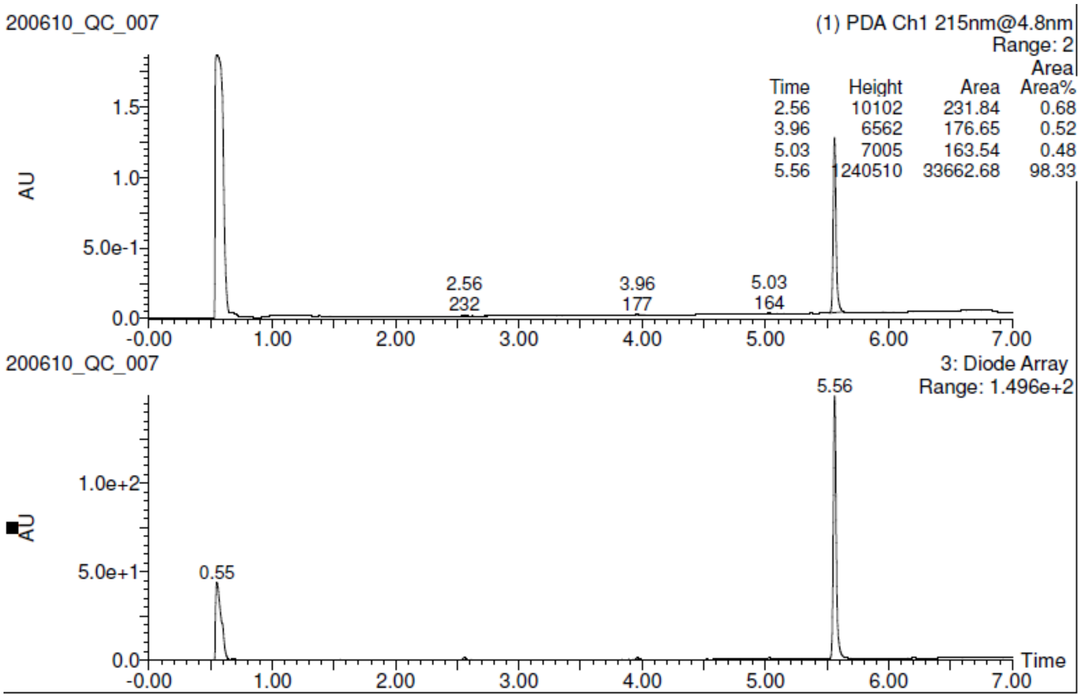

Compound **22**.

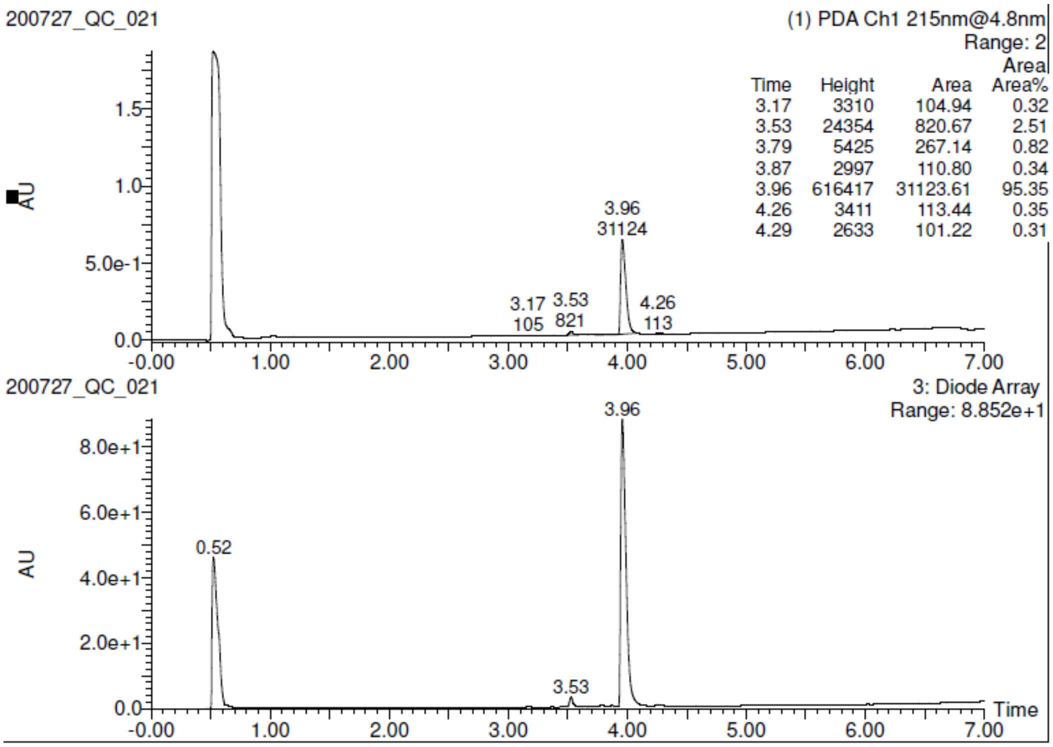

Compound **23**.

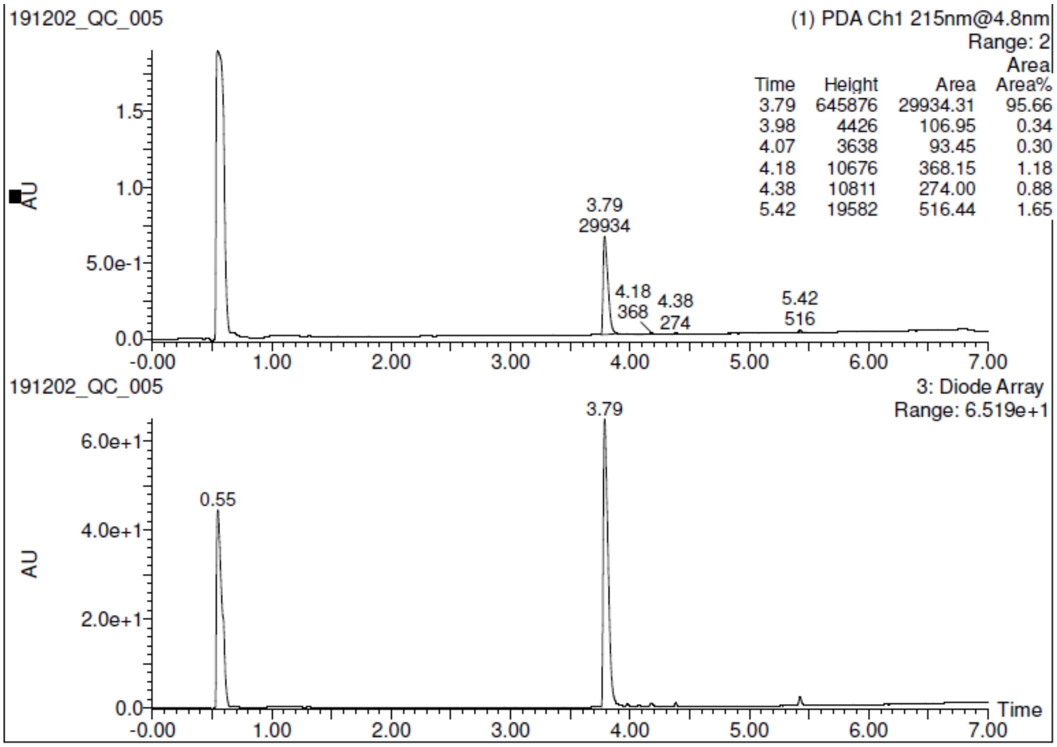

Compound **24**.

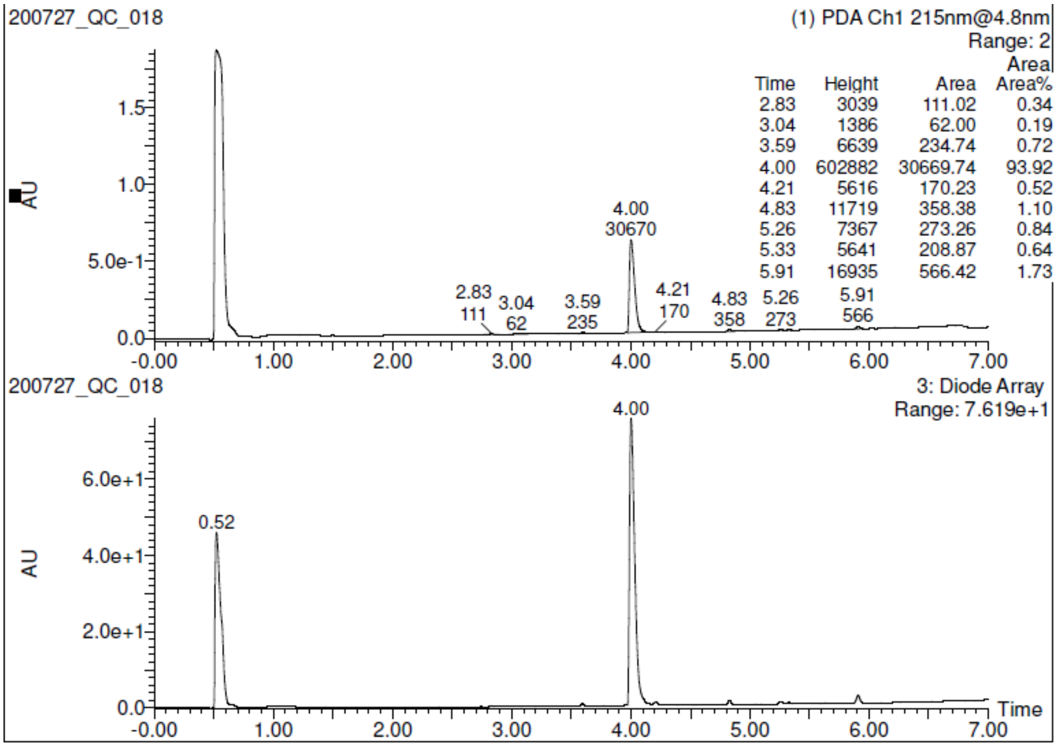

Compound **25**.

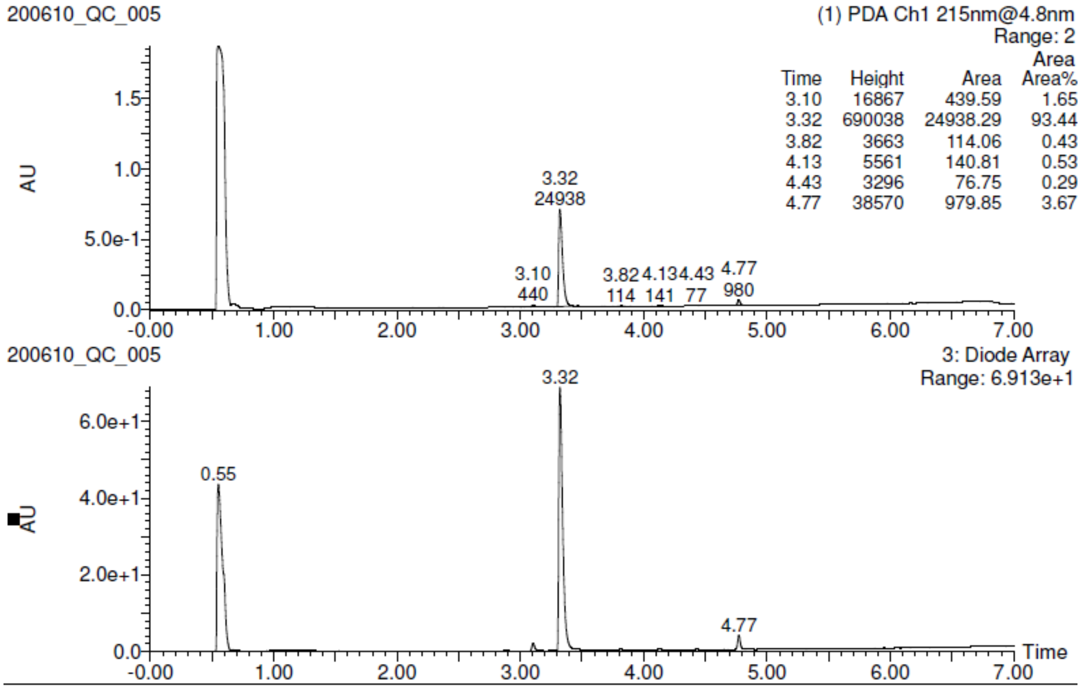

Compound **26**.

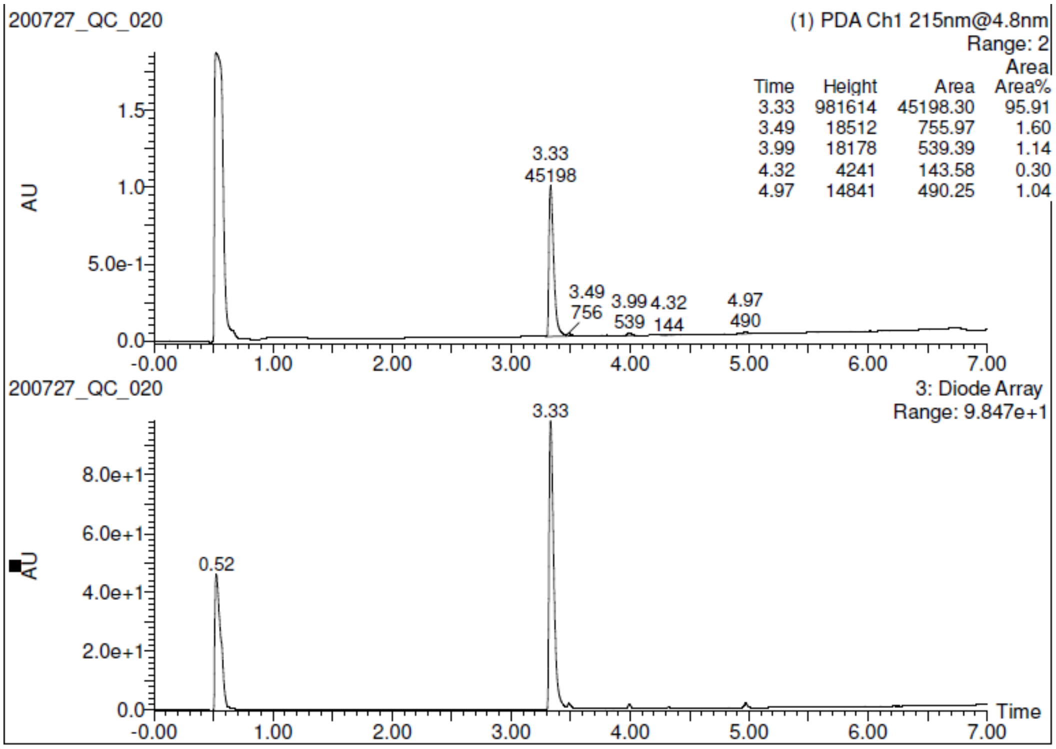

Compound **27**.

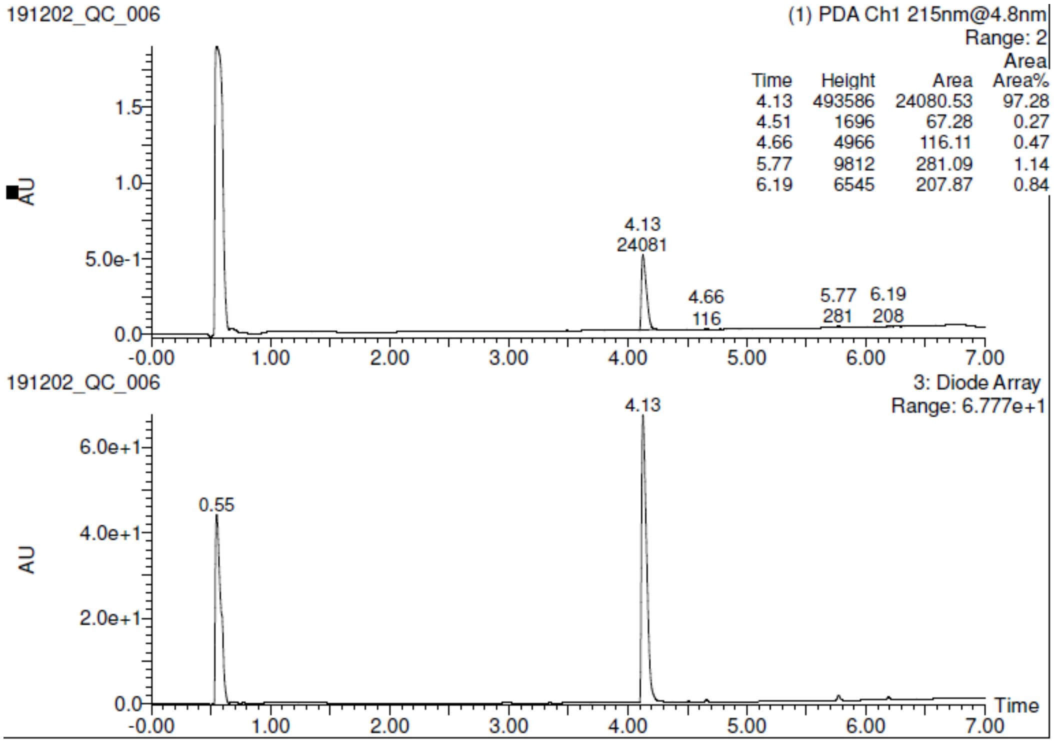

Compound **28**.

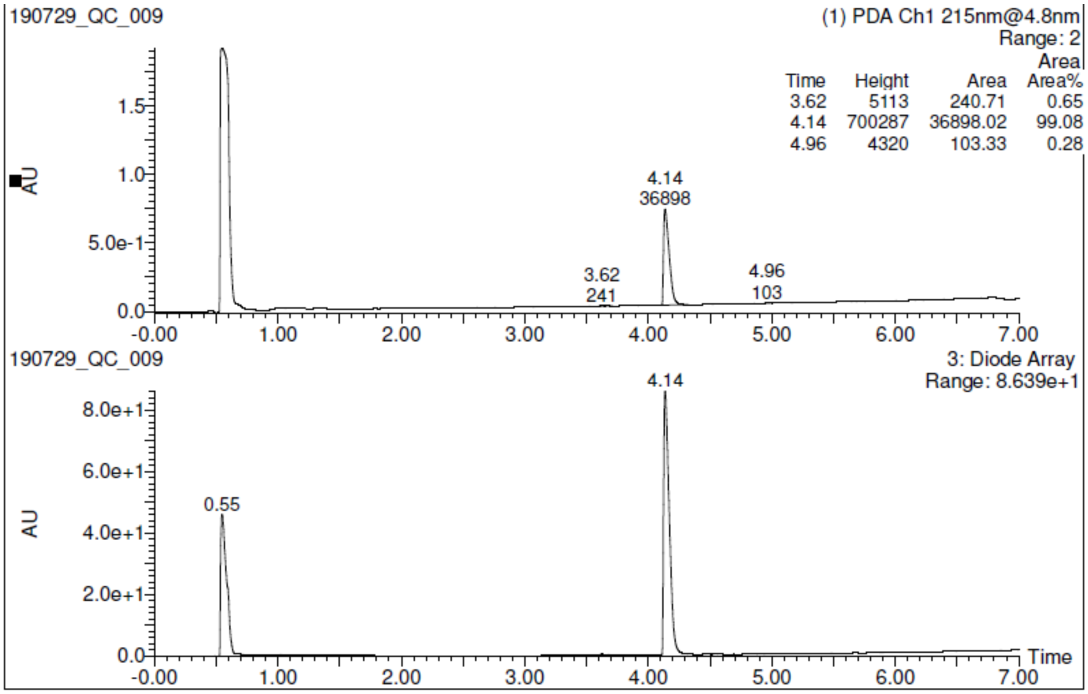

Compound **29**.

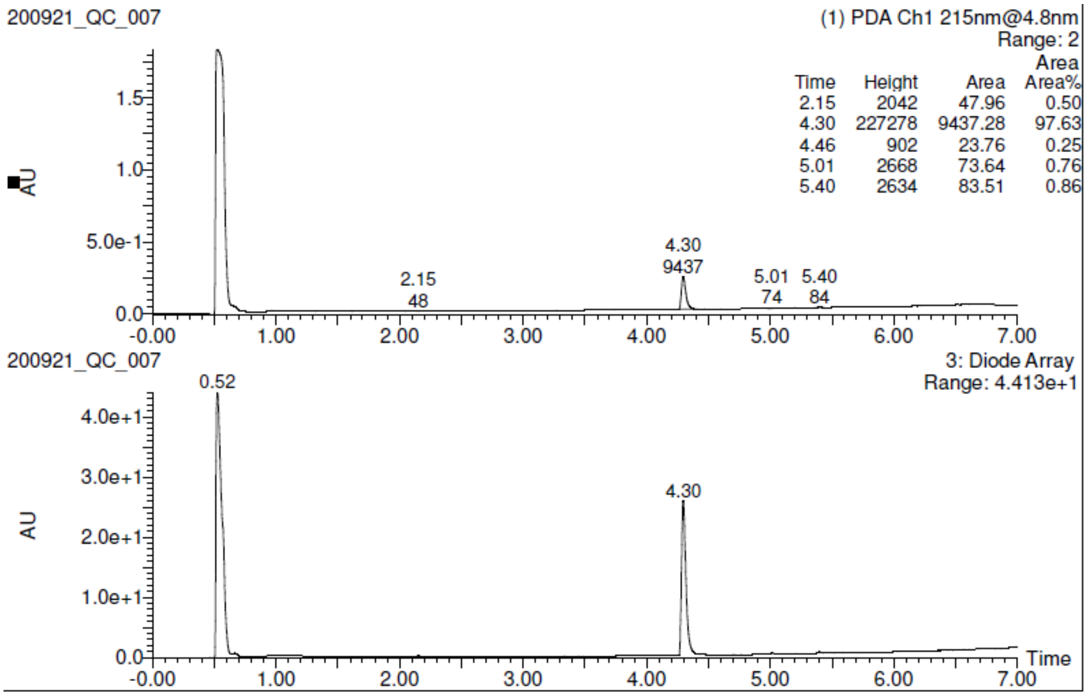

3. Supplemental Table S1

Summary of microscale thermophoresis and NMR binding check. Soluble compounds were screened for their binding to His-CDC42. The protein was previously activated/inactivated by loading with either GppNHp (active) or GDP (inactive). Assays were set up in Tris-Cl buffer (**X**) for the binding of the compounds to His-CDC42 loaded with GppNHp (active) or GDP (inactive). PBS buffer (**Y**) was also tested for the binding of the compounds to the active His-CDC42. The amplitude (**A**) as well as the signal to noise ratio (**S/N**) cut-off at 2.5 sec is reported. **A** is considered acceptable when higher than 1.5, while S/N greater than 5 concluded binding. S/N greater than 12 highlighted an excellent binding confidence.

		MST				NMR
Compound	Nucleotide	S/N < 5	5 < S/N <12	S/N> 12	A> 1.5	
**3**	GppNHp	Y	X		XY	X
	GDP	X			X	X
**10**	GppNHp		XY		XY	XY
	GDP			X	X	XY
**11**	GppNHp	XY			X	
	GDP	X				
**12**	GppNHp	Y	X		X	X
	GDP		X		X	X
**14**	GppNHp		XY		XY	X
	GDP	X			X	X
**16**	GppNHp	XY			XY	
	GDP	X			X	
**17** (ARN22089)	GppNHp			XY	XY	X

	GDP		X		X	X
**22**	GppNHp		XY		XY	X
	GDP	X			X	X
**23**	GppNHp	XY			X	
**23**	GDP	X			X	
**24**	GppNHp	XY				
	GDP	X				
**25**	GppNHp	Y		X	XY	X
	GDP		X		X	X
**27** (ARN25062)	GppNHp	X	Y		XY	X
	GDP		X		X	X
**28** (ARN24928)	GppNHp	X	Y		XY	XY
	GDP	X			X	XY

4. Supplementary Figure S1

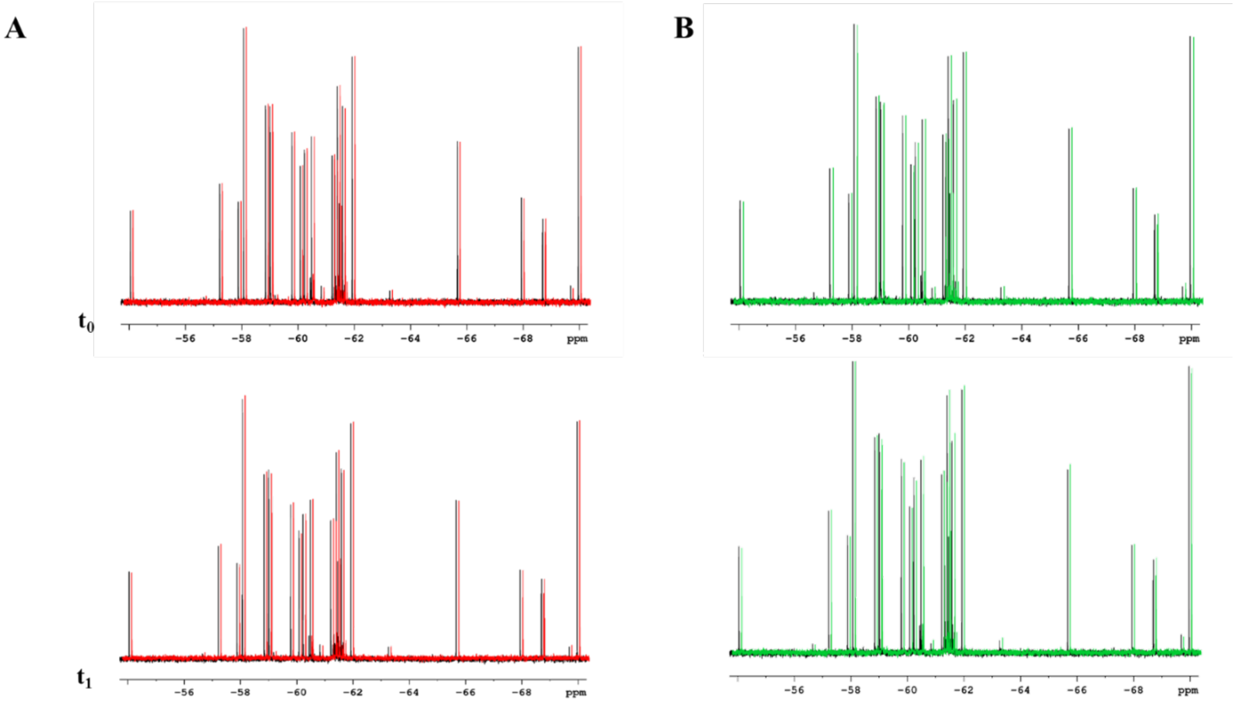

^{19}F T_2 filter spectra of a 20 μM of a mixture of 25 CF_3 fragments in absence (A and B, black) and in presence of 2 μM CDC42 loaded with GppNHp (A, red) and 2 μM Cdc42 loaded with GDP (B, green) registered just after preparation (t_0, top) and after 24 hours (t_1, bottom). No difference in the ^{19}F NMR signals of the compounds are visible after the protein addiction and after 24h, indicating that no diffused/aspecific binding are present after protein addition and also that the proteins are stable for at least 24 hours.

5. Figure S2

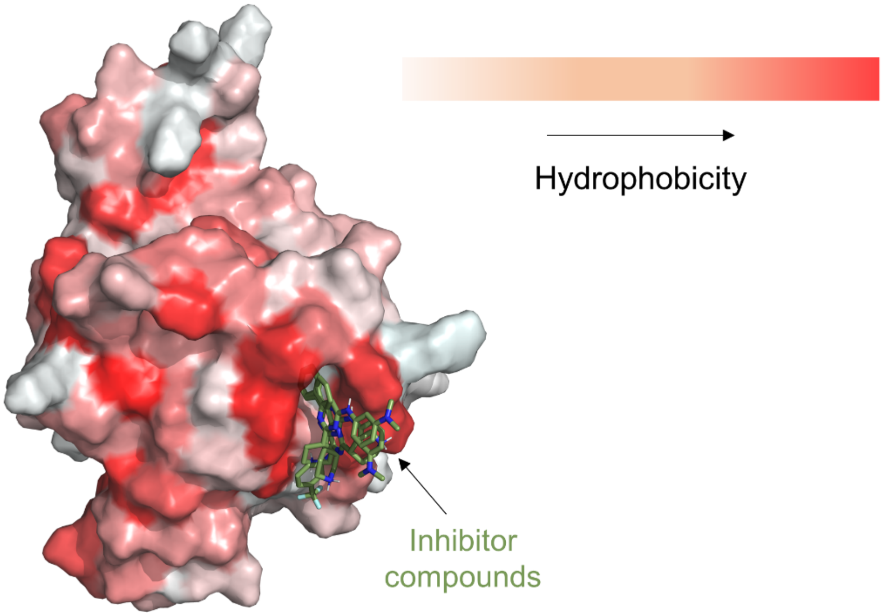

The phenyl on C6 represents the predicted anchor point of the reported set of compounds. The model structure of compounds **17** (ARN22089-lead compound), and two backups **27** (ARN25062) and **28** (ARN24928) bound to CDC42 is reported. The structure of CDC42 is represented as surface while the inhibitor compounds are shown as green sticks. The color scheme used to represent the protein surface is based on the Eisenberg hydrophobicity scale.[1] As shown, the hydrophobic nature of the allosteric drug-binding pocket smoothly accommodates the phenyl on position C6, which represents the anchor point in all the reported docking poses.

6. Figure S3

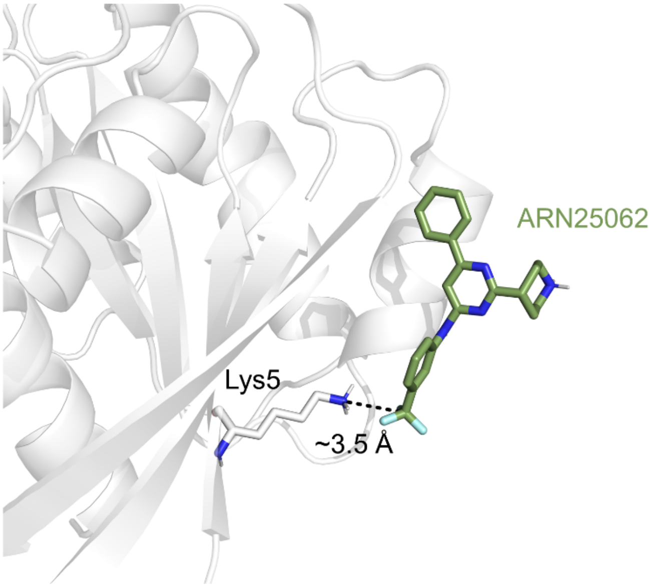

Lys5 could determine ARN25062 binding mode to the CDC42 effector pocket. The model structure of compound **27** (ARN25062) compound bound to the allosteric drug-binding pocket of CDC42 is reported. The structure of CDC42 is represented as white cartoon while **27** − ARN25062 and the close Lys5 residue are reported as green and white sticks, respectively. The observed inverted arrangement of pyridine core could be due to the attraction between the positive and the negative charges of the amino and trifluoromethyl group present in Lys5 and **27** − ARN25062, respectively, whose centers of mass lay ~3.5 Å apart from each other.

7. *In vitro* metabolic stability

10mM DMSO stock solution of test compound was pre-incubated at 37˚C for 15min with mouse liver microsomes in0.1M Tris-HCl buffer (pH 7.4) with 10% DMSO. The final concentration was 4.6µM. After pre-incubation, the co-factors (NADPH, G6P, G6PDH and $MgCl_2$ pre-dissolved in 0.1M Tris-HCl) were added to the incubation mixture and the incubation was continued at 37˚C for 1h. At each time point (0, 5, 15, 30, 60min), 30µL of incubation mixture was diluted with 200µL cold CH_3CN spiked with 200nM of an appropriate internal standard, followed by centrifugation at 3.270 x g for 15min. The supernatant was further diluted with H_2O (1:1) for analysis. A reference incubation mixture (microsomes *without* cofactors) was prepared for each test compound and analyzed at t = 0 and 60min in order to verify the compounds stability in the matrix. The two time points were diluted as for the time points of the incubation mixture above. The concentration of test compound was quantified by LC-MS/MS on a Waters ACQUITY UPLC-MS system consisting of a Triple Quadrupole Detector (TQD) Mass Spectrometer equipped with an Electrospray Ionization interface and a Photodiode Array Detector from Waters Inc. (Milford, MA, USA). The analyses were run on an ACQUITY UPLC BEH C_{18} (50x2.1mmID, particle size 1.7µm) with a VanGuard BEH C_{18} pre-column (5x2.1mmID, particle size 1.7µm) at 40°C, using 0.1% HCOOH in H_2O (A) and 0.1% HCOOH in CH_3CN (B) as mobile phase. Electrospray ionization was applied in positive mode. Compound-dependent parameters as MRM transitions and collision energy were developed for each compound. The percentage of test compound remaining at each time point relative to t=0 was calculated by the response factor on the basis of the internal standard peak area. The percentage of test compound versus time was plotted and fitted by GraphPad Prism (GraphPad Software, Version 5 for Windows, CA, USA, www.graphpad.com) to estimate the compounds half-life ($t_{½}$) which was reported as mean value along with the standard deviation (*n = 3*).

8. Aqueous kinetic solubility

The aqueous kinetic solubility was determined from a 10mM DMSO stock solution of test compound in Phosphate Buffered Saline (PBS) at pH 7.4. The study was performed by incubation of an aliquot of 10mM DMSO stock solution in PBS (pH 7.4) at a target concentration of 250µM resulting in a final concentration of 2.5% DMSO. The incubation was carried out under shaking at 25°C for 24h followed by centrifugation at 21.100 x g for 30min. The supernatant was further

diluted (4:1) in CH_3CN and analyzed by -LC-MS for the quantification of dissolved compound by UV at 215nm. The analyses were performed on a Waters ACQUITY UPLC-MS system consisting of a Single Quadrupole Detector (SQD) Mass Spectrometer equipped with Electrospray Ionization interface and a Photodiode Array Detector from Waters Inc. (Milford, MA, USA). The analyses were run on an ACQUITY UPLC BEH C_{18} column (50x2.1mmID, particle size 1.7µm) with a VanGuard BEH C_{18} pre-column (5x2.1mmID, particle size 1.7µm), using 10mM NH_4OAc in H_2O at pH 5 adjusted with AcOH (A) and 10mM NH_4OAc in CH_3CN-H_2O (95:5) at pH 5 (B) as mobile phase. The aqueous kinetic solubility (in µM) was calculated by dividing the peak areas of dissolved test compound and test compound in the reference (250µM of test compound in CH_3CN) and multiply by the target concentration and dilution factor.

9. Pharmacokinetic studies

Animal models:

Male C57B6/J mice, 8 weeks old were used (Charles River). All procedures were performed in compliance with the Ethical Guidelines of European Communities Council (Directive 2010/63/EU of 22 September 2010) and accepted by the Italian Ministry of Health. All efforts were made to minimize animal suffering and to use the minimal number of animals required to produce reliable results, according to the "3Rs concept". Animals were group-housed in ventilated cages and had free access to food and water. They were maintained under a 12-hour light/dark cycle (lights on at 8:00 am) at controlled temperature (21°C ± 1°C) and relative humidity (55% ± 10%).

Animal treatment:

27 − ARN25062 and **28** − ARN 24928 was administered P.O. and I.V. to C57BL/6 male mice at 10 and 3 mg/kg. The vehicle used was PEG400/Tween 80/saline solution at 10/10/80% in volume, respectively. Three animals per each time point were treated. Blood samples at 0, 15, 30, 60, 120, 240, and 480 min after administration were collected for the P.O. arm. Blood samples at 0, 5, 15, 30, 60, 120, and 240 min after administration were collected for the I.V. arm. Plasma was separated from blood by centrifugation for 15 min at 1500 rpm a 4 °C, transferred to Eppendorf tubes, and frozen (−80 °C). Control animals treated with vehicle only were also included in the experimental protocol.

Pharmacokinetic measurements:

Plasma samples were centrifuged at 21.100 x g for 15min at 4°C. An aliquot of each plasma sample was extracted (1:3) with cold CH_3CN containing 200nM of an appropiate internal standard. A calibration curve was prepared in naïve mouse plasma over a 1nM – 10µM range. Three quality control samples were prepared by spiking the parent compound in naïve mouse plasma to 20, 200 and 2000nM as final concentrations. The calibrators and quality control samples were extracted (1:3) with the same extraction solution as the plasma samples. The plasma samples, calibrators and quality control samples were centrifuged at 3.270 x g for 15min at 4°C. The supernatants were further diluted (1:1) with H_2O, and analyzed by LC-MS/MS on a Waters ACQUITY UPLC-MS system consisting of a Triple Quadrupole Detector (TQD) Mass Spectrometer equipped with an Electrospray Ionization interface and a Photodiode Array Detector from Waters Inc. (Milford, MA, USA). Electrospray ionization was applied in positive mode. Compound-dependent parameters as MRM transitions and collision energy were developed for the parent compound and the internal standard. The analyses were run on an ACQUITY UPLC BEH C_{18} (50x2.1mmID, particle size 1.7µm) (for ARN25062 and ARN24928) and ACQUITY UPLC BEH C_{18} (100x2.1mmID, particle size 1.7µm) (for ARN22089) both with a VanGuard BEH C_{18} pre-column (5x2.1mmID, particle size 1.7µm) at 40°C, using H_2O + 0.1% HCOOH (A) and CH_3CN + 0.1% HCOOH (B) as mobile phase. All samples (plasma samples, calibrators and quality controls) were quantified by MRM peak area response factor in order to determine the levels of the parent compound in plasma. The plasma concentrations versus time were plotted and the profiles were fitted using PK Solutions Excel Application (Summit Research Service, USA) in order to determine the pharmacokinetic parameters.

REFERENCES

1. Eisenberg, D.; Schwarz, E.; Komaromy, M.; Wall, R., Analysis of membrane and surface protein sequences with the hydrophobic moment plot. *J Mol Biol* **1984,** *179* (1), 125-42.
